# Photoactive Neutral Three-Coordinate Cu(I) Complexes of Anionic N‑Heterocyclic Carbenes

**DOI:** 10.1021/jacsau.5c00357

**Published:** 2025-06-09

**Authors:** Lars E. Burmeister, Lucie J. Groth, Philipp R. Meinhold, Johannes P. Zurwellen, Dirk Bockfeld, René Frank, Michael Karnahl, Matthias Tamm, Stefanie Tschierlei

**Affiliations:** † Department of Energy Conversion, Institute of Physical and Theoretical Chemistry, 26527 Technische Universität Braunschweig, Rebenring 31, 38106 Braunschweig, Germany; ∥ Institute of Inorganic and Analytical Chemistry, 26527 Technische Universität Braunschweig, Hagenring 30, 38106 Braunschweig, Germany

**Keywords:** three-coordinate Cu(I) complexes, anionic N-heterocyclic carbenes (NHCs), thermally activated delayed fluorescence (TADF), high triplet energy photosensitizers, TDDFT calculations, photocatalysis

## Abstract

Three-coordinate Cu­(I) complexes are promising candidates for photoactive compounds, but their application in photocatalysis remains largely unexplored. Here, we report the synthesis and comprehensive characterization of four novel three-coordinate Cu­(I) complexes featuring an anionic N-heterocyclic carbene ligand with a weakly coordinating tris­(pentafluorophenyl)­borate moiety (WCA-NHC) and different methyl substituted dipyridylamine-based N,N′-ligands. This ligand design significantly improves the stability and photophysical properties of these complexes in solution. Steady-state and time-resolved spectroscopy, electrochemical measurements, temperature-dependent emission studies and quantum chemical calculations were used to elucidate the electronic and excited-state properties of these complexes. Our results demonstrate metal-to-ligand charge transfer absorption and thermally activated delayed fluorescence (TADF), leading to extended excited-state lifetimes (up to 8.6 μs) and high excited-state energies (≈2.7 eV). All four complexes efficiently photosensitize the norbornadiene-to-quadricyclane photoisomerization, a key reaction for molecular solar thermal energy storage (MOST). By demonstrating that careful ligand selection allows the design of three-coordinate Cu­(I) complexes with excellent photophysical and photocatalytic properties, this study expands the scope of Cu­(I) photosensitizers and lays the foundation for further applications in photochemistry.

## Graphical abstract

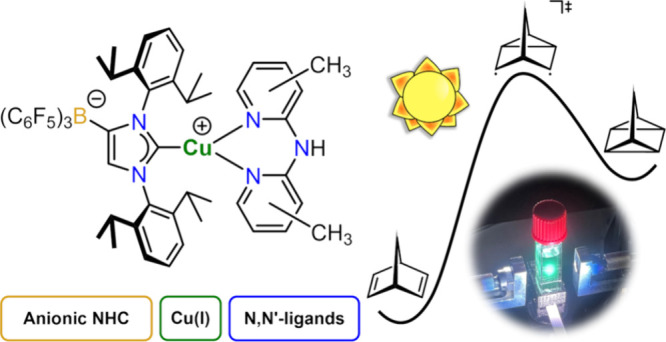

## Introduction

Over the past decades, photoactive Cu­(I) complexes have garnered significant interest for their successful applications in organic light-emitting diodes (OLEDs),^1–3^ dye-sensitized solar cells (DSSCs),^1,4,5^ and photocatalysis.^6–8^ The Cu­(I) center is well suited for such applications due to its d^10^ electron configuration (completely filled d-shell), which minimizes the involvement of non-radiative metal-centered (MC) states.^9–11^ In addition, the high natural abundance of copper (5·10^–3^ mass-%) in the Earth’s crust makes Cu­(I) compounds a cost-effective and more sustainable alternative to traditional complexes based on rare and precious metals, such as ruthenium (1·10^–6^ mass-%) or iridium (1·10^–7^ mass-%).^12,13^ Moreover, Cu­(I) complexes have been shown to undergo thermally activated delayed fluorescence (TADF), which enables the harvesting of singlet and triplet excitons and theoretically allows for an internal quantum efficiency of 100% for OLED technologies.^14–16^

Since the pioneering work of McMillin and Sauvage in the 1980s,^17,18^ extensive research has focused on four-coordinate tetrahedral Cu­(I) complexes bearing diimine and/or diphosphine ligands.^7,10,19^ Two-coordinate Cu­(I) complexes with a linear coordination geometry have recently emerged as a valuable class of luminescent dyes, owing to their low non-radiative decay rates and, consequently, high emission quantum yields (ϕ_em_).^2,20–24^ Three-coordinate Cu­(I) compounds, on the other hand, have attracted increasing interest for their excellent photophysical properties in the solid state, making them promising candidates for lighting technologies or telecommunication.^3,25–27^ Yet, their photophysical and photocatalytic properties in solution remain largely unexplored, likely due to limited stability.

A recent study by Grudzien et al. demonstrated that the three-coordinate [1,10-phenanthroline-Cu-IDipp]­PF_6_ complex (IDipp = 1,3-bis­(2,6-diisopropylphenyl)-imidazolin-2-ylidene) can participate in photoinduced energy and electron transfer reactions,^28^ underlining its potential as a viable alternative to its four-coordinate counterparts. However, this study only provided preliminary insights into the underlying structure–activity relationships. Just recently, Teets and co-workers reported three-coordinate Cu­(I) complexes bearing β-diketiminate and isocyanide ligands as a new class of promising photocatalysts.^29,30^ Thus, the development and detailed investigation of solution-stable, three-coordinate Cu­(I) complexes with enhanced photophysical properties offers substantial potential to expand the range of Earth-abundant photosensitizers, particularly for applications in photocatalysis.

A promising strategy to stabilize three-coordinate complexes is the incorporation of bulky, strongly σ-donating and (weakly) π-accepting ligands such as N-heterocyclic carbenes (NHCs). Previous reports of luminescent three-coordinate Cu­(I)-NHC complexes include both cationic and neutral species, featuring either an anionic chelate or an anionic carbene ligand (cf. Figure 1).^31–38^ While cationic Cu­(I)-NHC complexes containing N-donor ligands exhibit high emission quantum yields of up to 88% in the solid state,^34^ their quantum yields in solution remain below 0.1%, except for those with dicarbene CNC-pincer ligands.^39,40^ In contrast, neutral Cu­(I) complexes with anionic N,N′-ligands, first introduced by Thompson and co-workers,^31^ exhibit considerably higher ϕ_em_ in solution, reaching up to 17% in cyclohexane.^32^ However, these systems differ significantly in ligand architecture, limiting direct comparability.

**Figure 1:**
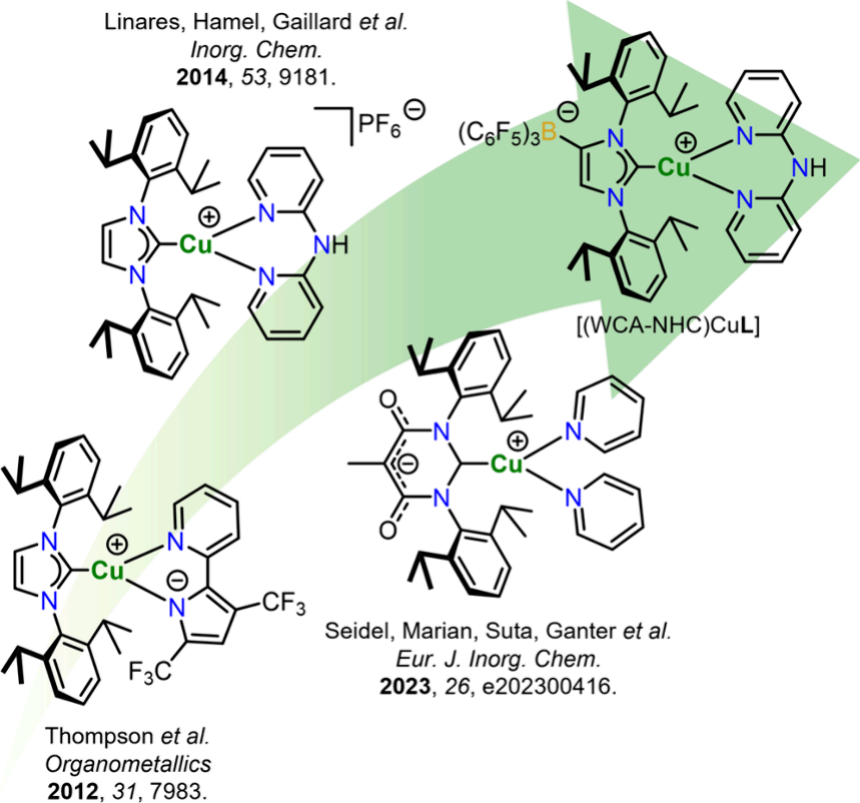
Overview of selected photoactive three-coordinate Cu­(I)-complexes described in the literature^32,34,36^ in comparison to the novel approach investigated herein using an anionic NHC with a weakly coordinating tris­(pentafluorophenyl) borate moiety (WCA-NHC).

To date, only one recent study has reported neutral Cu­(I) complexes containing an anionic NHC, as described by Seidel, Marian, Suta, Ganter, and co-workers in 2023 (Figure 1).^36^ Although these complexes are emissive in the solid state, a detailed study of their properties in solution was not feasible due to rapid ligand dissociation.

In this work, we introduce a novel strategy for developing stable, neutral, three-coordinate Cu­(I)-NHC complexes. Building on previous work by Linares, Hamel, Gaillard, and co-workers (Figure 1), which demonstrated the tunability of MLCT excited states via N,N′-ligand modifications,^34,35,37^ we extend this concept by modifying the ligand environment. Our approach utilizes the dipyridylamine-based ligands **L2-L6** (Figure 2) as chelating ligands, while replacing the carbene ligand with an anionic NHC featuring a weakly coordinating fluoroborate moiety in the backbone (WCA-NHC, Figure 1, top right). In the resulting zwitterionic complexes, the negative charge is located at the borate atom, while the pentafluoro­phenyl rings prevent potentially unfavorable interactions with the copper center. In our hands, these ligands have been widely applied in transition metal and main group element chemistry,^41,42^ including the preparation of complexes with coinage metals (Cu, Ag, Au).^43,44^

**Figure 2:**
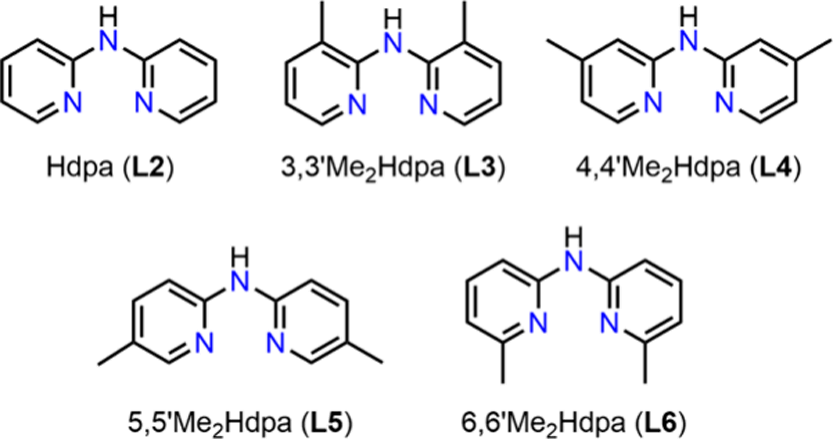
Dipyridylamine-based ligands (**L2**−**L6**) used in this work.

Accordingly, this study presents the synthesis and comprehensive structural characterization of four novel three-coordinate Cu­(I) complexes bearing a WCA-NHC ligand. Their photophysical and electrochemical properties were investigated using a combination of cyclic voltammetry, steady-state and time-resolved absorption/emission spectroscopy, and temperature-dependent emission studies to probe TADF characteristics. Experimental findings were further supported by (time-dependent) density functional theory (TD)­DFT calculations. Finally, the applicability of these complexes as photosensitizers was demonstrated in the light-driven isomerization of norbornadiene (NBD) to quadricyclane (QC), a reaction of particular interest for molecular solar thermal energy storage (MOST) systems.^45–47^

## Results and Discussion

### Synthesis

The Cu­(I) complexes were prepared by the reaction of the copper­(I) complex [(WCA-NHC)­Cu­(toluene)] (**C1**)^44^ with the corresponding N,N′-ligands **L2**-**L5** in toluene solution. Within 2 h, complexes **C2**-**C5** were formed by stirring at room temperature under argon atmosphere (Scheme 1). The volatiles were removed, and after washing with toluene, the expected complexes **C2**-**C5** were isolated in moderate to good yields of 52–86%. This approach, similar to that of Seidel, Marian, Suta, Ganter and co-workers^36^ via a coordination polymer of an anionic carbene and Cu­(I), prevents the exchange of an anion for the N-donor ligand, allowing easier coordination to the copper center and subsequent workup without the need for salt removal. The characteristic signal for the carbene carbon is observed in the ^13^C­{^1^H} NMR at 181.8–182.8 ppm in deuterated tetrahydrofuran (THF-*d*_8_, Figures S13, S20, S27, and S34), which is in the same range as for the cationic complexes.^34^ In a reaction of the most sterically demanding N,N′-ligand **L6** with complex **C1**, the linear heteroleptic complex **C6** was isolated instead of a structurally similar three-coordinate complex (Scheme 1 bottom). In this complex, the deprotonated secondary amine coordinates to the Cu­(I) center and one nitrogen atom of the two pyridyl rings is protonated. This is in contrast to the ligand scrambling observed for the corresponding cationic complexes, where homoleptic complexes were found,^34^ but it underscores the observation that **L6**, in combination with the functionalized IDipp ligand, is sterically too demanding to form trigonal complexes. The structure and purity of the Cu­(I) complexes **C2–C6** were verified by ^1^H, ^11^B­{^1^H}, ^13^C­{^1^H} and ^19^F­{^1^H} nuclear magnetic resonance (NMR) spectroscopy, single-crystal X-ray diffraction (scXRD), and elemental analysis (see SI for further details).

**Scheme 1:**
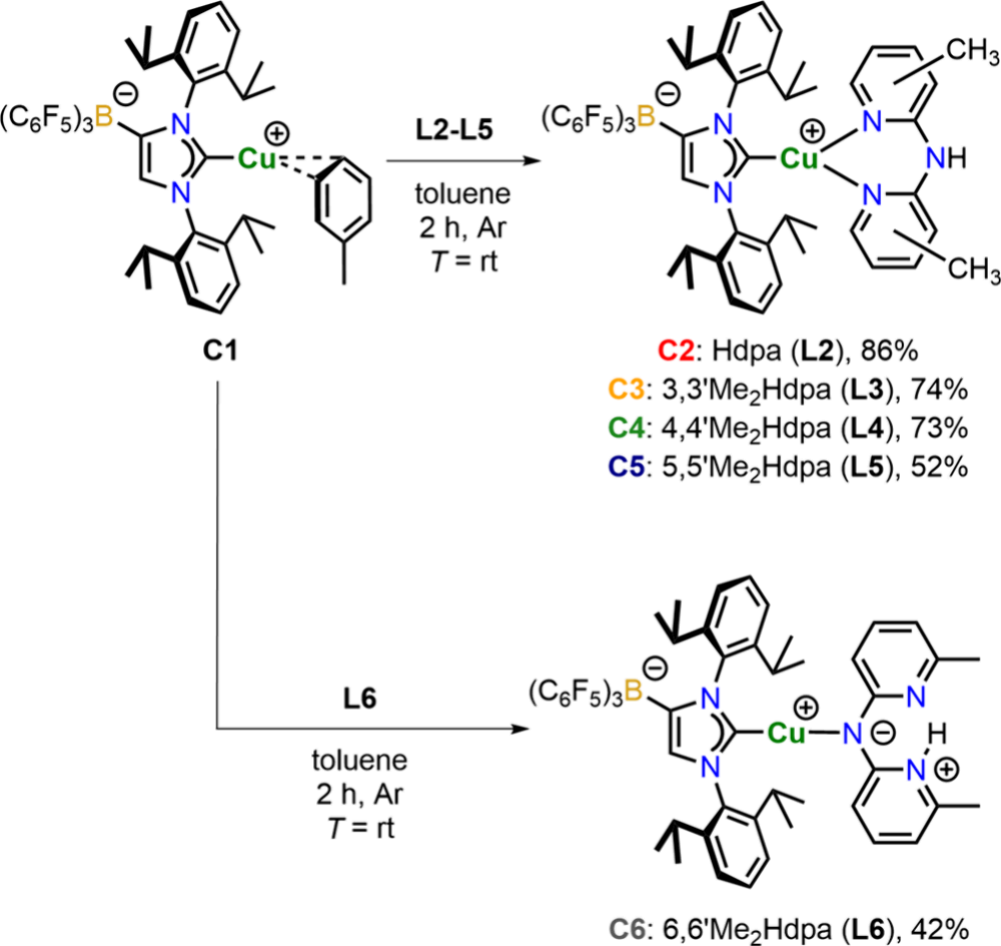
Synthetic Route Towards the Complexes **C2**–**C6**

### Structural Characterization

Single crystals suitable for scXRD were obtained from THF or toluene solutions by layering with *n*-pentane or *n*-hexane (Figure 3). During the crystallization of **C2**, three different structures were obtained (**C2-I**, **C2-II** and **C2-III**, Tables S1–S3). **C2-I** and **C2-II** were derived from two separate crystals of the same crystallization experiment, in which a toluene solution was layered with *n*-pentane. In contrast, **C2–III** was obtained by layering a THF solution with *n*-pentane.

**Figure 3:**
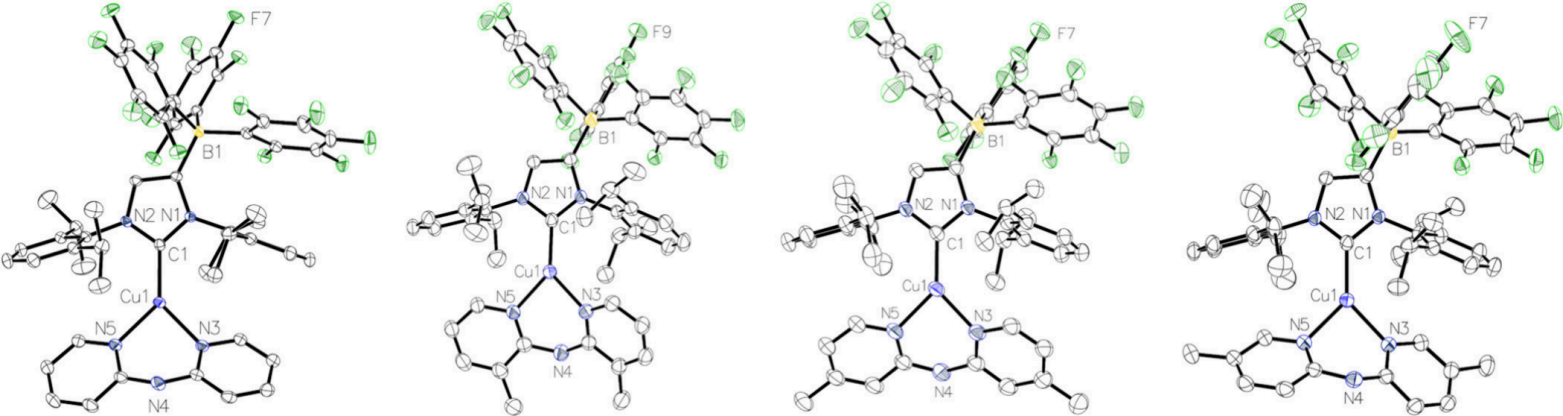
Molecular structures of the complexes **C2**–**C5** with 50% probability displacement ellipsoids. All hydrogen atoms and co-crystallized solvents are omitted for clarity. Carbon atoms are shown in white, nitrogen in blue, boron in yellow, fluorine in green, and copper in light blue.

**C2–I** and **C2–III** display significantly different structural parameters, in particular in terms of plane and twist angles (Figure S46). The plane angles range from 49.14° to 22.3° (Table 1). For **C2–II**, the data quality is insufficient for detailed interpretation of the bonding parameters, but the structure suggests a considerably different twist angle of approximately 40°, while maintaining a large plane angle. These findings indicate that the ligand orientation is primarily influenced by packing effects in the solid state and that the energy barriers between the different conformers are relatively low.

**Table 1:** Selected Bond Lengths (in pm) and Angles (in °) of the Complexes **C2** –**C6** Obtained from scXRD Measurements (exp.) and DFT Calculations (calc.)^a^

			Cu1–N3		C1–Cu1–N3		
Compound		Cu1–C1	Cu1–N5	N3–Cu1–N5	C1–Cu1–N5	Plane Angle	Twist Angle
**C2-I**	exp.	190.85(15)	204.73(13)	89.78(5)	134.99(6)	49.14(17)	5.39(9)
			203.89(12)		134.01(6)		
**C2-III**	exp.	192.68(16)	208.0(2)	88.63(8)	134.68(9)	22.3(2)	4.46(11)
			206.74(18)		136.68(9)		
	calc.	191.76	207.31	87.87	136.47	45.77	14.80
			207.68		134.63		
**C3**	exp.	190.90(18)	204.82(15)	88.89(7)	135.36(7)	45.72(16)	15.53(9)
			204.27(18)		135.16(6)		
	calc.	191.84	206.71	87.63	137.07	44.97	14.99
			208.41		134.52		
**C4**	exp.	191.02(19)	204.19(13)	88.60(6)	136.00(7)	46.23(13)	7.58(8)
			205.92(17)		134.29(6)		
	calc.	191.70	206.91	87.79	136.61	45.64	15.33
			207.43		134.61		
**C5**	exp.	190.48(15)	206.29(13)	89.25(6)	136.46(7)	49.08(16)	12.31(8)
			203.42(19)		133.60(8)		
	calc.	191.62	207.08	87.76	135.69	50.12	18.06
			207.63		135.34		
**C6**	exp.	187.89(17)	190.23(15)		177.53(7)		

a Comparison of the bond lengths of copper and the ligands (Cu–C and Cu–N), the bite angle of the N,N′-ligand (N–Cu–N), the angle defined by the carbene, copper, and N,N′-ligand (C–Cu–N), and the plane and twist angles between the imidazole ring plane and the plane spanning the three nitrogen atoms of the N,N′-ligand.

The neutral complexes **C2**-**C5** exhibit similar bond lengths and angles (Table 1), which are comparable to those observed in the cationic complexes.^34^ The Cu–C_carbene_ bond lengths range from 190.48(15) to 192.68(16) pm, closely matching those in the cationic counterparts (190.2(2)−192.08(18) pm). The Cu–N bond lengths vary between 203.42(19)−208.0(2) pm, again aligning with the charged systems (203.7(2)-206.5(4) pm). The bite angles of 88.60(6)-89.78(5)° are slightly smaller than those observed for the cationic complexes (89.62(7)-91.04(9)°), but remain close to 90°. The sum of the angles around the copper center is approximately 360° in both the neutral and charged systems, confirming a trigonal planar geometry. These structural similarities highlight the minimal impact of introducing a weakly coordinating borate moiety in the ligand backbone, making these complexes well-suited for direct comparison with their structurally related cationic congeners.

In complex **C6**, the carbene and the amide ligands adopt a nearly linear arrangement and show bonding parameters similar to those of previously reported NHC-stabilized amide complexes (Figure 4). The Cu1–C1 bond length of 187.89(17) pm falls within the reported range of (185.0(6)-189.7(5) pm).^48,49^ The Cu1–N3 bond is 190.23(15) pm, placing it as the longest reported Cu–N bond length compared to related amides complexes (183.7(4)-188.0(4) pm).^48,50^ This elongation is expected, as the ligand charge is overall neutral rather than anionic.

**Figure 4:**
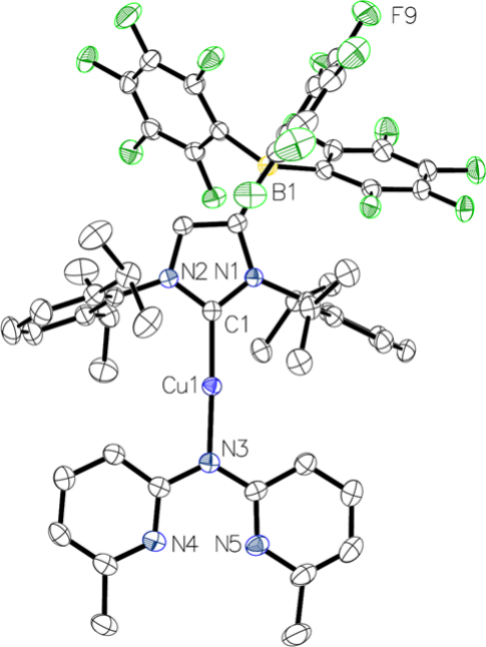
Molecular structure of complex **C6** with 50% probability displacement ellipsoids. All hydrogen atoms and co-crystallized solvents are omitted for clarity.

To validate the method applied for the quantum chemical calculations, the optimized geometries (PBE0-D3­(BJ)/def2-TZVP, CPCM­(THF); see SI) were compared with the experimental molecular structures (Table 1). In general, the DFT calculations predict slightly longer Cu–C and Cu–N bond lengths. In addition, the calculated bite angles of the N,N′-ligands tend to be slightly smaller than the experimentally observed angles. The plane and twist angles show good agreement with the experimentally observed values, e.g. **C5** exp. 49.08(16)° and 12.31(8)° vs. calc. 50.12° and 18.06°. Minor discrepancies can be attributed to packing effects in the crystal structures. Overall, the optimized geometries closely match the experimental structures, confirming the reliability of the quantum chemical methods used.

### Molecular Orbitals

To gain deeper insights into the electronic structure of the complexes, the molecular orbitals were determined by DFT calculations using the validated method mentioned above. The HOMOs of the complexes (Figure 5 top and SI Chapter 6) are primarily localized on the copper center, with additional contributions from the N-donor orbitals of the N,N′-ligand and the π-orbitals of the imidazole ring. In contrast, the LUMO is solely localized on the N,N′-ligand. However, the introduction of the methyl substituents (**C3**-**C5**) has little effect on the HOMO energies (cf. Figure 5 bottom). Conversely, the LUMO energies are strongly influenced by the introduction of electron-donating methyl groups. According to the DFT calculations, the +I-effect of the methyl groups causes an increase of the orbital energies from **C2** (0.00 eV) to **C3** (+0.07 eV) and **C5** (+0.07 eV). The greatest destabilization is observed at **C4** (+0.12 eV), where the methyl substituents are introduced in the 4,4′-positions. This trend can be rationalized by analyzing the contributions of the atomic orbitals in the different positions of the unsubstituted complex **C2** (cf. Table S8 and Figure S54).^51–53^ The highest orbital contribution to the LUMO was found for the atomic orbitals of the carbon atoms in 4,4′-positions (8.0% and 13.8%), while the contributions of the carbon atoms in 5,5′-positions (5.6% and 6.6%) and 3,3′-positions (2.5% and 4.4%) were significantly lower. Thus, electronic communication is predicted to be strongest when the methyl substituents are attached at the 4,4′-positions.

**Figure 5:**
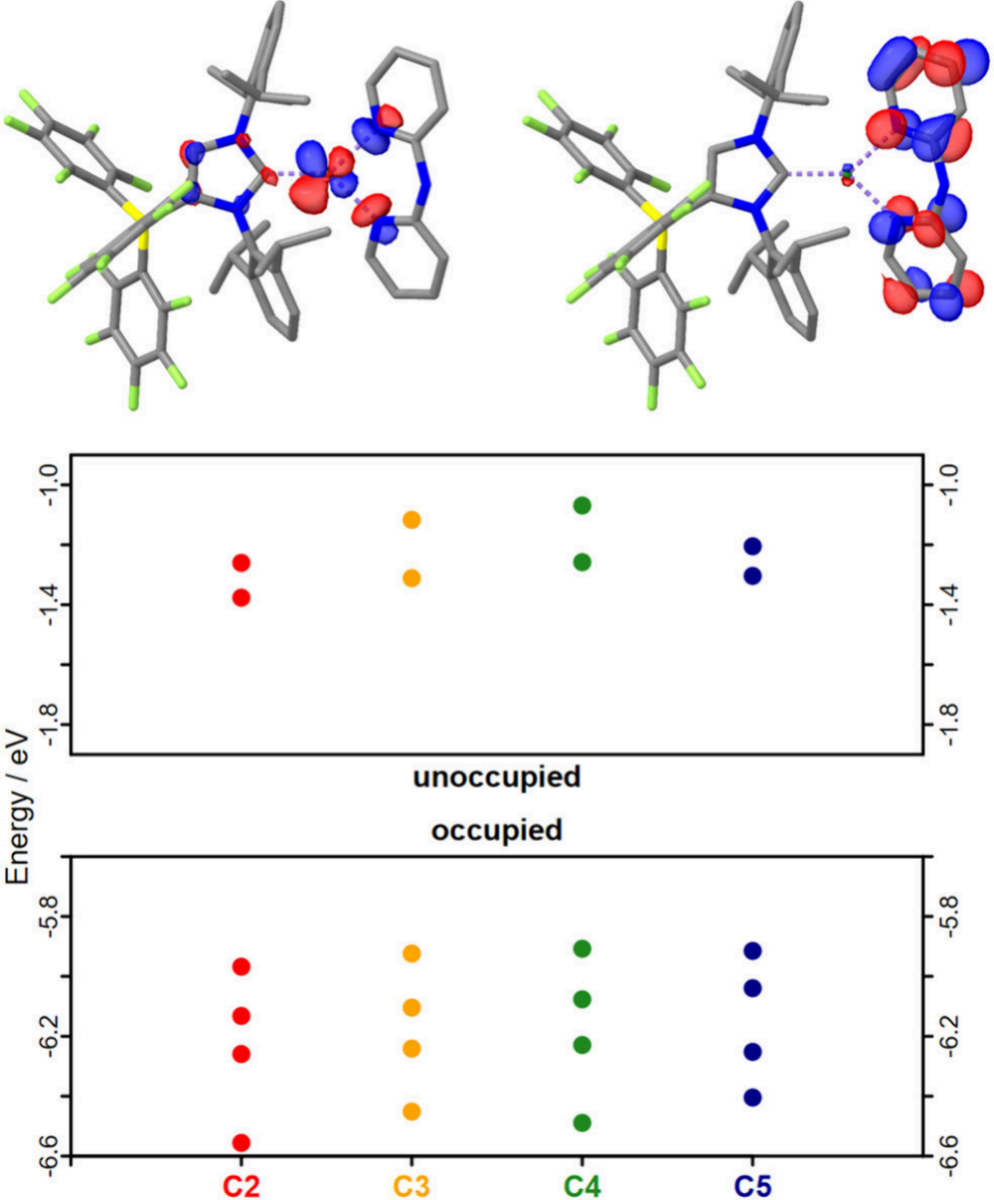
Top: Representation of the HOMO (top left) and LUMO (top right) of complex **C2** obtained from DFT calculations. Volumes are depicted with an isosurface value of 0.05. Bottom: Energy diagram depicting the orbital energies of the LUMO+1 and LUMO and HOMO, HOMO–1, HOMO–2, and HOMO–3 of the complexes (**C2**–**C5**).

### Electrochemical Properties

To examine the orbital energies and validate the trends predicted by the DFT calculations, the electrochemical behavior of the complexes **C2**-**C5** was studied by cyclic voltammetry (Table 2). All complexes are irreversibly oxidized at similar potentials of 0.69–0.79 V. Analysis of the spin densities of the oxidized species, as predicted by DFT calculations, indicates that the oxidation can be attributed to the Cu­(I)/Cu­(II) redox couple (Figures S53, S56, S58, and S60). The observed oxidation potentials and the assignment to a formal oxidation of the metal center are consistent with previously reported cationic complexes by Glinton et al.^37^ Notably, Glinton and co-workers observed a (quasi-)­reversible oxidation.^37^ Furthermore, the complexes exhibit an irreversible reduction at potentials ranging from −2.80 V for **C2** to −3.00 V for **C4**. Spin density analysis reveals that the singly occupied molecular orbitals (SOMOs) are localized on the respective N,N′-ligands. Thus, the reduction can be attributed to the **L**(−I)/**L**(0) redox couple as also observed for the cationic complexes^34,37^ and aligns well with the observed reduction potentials of Mo- and W-based complexes containing **L2**.^54,55^ The most significant cathodic shift, observed for **C4** (Δ = −0.20 V) compared to the unsubstituted complex **C2**, further supports the enhanced electronic communication between the dipyridylamine moiety and the methyl groups when they are attached at the 4,4′-positions. Overall, the oxidation and reduction potentials of these complexes correspond closely to the trends for the HOMO and LUMO energies derived from the DFT calculations. Furthermore, the observed electrochemical energy gaps ΔE (Table 2) correlate well with the observed optical energy gaps from the UV/vis absorption spectra discussed below.

**Table 2:** Redox Potentials of the Irreversible Reduction (*E*_red_) and Oxidation (*E*_ox_) Events as well as the Electrochemical Gap (Δ*E*) of the Complexes **C2** –**C5** in Tetrahydrofuran Solution (Inert) Containing [Bu_4_N]­[PF_6_] (*c* = 0.1 M) as the Supporting Electrolyte^a^

	*E*_red_/V	*E*_ox_/V	Δ*E*/V
**C2**	–2.80	0.69	3.49
**C3**	–2.89	0.75	3.64
**C4**	–3.00	0.79	3.79
**C5**	–2.91	0.73	3.64

a All values are referenced against the ferrocene/ferrocenium (Fc/Fc^+^) redox couple.

### Photophysical Properties

Upon UV irradiation, the colorless Cu­(I) complexes exhibit deep green emission in solution. To explore their photophysical properties in detail, **C2**-**C5** were analyzed using a combination of steady-state and time-resolved spectroscopic techniques in tetrahydrofuran under inert conditions. The experimental findings were further supported by TDDFT calculations (see SI Chapter 7).

The absorption spectra of the Cu­(I) complexes exhibit two distinct absorption maxima in the UV region at approximately 260 and 310 nm (Figure 6). These maxima closely correspond to the absorption bands of the N,N′-ligands **L2**-**L5** (Figure S73) and are attributed to ligand-centered (LC) π* ← π transitions. In addition, the absorption spectra of the complexes **C2**-**C5** reveal a shoulder above 325 nm, which is absent in the ligand spectra. This band is attributed to π* ← d metal-to-ligand charge transfer (MLCT) transitions, which is supported by the TDDFT calculations (see SI Chapter 7). Furthermore, this characteristic absorption is in good agreement with the absorption profiles of the structurally related cationic complexes.^34,35,37^ This suggests that the WCA-NHC ligand has only a minor influence on the absorption characteristics.

**Figure 6:**
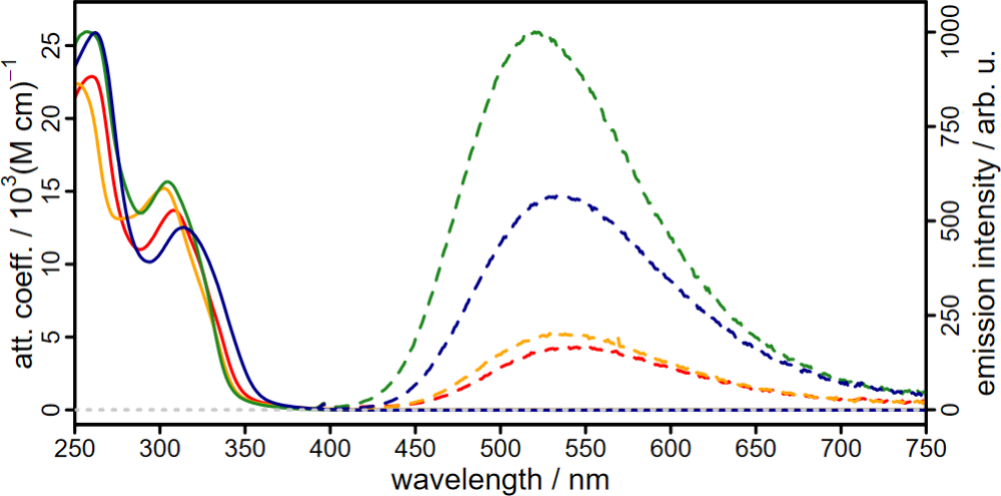
UV/vis absorption spectra (solid lines) and emission spectra (dashed lines) of the complexes **C2** (red), **C3** (orange), **C4** (green), and **C5** (blue) in inert tetrahydrofuran solution excited at 355 nm.

The emission quantum yields again follow the general trend (Table 3), increasing from the unsubstituted complex **C2** (1.8%) to **C3** (2.2%), **C5** (6.6%), and reaching the highest value for **C4** (11.5%). A similar trend was found for the emission lifetimes of the complexes (Table 3), which range from 2.9 to 8.6 μs, providing an excellent basis for applications in photocatalysis.^56,57^ The radiative and non-radiative decay rate constants (*k*_r_ and *k*_nr_) were estimated (Table 3, see SI Equations S2 and S3), revealing a steady increase in *k*_r_ from **C2** to **C5**, while *k*_nr_ is lowest for **C4**. This trend can be rationalized by electronic effects that stabilize the transient excited states.

**Table 3:** Photophysical Properties Including the Radiative (*k*_r_) and Non-Radiative (*k*_nr_) Rate Constants and the Excited-State Energies of the First Excited Singlet (*E*_S1_) and Triplet (*E*_T1_) States of the Complexes (**C2**–**C5**) in Inert Tetrahydrofuran Solution (Ar Atmosphere)^a^

	λ_abs_/nm (ε/10^3^ M^–1^cm^–1^)	λ_em_/nm^b^	ϕ_em_/%^b^	τ_em_/μs^b^	*k*_r_/10^3^ s^–1^	*k*_nr_/10^5^ s^–1^	*E*_S1_/eV	*E*_T1_/eV	Δ*E*_S1‑T1_/eV
**C2**	260 (22.9), 308 (13.7)	544	1.8	2.9	6.2	3.4	2.71	2.65	0.06
**C3**	252 (22.4), 303 (15.2)	529	2.2	2.6	8.5	3.8	2.74	2.65	0.09
**C4**	257 (26.0), 304 (15.7)	520	11.5	8.6	13.4	1.0	2.79	2.73	0.06
**C5**	262 (25.9), 314 (12.5)	533	6.6	4.3	15.3	2.2	2.76	2.69	0.07

a The respective energy gaps (Δ*E*_S1‑T1_) were obtained from temperature dependent emission lifetime measurements.

b Excitation wavelength (λ_exc_) = 355 nm.

DFT calculations and reduction potential analyses indicate that the strongest electronic communication occurs at the 4,4′-positions of the N,N′-ligand. The MLCT transition can be interpreted as a formal reduction of the N,N′-ligand, where the methylpyridine moiety stabilizes the resulting radical species more effectively than the unsubstituted pyridine. Among the substituted ligands, the 4,4′-methyl groups yield the most stabilized radical species, resulting in the highest quantum yields and longest emission lifetimes for **C4**. This is further supported by spin density analyses of the relaxed T_1_ states of the respective complexes. The spin densities were found to be delocalized over the Cu-**L** moiety, with a relatively larger contribution in the 4- and 4′-positions compared to the 3,3′- and 5,5′-positions (Figures S66, S68, S70, and S72). Importantly, the observed emission quantum yields and emission lifetimes are among the highest reported for three-coordinate Cu­(I) carbene complexes in solution.^40^ In contrast, related cationic complexes exhibit no emission in solution,^38^ suggesting that excited state quenching by the anion may play an important role in these compounds. Since the introduction of the tris­(pentafluorophenyl)­borate moiety induces only minor structural and electronic changes, it is likely that suppressing the excited-state quenching by the anion significantly enhances the emission properties in solution. This hypothesis aligns with the higher quantum yields observed for other neutral complexes bearing anionic bidentate ligands.^31–33^

The presence of a large Stokes shift (about 200 nm) and long emission often suggest phosphorescence, however, the possibility of thermally activated delayed fluorescence (TADF) must also be considered in the excited-state decay processes of these complexes.^35,38,58^ To investigate this, the temperature dependence of the emission properties (λ_em_ and τ_em_) in 2-methyltetrahydrofuran was analyzed. Upon cooling from 300 K, all four complexes exhibit an initial decrease in emission intensity until around 240 K, followed by a subsequent intensity increase (Figures S75–S78). Simultaneously, a bathochromic shift of the emission maxima is observed until the glass transition of the solvent matrix is reached.

Up to 240 K, only minor changes in the excited-state lifetimes are detected, but beyond this point, a pronounced increase occurs. By fitting the temperature-dependent emission lifetimes with a Boltzmann-like equation (Equation S5), the energy separations between the lowest triplet and singlet state (ΔE_S1‑T1_) were determined to be in the range of 0.06 to 0.09 eV (Table 3). These values are well below the 0.37 eV threshold commonly associated with TADF, as defined by Thompson, Yersin and co-workers.^58^ This strongly suggests that at room temperature, the observed excited-state decay is best described as TADF.

Further cooling below the glass transition of the solvent matrix results in a significant hypsochromic shift, accompanied by an increase in both emission intensity and excited-state lifetime. This implies a high degree of geometric relaxation in solution, which is hindered in the glassy matrix. Such relaxation processes of the excited state have been previously discussed for structurally related Au­(I) and Cu­(I) complexes and were attributed to a pseudo Jahn–Teller distortion.^31,32,59,60^ Indeed, TDDFT calculations of the relaxed first excited singlet (S_1_) and triplet (T_1_) states confirmed a substantial molecular distortion from the Y-shaped geometry of the ground state toward a T-shaped geometry in both excited states. This structural change is reflected in the widening of one of the C–Cu–N angles from approximately 135° to 155° (Table S17).

The deep green emission (λ_em_ = 520–544 nm for **C2**-**C5**) further confirms the high energy stored in the excited states of these Cu­(I) complexes. Their excited-state energies were estimated (Table 3) from the steady-state emission spectra at room temperature and the experimentally determined energy gaps (ΔE_S1‑T1_, see SI Chapter 11 for further details). All complexes (**C2**-**C5**) possess high excited-state energies for both their first singlet and triplet excited states. Remarkably, the E_T1_ of these complexes exceeds those of typical four-coordinate homo- and heteroleptic Cu­(I) complexes, as well as of [Ru­(bpy)_3_]^2+^, being on par with Ir­(III)-based sensitizers (Figure 7).^9,61,62^

**Figure 7:**
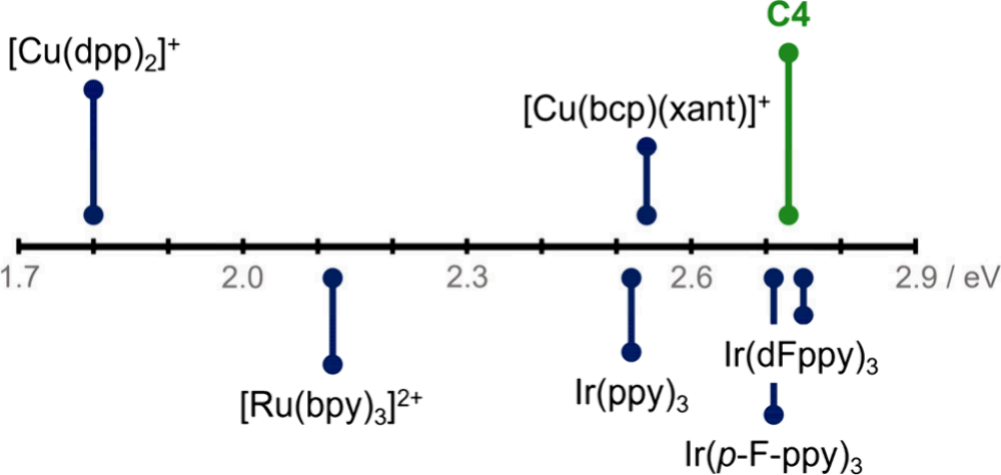
Triplet energies of selected photosensitizers^9,61,62^ in comparison to **C4**. Abbreviations: dpp = 2,9-diphenyl-1,10-phenanthroline, bpy = 2,2’-bipyridine, bcp = 2,9-dimethyl-4,7-diphenyl-1,10-phenanthroline, xant = (9,9-dimethyl-9*H*-xanthene-4,5-diyl)­bis­(diphenylphosphane), ppy = 2-phenylpyridine, *p*-F-ppy = 2-(4-fluorophenyl)-pyridine, dFppy = 2-(2,4-difluorophenyl)-pyridine.

### Photocatalysis

These Cu­(I) complexes exhibit long-lived and high-energy emissions, making them effective as photosensitizers for challenging energy transfer reactions. To evaluate their catalytic potential, the photoinduced [2 + 2] cycloaddition reaction of norbornadiene (NBD) to quadricyclane (QC) was chosen as a proof-of-concept study. This reaction has long been recognized as a potential molecular solar thermal energy storage (MOST) system, because of the sufficiently long half-life of QC and its high energy storage capacity (∼1000 kJ/kg).^45,47,63^ However, direct excitation of NBD results in low conversion due to its poor absorption of visible light.^46,64,65^ To overcome this limitation, various strategies have been explored, utilizing photosensitizers to capture a broader portion of the solar spectrum and to populate the triplet state of NBD (E_T1_ ≈ 2.7 eV) through triplet energy transfer (TET).^66^ However, previous bimolecular systems often relied on either small organic sensitizers^64,67^ or preassociated Cu­(I)-NBD complexes,^68–70^ which absorb only a small fraction of the solar spectrum and lack tunability. Other systems employ expensive noble metal-based photosensitizers, such as Ir­(III) complexes.^71,72^ The Cu­(I)-NHC complexes presented in this study offer an Earth-abundant and tunable alternative to these systems.

Among the investigated complexes, **C4** was initially selected as the primary photosensitizer due to its highest quantum yield (11.5%), longest emission lifetime (8.6 μs), and highest excited-state energy. Emission quenching experiments were conducted to prove energy transfer between **C4** and NBD (see SI Chapter 13). Irradiation of an NMR tube (UV transmission >320 nm) containing **C4** and NBD in THF-*d*_8_ with a Xe arc lamp resulted in a yield of 87% after 2 h (Figure 8). A Xe arc lamp was chosen as the light source to approximate the solar spectrum, which aligns with the concept of molecular solar thermal energy storage. In addition, photostability measurements of **C4** revealed only minor spectral changes under irradiation, suggesting good stability under the applied conditions (Figure S83).

**Figure 8:**
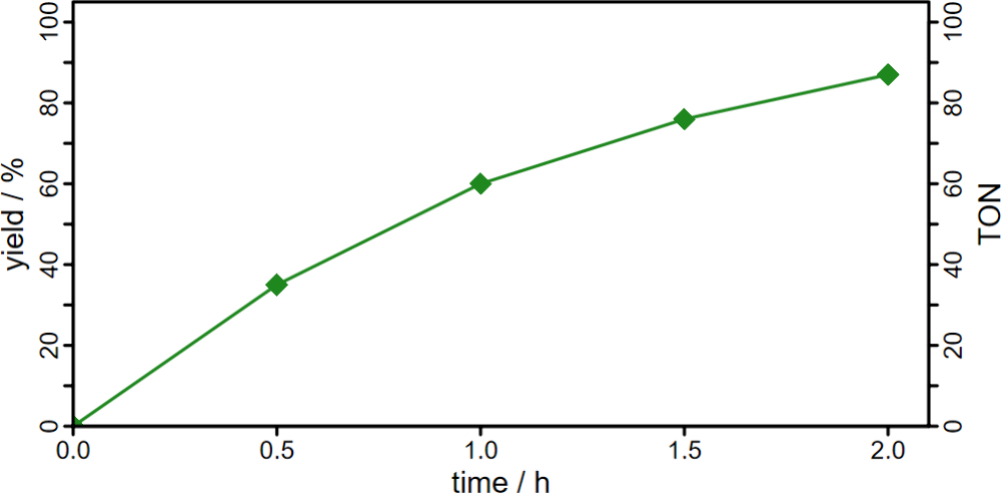
Reaction yield and turnover number (TON) of QC over time using **C4** as a photosensitizer.

Encouraged by this result, the photocatalytic efficiency of all Cu­(I) complexes (**C2**–**C5**) were evaluated under standardized conditions (Table 4). A clear trend emerges: **C4** and **C5** demonstrate the highest catalytic efficiencies, achieving yields of 60% and 65% after 1 h, respectively. The slightly enhanced performance of **C5** can be attributed to its more red-shifted absorption, which improves light harvesting while maintaining a sufficiently high E_T1_. In contrast, **C3** showed the lowest activity (26% yield), likely due to its slightly reduced absorption at the irradiation wavelength (>320 nm) and its excited-state energy being close to the threshold required for NBD activation. **C2** performed slightly better (38% yield), potentially benefiting from better absorption characteristics compared to **C3**. The control experiment without a sensitizer yielded only a 7% conversion, confirming that the reaction proceeds through photosensitization rather than direct excitation. Additionally, Ir­(dFppy)_3_ was investigated as a photosensitizer under equivalent conditions as a benchmark system (see Table 4, for further details see SI Chapter 14). Albeit showing a superior conversion, which may be attributed to its red-shifted absorption profile^73^ and slightly higher excited-state energy (E_T1_ = 2.75 eV, cf. Figure 7),^62^ the overall yield is still in a comparable range to the best performing Cu­(I)-based sensitizers **C5** and **C4**.

**Table 4:**
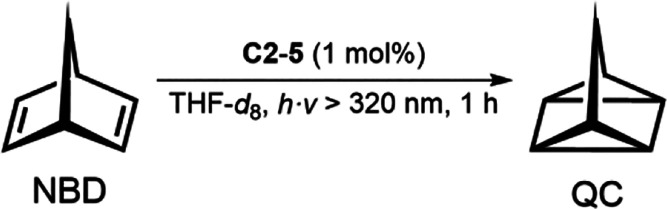
Results of the Photocatalytic Isomerization of Norbornadiene (NBD) to Quadricyclane (QC)^a^

Entry	Complex	Yield/% (Conversion/%)	TON (1 h)
1	**C2**	38 (38)	38
2	**C3**	26 (26)	26
3	**C4**	60 (63)	60
4	**C5**	65 (66)	65
5		7 (7)	7
6	**C4** ^b^	0 (0)	0
7	Ir(dFppy)_3_	76 (99)	76

a Values are reported after 1 h of irradiation with a 150 W Xe arc lamp (see SI Chapter 14 for further details). Yields were directly determined by ^1^H NMR spectroscopy. TON stands for turnover number.

b Control experiment without irradiation.

Overall, these results demonstrate that all investigated Cu­(I) complexes effectively perform the challenging energy transfer reaction, emphasizing their high excited-state energies and sufficient lifetimes. Additionally, the design and electronic structure of the N,N′-ligand significantly influences the MLCT excited states, directly impacting the catalytic activity.

## Conclusion

To conclude, we have successfully synthesized and thoroughly characterized four novel three-coordinate Cu­(I) complexes featuring an anionic N-heterocyclic carbene (NHC) ligand. Our refined synthetic approach replaces the carbene ligand of conventional cationic Cu­(I) complexes with an anionic NHC, paired with a weakly coordinating tris­(pentafluorophenyl)­borate anion (WCA-NHC).

Electrochemical studies revealed an irreversible oxidation process attributed to the Cu­(I)/Cu­(II) redox couple and a reduction event localized on the N,N′-ligand. The introduction of methyl substituents at the 4,4′-positions of the N,N′-ligand resulted in the strongest electronic influence, as evidenced by both electrochemical and computational data.

Photophysical investigations showed that the lowest-energy absorption band primarily originates from metal-to-ligand charge transfer (MLCT) transitions. The Cu­(I) complexes exhibit small singlet–triplet energy separations (0.06−0.09 eV), characteristic of thermally activated delayed fluorescence (TADF). Temperature-dependent emission studies suggest significant excited-state structural relaxation, supported by TDDFT calculations, which revealed a pseudo-Jahn–Teller distortion that accounts for the observed hypsochromic shift at cryogenic temperatures.

Notably, all complexes show high excited-state energies (≈2.7 eV), surpassing those of conventional four-coordinate Cu­(I) complexes and matching the performance of Ir­(III)-based photosensitizers. Additionally, their high quantum yields (up to 11.5%) and sufficiently long excited-state lifetimes (up to 8.6 μs) provide an excellent foundation for participation in photoinduced bimolecular reactions.

All four Cu­(I) complexes demonstrated activity as photosensitizers in the norbornadiene-to-quadricyclane photoisomerization, a reaction relevant for molecular solar thermal energy storage (MOST) applications. Due to the fine-tuning of the MLCT excited states in **C4** and **C5**, these complexes exhibited the highest catalytic efficiencies. Notably, this study represents the first comprehensive investigation of the structure–activity relationships of three-coordinate Cu­(I)-NHC complexes in photocatalysis, marking a significant advancement in the field. This expands the applicability of Cu­(I) photosensitizers beyond conventional four-coordinate systems and demonstrates that strategic ligand design can overcome previous limitations.

Our findings highlight the potential of anionic NHC ligands in broadening the scope of photoactive Cu­(I) complexes. The synthetic strategy employed here enables precise tuning of MLCT properties through modifications of the N,N′-ligand, providing a versatile platform for further optimization. A major challenge that remains is the limited absorption in the visible spectral range. Therefore, future work will focus on shifting the absorption profile into the visible region and minimizing structural distortions to enhance the light harvesting efficiency while maintaining advantageous photophysical properties.

## Materials and Methods

Detailed explanation on the materials and methods applied, including experimental and computational details as well as additional information on the syntheses, NMR spectra, (TD)­DFT calculations, electrochemistry, absorption and (temperature dependent) emission spectroscopy, as well as photocatalytic experiments are given in the Supporting Information (SI).

## Supplementary Material



## Notes

Experimental and computational details, description of the synthetic procedures including NMR spectra and crystallographic data; further information on electrochemical, computational, and spectroscopic data as well as a detailed description of the photocatalytic experiments (PDF)

## Notes

The crystallographic data are available free of charge from the joint Cambridge Crystallographic Data Center and the Fachinformationszentrum Karlsruhe via www.ccdc.cam.ac.uk/structures/ (CCDC 2432855−2432861).
