# Supplementary material for: Photoactive Neutral Three-Coordinate Cu(I) Complexes of Anionic N‑Heterocyclic Carbenes
Source: JACS Au. 2025 Jun 9;5(6):2792–801. doi: 10.1021/jacsau.5c00357 (PMC12188385; doi:10.1021/jacsau.5c00357)
Supplement: Supplementary file 1 [file au5c00357_si_001.pdf]

Supporting Information

Photoactive Neutral Three-Coordinate Cu(I) Complexes of N-Heterocyclic Carbenes

Lars E. Burmeister,[1,†] Lucie J. Groth,[2,†] Philipp R. Meinhold,[2] Johannes P. Zurwellen,[1] Dirk Bockfeld,[2] René Frank,[2] Michael Karnahl,[1] Matthias Tamm,[2,] Stefanie Tschierlei[1,*]*

[1] Department of Energy Conversion, Institute of Physical and Theoretical Chemistry, Technische Universität Braunschweig, Rebenring 31, 38106 Braunschweig, Germany.
[2] Institute of Inorganic and Analytical Chemistry, Technische Universität Braunschweig, Hagenring 30, 38106 Braunschweig, Germany.

[†] These authors have contributed equally to this work and share first authorship.
*Correspondence:
Prof. Dr. Matthias Tamm: m.tamm@tu-bs.de
Prof. Dr. Stefanie Tschierlei: s.tschierlei@tu-bs.de

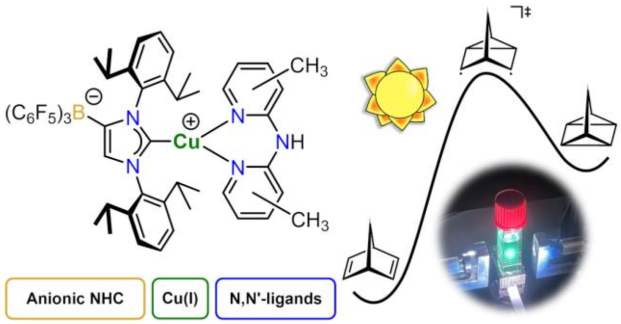

Supporting Information - Table of Contents

1 Experimental Details 2
2 Synthetic Procedures 6
3 NMR Spectra 14
4 Crystallographic Data 49
5 Electrochemical Data 57
6 Density Functional Theory (DFT) Calculations 58
7 Time-Dependent Density Functional Theory (TDDFT) 66
8 UV/vis Absorption 82
9 Time-resolved Emission 83
10 Temperature Dependent Spectroscopy 84
11 Determination of E^{00} 89
12 Photostability 90
13 Stern-Volmer Quenching Experiments 91
14 Photocatalysis 92
15 References 113

1 Experimental Details

NMR spectroscopy. ^{1}H, $^{13}C\{^{1}H\}$, $^{11}B\{^{1}H\}$ and $^{19}F\{^{1}H\}$ NMR spectra were recorded on Bruker AVII300 (300 MHz), AVIIHD300 (300 MHz), AVIII400 (400 MHz), AVII500 (500 MHz) or AVII600 (600 MHz). ^{1}H NMR spectra for monitoring the catalytic conversion of norbornadiene (NBD) to quadricyclane (QC) were performed with a relaxation delay (D1 = 60 sec) and reduced number of scans (NS = 8). All spectra were recorded at room temperature. The spectra were processed using TopSpin 4.3.0. The chemical shifts are given in ppm relative to residual solvent peaks (^{1}H: δ = 1.72 (THF-*d8*), 3.58 (THF-*d8*), 7.26 ($CDCl_3$) ppm; ^{13}C: 25.31 (THF-*d8*), 67.21 (THF-*d8*), 77.16 ($CDCl_3$) ppm). Coupling constants (*J*) are reported in Hertz (Hz), and splitting patterns are indicated as s (singlet), d (doublet), t (triplet), q (quartet), sept (septet), m (multiplet) and br (broad). The signals corresponding to the aromatic residues are assigned by *i* (*ipso*), *o* (*ortho*), *m* (*meta*) and *p* (*para*) if not indicated otherwise.

Elemental analysis. Elemental analyses were performed on a Vario Micro Cube System (VarioMICRO V4.0.15). Solvents used in the calculation of the theoretical values were was well found in the corresponding NMR spectra and crystal structures.

X-ray diffraction studies. Crystals were mounted on a Hamton CryoLoop™ with per-fluorinated inert oil. Data were recorded on Rigaku XtaLAB Synergy S Single Source diffractometers equipped with a PhotonJet Cu- or Mo-microfocus source and a HyPix-6000HE detector. Data reduction was performed with CrysalisPro.[1] Absorption corrections were based on multi-scans for compound **C2-II** and additionally, face indexation and integration on a Gaussian grid was applied for all other compounds. The structures were solved by intrinsic phasing with SHELXT-2018/2[2] and refined on F^2 using the program SHELXL-2018/3[3] in OLEX^2.[4] If not otherwise noted in the corresponding tables below, H atoms were placed in idealized positions and refined using a riding model.

Synthetic details. All reactions were performed under a strictly dry argon (Ar) atmosphere using standard Schlenk-line techniques or in a glovebox under dry Ar atmosphere (MBraun 200B). The used glassware was oven-dried at 130 °C and evacuated multiple times before use. The solvents were dried with an MBraun solvent purification system, degassed and stored over 3-5 Å molecular sieves. THF-d_8 was dried over Na/K and stored over 3 Å molecular sieves or used directly for the photocatalytic conversion, $CDCl_3$ was dried over CaH_2 and stored over 3-5 Å molecular sieves. The starting materials were purchased from commercial sources (TCI, Merck, Roth, Alfa-Aesar) and if necessary purified by conventional methods. Norbornadiene (NBD) was distilled in the dark prior to use. $(C_6F_5)_3B$-IDipp-Li(Toluene)[5] and ligands **L2**-**L6**[6] were prepared according to literature known procedures. Solvate $(C_6F_5)_3B$-IDipp-Cu(Toluene)[7] (**C1**) was generated following the literature known procedure and used without isolation. The workup of ligands **L2**-**L6** was modified as described below. Reaction temperatures refer to the temperature of the silicon oil heating bath.

(TD)DFT calculations. Quantum chemical calculations were performed on the density functional theory level using the ORCA program package (version 5.0.4).[8,9] All calculations were performed using the PBE0 hybrid-functional, in combination with the Karlsruhe valence polarization basis set of triple-ζ quality (PBE0/def2-TZVP).[10,11] The RI-J approximation for coulomb integrals and

COSX numerical chain-of-sphere integration for Hartree-Fock exchange in combination with the def2/J auxiliary basis set was applied.[12–16] Dispersion interactions were taken into account by applying Grimme's D3 dispersion correction including the Becke-Johnson damping scheme.[17,18] Tight convergence criteria were chosen for all calculations. Solvent effects were simulated by applying the conductor-like polarizable continuum model for tetrahydrofuran.[19] The optimized ground state geometries were verified as minima on the potential energy surface by frequency calculations (analytical). Orbital composition analysis of the atomic orbital contributions to the molecular orbitals were calculated by Hirshfeld analysis using the multifunctional wave analyzer (Mulitwfn).[20–22] Atomic densities were obtained by the build-in sphericalized atomic densities. For the calculation of electronic transitions and the prediction of absorption spectra TDDFT was applied. Calculations were performed using the Tamm-Dancoff approximation.[23] Excited-state geometries of the S_1 and T_1 states were optimized by applying TDDFT. The DFT and TDDFT data were visualized and analyzed using the UCSF ChimeraX program.[24–26] Plane and twist angles of the optimized geometries were calculated using the Olex2-1.5 program.[4]

General details for spectroscopic measurements. Samples for spectroscopic analyses were prepared under inert conditions using Schlenk technique or in a glove box (MBraun 200B) under dry Ar atmosphere. THF was dried over Na (with benzophenone) and distilled under Ar atmosphere. 2-Methyltetrahydrofuran was dried over Na/K and vacuum distilled.

Electrochemistry. Cyclic voltammetry was carried out using a Metrohm Autolab potentiostat PGSTAT204. Measurements were acquired in inert tetrahydrofuran (Ar atmosphere) with an analyte concentration of 1 mM and $[Bu_4N][PF_6]$ (0.1 M) as the supporting electrolyte. A three-electrode configuration consisting of a glassy carbon disc (⌀ = 3 mm) as a working electrode, a platinum wire as a counter electrode and a non-aqueous Ag/Ag^+ reference electrode was used. The scan rate was set to 100 mV/s. All data are referenced against the ferrocene/ferrocenium (Fc/Fc^+) couple, by adding ferrocene to the solution after each measurement. All reduction and oxidation potentials were obtained from the respective minima/maxima.

UV/vis absorption spectroscopy. Absorption spectra were acquired using a Jasco V-770 UV-visible/NIR spectrophotometer. All spectra were recorded continuously (400 nm/min) in the range from 850 - 250 nm with a data interval of 1 point/nm. Samples of the ligands were prepared in a fluorescence quartz glass cuvette (10 x 10 mm, Hellma® Analytics) using tetrahydrofuran under ambient conditions. Samples of the complexes were prepared in a quartz glass cuvette (1 x 10 mm, Hellma® Analytics) using inert tetrahydrofuran (Ar atmosphere). All spectra are baseline corrected.

Steady-state emission spectroscopy. Emission spectra were recorded using a Horiba Jobin-Yvon FluoroMax Plus-C emission spectrometer. All samples were prepared under inert conditions (Ar atmosphere) using a sealed fluorescence quartz glass cuvette (10 x 10 mm, Hellma® Analytics) The optical densities of the respective solutions were adjusted to 0.1 ± 0.01 at the excitation wavelength (λ_{exc} = 355 nm). A 380 nm longpass filter was introduced in front of the detector. For the determination of the quantum yields [Cu(bathocuproine)(xantphos)]PF_6 in tetrahydrofuran (Φ_R = 0.08)[27] was used as reference. Quantum yields of the compounds (Φ_c) were calculated according to eq. S1 using the absorbance of the compound (A_C) and the reference (A_R) at the

excitation wavelength, along with the integral of the compound's emission (I_C) and the reference's emission (I_R).[28] The emission curves were integrated from 410 to 780 nm.

$$\Phi_c = \Phi_R \left(\frac{A_R}{A_C}\right)\left(\frac{I_C}{I_R}\right) \quad \text{(eq. S1)}$$

Time-resolved emission spectroscopy. Emission lifetimes were measured utilizing a Q-switched pulsed Nd:YAG laser system with excitation pulses of approximately 6 ns centered at 355 nm. The laser power was adjusted to around 1.0 mJ per pulse at the sample. Emission was detected around the respective emission maxima by a photomultiplier tube integrated with an Edinburgh Instruments LP980 spectrometer. Emission lifetimes were recorded under oxygen free conditions in inert tetrahydrofuran (Ar atmosphere) in sealed fluorescence quartz glass cuvettes (10 x 10 mm, Hellma® Analytics) at room temperature. The optical densities of the respective solutions were adjusted to 0.1 ± 0.01 at the excitation wavelength (λ_{exc} = 355 nm).

Estimation of k_r and k_{nr}. Radiative (k_r) and non-radiative decay rate constants (k_{nr}) were estimated by applying the following formalism (eq. S2 and S3).[29–31]

$$k_r = \frac{\phi_{em}}{\tau_{em}} \quad \text{(eq. S2)}$$

$$k_{nr} = \frac{1}{\tau_{em}} - k_r \quad \text{(eq. S3)}$$

Temperature dependent measurements. Temperature dependent measurements were performed with an Oxford Instruments Optistat DN-X cryostat in combination with an Oxford Instruments MercuryiTC temperature controller using nitrogen as the cryogen and helium as the exchange gas. The samples were prepared under oxygen free conditions (Ar atmosphere) in dry 2-methyltetrahydrofuran.

Photostability measurements. UV/vis absorption spectra for the evaluation of the photostability of **C4** were obtained with an Avantes AvaSpec-ULS2048CL spectrophotometer. A 150 W Xe arc lamp (LOT-QuantumDesign GmbH, LSE140/160.25C) was operated at 121 W and used as light source. A 280 nm cut-off filter was placed between the light source and the sample to block any undesired wavelengths. The measurements were carried out under inert conditions (Ar atmosphere) in a sealed quartz glass cuvette (10 x 10 mm, Hellma® Analytics) equipped with a magnetic stir bar (400 rpm).

Stern-Volmer Analysis. Stern-Volmer quenching experiments were carried out by sequential addition of 0.1 mL NBD to a solution of **C4** (2 mL, OD = 0.07) in inert THF (Ar atmosphere). The emission lifetimes were obtained as described above. The quenching constant (k_q) was determined according to the Stern-Volmer relation (eq. S4).[31]

$$\frac{\tau_0}{\tau} - 1 = K_{SV}[Q] = k_q \tau_0 [Q] \quad \text{(eq. S4)}$$

In this equation, τ_0 and τ are the emission lifetimes of the complex in absence and presence of the quencher, respectively. The Stern-Volmer constant (K_{SV}) represents the slope of a linear fit of $\frac{\tau_0}{\tau} - 1$ *vs.* the quencher concentration ($[Q]$).

Photocatalysis. For preparing the stock solutions all volumetric measurements were performed using 100 µL, 250 µL or 500 µL MICROLITER® syringes by Hamilton or volumetric flasks of 2 mL and 5 mL. Weights of the catalysts were determined using a Mettler Toledo MX5 scale. The photocatalytic conversion of norbornadiene (NBD) to quadricyclane (QC) was carried out by irradiating respective inert THF-d_8 (Ar atmosphere) solutions containing NBD and Cu photosensitizer in an NMR tube (Wilmad, Type 1, Class A glass; UV transmission > 320 nm) with a 150 W Xe arc lamp (LOT-QuantumDesign GmbH, LSE140/160.25C). The Xe arc lamp was operated at 130 W and the distance between the sample and the light source was 30 cm. The intensity was determined with thermal power sensor (THORLABS, S415C) to 0.9 W in front of the sample. No active temperature control was applied during the photocatalytic experiments. All reactions were carried out under ambient laboratory conditions with passive heat dissipation. Photocatalytic experiments were reproduced two times.

2 Synthetic Procedures

Di(pyridin-2-yl)amine (L2, Hdpa)

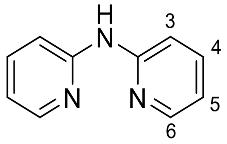

The synthesis was adapted from GAILLARD.[6]

Tris(dibenzylidenaceton)dipalladium(0) ($Pd_2(dba)_3$) (0.1 g, 0.12 mmol, 2 mol%), 1,3-bis(diphenylphosphino)propan (dppp) (0.1 g, 0.24 mmol, 4 mol%) and KO^{t}Bu (1.0 g, 8.8 mmol, 1.4 eq.) were dissolved in toluene (25 mL). After the addition of 2-bromopyridine (0.65 mL, 5.8 mmol, 1.0 eq.) and 2-aminopyridine (0.7 g, 7.6 mmol, 1.2 eq.) the reaction mixture was heated to reflux for 16 h. The volatiles were removed under reduced pressure. The brown crude product was purified *via* a filter collumn (EtOAc) and additional sublimation (50 °C, 0.04 mbar) to obtain a white solid (0.68 g, 4.0 mmol, 62 %).

^{1}H NMR (400 MHz, THF-d_8): δ = 8.75 (s br., 1H, N*H*), 8.15 (ddd, $^3J_{HH}$ = 4.9 Hz, $^4J_{HH}$ = 2.0 Hz, $^5J_{HH}$ = 0.9 Hz, 2H, H_6), 7.71 (ddd, $^3J_{HH}$ = 8.4 Hz, $^4J_{HH}$ =0.9 Hz, $^5J_{HH}$ = 0.9 Hz, 2H, H_3), 7.53 (ddd, $^3J_{HH}$ = 8.5 Hz, $^3J_{HH}$ = 7.1 Hz, $^4J_{HH}$ = 2.0 Hz, 2H, H_4), 6.74 (ddd, $^3J_{HH}$ = 7.3 Hz, $^3J_{HH}$ = 4.9 Hz, $^4J_{HH}$ = 1.0 Hz, 2H, H_5) ppm.

^{13}C{^{1}H} NMR (100 MHz, THF-d_8): δ = 155.8 (s, NH-*C*), 148.3 (s, C_6), 137.7 (s, C_4), 116.3 (s, C_5), 112.5 (s, C_3) ppm.

Bis(3-methylpyridin-2-yl)amine (L3, 3,3'Me_2Hdpa)

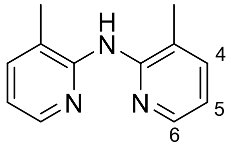

The synthesis was adapted from GAILLARD.[6]

$Pd_2(dba)_3$ (0.11 g, 0.1 mmol, 2 mol%) and dppp (0.23 g, 0.1 mmol, 10 mol%) were dissolved in toluene (25 mL). After the addition of 2-bromo-3-methylpyridine (0.65 mL, 5.9 mmol, 1.0 eq.), 2-amino-3-methylpyridine (0.7 mL, 7.0 mmol, 1.2 eq.) and KO^{t}Bu (0.91 g, 8.1 mmol, 1.4 eq.) the reaction mixture was heated to reflux for 17 h. The volatiles were removed under reduced pressure. The brown crude product was purified *via* a filter column (EtOAc) and additional sublimation (80 °C, 0.04 mbar) to obtain a white solid (0.45 g, 2.81 mmol, 39 %).

^{1}H NMR (300 MHz, $CDCl_3$): δ = 8.14 (dm, $^3J_{HH}$ = 5.0 Hz, 2H, H_6), 7.45 (dm, $^3J_{HH}$ = 7.4 Hz, 2H, H_4), 6.88 (dd, $^3J_{HH}$ = 7.4 Hz, $^3J_{HH}$ = 5.0 Hz, 2H, H_5), 6.38 (s br., 1H, N*H*), 2.23 (s, 6H, C*H*$_3$) ppm.

^{13}C{^{1}H} NMR (76 MHz, $CDCl_3$): δ = 153.6 (s, NH-*C*), 145.7 (s, C_6), 138.7 (s, C_4), 123.5 (s, *C*-CH_3), 118.1 (s, C_5), 18.2 (s, *C*H_3) ppm.

Bis(4-methylpyridin-2-yl)amine (L4, 4,4'Me$_2$Hdpa)

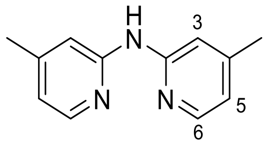

The synthesis was adapted from GAILLARD.[6]

$Pd_2(dba)_3$ (0.11 g, 0.1 mmol, 2 mol%), dppp (0.23 g, 0.56 mmol, 10 mol%) and KO^{t}Bu (0.91 g, 8.1 mmol, 1.4 eq.) were dissolved in toluene (25 mL). After the addition of 2-bromo-4-methylpyridine (0.65 mL, 5.9 mmol, 1.0 eq.) and 2-amino-4-methylpyridine (0.76 g, 7.0 mmol, 1.2 eq.) the reaction mixture was heated to reflux for 17 h. The volatiles were removed under reduced pressure. The brown crude product was purified *via* a filter column (EtOAc) and additional sublimation (50 °C, 0.02 mbar) to obtain a white solid (0.51 g, 2.56 mmol, 44 %).

^{1}H NMR (300 MHz, $CDCl_3$): δ = 8.12 (dm, $^3J_{HH}$ = 5.2 Hz, 2H, H_6), 7.57 (s br., 1H, N$\underline{H}$), 7.37-7.33 (m, 2H, H_3), 6.67 (dm, $^3J_{HH}$ = 5.2 Hz, 2H, H_5), 2.32 (s, 6H, C$\underline{H}_3$) ppm.

^{13}C{^{1}H} NMR (76 MHz, THF-d_8): δ = 154.4 (s, $\underline{C}$-CH_3), 149.0 (s, NH-$\underline{C}$), 147.5 (s, C_6), 117.9 (s, C_5), 112.0 (s, C_3), 21.4 (s, $\underline{C}H_3$) ppm.

Bis(5-methylpyridin-2-yl)amine (L5, 5,5'Me$_2$Hdpa)

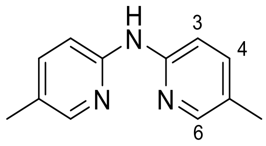

The synthesis was adapted from GAILLARD.[6]

$Pd_2(dba)_3$ (0.1 g, 0.11 mmol, 2 mol%), dppp (0.23 g, 0.56 mmol, 10 mol%) and KO^{t}Bu (0.9 g, 8.1 mmol, 1.4 eq.) were dissolved in toluene (25 mL). After the addition of 2-bromo-5-methylpyridine (1.0 g, 5.85 mmol, 1.0 eq.) and 2-amino-5-methylpyridine (0.76 g, 7.0 mmol, 1.2 eq.) the reaction mixture was heated to reflux for 17 h. The volatiles were removed under reduced pressure. The brown crude product was purified *via* a filter column (EtOAc) and additional sublimation (60 °C, 0.01 mbar) to obtain a white solid (0.58 g, 2.91 mmol, 50 %).

^{1}H NMR (300 MHz, THF-d_8): δ = 8.10-8.05 (m, 2H, H_6), 7.69 (s br., 1H, N$\underline{H}$), 7.42-7.38 (m, 4H, $H_{3,4}$), 2.46 (d, $^4J_{HH}$ = 0.8 Hz, 6H, C$\underline{H}_3$) ppm.

^{13}C{^{1}H} NMR (76 MHz, THF-d_8): δ = 152.4 (s, NH-$\underline{C}$), 147.6 (s, C_6), 138.6 (s, C_5), 125.1 (s, $\underline{C}$-CH_3), 111.1 (s, C_3), 17.8 (s, $\underline{C}H_3$) ppm.

Bis(6-methylpyridin-2-yl)amine (L6, 6,6'Me_2Hdpa)

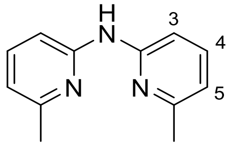

The synthesis was adapted from GAILLARD.[6]

$Pd_2(dba)_3$ (0.1 g, 0.12 mmol, 2 mol%), dppp (0.1 g, 0.24 mmol, 4 mol%) and KO^{t}Bu (0.9 g, 8.1 mmol, 1.4 eq.) were dissolved in toluene (25 mL). After the addition of 2-bromo-6-methylpyridine (0.6 mL, 6.4 mmol, 1.0 eq.) and 2-amino-6-methylpyridine (0.7 g, 7.0 mmol, 1.2 eq.) the reaction mixture was heated to reflux for 17 h. The volatiles were removed under reduced pressure. The brown crude product was purified *via* a filter column (EtOAc) and additional sublimation (80 °C, 0.04 mbar) to obtain a white solid (0.45 g, 2.81 mmol, 39 %).

^{1}H NMR (300 MHz, $CDCl_3$): δ = 7.48 (ddd, $^3J_{HH}$ = 8.3 Hz, $^3J_{HH}$ = 7.3 Hz, 2H, H_4), 7.35 (dm, $^3J_{HH}$ = 8.3 Hz, 2H, H_3), 7.19 (s br., 1H, N$\underline{H}$), 6.69 (dm, $^3J_{HH}$ = 7.3 Hz, 2H, H_5), 2.46 (s, 6H, C$\underline{H}_3$) ppm.

^{13}C{^{1}H} NMR (76 MHz, $CDCl_3$): δ = 156.9 (s, $\underline{C}$-CH_3), 153.6 (s, NH-$\underline{C}$), 138.1 (s, C_4), 115.7 (s, C_5), 108.4 (s, C_3), 24.4 (s, $\underline{C}H_3$) ppm.

$B(C_6F_5)_3$-IDipp-Cu-Hdpa (C2)

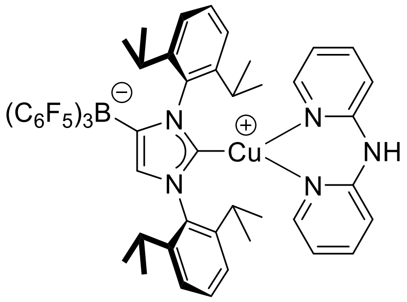

$B(C_6F_5)_3$-IDipp-Li(tol) (100.3 mg, 0.1 mmol, 1.0 eq) and CuCl (10.2 mg, 0.1 mmol, 1.0 eq.) were suspended in toluene (5 mL) and stirred for 18 h at rt. The resulting suspension was filtered *via* celite, and added to a solution of **L2** (17.1 mg, 0.1 mmol, 1.0 eq.) in toluene (2 mL). After 2 h were the volatiles removed *in vacuo* and the resulting solid was washed with a minimal amount of toluene. The solid was dried *in vacuo* to yield complex **C2** (105.4 mg, 0.86 mmol, 86 %). Single crystals suitable for scXRD were obtained by layering a solution in toluene with *n*-pentane at rt (**C2-I**, **C2-II**) or by layering a solution in THF with *n*-pentane at rt (**C2-III**).

Elemental analysis (%): calcd for $C_{55}H_{44}BCuF_{15}N_5 \cdot C_7H_8$: C 60.72, H 4.27, N 5.71; found C 60.69, H 4.21, N 5.68.

^{1}H NMR (500 MHz, THF-d_8): δ = 9.01 (s br, 1H, NH), 7.52 (ddd, $^3J_{HH}$=8.4 Hz, $^3J_{HH}$=7.3 Hz, $^4J_{HH}$=1.8 Hz, 2H, $H_{4,Hdpa}$), 7.42 (t, $^3J_{HH}$=7.8 Hz, 1H, *p*-Dipp$_A$), 7.41 (t, $^3J_{HH}$=7.7 Hz, 1H, *p*-Dipp$_B$), 7.22 (d, $^3J_{HH}$=7.7 Hz, *m*-Dipp$_A$), 7.20-7.05 (m, toluene), 7.09 (d, $^3J_{HH}$=7.8 Hz, 2H, *m*-Dipp$_B$), 6.73 (d br, $^3J_{HH}$=8.4 Hz, 2H $H_{3,Hdpa}$), 6.45 (s br, 1H, C$\underline{H}$=C), 6.42 (dd, $^3J_{HH}$=7.3 Hz, $^3J_{HH}$=5.3 Hz, 2H, $H_{5,Hdpa}$), 6.19 (dm, $^3J_{HH}$=5.3 Hz, 2H, $H_{6,Hdpa}$), 3.15 (sept, $^3J_{HH}$=6.8 Hz, 2H, $C\underline{H}_B(CH_3)_2$), 2.90 (sept, $^3J_{HH}$=6.8 Hz, 2H, $C\underline{H}_A(CH_3)_2$), 2.31(s, toluene), 1.10 (d, $^3J_{HH}$=6.8 Hz, 6H, $CH(C\underline{H}_{3,A})_2$), 1.07 (d, $^3J_{HH}$=6.8 Hz, 6H, $CH(C\underline{H}_{3,B})_2$), 1.14 (d, $^3J_{HH}$=6.8 Hz, 6H, $CH(C\underline{H}_{3,A})_2$), 0.82 (d, $^3J_{HH}$=6.9 Hz, 6H, $CH(C\underline{H}_{3,B})_2$) ppm.

^{13}C{^{1}H} NMR (126 MHz, THF-d_8): δ = 182.0 (s, $C_{carbene}$), 154.2 (s, $C_{2,Hdpa}$), 149.9 (dm, $^1J_{CF}$=239 Hz, *o*-C_6F_5), 149.6 (s, $C_{6,Hdpa}$), 149.2 (q, $^1J_{BC}$ = 60 Hz, C=$\underline{C}$B), 148.4 (s, *o*-Dipp$_B$), 147.3 (s, *o*-Dipp$_A$), 140.1 (s, *i*-Dipp$_B$), 139.6 (s, $C_{4,Hdpa}$), 139.5 (dm, $^1J_{CF}$=243 Hz, *p*-C_6F_5), 138.4 (s, toluene), 138.3 (s, *i*-Dipp$_A$), 137.4 (dm, $^1J_{CF}$=243 Hz, *m*-C_6F_5), 131.4 (s br, $\underline{C}$H=C), 130.6 (s, *p*-Dipp$_A$), 130.1 (s, *p*-Dipp$_B$), 129.7 (s, toluene), 128.9 (s, toluene), 126.0 (s, toluene), 125.0 (s, *m*-Dipp$_A$), 124.3 (s, *m*-Dipp$_B$), 117.4 (s, $C_{5,Hdpa}$), 114.0 (s, $C_{3,Hdpa}$), 28.8 (s, $\underline{C}_AH(CH_3)_2$), 28.5 (s, $\underline{C}_BH(CH_3)_2$), 27.0 (s, $CH(\underline{C}_BH_3)_2$), 24.4 (s, $CH(\underline{C}_AH_3)_2$), 24.2 (s, $CH(\underline{C}_AH_3)_2$), 22.1 (s, $CH(\underline{C}_BH_3)_2$), 21.5 (s, toluene) ppm.

Signal for *i*-C_6F_5 was not observed.

^{11}B{^{1}H} NMR (160 MHz, THF-d_8): δ = −15.3 (s) ppm.

^{19}F{^{1}H} NMR (471 MHz, THF-d_8): δ = −128.2 (s br, 6F, *m*-C_6F_5), −163.0 (t, $^3J_{FF}$=20 Hz, 3F, *p*-C_6F_5), −167.1-(−167.6) (m, 6F, *o*-C_6F_5) ppm.

$B(C_6F_5)_3$-IDipp-Cu-3,3'Me_2Hdpa (C3)

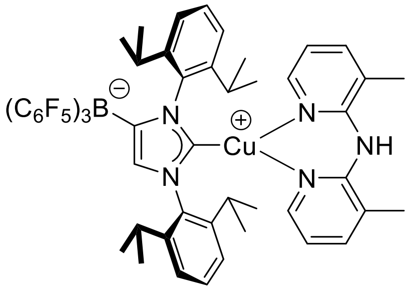

$B(C_6F_5)_3$-IDipp-Li(tol) (100 mg, 0.1 mmol, 1 eq.) was dissolved in toluene (5 mL), CuCl (17 mg, 0.1 mmol, 1 eq.) was added and the suspension stirred for 25 h at rt. The resulting suspension was filtered over celite. The solution was added to **L3** (20 mg, 0.1 mmol, 1 eq.) and stirred for 2 h at rt. To the resulting suspension *n*-pentane was added to ascertain complete precipitation. The supernatant solution was removed and the solid dried under vacuum to obtain **C3** as a white powder (86 mg, 0.074 mmol, 74 %). Single crystals suitable for scXRD were obtained by layering a solution in toluene with *n*-hexane at −40 °C.

Elemental analysis (%): calcd for $C_{55}H_{44}BCuF_{15}N_5 \cdot 0.5(C_7H_8)$: C 60.08, H 4.42, N 5.79; found C 59.99, H 4.50, N 6.02.

^{1}H NMR (600 MHz, THF-d_8): δ = 7.47 (dm, $^{3}J_{HH}$=7.4 Hz, 2H, $H_{4,Hdpa}$), 7.41 (t, $^{3}J_{HH}$=7.8 Hz, 1H, *p*-$Dipp_A$), 7.40 (t, $^{3}J_{HH}$=7.8 Hz, 1H, *p*-$Dipp_B$), 7.20 (d, $^{3}J_{HH}$=7.8 Hz, 2H, *m*-$Dipp_A$), 7.20-7.05 (m, toluene), 7.07 (d, $^{3}J_{HH}$=7.8 Hz, 2H, *m*-$Dipp_B$), 7.05 (s br, 1H, NH), 6.46 (s br, 1H, C$\underline{H}$=C), 6.43 (dd, 2H, $^{3}J_{HH}$=5.4 Hz, $^{3}J_{HH}$=7.4 Hz, $H_{5,Hdpa}$), 6.20 (dm, $^{3}J_{HH}$=5.4 Hz, 2H, $H_{6,Hdpa}$), 3.13 (sept, $^{3}J_{HH}$=6.8 Hz, 2H, $C\underline{H}_B(CH_3)_2$), 2.90 (sept, $^{3}J_{HH}$=6.8 Hz, 2H, $C\underline{H}_A(CH_3)_2$), 2.30 (s, 6H, pyr-CH_3), 2.30 (s, toluene), 1.09 (d, $^{3}J_{HH}$=6.8 Hz, 6H, $CH(C\underline{H}_{3,A})_2$), 1.08 (d, $^{3}J_{HH}$=6.8 Hz, 6H, $CH(C\underline{H}_{3,B})_2$), 1.06 (d, $^{3}J_{HH}$=6.8 Hz, 6H, $CH(C\underline{H}_{3,A})_2$), 0.95 (d, $^{3}J_{HH}$=6.8 Hz, 6H, $CH(C\underline{H}_{3,B})_2$) ppm.

$^{13}C\{^{1}H\}$ NMR (150 MHz, THF-d_8): δ = 182.0 (s, $C_{carbene}$), 152.3 (s, $C_{2,Hdpa}$), 149.7 (dm, $^{1}J_{CF}$=239 Hz, *o*-C_6F_5), 149.1 (q, $^{1}J_{BC}$ = 60 Hz, C=$\underline{C}$B), 148.4 (s, *o*-$Dipp_B$), 147.3 (s, $C_{6,Hdpa}$), 147.2 (s, *o*-$Dipp_A$), 140.4 (s, $C_{4,Hdpa}$), 140.1 (s, *i*-$Dipp_B$), 139.4 (dm, $^{1}J_{CF}$=243 Hz, *p*-C_6F_5), 138.4 (s, toluene), 138.2 (s, *i*-$Dipp_A$), 137.4 (dm, $^{1}J_{CF}$=243 Hz, *m*-C_6F_5), 131.4 (s br, $\underline{C}$H=C), 130.4 (s, *p*-$Dipp_A$), 130.1 (s, *p*-$Dipp_B$), 129.6 (s, toluene), 128.9 (s, toluene), 126.0 (s, toluene), 124.9 (s, *m*-$Dipp_A$), 124.3 (s, *m*-$Dipp_B$), 118.5 (s, $C_{5,Hdpa}$), 28.8 (s, $\underline{C}_AH(CH_3)_2$), 28.5 (s, $\underline{C}_BH(CH_3)_2$), 27.1 (s, $CH(\underline{C}_BH_3)_2$), 24.5 (s, $CH(\underline{C}_AH_3)_2$), 24.3 (s, $CH(\underline{C}_AH_3)_2$), 22.1 (s, $CH(\underline{C}_BH_3)_2$), 21.5 (s, toluene), 17.0 (s, Ar-$\underline{C}H_3$) ppm.

Signal for *i*-C_6F_5 was not observed.

$^{11}B\{^{1}H\}$ NMR (160 MHz, THF-d_8): δ = −15.3 (s) ppm.

$^{19}F\{^{1}H\}$ NMR (565 MHz, THF-d_8): δ = −128.1 (s br, 6F, *m*-C_6F_5), −162.7-(−163.2) (m, 3F, *p*-C_6F_5), −167.1-(−167.7) (m, 6F, *o*-C_6F_5) ppm.

$B(C_6F_5)_3$-IDipp-Cu-4,4'Me_2Hdpa (C4)

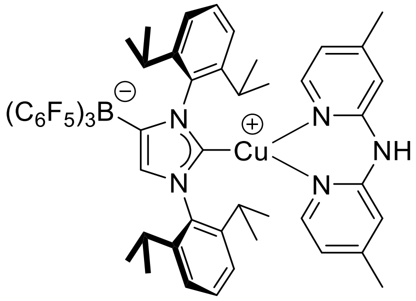

$B(C_6F_5)_3$-IDipp-Li(tol) (100 mg, 0.1 mmol, 1 eq.) was dissolved in toluene (5 mL), CuCl (17 mg, 0.17 mmol, 1.7 eq.) was added and the suspension stirred for 23 h at rt. The resulting suspension was filtered over celite. The solution was added to **L4** (20 mg, 0.1 mmol, 1 eq.) and stirred for 2 h at rt. The volatiles were removed *in vacuo*, the resulting solid dissolved in a minimal amount of THF and layered with *n*-hexane. The supernatant solution was removed and the solid dried *in vacuo* to obtain **C4** as a white powder (91 mg, 0.073 mmol, 73 %). Single crystals suitable for scXRD were obtained by layering a solution in THF with *n*-pentane at −40 °C.

Elemental analysis (%): calcd for $C_{55}H_{44}BCuF_{15}N_5{\cdot}C_7H_8$: C 61.23, H 4.58, N 5.58; found C 61.73, H 4.74, N 5.29.

^{1}H NMR (500 MHz, THF-d_8): δ = 8.75 (s br, 1H, NH), 7.42 (t, $^3J_{HH}$=7.8 Hz, 1H, *p*-Dipp$_A$), 7.41 (t, $^3J_{HH}$=7.8 Hz, 1H, *p*-Dipp$_B$), 7.23 (d, $^3J_{HH}$=7.8 Hz, *m*-Dipp$_A$), 7.20-7.05 (m, toluene), 7.09 (d, $^3J_{HH}$=7.8 Hz, 2H, *m*-Dipp$_B$), 6.50 (s br, 2H, $H_{3,Hdpa}$), 6.44 (s br, 1H, C$\underline{H}$=C), 6.26 (dd, $^3J_{HH}$=5.7 Hz, $^4J_{HH}$=1.2 Hz, 2H, $H_{5,Hdpa}$), 6.00 (d, $^3J_{HH}$=5.7 Hz, 2H, $H_{6,Hdpa}$), 3.15 (sept, $^3J_{HH}$=6.8 Hz, 2H, C$\underline{H}_B(CH_3)_2$), 2.92 (sept, $^3J_{HH}$=6.8 Hz, 2H, C$\underline{H}_A(CH_3)_2$), 2.30 (s, toluene), 2.18 (s, 6H, Ar-CH_3), 1.09 (d, $^3J_{HH}$=6.8 Hz, 6H, CH(C$\underline{H}_{3,A})_2$), 1.07 (d, $^3J_{HH}$=6.8 Hz, 6H, CH(C$\underline{H}_{3,B})_2$), 1.06 (d, $^3J_{HH}$=6.8 Hz, 6H, CH(C$\underline{H}_{3,A})_2$), 0.95 (d, $^3J_{HH}$=6.8 Hz, 6H, CH(C$\underline{H}_{3,B})_2$) ppm.

^{13}C{^{1}H} NMR (126 MHz, THF-d_8): δ = 182.4 (s, $C_{carbene}$), 154.2 (s, $C_{2,Hdpa}$), 151.3 (s, $C_{4,Hdpa}$), 149.7 (dm, $^1J_{CF}$=239 Hz, *o*-C_6F_5), 149.4 (s, $C_{6,Hdpa}$), 148.9 (q, $^1J_{BC}$ = 60 Hz, C=$\underline{C}$B), 148.4 (s, *o*-Dipp$_B$), 147.3 (s, *o*-Dipp$_A$), 140.4 (s, *i*-Dipp$_B$), 139.4 (dm, $^1J_{CF}$=243 Hz, *p*-C_6F_5), 138.4 (s, toluene), 138.3 (s, *i*-Dipp$_A$), 137.2 (dm, $^1J_{CF}$=243 Hz, *m*-C_6F_5), 131.4 (s br, $\underline{C}$H=C), 130.5 (s, *p*-Dipp$_A$), 130.0 (s, *p*-Dipp$_B$), 129.7 (s, toluene), 128.9 (s, toluene), 126.0 (s, toluene), 125.0 (s, *m*-Dipp$_A$), 124.3 (s, *m*-Dipp$_B$), 118.5 (s, $C_{5,Hdpa}$), 113.7 (s, $C_{3,Hdpa}$), 28.8 (s, $\underline{C}_A H(CH_3)_2$), 28.5 (s, $\underline{C}_B H(CH_3)_2$), 27.0 (s, CH($\underline{C}_B H_3)_2$), 24.4 (s, CH($\underline{C}_A H_3)_2$), 24.3 (s, CH($\underline{C}_B H_3)_2$), 22.1 (s, CH($\underline{C}_A H_3)_2$), 21.5 (s, toluene), 20.8 (s, Ar-$\underline{C}H_3$) ppm.

Signal for *i*-C_6F_5 was not observed.

^{11}B{^{1}H} NMR (160 MHz, THF-d_8): δ = −15.3 (s) ppm.

^{19}F{^{1}H} NMR (471 MHz, THF-d_8): δ = −128.2 (s br, 6F, *m*-C_6F_5), −163.0 (t, $^3J_{FF}$=20 Hz, 3F, *p*-C_6F_5), −167.1-(−167.7) (m, 6F, *o*-C_6F_5) ppm.

$B(C_6F_5)_3$-IDipp-Cu-5,5'Me_2Hdpa (C5)

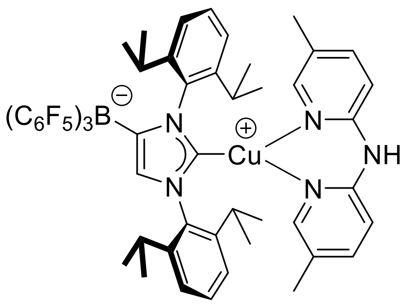

$B(C_6F_5)_3$-IDipp-Li(tol) (107 mg, 0.1 mmol, 1.0 eq.) was dissolved in toluene (5 mL), CuCl (21 mg, 0.21 mmol, 2.1 eq.) was added and the suspension stirred for 16 h at rt. The resulting suspension was filtered over celite. The solution was added to **L5** (20.3 mg, 0.1 mmol, 1.0 eq.) and stirred for 2 h at rt. The volatiles were removed *in vacuo* and the resulting solid washed with a minimal amount of toluene. The solid was dried *in vacuo* to obtain **C5** as a white powder (65 mg, 0.052 mmol, 52 %). Single crystals suitable for scXRD were obtained by layering a solution in THF with *n*-pentane at −40°C.

Elemental analysis (%): calcd for $C_{55}H_{44}BCuF_{15}N_5{\cdot}0.75C_7H_8$: C 60.66, H 4.50, N 5.68; found C 60.82 H 4.64, N 5.45.

^{1}H NMR (600 MHz, THF-d_8): δ = 8.70 (s br, 1H, NH), 7.38 (t, $^3J_{HH}$=7.6 Hz, 1H, *p*-Dipp$_B$), 7.36 (dd, $^3J_{HH}$=8.6 Hz, $^4J_{HH}$=2.4 Hz, 2H, $H_{4,Hdpa}$), 7.32 (t, $^3J_{HH}$=7.6 Hz, 1H, *p*-Dipp$_B$), 7.20-7.05 (m, toluene), 7.16 (d, $^3J_{HH}$=7.6 Hz, *m*-Dipp$_A$), 7.09 (d, $^3J_{HH}$=7.6 Hz, 2H, *m*-Dipp$_B$), 6.66 (d, $^3J_{HH}$=8.6 Hz, 2H, $H_{3,Hdpa}$), 6.40 (s br, 1H, C$\underline{H}$=C), 6.40-6.37 (m, 2H, $H_{6,Hdpa}$), 3.14 (sept, $^3J_{HH}$=6.8 Hz, 2H, C$\underline{H}_B$(CH$_3$)$_2$), 2.90 (sept, $^3J_{HH}$=6.8 Hz, 2H, C$\underline{H}_A$(CH$_3$)$_2$), 2.30 (s, toluene), 2.00 (s, 6H, Ar-CH$_3$), 1.09 (d, $^3J_{HH}$=6.8 Hz, 6H, CH(C$\underline{H}_{3,A}$)$_2$), 1.07 (d, $^3J_{HH}$=6.8 Hz, 6H, CH(C$\underline{H}_{3,B}$)$_2$), 1.06 (d, $^3J_{HH}$=6.8 Hz, 6H, CH(C$\underline{H}_{3,A}$)$_2$), 0.95 (d, $^3J_{HH}$=6.8 Hz, 6H, CH(C$\underline{H}_{3,B}$)$_2$) ppm.

^{13}C{^{1}H} NMR (151 MHz, THF-d_8): δ = 182.8 (s, $C_{carbene}$), 152.4 (s, $C_{2,Hdpa}$), 149.7 (dm, $^1J_{CF}$=239 Hz, *o*-C_6F_5), 148.9 (q, $^1J_{BC}$ = 60 Hz, C=$\underline{C}$B), 148.2 (s, *o*-Dipp$_B$), 147.9 (s, $C_{6,Hdpa}$), 146.8 (s, *o*-Dipp$_A$), 140.9 (s, $C_{4,Hdpa}$), 140.4 (s, *i*-Dipp$_B$), 139.4 (dm, $^1J_{CF}$=243 Hz, *p*-C_6F_5), 138.4 (s, toluene), 138.0 (s, *i*-Dipp$_A$), 137.4 (dm, $^1J_{CF}$=243 Hz, *m*-C_6F_5), 131.2 (s br, $\underline{C}$H=C), 130.4 (s, *p*-Dipp$_A$), 130.2 (s, *p*-Dipp$_B$), 129.7 (s, toluene), 128.9 (s, toluene), 127.0 (s, $C_{5,Hdpa}$), 126.0 (s, toluene), 124.5 (s, *m*-Dipp$_A$), 129.9 (s, *m*-Dipp$_B$), 113.8 (s, $C_{3,Hdpa}$), 28.8 (s, $\underline{C}_A$H(CH$_3$)$_2$), 28.5 (s, $\underline{C}_B$H(CH$_3$)$_2$), 26.9 (s, CH($\underline{C}_B$H$_3$)$_2$), 24.6 (s, CH($\underline{C}_A$H$_3$)$_2$), 24.0 (s, CH($\underline{C}_A$H$_3$)$_2$), 22.2 (s, CH($\underline{C}_B$H$_3$)$_2$), 21.5 (s, toluene), 18.0 (s, Ar-$\underline{C}$H$_3$) ppm.

Signal for *i*-C_6F_5 was not observed.

^{11}B{^{1}H} NMR (160 MHz, THF-d_8): δ = −15.3 (s) ppm.

^{19}F{^{1}H} NMR (471 MHz, THF-d_8): δ = −125.2 (s br, 6F, *m*-C_6F_5), −163.1 (t, $^3J_{FF}$=20 Hz, 3F, *p*-C_6F_5), −167.1-(−167.7) (m, 6F, *o*-C_6F_5) ppm.

$B(C_6F_5)_3$-IDipp-Cu-6,6'Me_2Hdpa (C6)

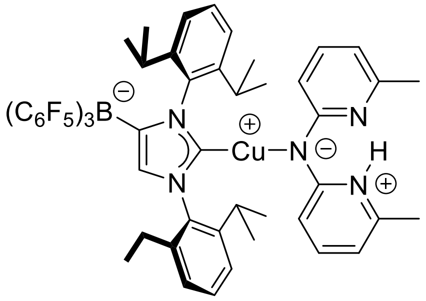

$B(C_6F_5)_3$-IDipp-Li(tol) (105 mg, 0.1 mmol, 1.0 eq) and CuCl (20.7 mg, 0.2 mmol, 2.0 eq.) were suspended in toluene (5 mL) and stirred for 18 h at rt. The resulting suspension was filtered *via* celite and added to a solution of **L6** (19.9 mg, 0.1 mmol, 1.0 eq.) in toluene (1 mL). The resulting suspension was after 2 h cooled to −40 °C causing further precipitation. The resulting solid was washed with a minimal amount of toluene and dried in vacuo to yield complex **C6** (52 mg, 0.042 mmol, 42 %). Single crystals suitable for scXRD were obtained by layering a solution in THF with *n*-pentane at −40 °C.

Elemental analysis (%): calcd for $C_{55}H_{44}BCuF_{15}N_5{\cdot}0.4C_7H_8$: C 59.84, H 4.38, N 5.84; found C 59.67 H 4.61, N 6.04.

^{1}H NMR (500 MHz, THF-*d*$_8$): δ = 7.59 (t, $^{3}J_{HH}$=7.8 Hz, 1H, *p*-Dipp$_A$), 7.54 (t, $^{3}J_{HH}$=7.8 Hz, 1H, *p*-Dipp$_B$), 7.38 (d, $^{3}J_{HH}$=7.8 Hz, *m*-Dipp$_A$), 7.20 (d, $^{3}J_{HH}$=7.8 Hz, 2H, *m*-Dipp$_B$), 7.10 (dd, $^{3}J_{HH}$=7.4 Hz, $^{3}J_{HH}$=8.5 Hz, 2H, H$_{6,6'MeHdpa}$), 6.64 (s br, 1H, C$\underline{H}$=C), 6.58 (d, $^{3}J_{HH}$=7.4 Hz, 2H, H$_{6,6'MeHdpa}$), 5.42 (d, $^{3}J_{HH}$=8.5 Hz, 2H, H$_{6,6'MeHdpa}$), 3.08 (sept, $^{3}J_{HH}$=6.8 Hz, 2H, C$\underline{H}_B$(CH$_3$)$_2$), 2.90 (sept, $^{3}J_{HH}$=6.8 Hz, 2H, C$\underline{H}_A$(CH$_3$)$_2$), 2.40 (s, 6H, pyr-C$\underline{H}_3$) 1.20-1.13 (m, 18H, CH(C$\underline{H}_{3,A/B,}$)$_2$), 0.99 (d, $^{3}J_{HH}$=6.8 Hz, 6H, CH(C$\underline{H}_{3,B}$)$_2$) ppm.

^{13}C{^{1}H} NMR (126 MHz, THF-*d*$_8$): δ = 177.5 (s, C$_{carbene}$), 159.9 (s, C$_{2,Hdpa}$), 149.9 (s, C$_{6,Hdpa}$), 149.8 (dm, $^{1}J_{CF}$=239 Hz, *o*-C$_6$F$_5$), 148.8 (s, *o*-Dipp$_B$), 147.8 (s, *o*-Dipp$_A$), 140.9 (s, C$_{4,Hdpa}$), 150.0 (q, $^{1}J_{BC}$ = 60 Hz, C=$\underline{C}$B), 139.5 (dm, $^{1}J_{CF}$=243 Hz, *p*-C$_6$F$_5$), 138.7 (s, *i*-Dipp$_B$), 138.4 (s, toluene), 137.5 (dm, $^{1}J_{CF}$=243 Hz, *m*-C$_6$F$_5$), 136.5 (s, *i*-Dipp$_A$), 131.1 (s br, $\underline{C}$H=C), 130.8 (s, *p*-Dipp$_A$), 130.3 (s, *p*-Dipp$_B$), 129.5 (s, toluene), 128.9 (s, toluene), 126.0 (s, toluene), 124.9 (s, *m*-Dipp$_A$), 124.1 (s, *m*-Dipp$_B$), 116.8 (s, C$_{3,Hdpa}$), 114.5 (s, C$_{5,Hdpa}$), 29.0 (s, $\underline{C}_A$H(CH$_3$)$_2$), 28.6 (s, $\underline{C}_B$H(CH$_3$)$_2$), 27.6 (s, CH($\underline{C}_B$H$_3$)$_2$), 24.9 (s, CH($\underline{C}_A$H$_3$)$_2$), 24.4 (s, CH($\underline{C}_A$H$_3$)$_2$), 22.1 (s, CH($\underline{C}_B$H$_3$)$_2$), 21.3 (s, toluene), 21.2 (s, 6H, Ar-CH$_3$) ppm.

Signal for *i*-C$_6$F$_5$ was not observed.

^{11}B{^{1}H} NMR (160 MHz, THF-*d*$_8$): δ = −15.5 (s) ppm.

^{19}F{^{1}H} NMR (471 MHz, THF-*d*$_8$): δ = −128.4 (s br, 6F, *m*-C$_6$F$_5$), −162.7 (t, $^{3}J_{FF}$=20 Hz, 3F, *p*-C$_6$F$_5$), −166.9-(−167.3) (m, 6F, *o*-C$_6$F$_5$) ppm.

3 NMR Spectra

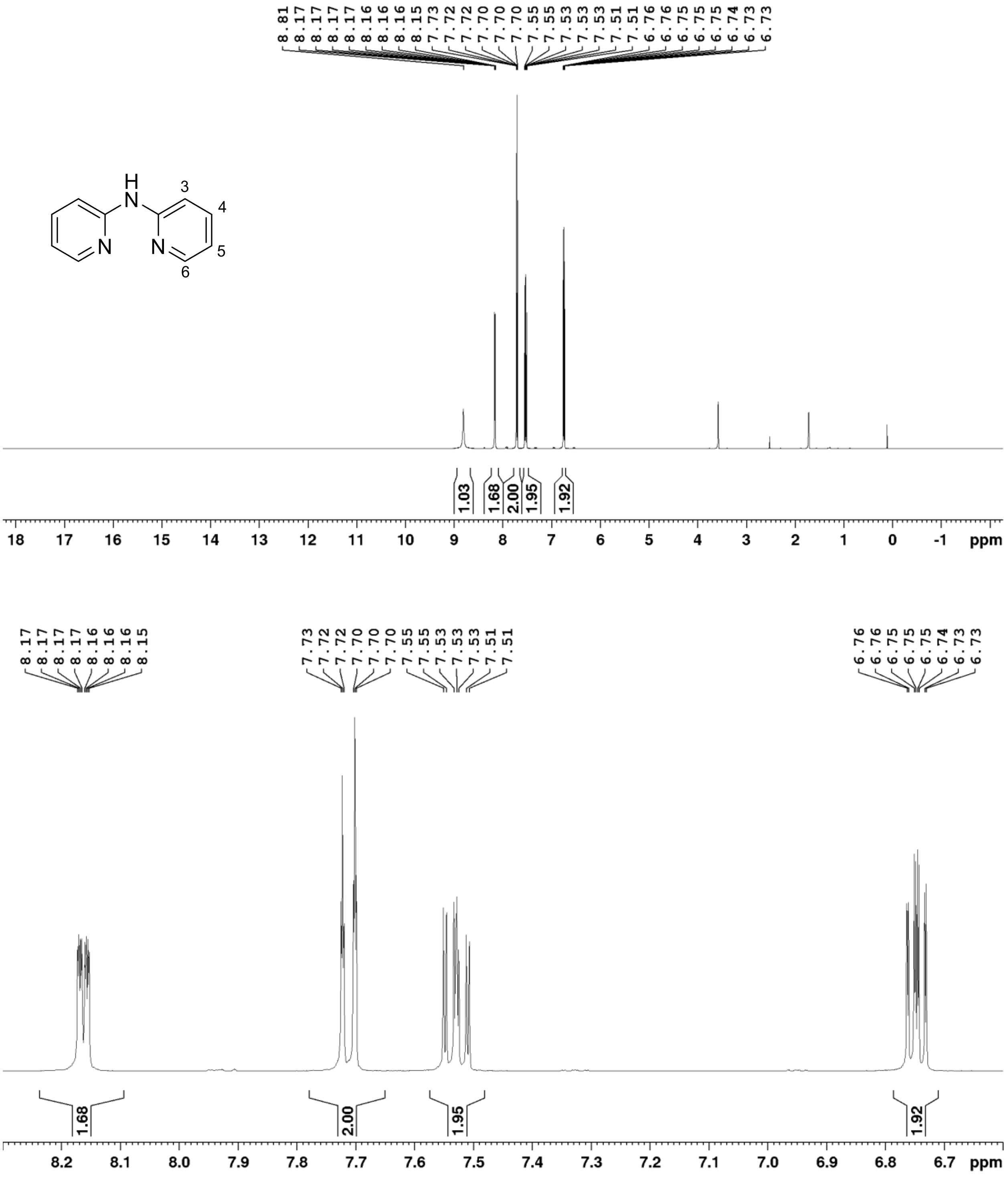

Figure S1. ^{1}H NMR of di(pyridin-2-yl)amine (**L2**) (top) and enlargement aromatic region (bottom) in THF-d_8.

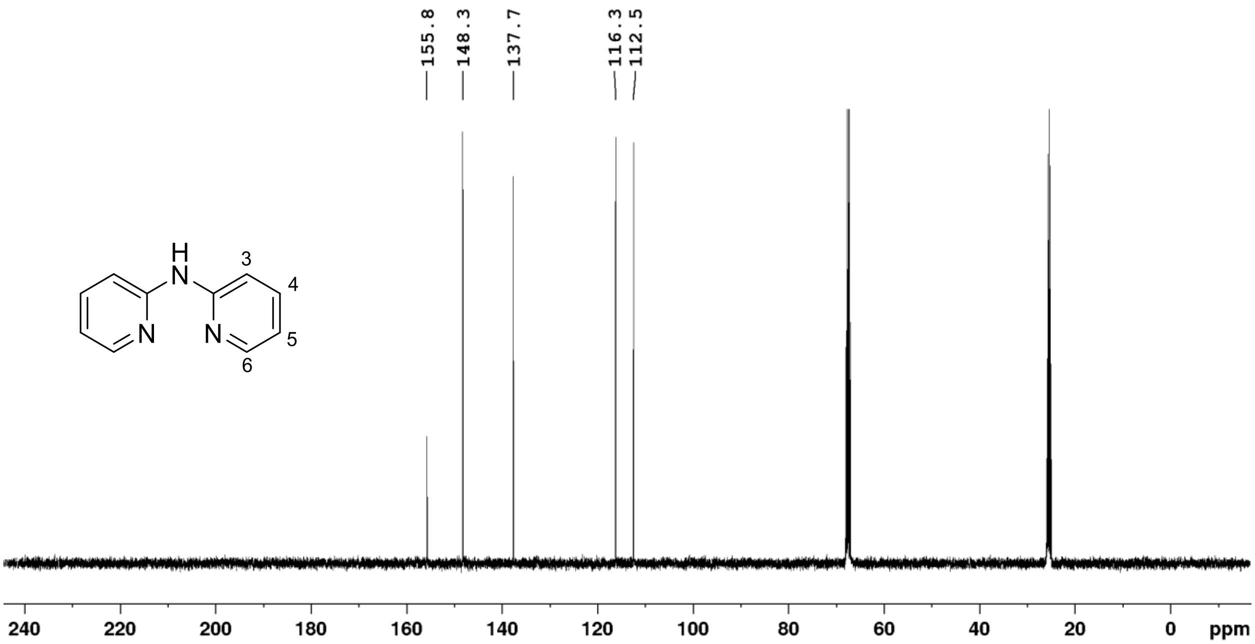

Figure S2. $^{13}C\{^1H\}$ NMR of di(pyridin-2-yl)amine (**L2**) in THF-d_8.

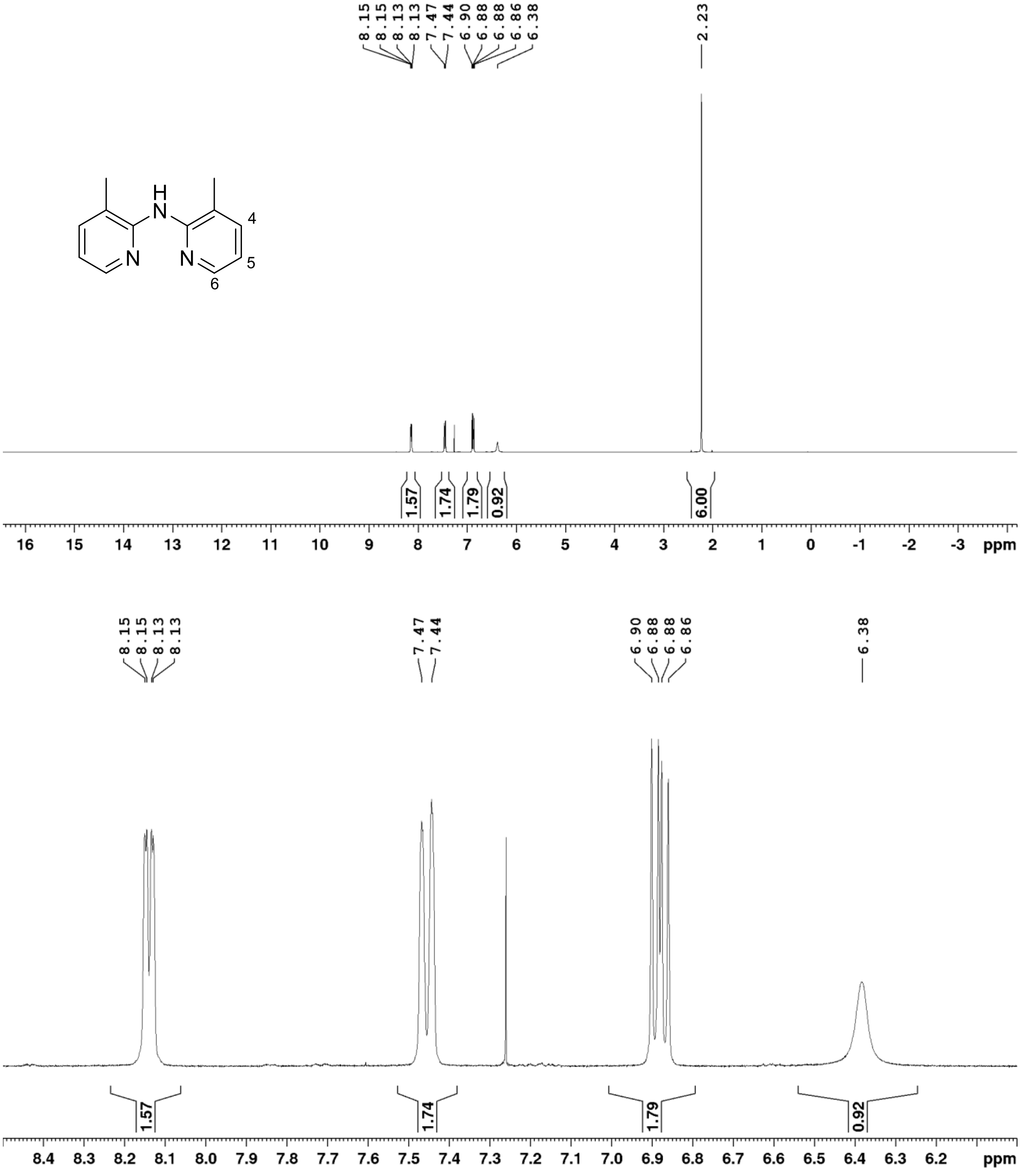

Figure S3. ^{1}H NMR of bis(3-methylpyridin-2-yl)amine (**L3**) (top) and enlargement aromatic region (bottom) in $CDCl_3$.

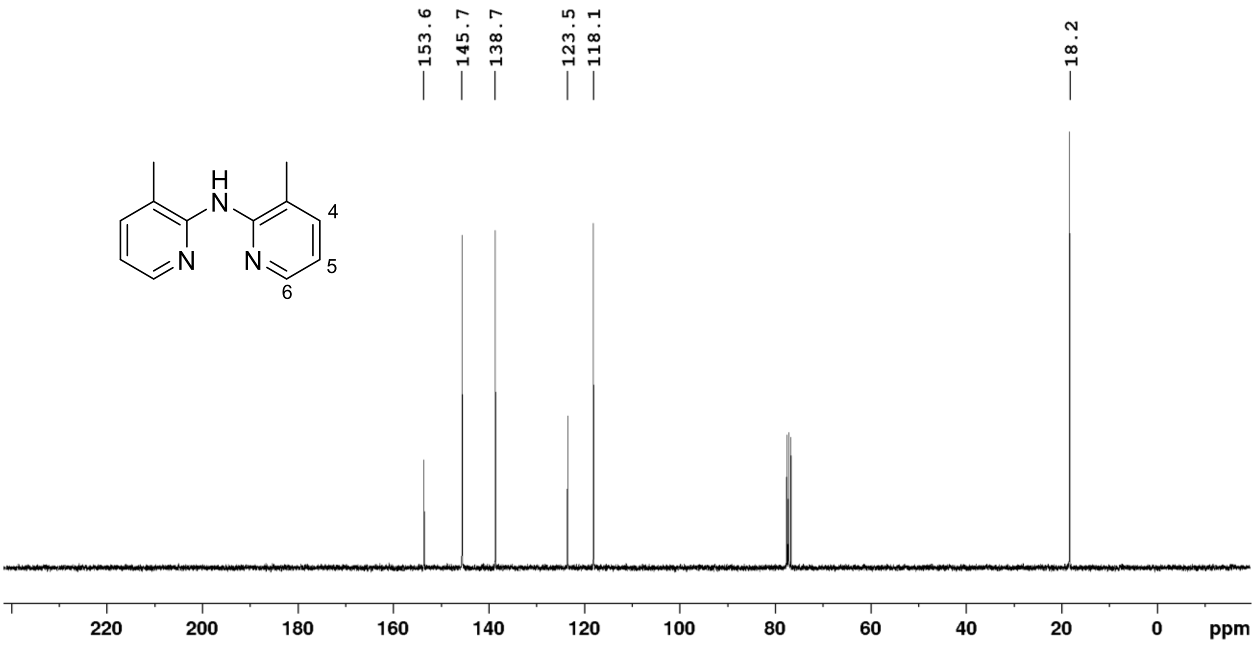

Figure S4. $^{13}C\{^{1}H\}$ NMR of bis(3-methylpyridin-2-yl)amine (**L3**) in $CDCl_3$.

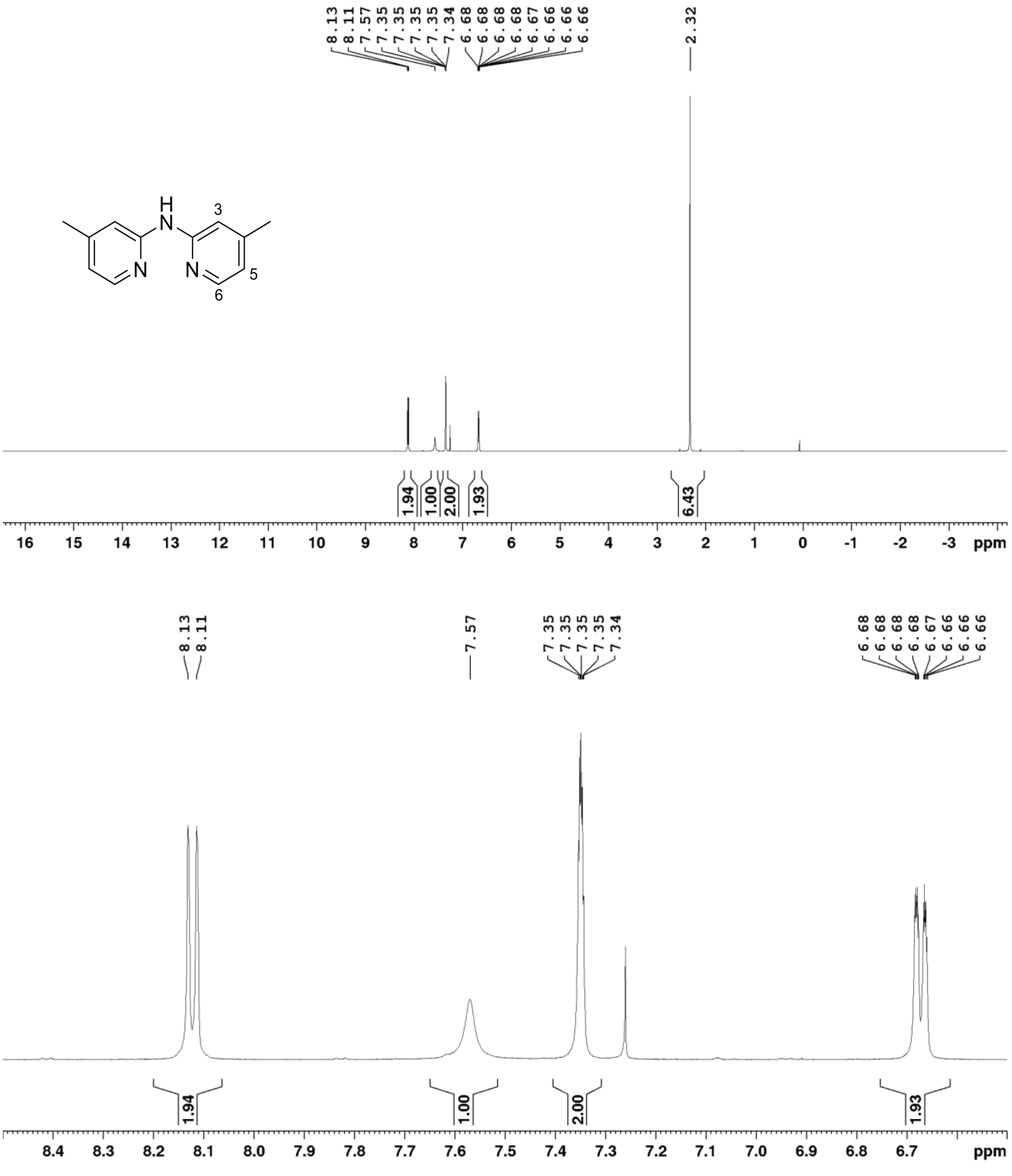

Figure S5. ^{1}H NMR of bis(4-methylpyridin-2-yl)amine (**L4**) (top) and enlargement of aromatic region (bottom) in $CDCl_3$.

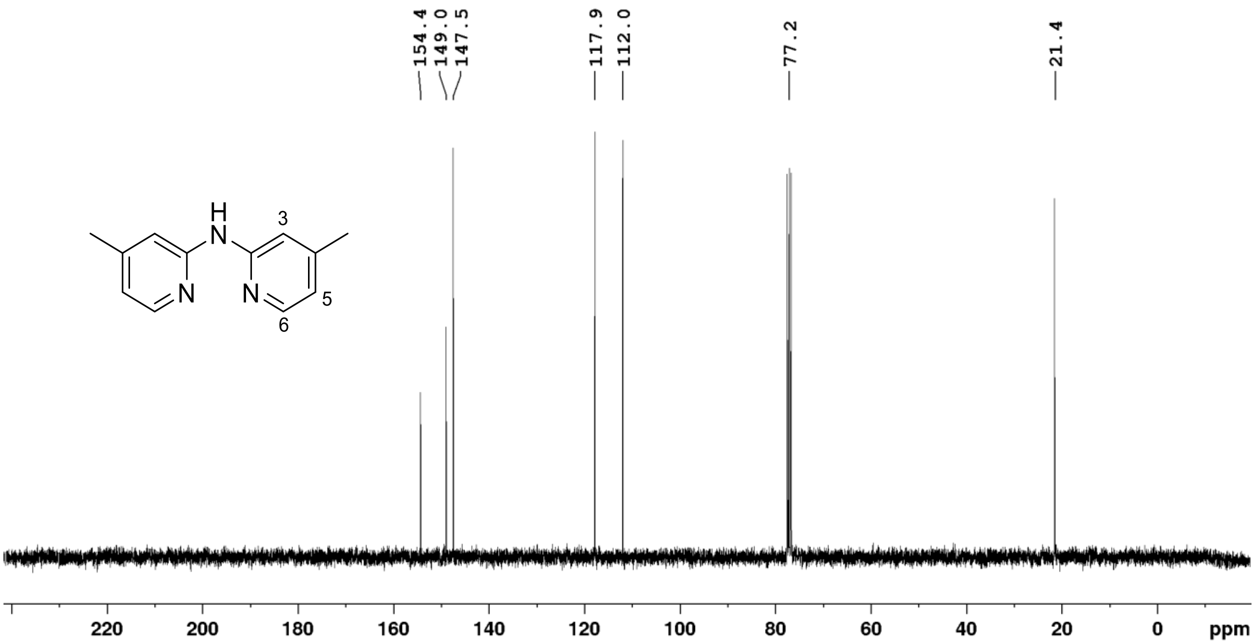

Figure S6. $^{13}C\{^{1}H\}$ NMR of bis(4-methylpyridin-2-yl)amine (**L4**) in $CDCl_3$.

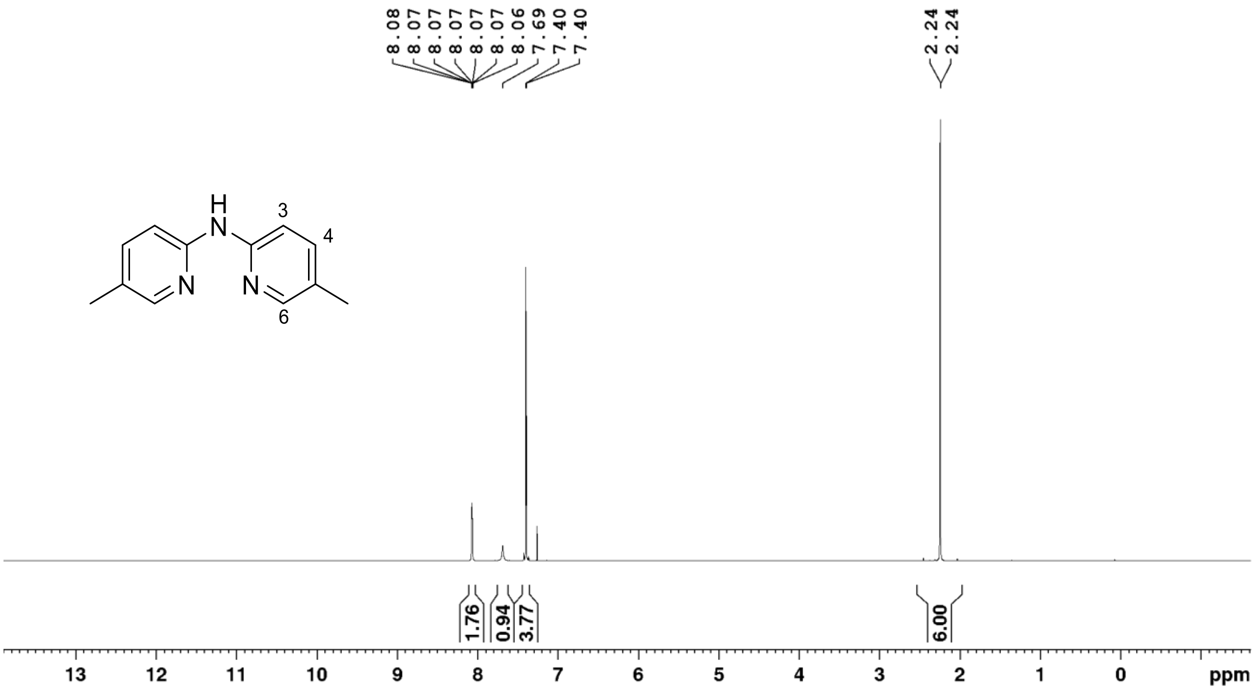

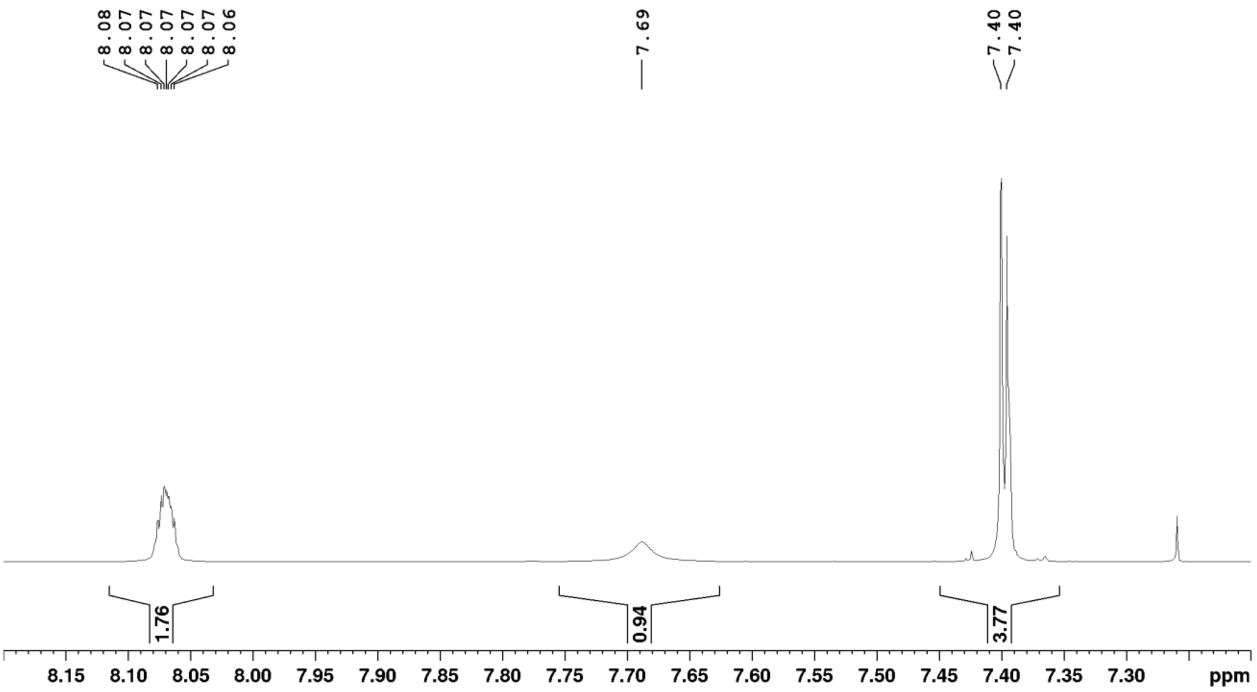

Figure S7. ^{1}H NMR of bis(5-methylpyridin-2-yl)amine (**L5**) (top) and enlargement of aromatic region (bottom) in $CDCl_3$.

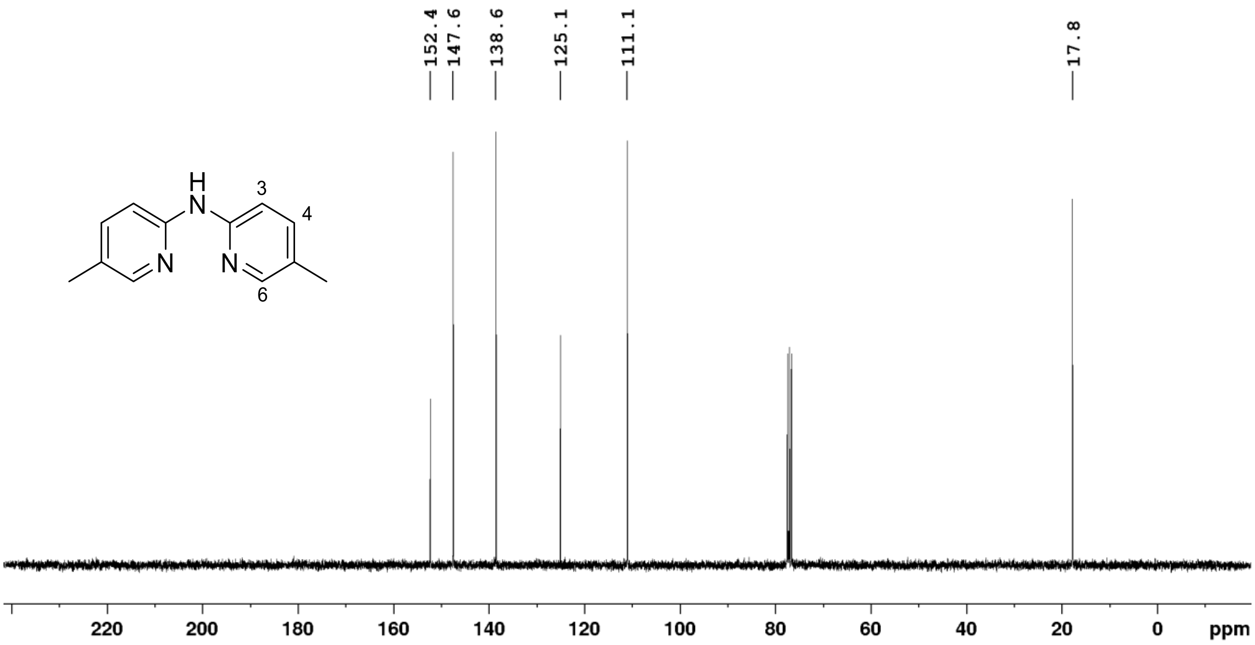

Figure S8. $^{13}C\{^{1}H\}$ NMR of bis(5-methylpyridin-2-yl)amine (**L5**) in $CDCl_3$.

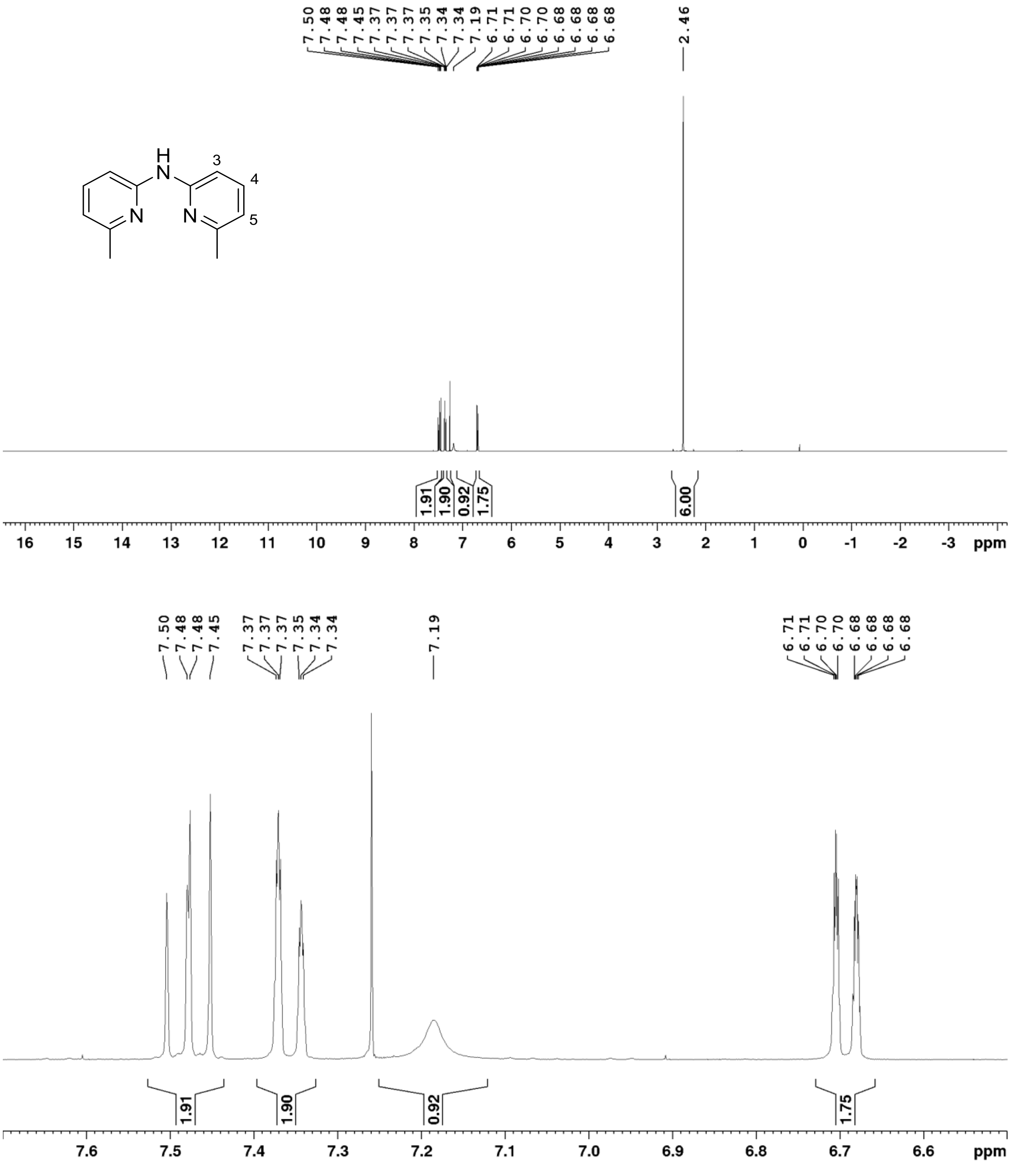

Figure S9. ^{1}H NMR of bis(6-methylpyridin-2-yl)amine (**L6**) (top) and enlargement of aromatic region (bottom) in $CDCl_3$.

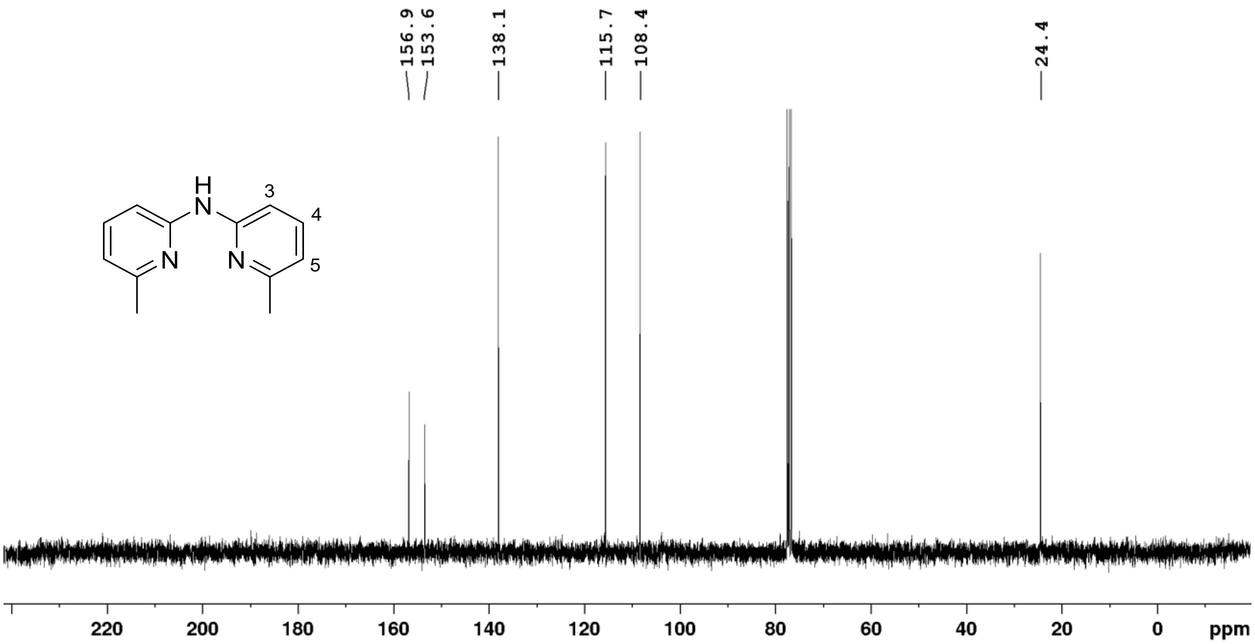

Figure S10. $^{13}C\{^{1}H\}$ NMR of bis(6-methylpyridin-2-yl)amine (**L6**) in $CDCl_3$.

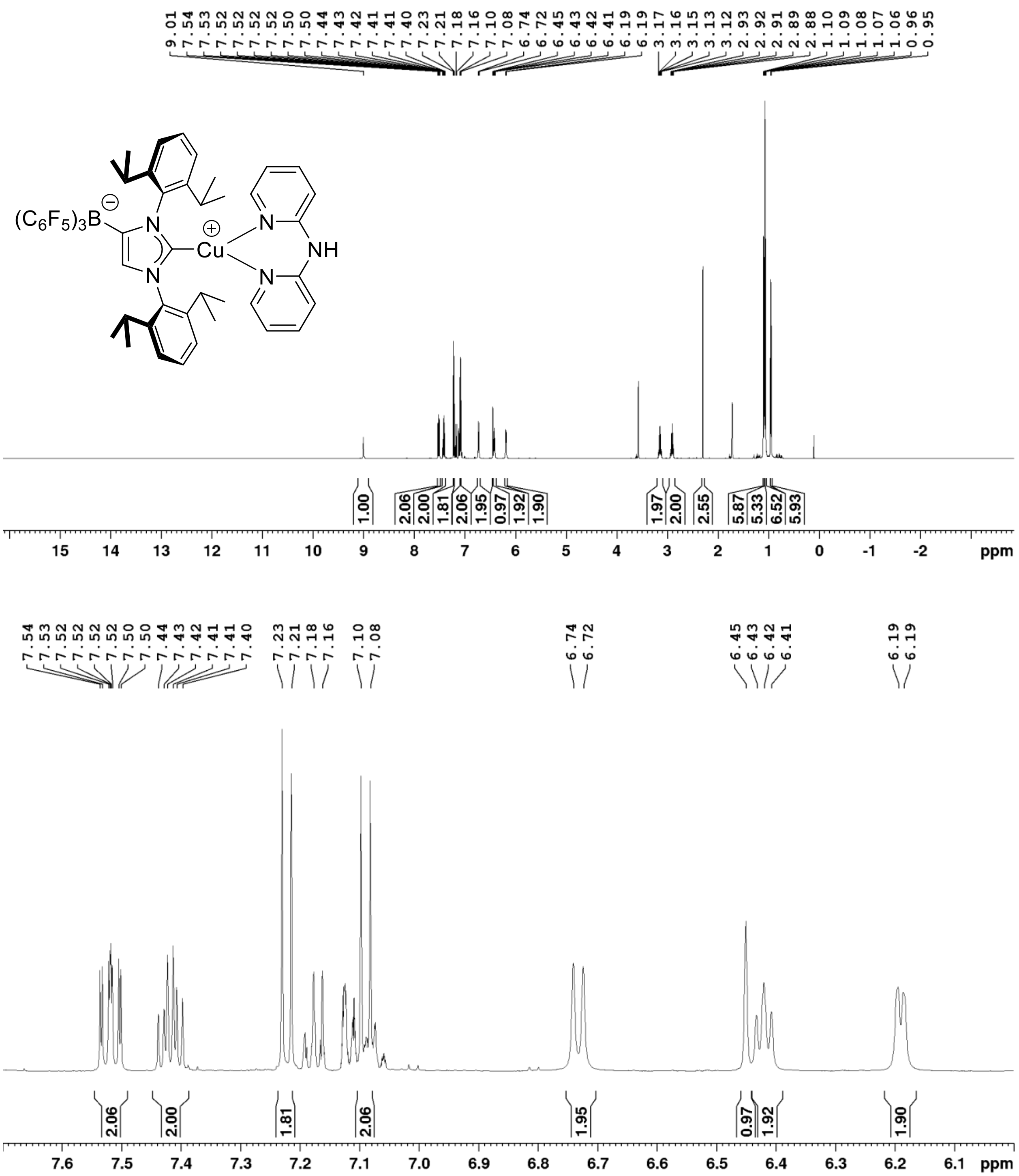

Figure S11. ^{1}H NMR of $B(C_6F_5)_3$-IDipp-Cu-Hdpa (**C2**) (top) and enlargement of aromatic region (bottom) in THF-d_8.

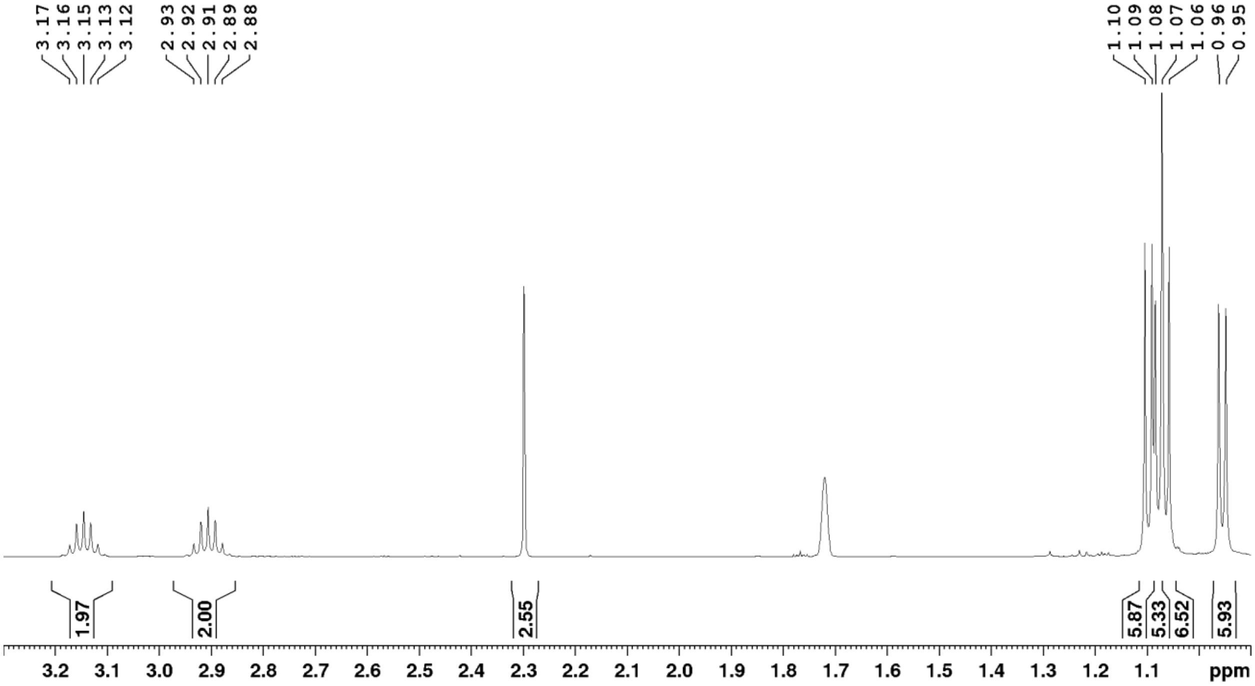

Figure S12. ^{1}H NMR of $B(C_6F_5)_3$-IDipp-Cu-Hdpa (**C2**) (aliphatic region enlarged) in THF-d_8.

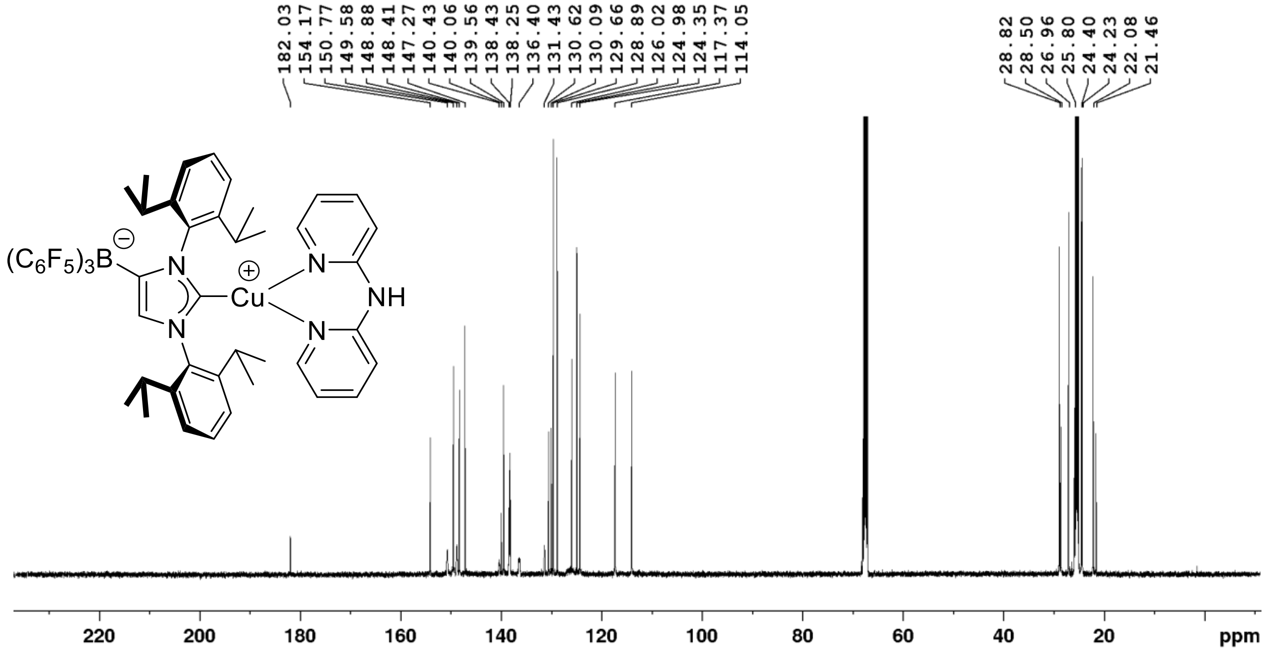

Figure S13. $^{13}C\{^{1}H\}$ NMR of $B(C_6F_5)_3$-IDipp-Cu-Hdpa (**C2**) in THF-d_8.

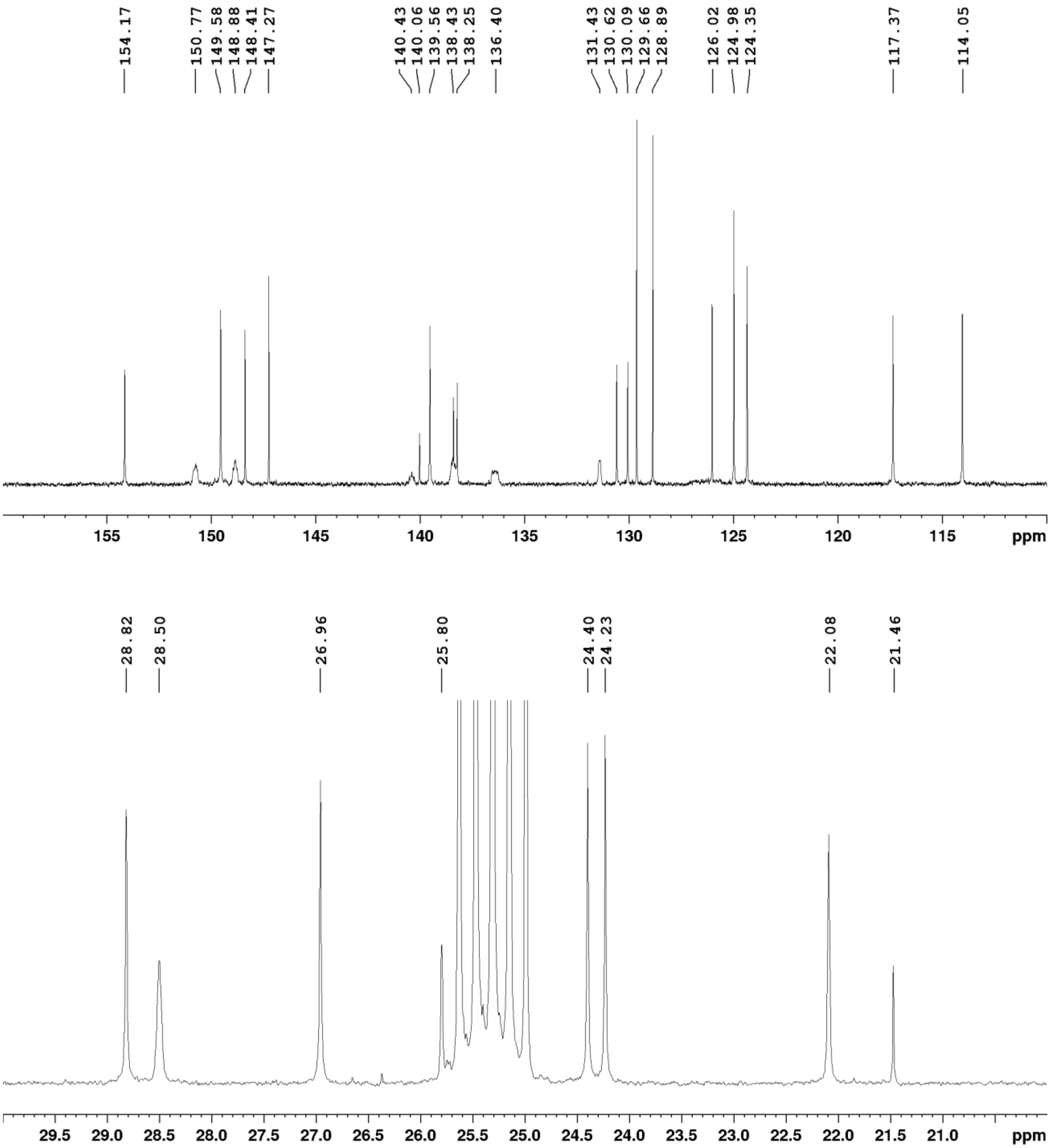

Figure S14. $^{13}C\{^{1}H\}$ NMR of $B(C_6F_5)_3$-IDipp-Cu-Hdpa (**C2**) with enlargement of aromatic region (top) and aliphatic region (bottom) in THF-d_8.

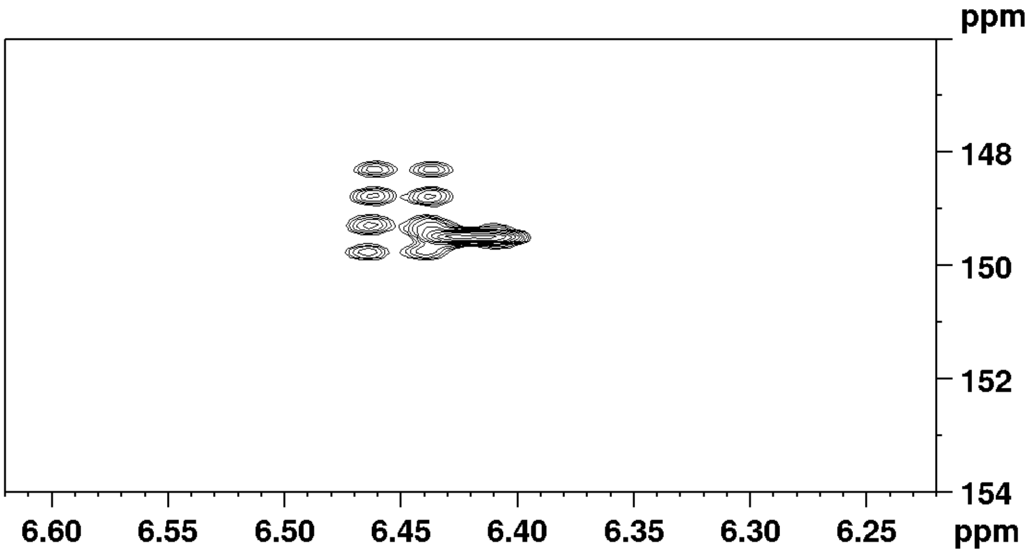

Figure S15. ^{1}H,^{13}C-HMBC of $B(C_6F_5)_3$-IDipp-Cu-Hdpa (**C2**) enlarged for identifying the signal for the boron bound carbon atom in the backbone in THF-d_8.

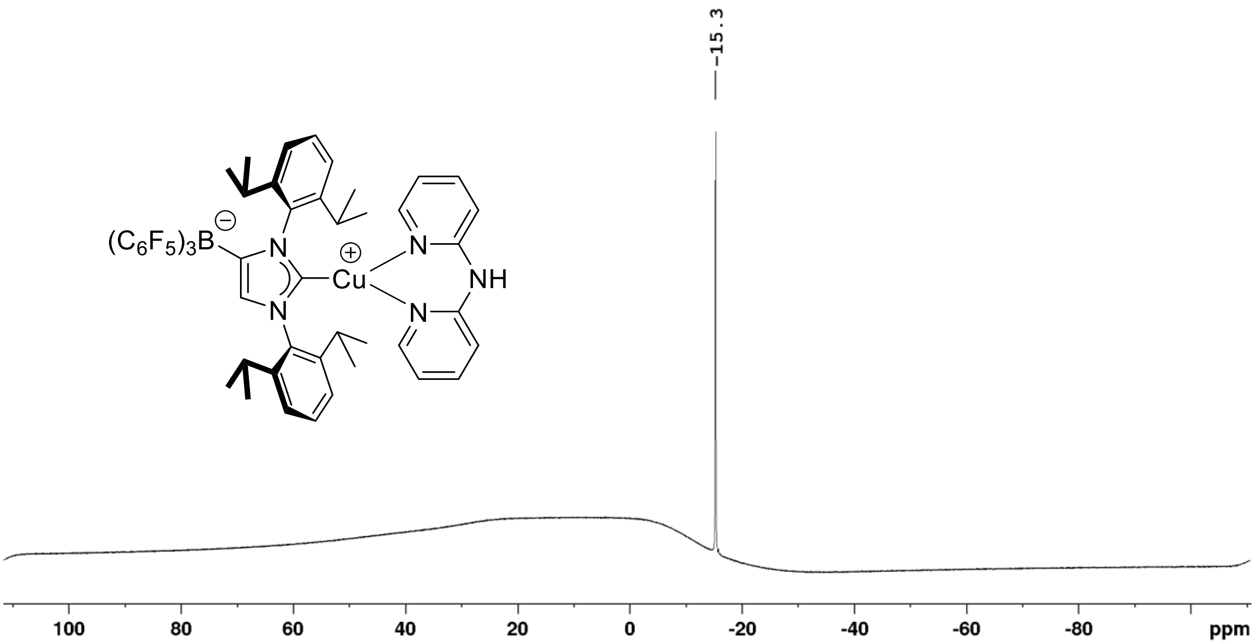

Figure S16. $^{11}B\{^{1}H\}$ NMR of $B(C_6F_5)_3$-IDipp-Cu-Hdpa (**C2**) in THF-d_8.

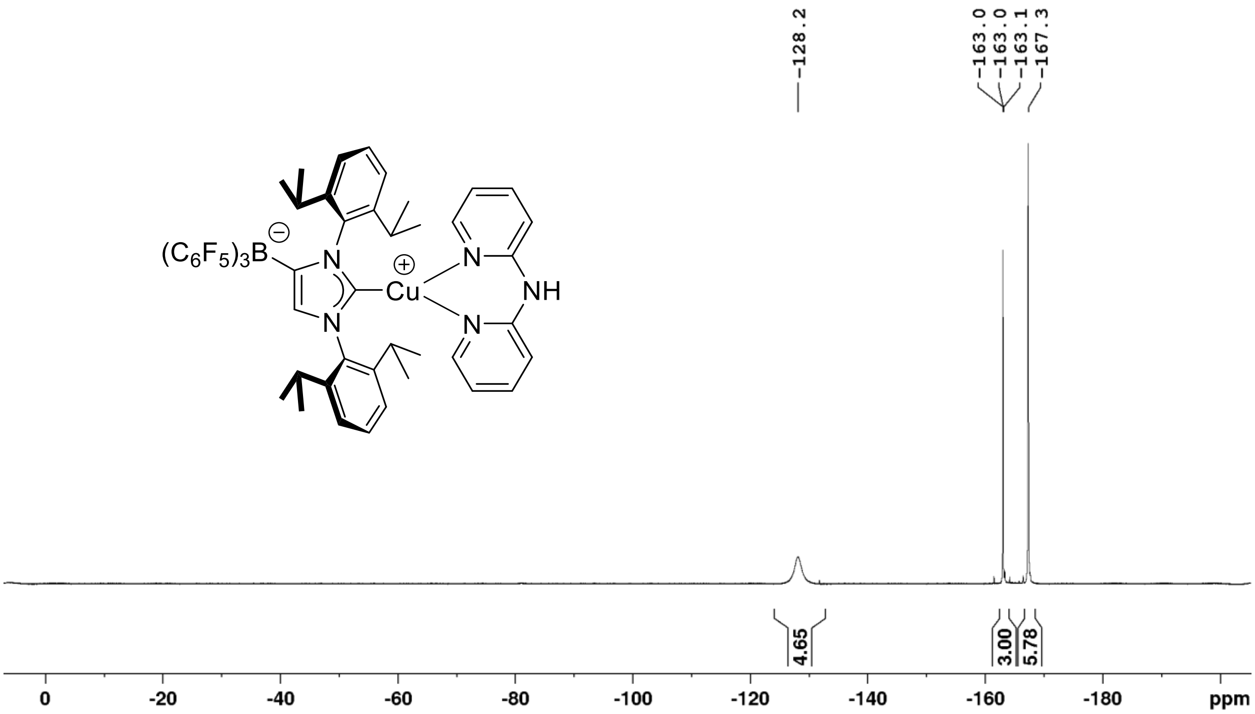

Figure S17. $^{19}F\{^{1}H\}$ NMR of $B(C_6F_5)_3$-IDipp-Cu-Hdpa (**C2**) in THF-d_8.

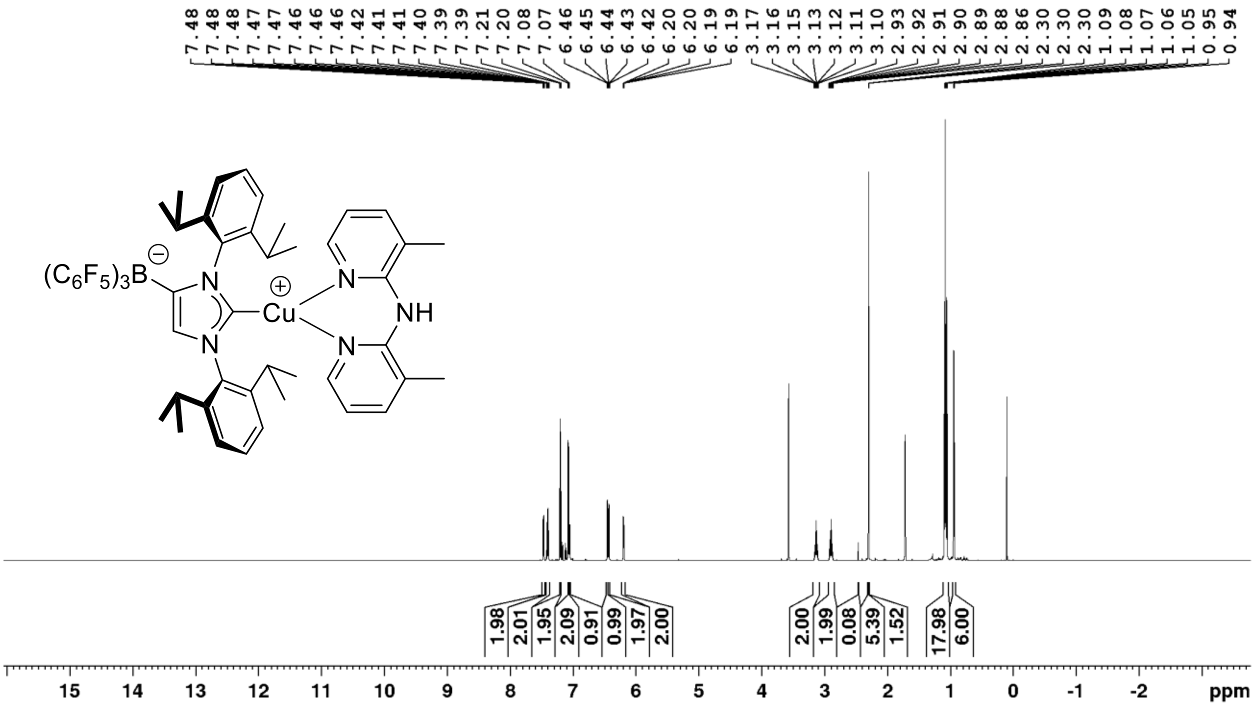

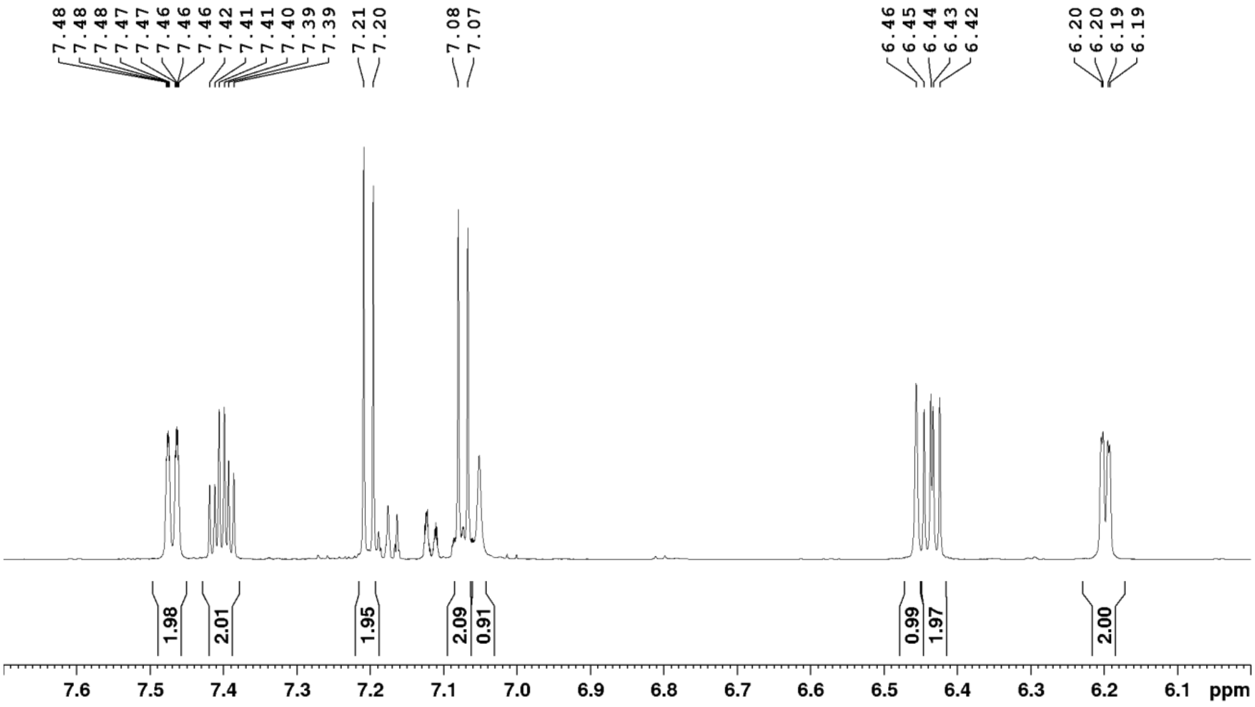

Figure S18. ^{1}H NMR of $B(C_6F_5)_3$-IDipp-Cu-3,3'Me_2Hdpa (**C3**) (top) and enlargement of aromatic region (bottom) in THF-d_8.

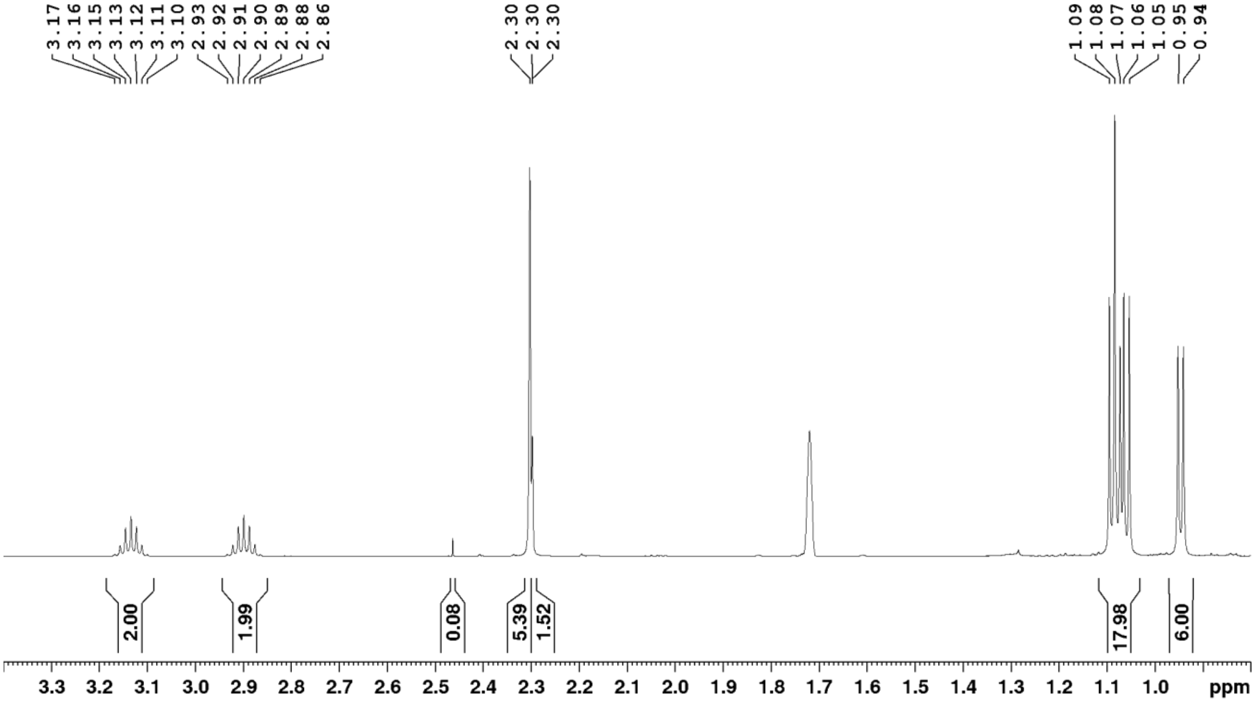

Figure S19. ^{1}H NMR of $B(C_6F_5)_3$-IDipp-Cu-3,3'Me_2Hdpa (**C3**) (aliphatic region enlarged) in THF-d_8.

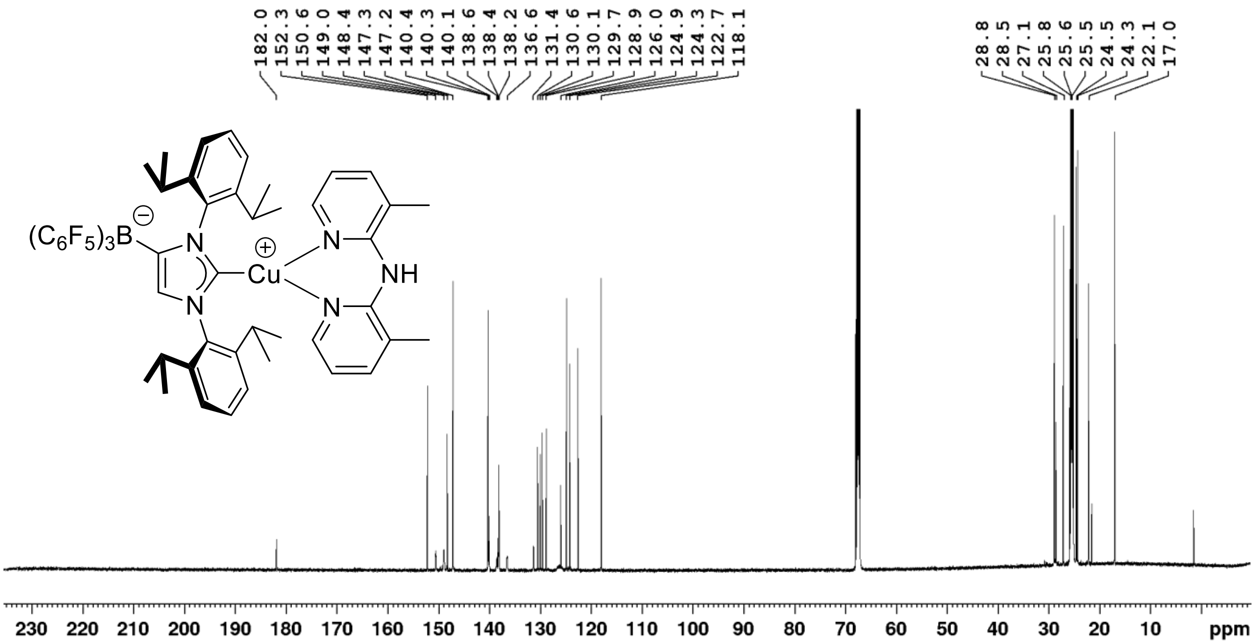

Figure S20. $^{13}C\{^{1}H\}$ NMR of $B(C_6F_5)_3$-IDipp-Cu-3,3'Me_2Hdpa (**C3**) in THF-d_8.

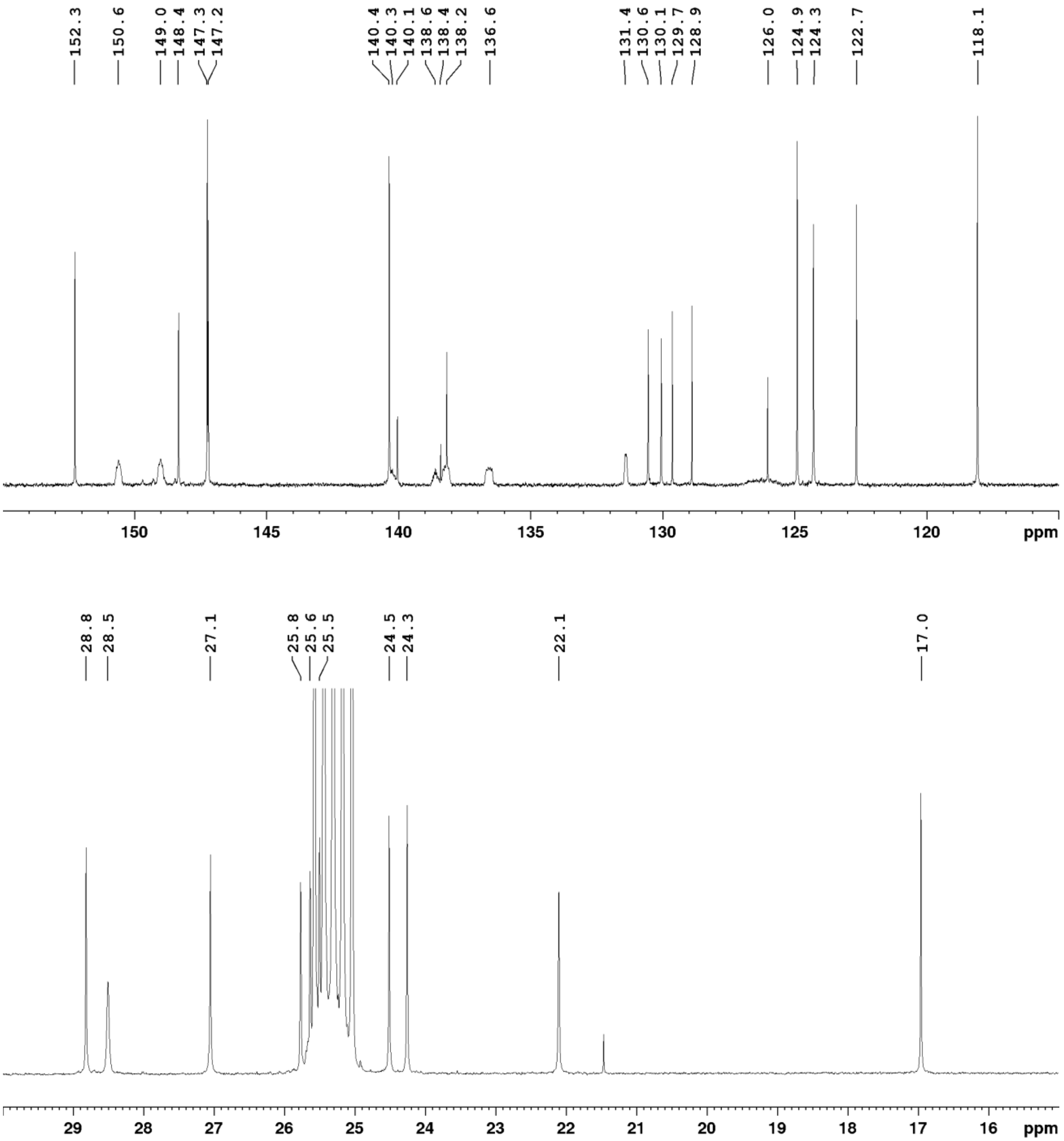

Figure S21. $^{13}C\{^{1}H\}$ NMR of $B(C_6F_5)_3$-IDipp-Cu-3,3'Me_2Hdpa (**C3**) with enlargement of aromatic region (top) and aliphatic region (bottom) in THF-d_8.

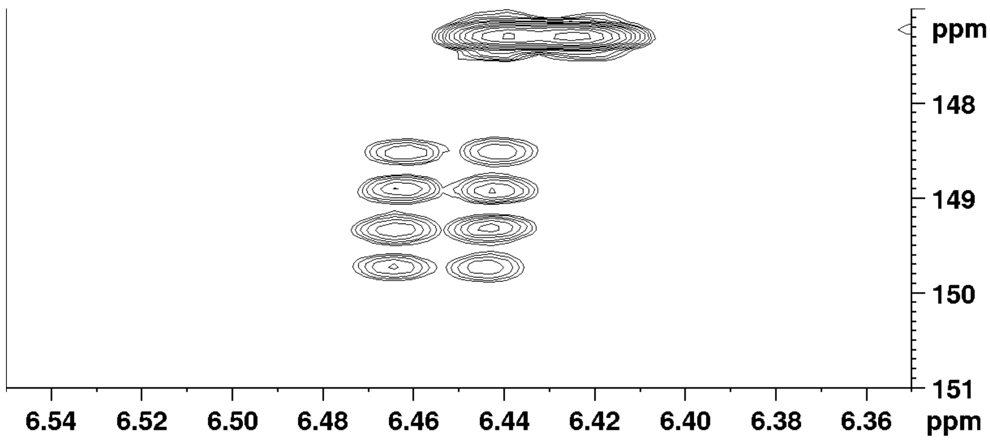

Figure S22. $^{1}H,^{13}C$-HMBC of $B(C_6F_5)_3$-IDipp-Cu-3,3'Me_2Hdpa (**C3**) enlarged for identifying the signal for the boron bound carbon atom in the backbone in THF-d_8.

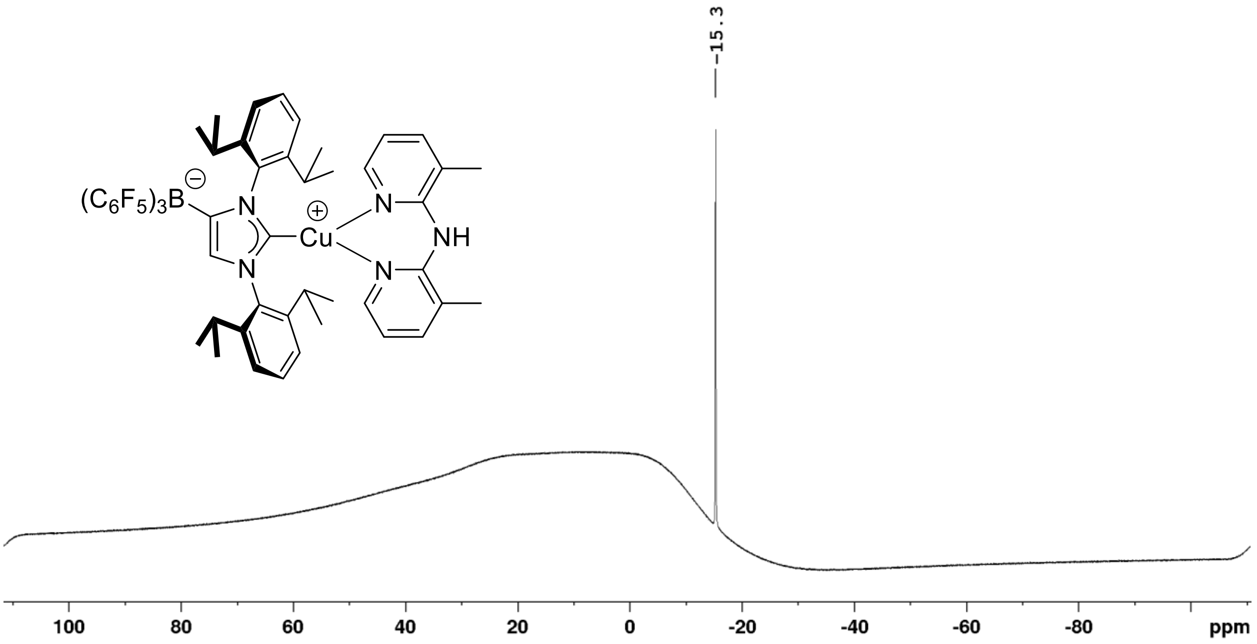

Figure S23. $^{11}B\{^{1}H\}$ NMR of $B(C_6F_5)_3$-IDipp-Cu-3,3'Me_2Hdpa (**C3**) in THF-d_8.

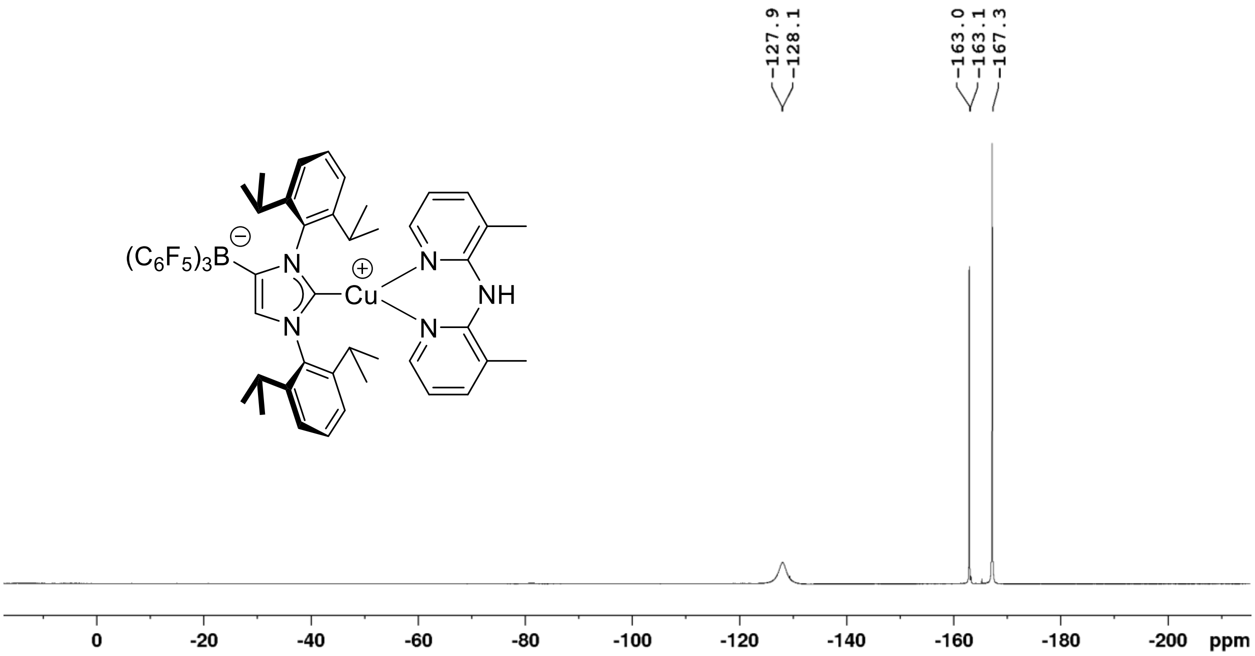

Figure S24. $^{19}F\{^1H\}$ NMR of $B(C_6F_5)_3$-IDipp-Cu-3,3'Me_2Hdpa (**C3**) in THF-d_8.

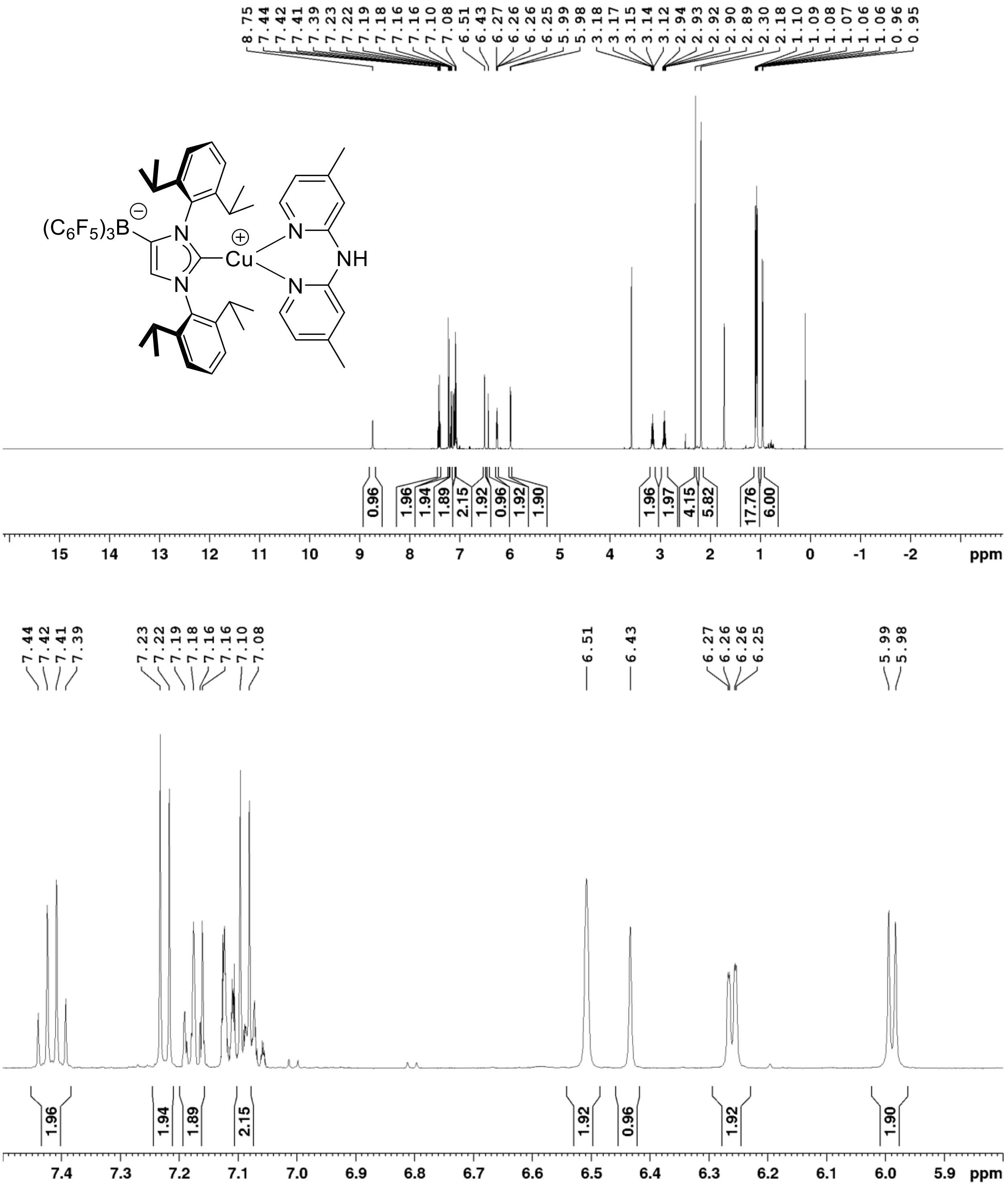

Figure S25. ^{1}H NMR of $B(C_6F_5)_3$-IDipp-Cu-4,4'Me_2Hdpa (**C4**) (top) and enlargement of aromatic region (bottom) in THF-d_8.

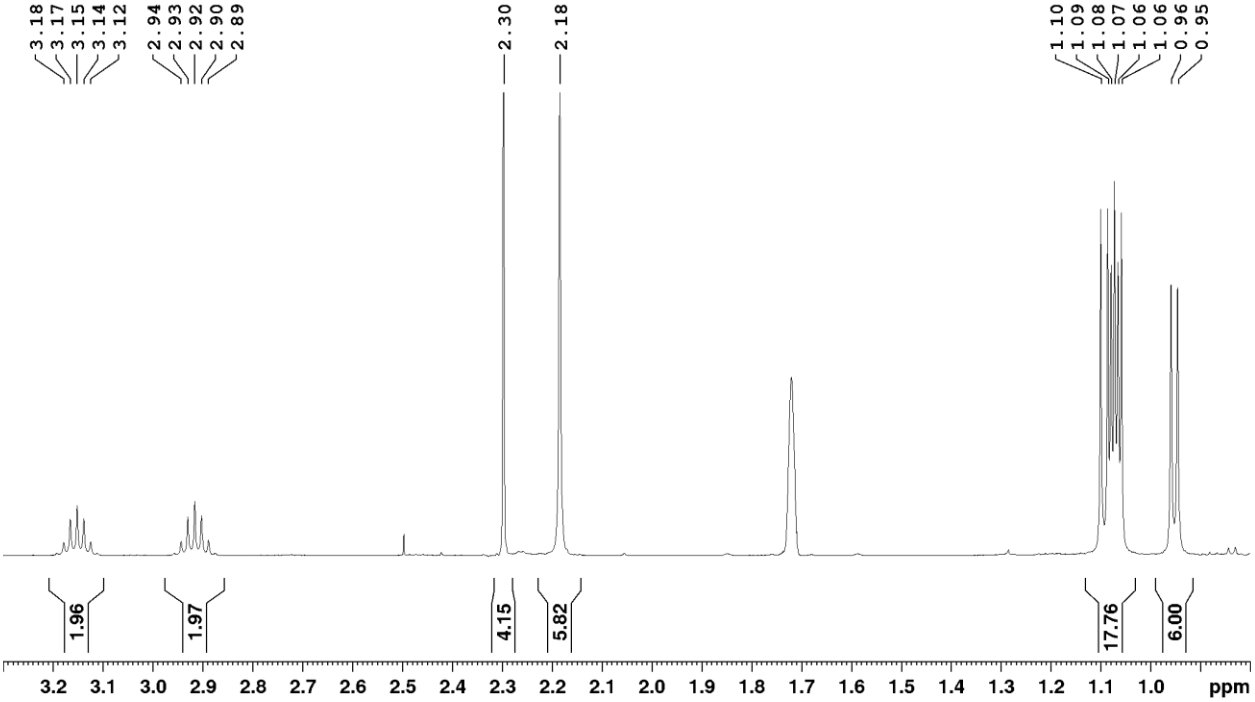

Figure S26. ^{1}H NMR of $B(C_6F_5)_3$-IDipp-Cu-4,4'Me_2Hdpa (**C4**) (aliphatic region enlarged) in THF-d_8.

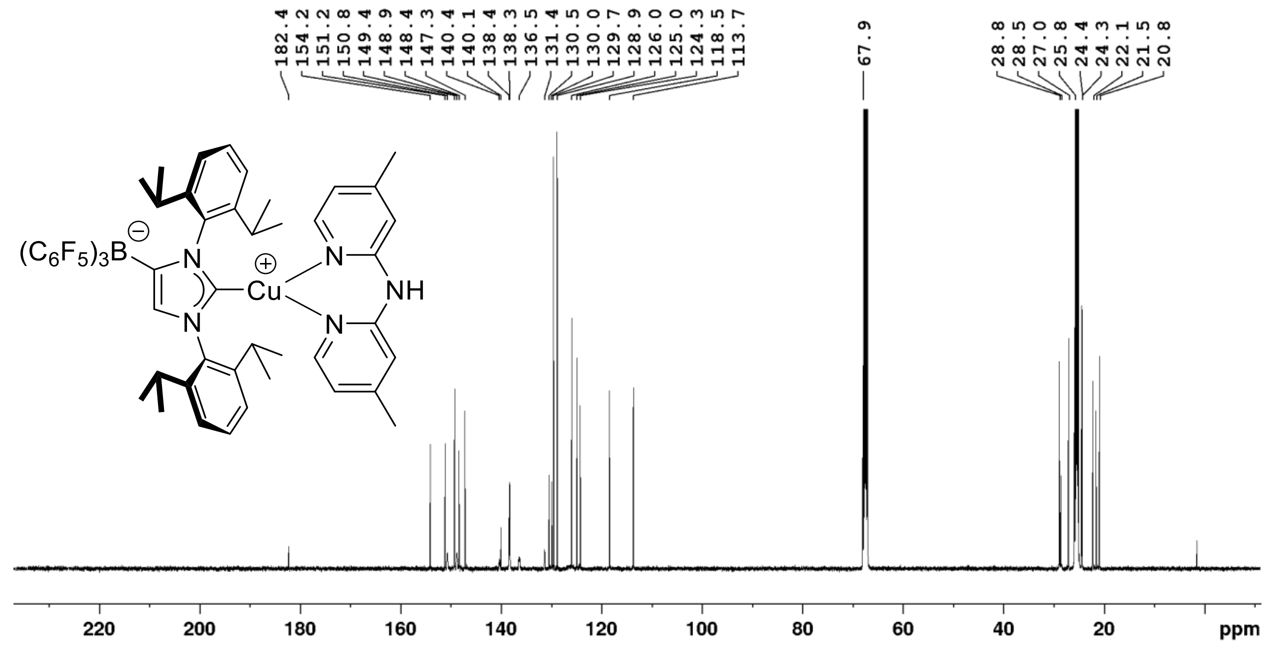

Figure S27. $^{13}C\{^1H\}$ NMR of $B(C_6F_5)_3$-IDipp-Cu-4,4'Me_2Hdpa (**C4**) in THF-d_8.

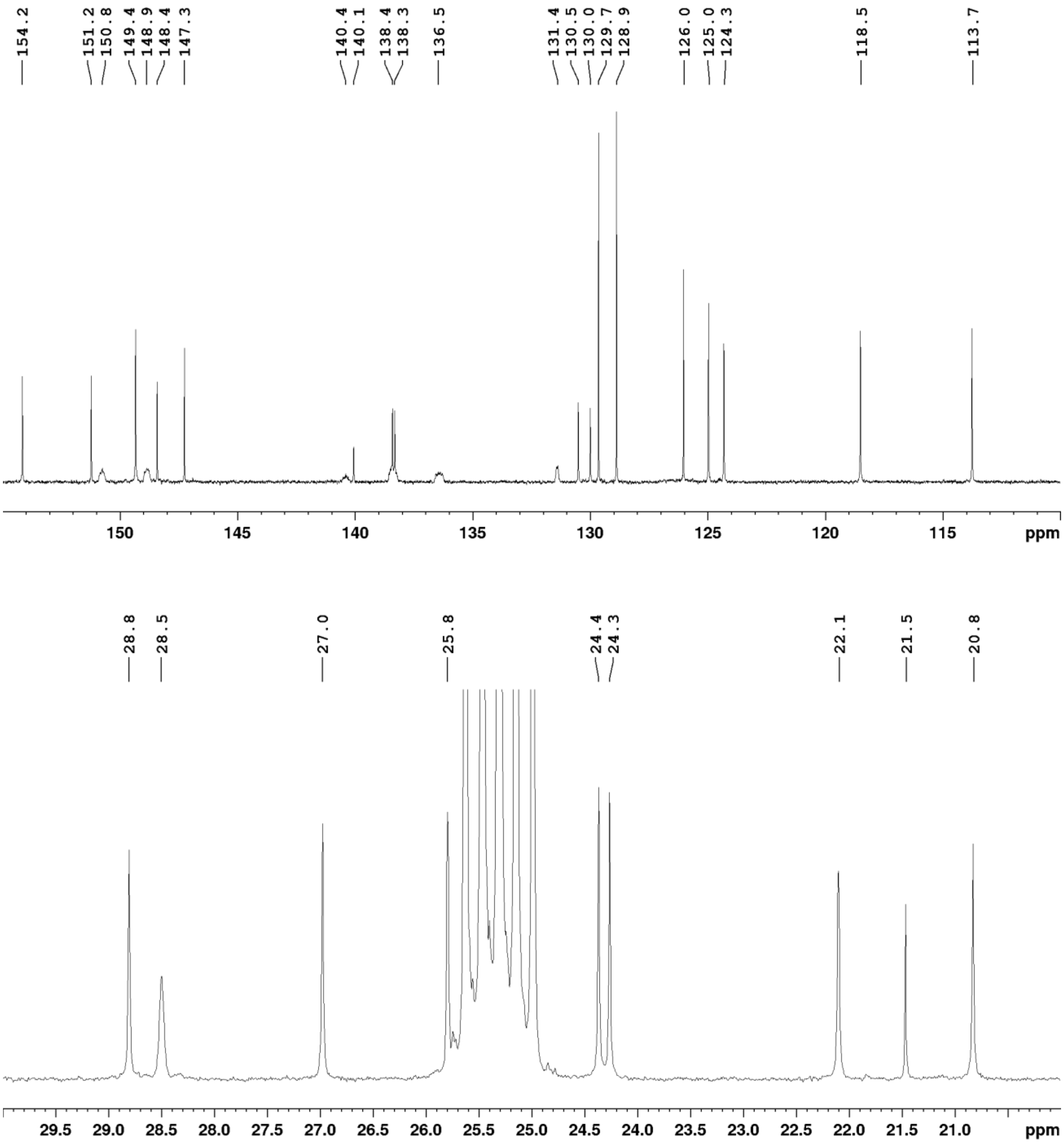

Figure S28. $^{13}C\{^{1}H\}$ NMR of $B(C_6F_5)_3$-IDipp-Cu-4,4'Me_2Hdpa (**C4**) with enlargement of aromatic region (top) and aliphatic region (bottom) in THF-d_8.

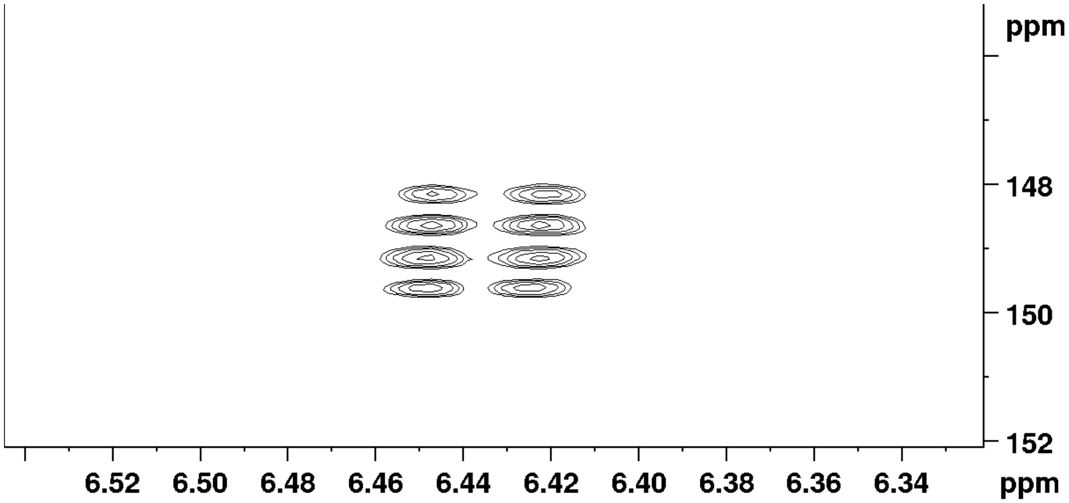

Figure S29. $^{1}H,^{13}C$-HMBC of $B(C_6F_5)_3$-IDipp-Cu-4,4'Me_2Hdpa (**C4**) enlarged for identifying the signal for the boron bound carbon atom in the backbone in THF-d_8.

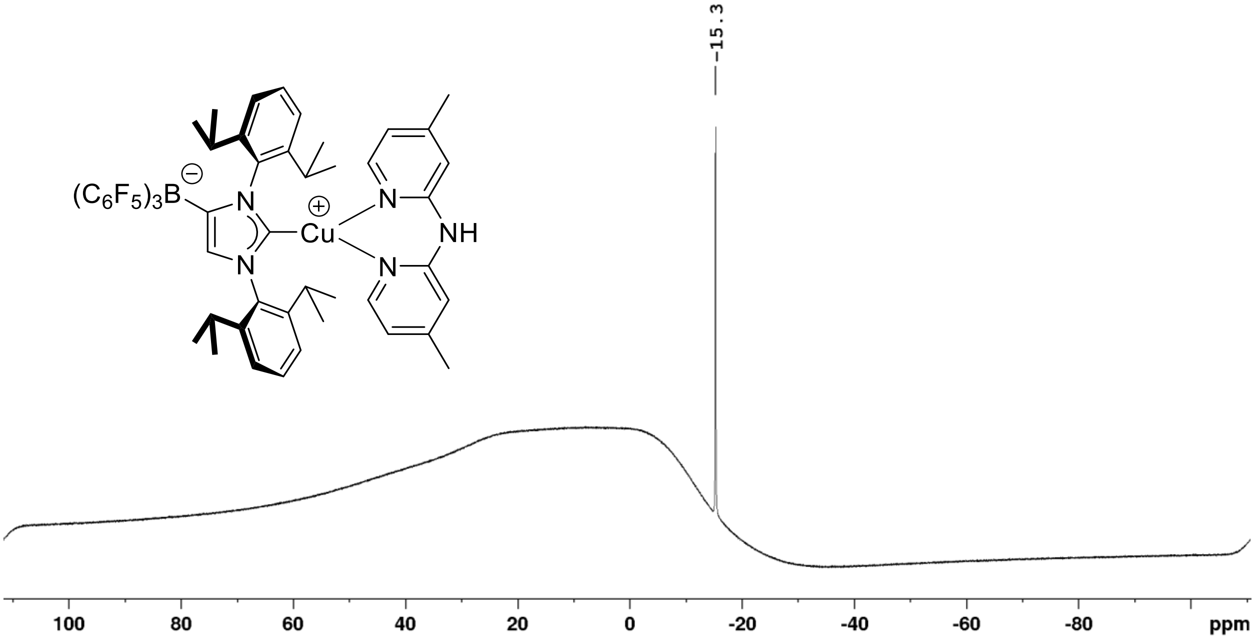

Figure S30. $^{11}B\{^{1}H\}$ NMR of $B(C_6F_5)_3$-IDipp-Cu-4,4'Me_2Hdpa (**C4**) in THF-d_8.

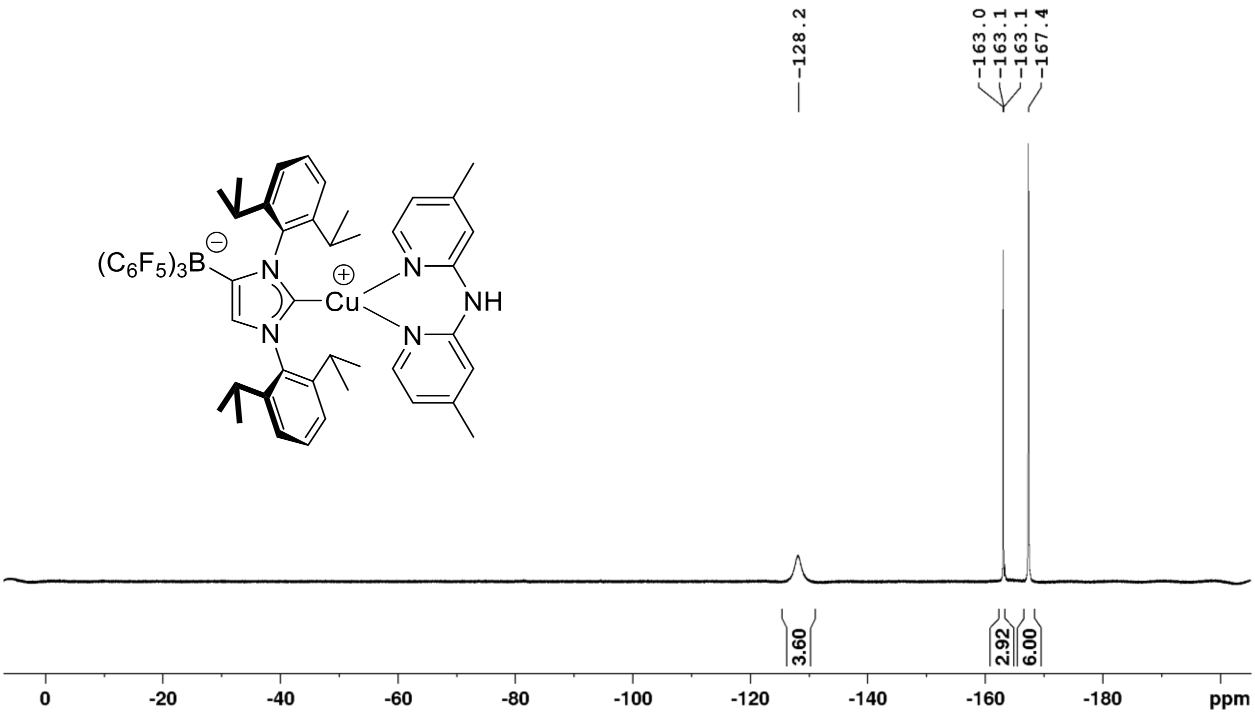

Figure S31. $^{11}B\{^1H\}$ NMR of $B(C_6F_5)_3$-IDipp-Cu-4,4'Me_2Hdpa (**C4**) in THF-d_8.

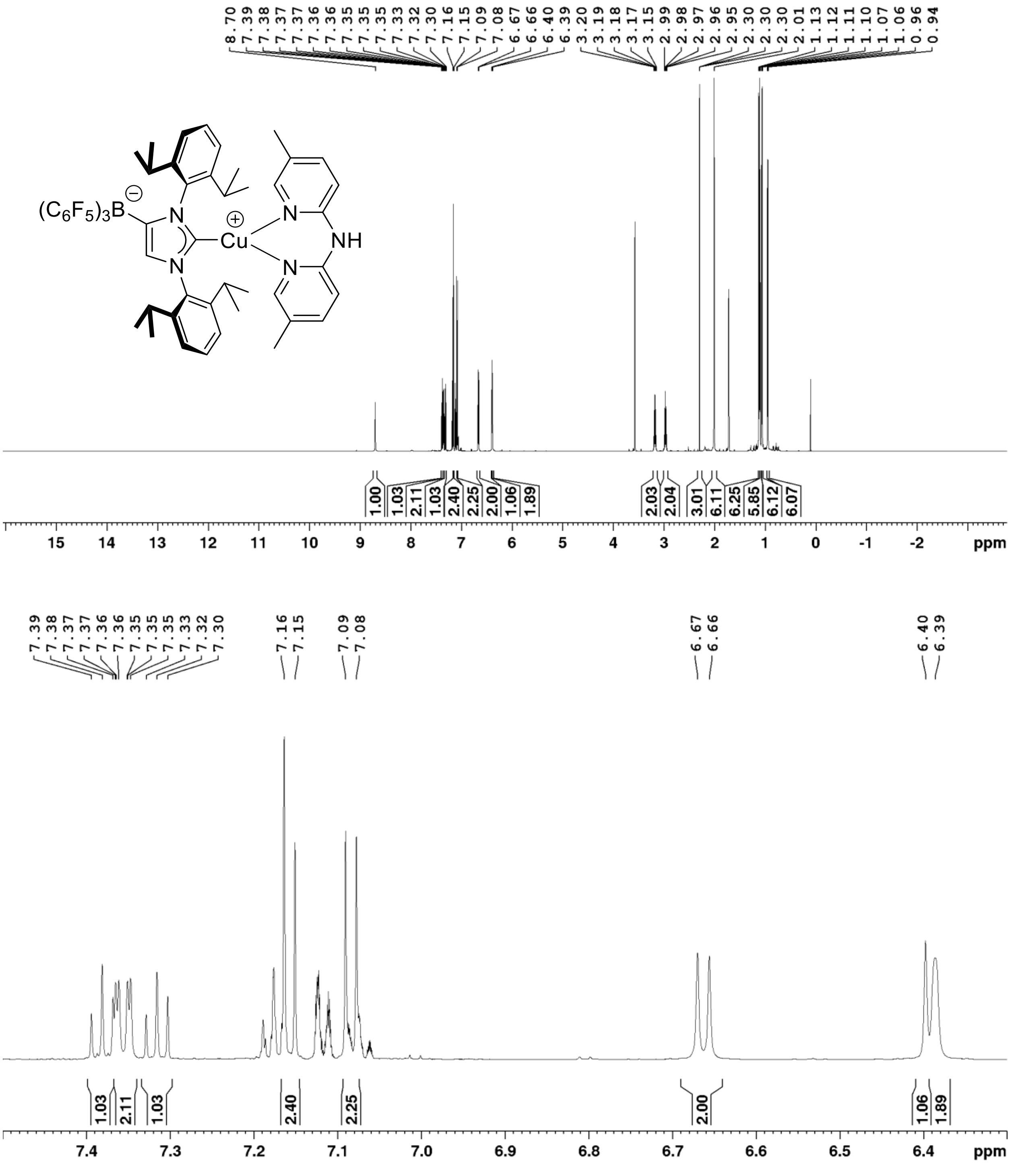

Figure S32. ^{1}H NMR of $B(C_6F_5)_3$-IDipp-Cu-5,5'Me_2Hdpa (**C5**) (top) and enlargement of aromatic region (bottom) in THF-d_8.

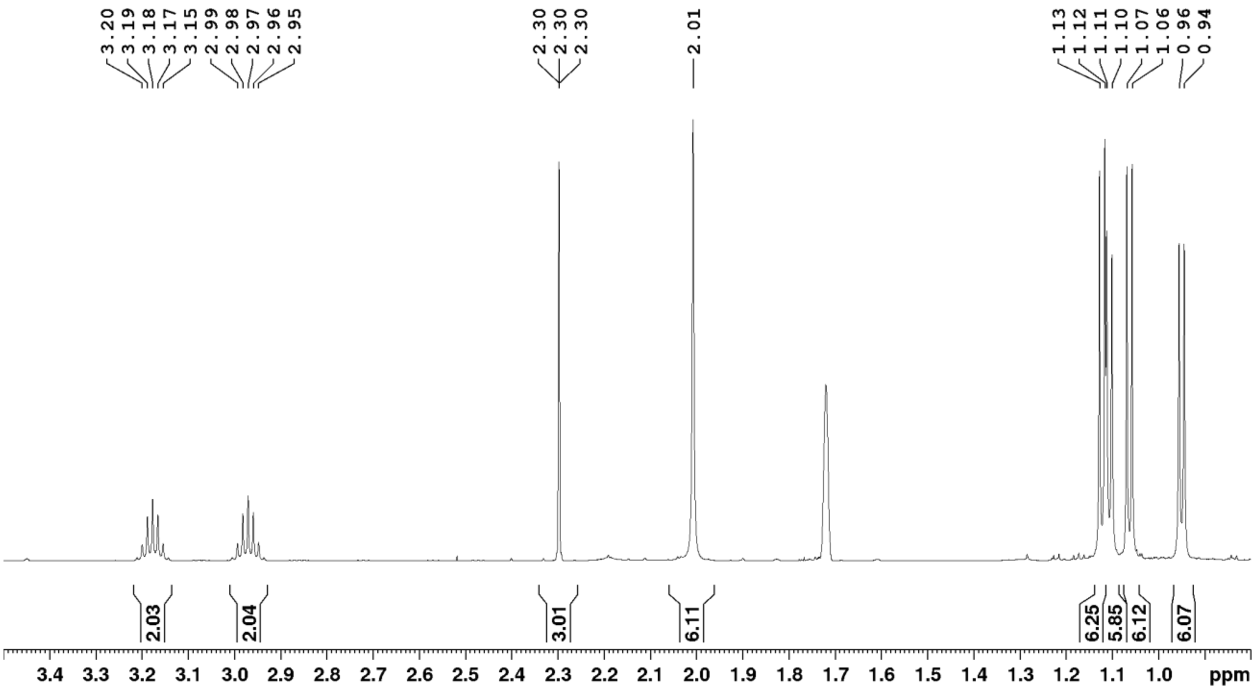

Figure S33. ^{1}H NMR of B(C_6F_5)$_3$-IDipp-Cu-5,5'Me_2Hdpa (**C5**) (aliphatic region enlarged) in THF-d_8.

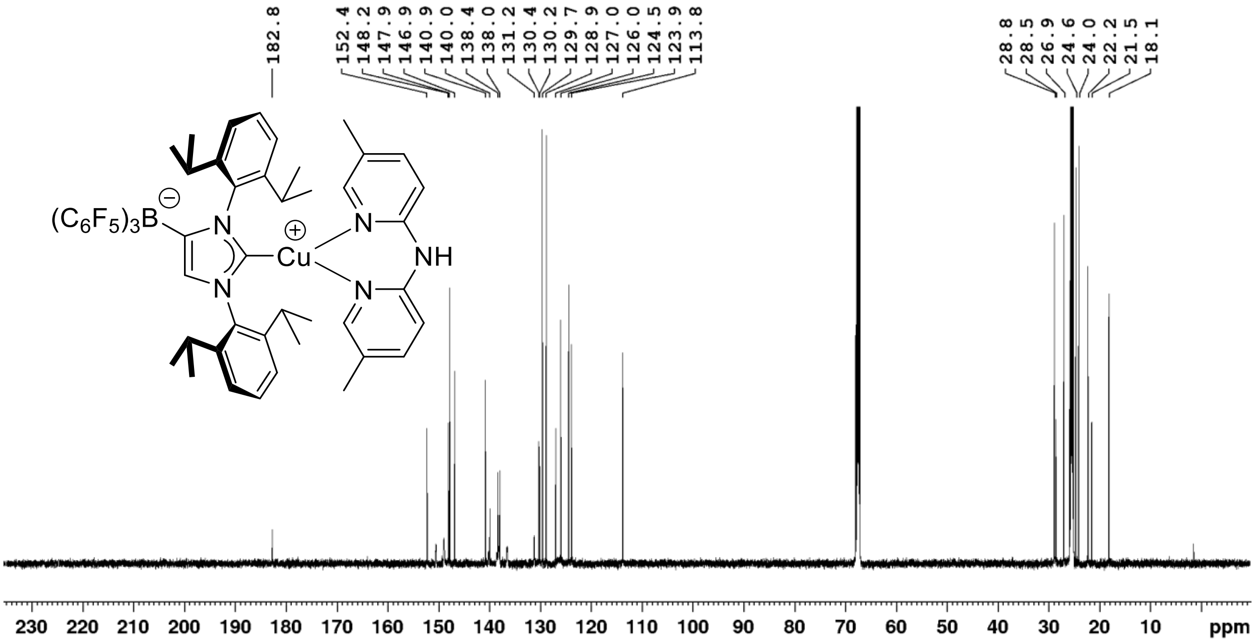

Figure S34. ^{13}C{^{1}H} NMR of B(C_6F_5)$_3$-IDipp-Cu-5,5'Me_2Hdpa (**C5**) in THF-d_8.

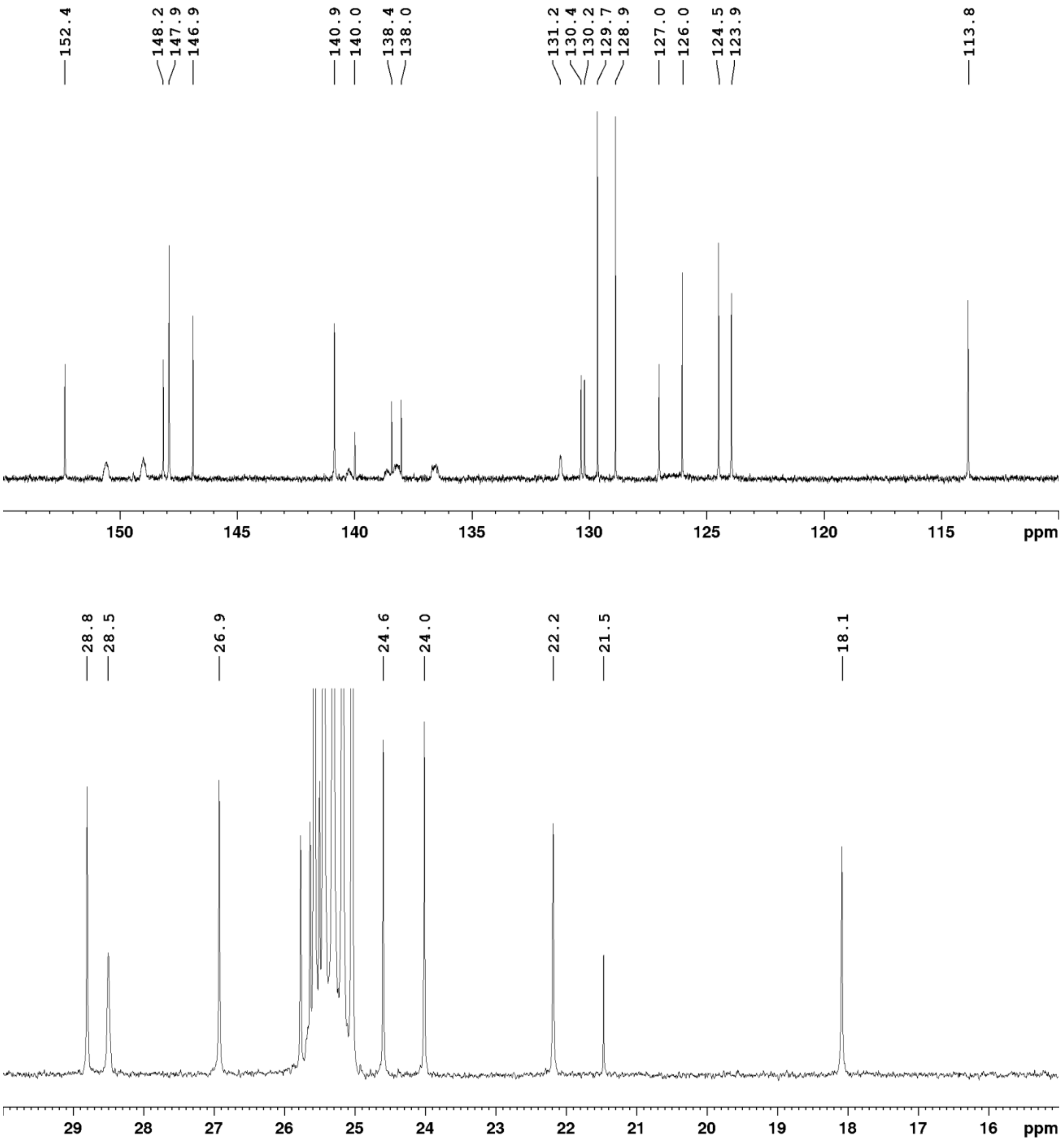

Figure S35. $^{13}C\{^{1}H\}$ NMR of $B(C_6F_5)_3$-IDipp-Cu-5,5'Me_2Hdpa (**C5**) with enlargement of aromatic region (top) and aliphatic region (bottom) in THF-d_8.

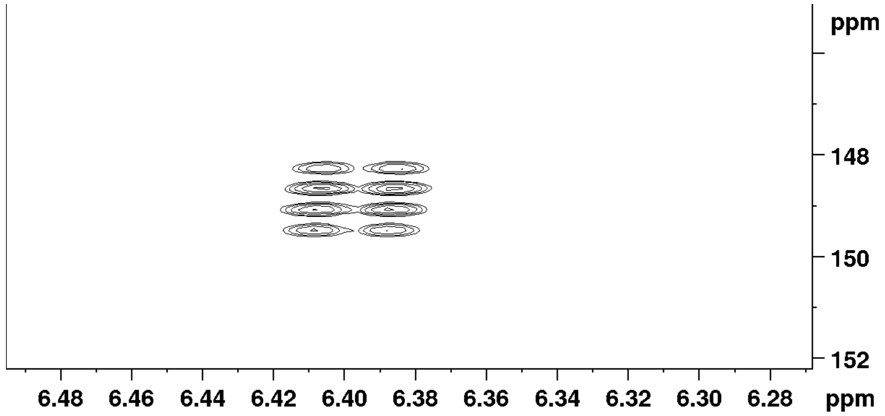

Figure S36. ^{1}H,^{13}C-HMBC of $B(C_6F_5)_3$-IDipp-Cu-5,5'Me_2Hdpa (**C5**) enlarged for identifying the signal for the boron bound carbon atom in the backbone in THF-d_8.

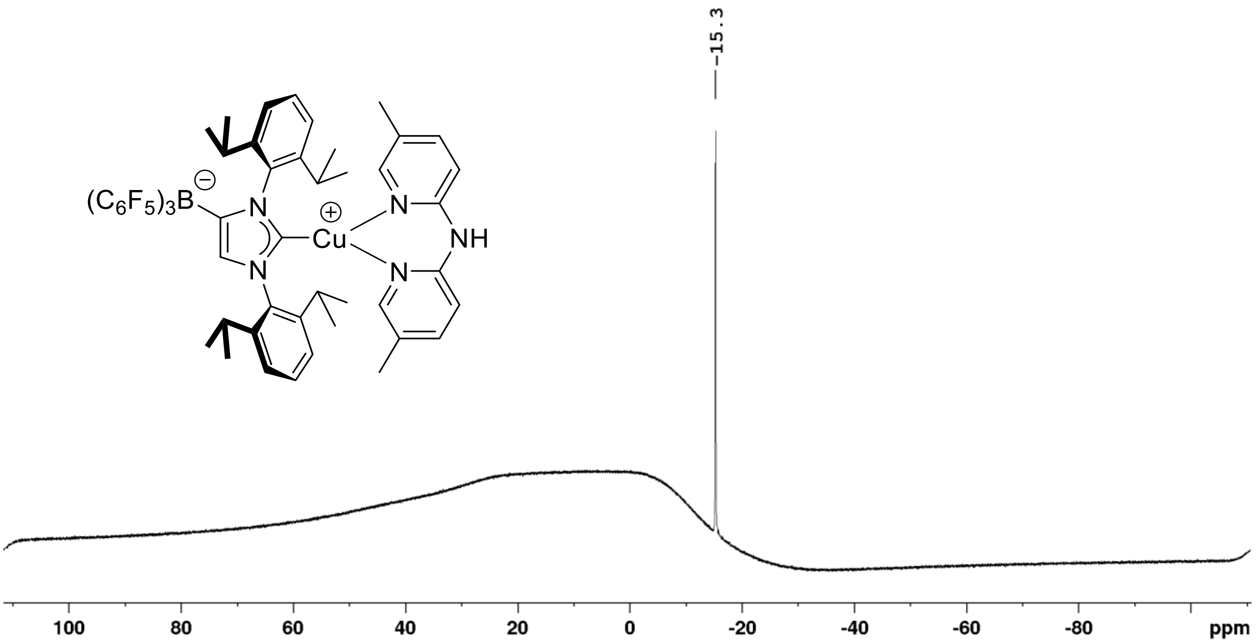

Figure S37. $^{11}B\{^{1}H\}$ NMR of $B(C_6F_5)_3$-IDipp-Cu-5,5'Me_2Hdpa (**C5**) in THF-d_8.

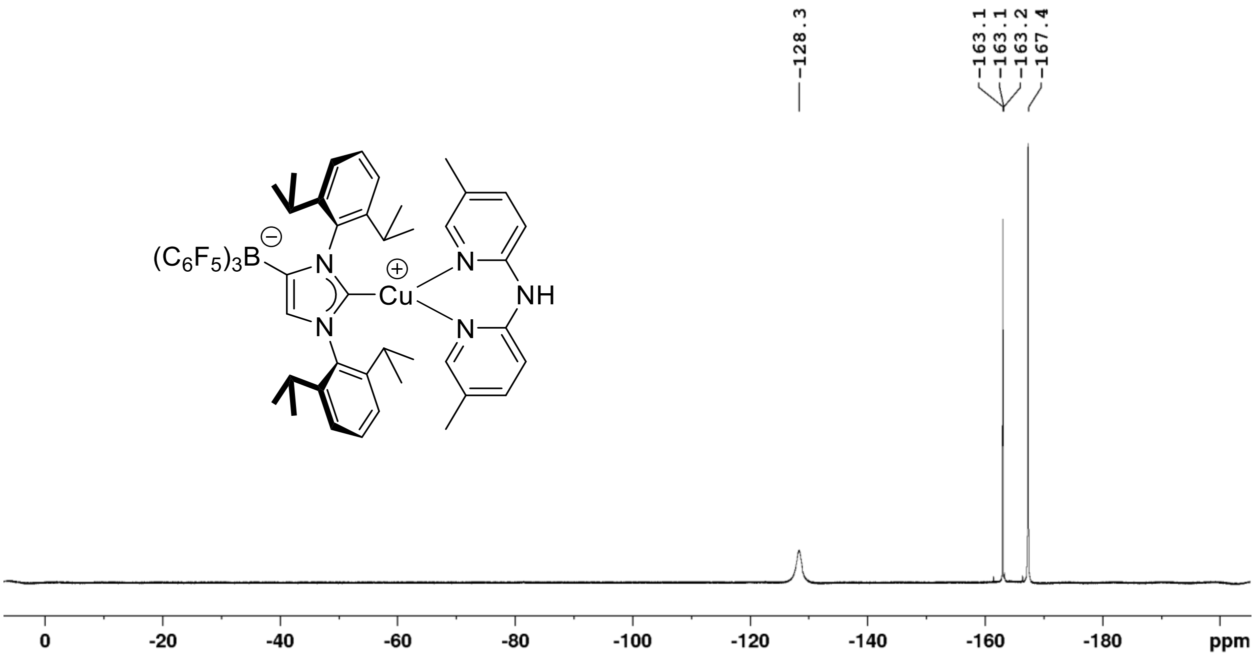

Figure S38. $^{19}F\{^{1}H\}$ NMR of $B(C_6F_5)_3$-IDipp-Cu-5,5'Me_2Hdpa (**C5**) in THF-d_8.

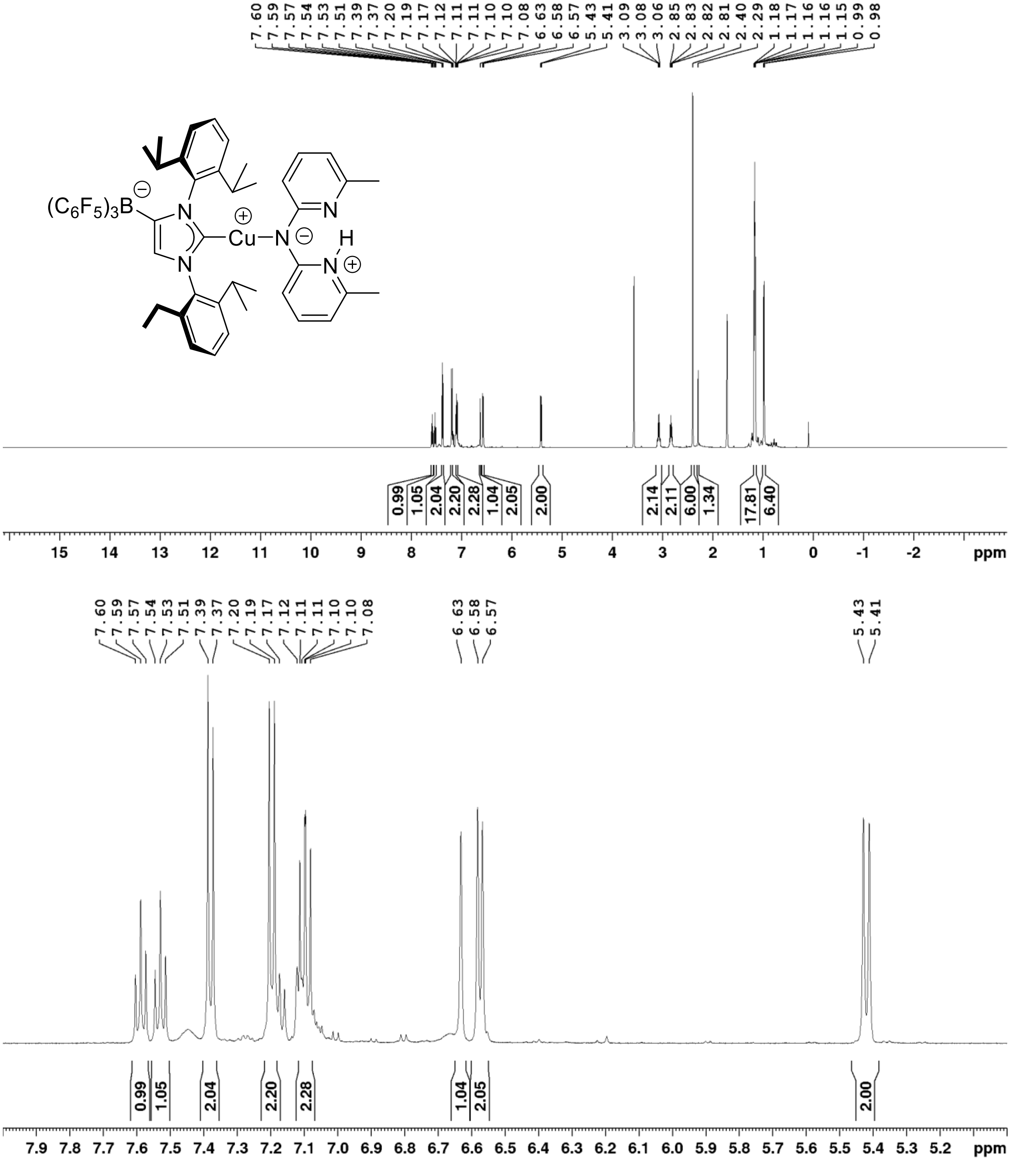

Figure S39. ^{1}H NMR of B(C_6F_5)$_3$-IDipp-Cu-6,6'Me_2Hdpa (**C6**) (top) with enlargement of aromatic region (bottom) in THF-d_8.

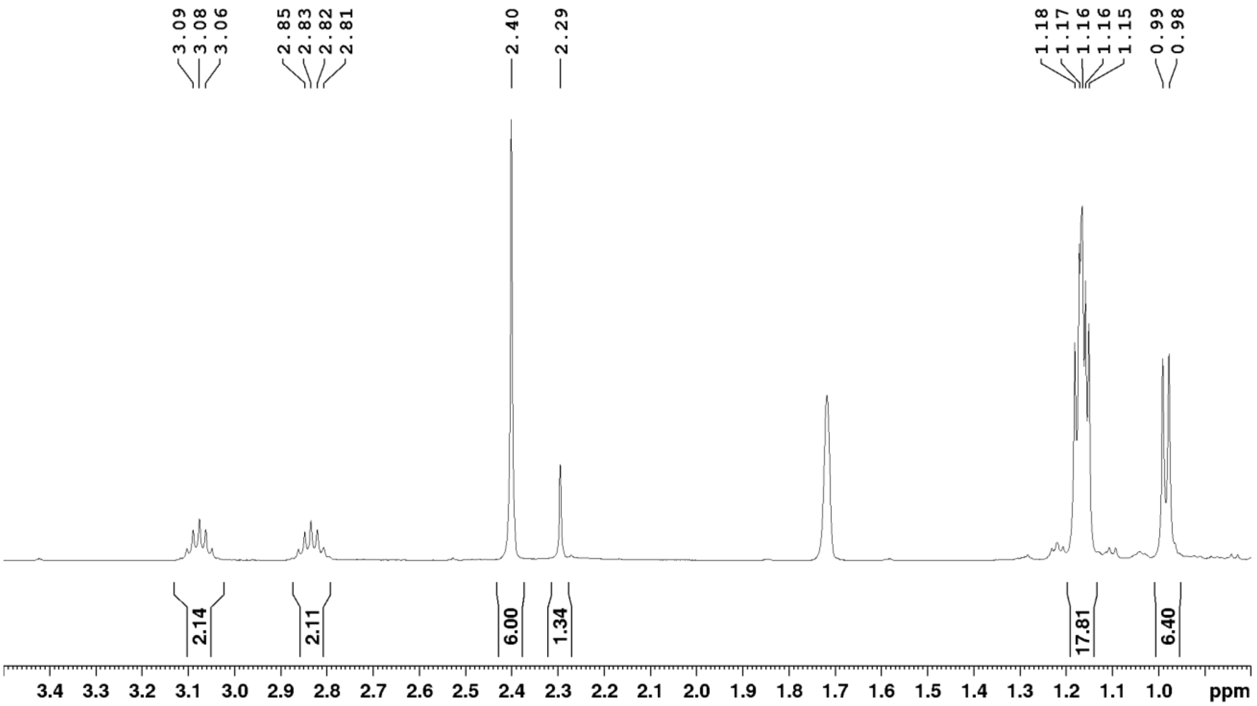

Figure S40. ^{1}H NMR of $B(C_6F_5)_3$-IDipp-Cu-6,6'Me_2Hdpa (**C6**) (aliphatic region enlarged) in THF-d_8.

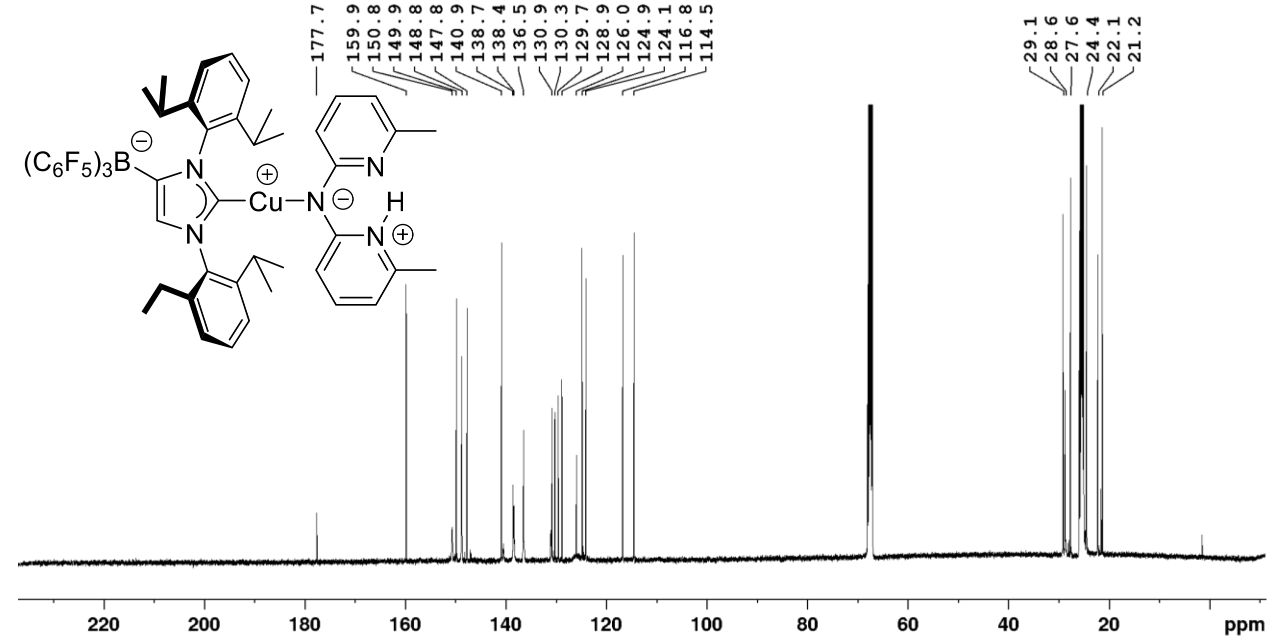

Figure S41. $^{13}C\{^1H\}$ NMR of $B(C_6F_5)_3$-IDipp-Cu-6,6'Me_2Hdpa (**C6**) in THF-d_8.

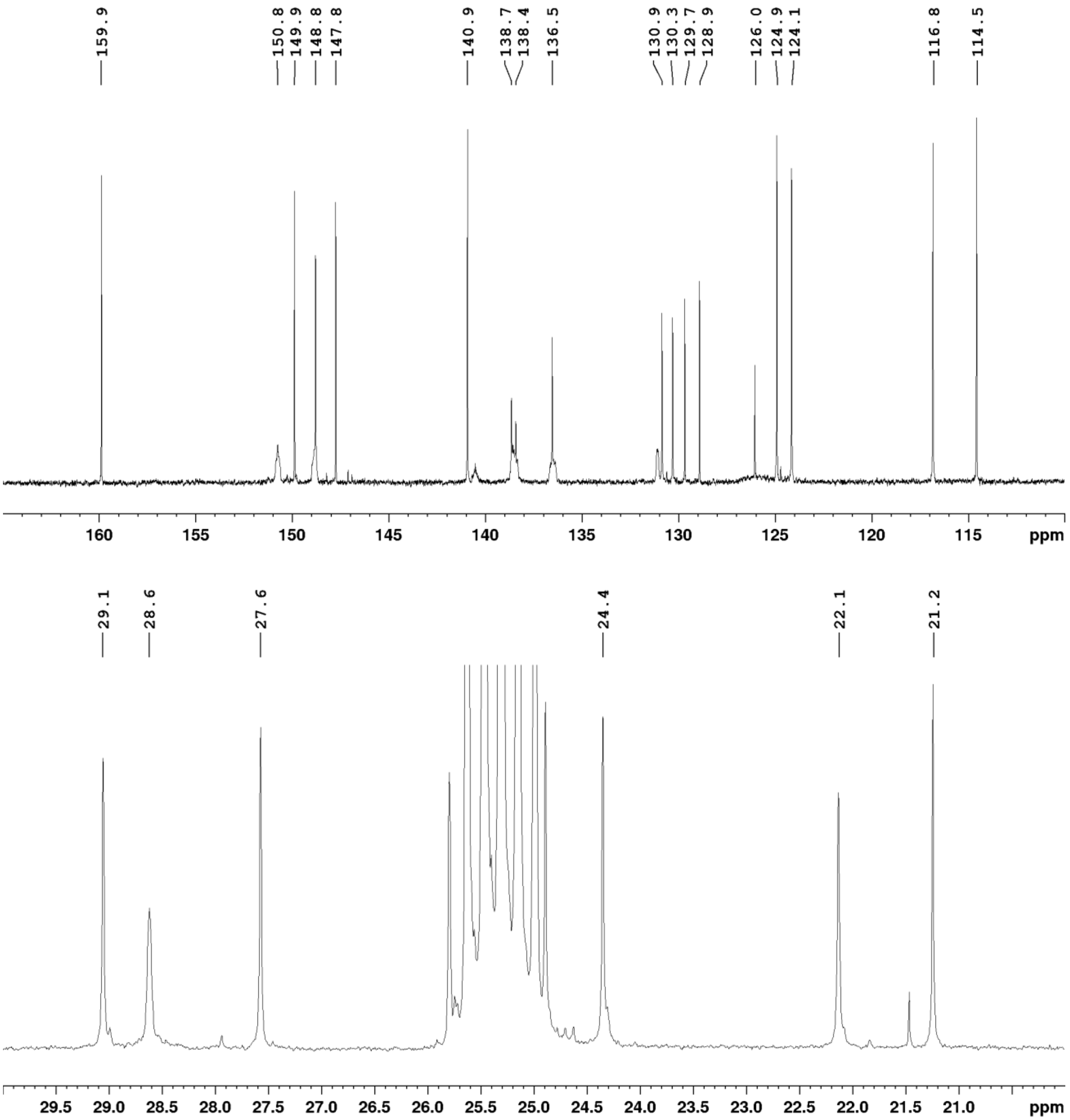

Figure S42. $^{13}C\{^{1}H\}$ NMR of $B(C_6F_5)_3$-IDipp-Cu-6,6'Me_2Hdpa (**C6**) with enlargement of aromatic region (top) and aliphatic region (bottom) in THF-d_8.

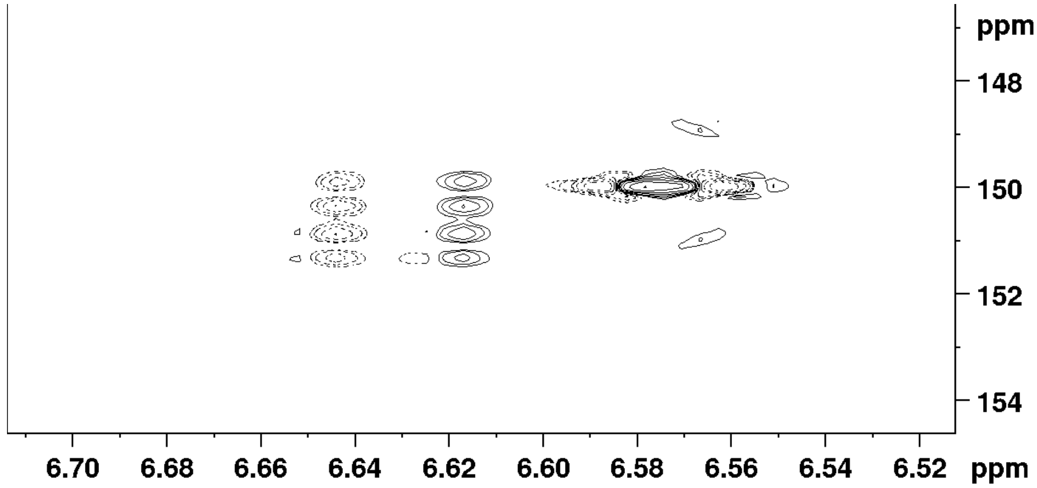

Figure S43. $^{1}H,^{13}C$-HMBC of $B(C_6F_5)_3$-IDipp-Cu-6,6'Me_2Hdpa (**C6**) enlarged for identifying the signal for the boron bound carbon atom in the backbone in THF-d_8.

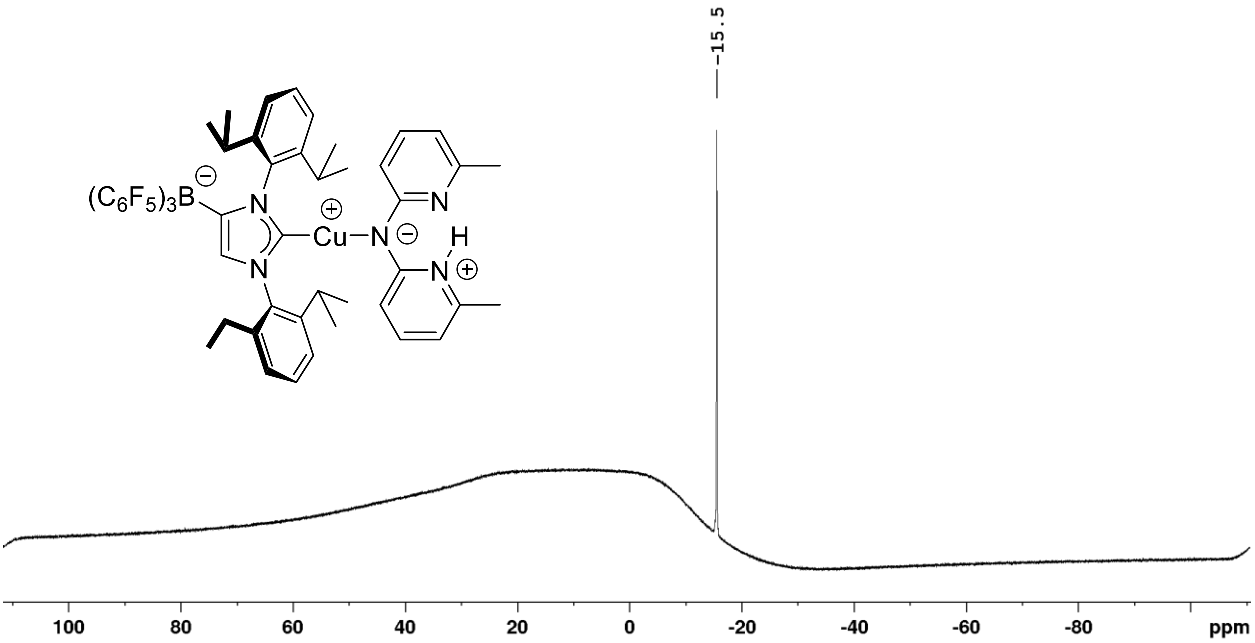

Figure S44. $^{11}B\{^{1}H\}$ NMR of $B(C_6F_5)_3$-IDipp-Cu-6,6'Me_2Hdpa (**C6**) in THF-d_8.

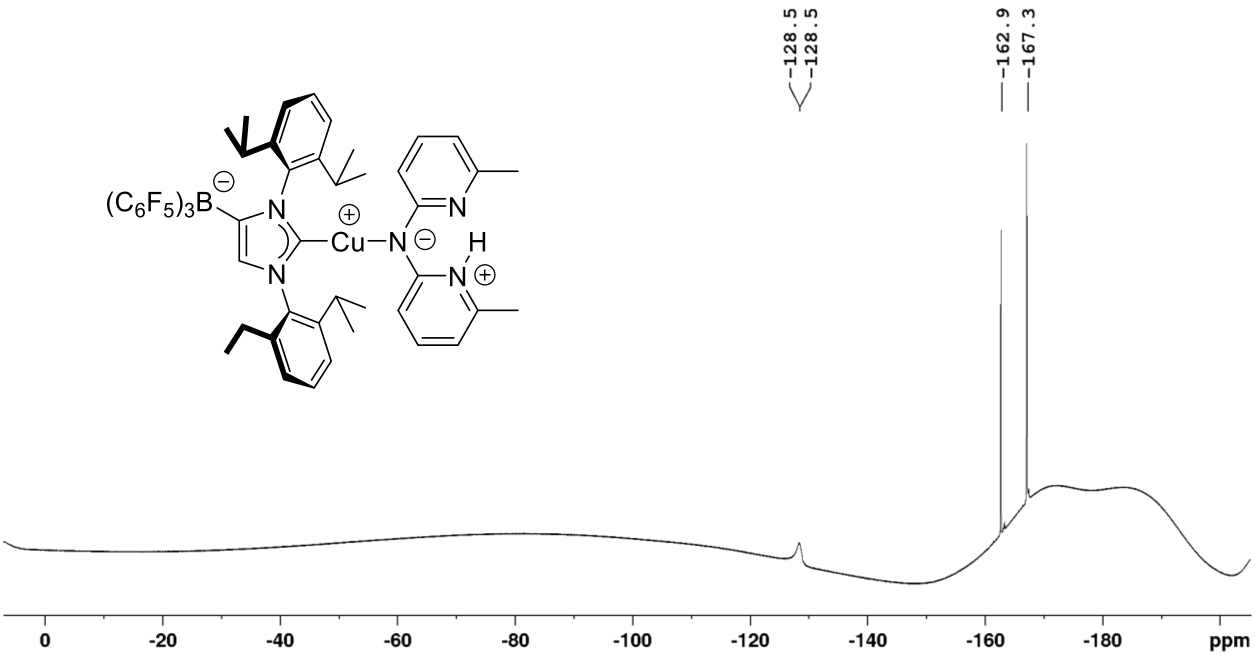

Figure S45. $^{19}F\{^{1}H\}$ NMR of $B(C_6F_5)_3$-IDipp-Cu-6,6'Me_2Hdpa (**C6**) in THF-d_8.

4 Crystallographic Data

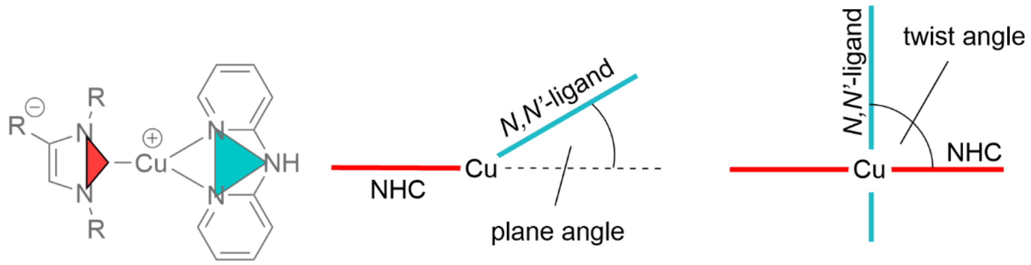

Figure S46. Definition of the plane angle (left) and twist angle (right).

Table S1. Crystallographic details for **C2-I**. The crystal structure is depicted with disordered co-crystalized solvent molecules.

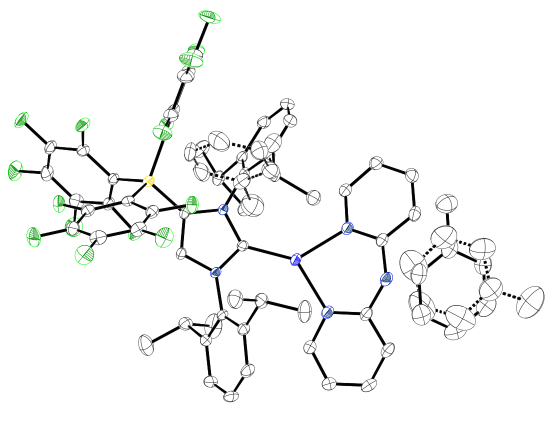	CCDC	2432855
	Temperature	100(2) K
	Wavelength	0.71073 Å
	Crystal system	triclinic
	Space group (No.)	$P\overline{1}$ (2)
	Unit Cell dimensions	
	a = 13.5723(5) Å	*α* = 69.238(4)°
	b = 14.5915(5) Å	*β* = 70.716(3)°
	c = 16.9800(7) Å	*γ* = 76.745(3)°
	Volume	2943.8(2) $Å^3$
	Z	2
Empirical formula	$C_{65.50}H_{56}BCuF_{15}N_5$	
Moiety formula	$C_{55}H_{44}BCuF_{15}N_5$, 1.5 C_7H_8	
Formula weight	1272.50	
Density (calculated)	1.436 g cm^{-3}	
Absorption coefficient	0.465 mm^{-1}	
F(000)	1306	
Crystal habitus	irregular (colourless)	
Crystal size	0.350 × 0.120 × 0.020 mm^3	
Θ range	2.294 to 31.506°	
Index ranges	$-19 \leq h \leq 19$, $-21 \leq k \leq 21$, $-24 \leq l \leq 24$	
Reflections collected	189263	
Independent reflections	19584 [R_{int} = 0.0698]	
Completeness to *Θ* = 25.242°	99.9 %	
Absorption correction	gaussian	
Max. and min. transmission	1.000 and 0.504	
Data / restraints / parameters	19584 / 45 / 871	
Goodness-of-fit on F^2	1.026	
Final *R* indices [$I > 2\sigma(I)$]	R_1 = 0.0498, wR_2 = 0.1143	
R indices (all data)	R_1 = 0.0734, wR_2 = 0.1232	
Largest diff. peak and hole	0.87 and -0.60 e $Å^{-3}$	
Crystallization Details	from toluene / *n*-pentane, rt.	
Measurement and Refinement Details: The toluene molecules were found disordered. One of those was found disordered over an inversion center. AFIX 66 restraints were applied to refine the toluene as a rigid group.		

Table S2. Crystallographic details for **C2-II**. The crystal structure is depicted with disordered co-crystalized solvent molecules.

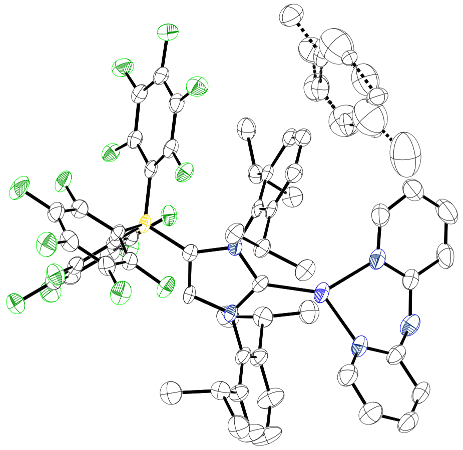	CCDC	2432856
	Temperature	100(2) K
	Wavelength	1.54184 Å
	Crystal system	monoclinic
	Space group (No.)	$P2_1/n$ (14)
	Unit Cell dimensions	
	a = 10.3730(8) Å	*α* = 90°
	b = 24.344(2) Å	*β* = 101.823(8)°
	c = 22.0945(17) Å	*γ* = 90°
	Volume	5460.9(8) $Å^3$
	Z	4
Empirical formula	$C_{62}H_{52}BCuF_{15}N_5$	
Moiety formula	$C_{55}H_{44}BCuF_{15}N_5$, C_7H_8	
Formula weight	1226.43	
Density (calculated)	1.492 g cm^{-3}	
Absorption coefficient	1.425 mm^{-1}	
F(000)	2512	
Crystal habitus	irregular (colourless)	
Crystal size	0.090 × 0.030 × 0.020 mm^3	
Θ range	2.733 to 54.240°	
Index ranges	$-10 \le h \le 10$, $-25 \le k \le 25$, $-21 \le l \le 23$	
Reflections collected	43661	
Independent reflections	6670 [R_{int} = 0.1344]	
Completeness to *Θ* = 54.240°	100.0 %	
Absorption correction	multi-scan	
Max. and min. transmission	1.00000 and 0.53531	
Data / restraints / parameters	6670 / 88 / 818	
Goodness-of-fit on F^2	0.983	
Final *R* indices [$I > 2\sigma(I)$]	R_1 = 0.0538, wR_2 = 0.1236	
R indices (all data)	R_1 = 0.0922, wR_2 = 0.1411	
Largest diff. peak and hole	0.71 and -0.49 e $Å^{-3}$	
Crystallization Details	from toluene / *n*-pentane, rt.	
Measurement and Refinement Details: The toluene molecule was found disordered and refined accordingly with restraints as a rigid group. This structure is similar to *CCDC 2432855* which contains 0.5 more toluene per asymmetric unit. Only very small crystals showing this structure were found besides the ones of *CCDC 2432855*. The structure suffers from poor data quality and only demonstrates that a different structure of this compound can be crystalized. Any geometric parameters of this compound should be derived from *CCDC 2432855*.		

Table S3. Crystallographic details for **C2-III**. The crystal structure is depicted with disordered co-crystalized solvent molecules.

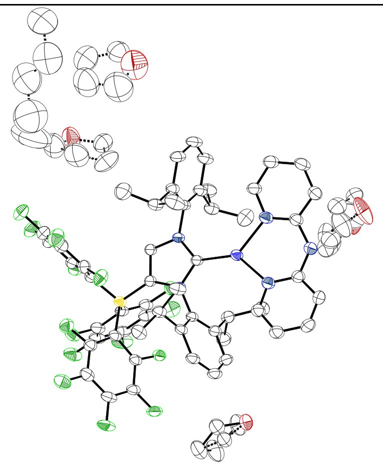

CCDC	2432857
Temperature	100(2) K
Wavelength	1.54184 Å
Crystal system	triclinic
Space group (No.)	$P\overline{1}$ (2)
Unit Cell dimensions	
a = 14.8033(5) Å	*α* = 66.070(5)°
b = 15.8027(9) Å	*β* = 85.569(3)°
c = 17.0651(7) Å	*γ* = 62.764(5)°
Volume	3215.4(3) Å^3
Z	2
Empirical formula	$C_{69.50}H_{74}BCuF_{15}N_5O_3$
Moiety formula	$C_{55}H_{44}BCuF_{15}N_5$, 3 C_4H_8O, 0.5 C_5H_{12}
Formula weight	1386.68
Density (calculated)	1.432 g cm^{-3}
Absorption coefficient	1.310 mm^{-1}
F(000)	1438
Crystal habitus	irregular (colourless)
Crystal size	0.300 × 0.090 × 0.050 mm^3
Θ range	3.388 to 76.841°
Index ranges	$-18 \le h \le 18$, $-19 \le k \le 19$, $-21 \le l \le 21$
Reflections collected	86158
Independent reflections	13105 [R_{int} = 0.0450]
Completeness to *Θ* = 67.684°	99.6 %
Absorption correction	gaussian
Max. and min. transmission	1.000 and 0.459
Data / restraints / parameters	13105 / 286 / 988
Goodness-of-fit on F^2	1.050
Final *R* indices [$I > 2\sigma(I)$]	R_1 = 0.0489, wR_2 = 0.1361
R indices (all data)	R_1 = 0.0551, wR_2 = 0.1407
Largest diff. peak and hole	0.61 and -0.66 e Å^{-3}
Crystallization Details	from THF / *n*-pentane, rt.
Measurement and Refinement Details: The hydrogen atom H4 on N4 was refined freely. Two molecules of THF and one molecule of n-hexane were found disordered on an inversion center and refined accordingly with SADI restraints on chemically equivalent distances.	

Table S4. Crystallographic details for **C3**. The crystal structure is depicted with co-crystalized solvent molecules.

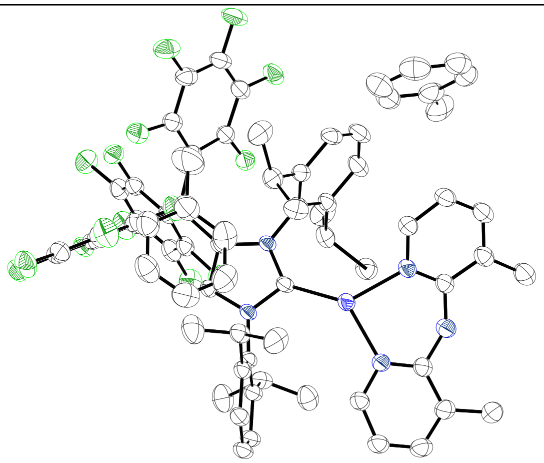	CCDC	2432858
	Temperature	100(2) K
	Wavelength	1.54184 Å
	Crystal system	monoclinic
	Space group (No.)	$P2_1/n$ (14)
	Unit Cell dimensions	
	a = 14.1751(2) Å	*α* = 90°
	b = 23.6230(4) Å	*β* = 107.065(2)°
	c = 19.5332(3) Å	*γ* = 90°
	Volume	6252.88(18) $Å^3$
	Z	4
Empirical formula	$C_{71}H_{64}BCuF_{15}N_5$	
Moiety formula	$C_{57}H_{48}BCuF_{15}N_5$, 2 C_7H_8	
Formula weight	1346.62	
Density (calculated)	1.430 g cm^{-3}	
Absorption coefficient	1.297 mm^{-1}	
F(000)	2776	
Crystal habitus	plate (colourless)	
Crystal size	0.390 × 0.190 × 0.030 mm^3	
Θ range	3.017 to 80.433°	
Index ranges	$-18 \le h \le 18$, $-30 \le k \le 28$, $-24 \le l \le 24$	
Reflections collected	136700	
Independent reflections	13510 [R_{int} = 0.0564]	
Completeness to *Θ* = 67.684°	100.0 %	
Absorption correction	gaussian	
Max. and min. transmission	1.000 and 0.324	
Data / restraints / parameters	13510 / 0 / 854	
Goodness-of-fit on F^2	1.064	
Final *R* indices [$I > 2\sigma(I)$]	R_1 = 0.0479, wR_2 = 0.1359	
R indices (all data)	R_1 = 0.0535, wR_2 = 0.1409	
Largest diff. peak and hole	0.78 and -0.46 e $Å^{-3}$	
Crystallization Details	from toluene / *n*-hexane, -40 °C	
Measurement and Refinement Details: The hydrogen atom H4 on N4 was refined freely.		

Table S5. Crystallographic details for **C4**. The crystal structure is depicted with co-crystalized solvent molecules.

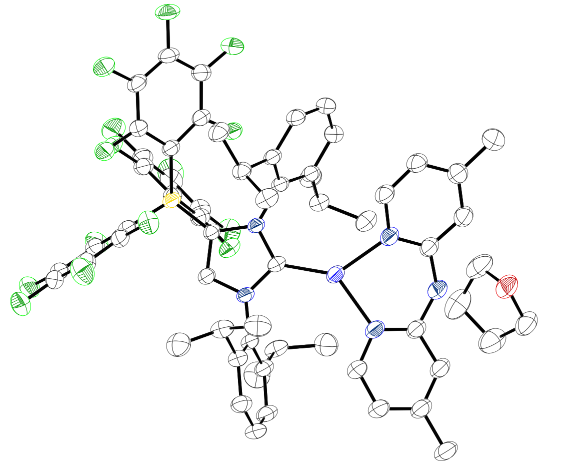

CCDC	2432859
Temperature	100(2) K
Wavelength	1.54184 Å
Crystal system	triclinic
Space group (No.)	$P\overline{1}$ (2)
Unit Cell dimensions	
a = 11.9750(3) Å	α = 72.855(2)°
b = 15.9736(4) Å	β = 77.236(2)°
c = 19.2185(5) Å	γ = 68.704(2)°
Volume	3246.08(15) $Å^3$
Z	2
Empirical formula	$C_{70}H_{76}BCuF_{15}N_5O_2$
Moiety formula	$C_{57}H_{48}BCuF_{15}N_5$, C_4H_8O, [C_4H_8O, C_5H_{12}]
Formula weight	1378.70
Density (calculated)	1.411 g cm^{-3}
Absorption coefficient	1.281 mm^{-1}
F(000)	1432
Crystal habitus	irregular (colourless)
Crystal size	0.390 × 0.240 × 0.140 mm^3
Θ range	2.426 to 80.438°
Index ranges	$-15 \leq h \leq 15$, $-20 \leq k \leq 20$, $-24 \leq l \leq 24$
Reflections collected	140282
Independent reflections	13974 [R_{int} = 0.0409]
Completeness to Θ = 67.684°	99.9 %
Absorption correction	gaussian
Max. and min. transmission	1.000 and 0.284
Data / restraints / parameters	13974 / 0 / 767
Goodness-of-fit on F^2	1.030
Final R indices [$I > 2\sigma(I)$]	R_1 = 0.0400, wR_2 = 0.1091
R indices (all data)	R_1 = 0.0409, wR_2 = 0.1097
Largest diff. peak and hole	0.38 and -0.55 e $Å^{-3}$
Crystallization Details	from THF / n-pentane, -40 °C
Measurement and Refinement Details: One molecule of THF and one molecule of n-pentane per asymmetric unit have been identified but could not be refined satisfactorily. Therefore, a solvent mask, the Olex2 implementation of the program SQUEEZE[32] has been used to mathematically remove the electron density of these molecules. The sum formula and derived parameters are based on two molecules of THF and one n-pentane per asymmetric unit. The solvent mask was calculated and 153 electrons were found in a volume of 672$Å^3$ in 1 void per unit cell.	

Table S6. Crystallographic details for **C5**. The crystal structure is depicted with disordered co-crystalized solvent molecules.

<table>
<tr><td rowspan="12">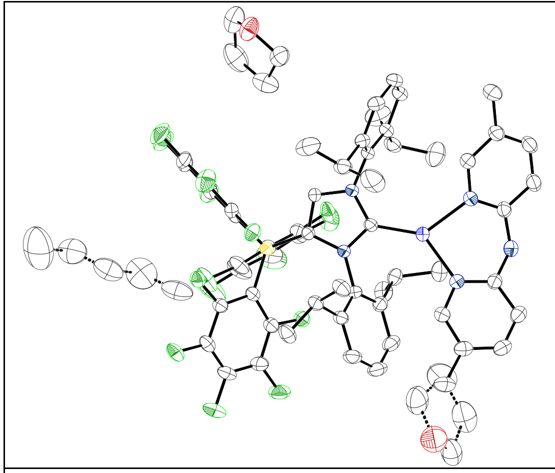</td><td>CCDC</td><td>2432860</td></tr>
<tr><td>Temperature</td><td>100(2) K</td></tr>
<tr><td>Wavelength</td><td>1.54184 Å</td></tr>
<tr><td>Crystal system</td><td>triclinic</td></tr>
<tr><td>Space group (No.)</td><td>$P\overline{1}$ (2)</td></tr>
<tr><td colspan="2">Unit Cell dimensions</td></tr>
<tr><td>a = 13.8187(4) Å</td><td>α = 83.659(2)°</td></tr>
<tr><td>b = 14.0429(4) Å</td><td>β = 85.641(2)°</td></tr>
<tr><td>c = 17.9804(4) Å</td><td>γ = 63.304(3)°</td></tr>
<tr><td>Volume</td><td>3096.81(16) Å^3</td></tr>
<tr><td>Z</td><td>2</td></tr>
<tr><td></td><td></td></tr>
<tr><td>Empirical formula</td><td colspan="2">$C_{65.50}H_{66}BCuF_{15}N_5O_{1.50}$</td></tr>
<tr><td>Moiety formula</td><td colspan="2">$C_{57}H_{48}BCuF_{15}N_5$, 1.5 C_4H_8O, 0.5 C_5H_{12}</td></tr>
<tr><td>Formula weight</td><td colspan="2">1306.58</td></tr>
<tr><td>Density (calculated)</td><td colspan="2">1.401 g cm^{-3}</td></tr>
<tr><td>Absorption coefficient</td><td colspan="2">1.307 mm^{-1}</td></tr>
<tr><td>F(000)</td><td colspan="2">1350</td></tr>
<tr><td>Crystal habitus</td><td colspan="2">needle (colourless)</td></tr>
<tr><td>Crystal size</td><td colspan="2">0.190 × 0.070 × 0.030 mm^3</td></tr>
<tr><td>Θ range</td><td colspan="2">2.474 to 80.262°</td></tr>
<tr><td>Index ranges</td><td colspan="2">$-17 \le h \le 17$, $-17 \le k \le 17$, $-22 \le l \le 20$</td></tr>
<tr><td>Reflections collected</td><td colspan="2">130093</td></tr>
<tr><td>Independent reflections</td><td colspan="2">13258 [R_{int} = 0.0905]</td></tr>
<tr><td>Completeness to Θ = 67.684°</td><td colspan="2">99.9 %</td></tr>
<tr><td>Absorption correction</td><td colspan="2">gaussian</td></tr>
<tr><td>Max. and min. transmission</td><td colspan="2">1.000 and 0.535</td></tr>
<tr><td>Data / restraints / parameters</td><td colspan="2">13258 / 117 / 863</td></tr>
<tr><td>Goodness-of-fit on F^2</td><td colspan="2">1.059</td></tr>
<tr><td>Final R indices [$I > 2\sigma(I)$]</td><td colspan="2">R_1 = 0.0435, wR_2 = 0.1147</td></tr>
<tr><td>R indices (all data)</td><td colspan="2">R_1 = 0.0518, wR_2 = 0.1207</td></tr>
<tr><td>Largest diff. peak and hole</td><td colspan="2">0.46 and -0.51 e Å^{-3}</td></tr>
<tr><td>Crystallization Details</td><td colspan="2">from THF / n-pentane, -40 °C</td></tr>
<tr><td colspan="3">Measurement and Refinement Details: The NH hydrogen atom H5 was refined freely. A molecule of n-pentane and THF per cell were found disordered via an inversion center. The molecules were placed with the FragmentDB plugin in Olex2.[33] SADI restraints have been applied to chemically equivalent C-C distances of the n-pentane molecule.</td></tr>
</table>

Table S7. Crystallographic details for **C6**. The crystal structure is depicted with disordered moieties and co-crystalized solvent molecules.

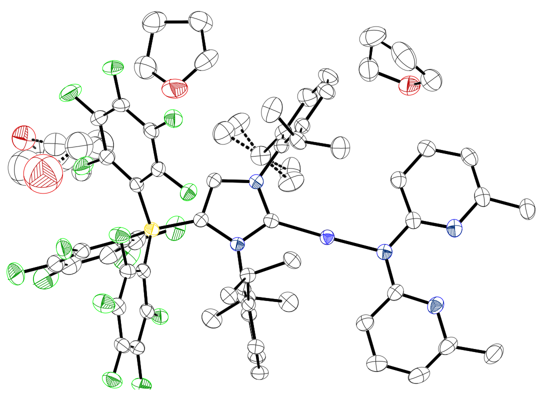	CCDC	2432861
	Temperature	100(2) K
	Wavelength	1.54184 Å
	Crystal system	monoclinic
	Space group (No.)	$P2_1/n$ (14)
	Unit Cell dimensions	
	a = 14.1553(9) Å	*α* = 90°
	b = 32.5490(2) Å	*β* = 103.5090(10)°
	c = 14.37080(10) Å	*γ* = 90°
	Volume	6438.0(4) $Å^3$
	Z	4
Empirical formula	$C_{69}H_{72}BCuF_{15}N_5O_3$	
Moiety formula	$C_{57}H_{48}BCuF_{15}N_5$, $_{3}C_4H_8O$	
Formula weight	1378.66	
Density (calculated)	1.422 g cm^{-3}	
Absorption coefficient	1.305 mm^{-1}	
F(000)	2856	
Crystal habitus	irregular (colourless)	
Crystal size	0.120 × 0.100 × 0.050 mm^3	
Θ range	3.442 to 80.417°	
Index ranges	-18 ≤ *h* ≤ 18, -41 ≤ *k* ≤ 41, -18 ≤ *l* ≤ 17	
Reflections collected	275159	
Independent reflections	13941 [R_{int} = 0.0393]	
Completeness to *Θ* = 67.684°	99.9 %	
Absorption correction	gaussian	
Max. and min. transmission	1.000 and 0.688	
Data / restraints / parameters	13941 / 141 / 925	
Goodness-of-fit on F^2	1.042	
Final *R* indices [$I > 2\sigma(I)$]	R_1 = 0.0449, wR_2 = 0.1216	
R indices (all data)	R_1 = 0.0470, wR_2 = 0.1233	
Largest diff. peak and hole	0.60 and -0.48 e $Å^{-3}$	
Crystallization Details	from THF / *n*-pentane, -40 °C	
Measurement and Refinement Details: The NH hydrogen atoms H4 and H5 were found disordered and refined accordingly. Additionally, an *i*Pr group and a molecule of THF was found disordered. SADI restraints were applied to chemically equivalent distances in the THF moiety.		

5 Electrochemical Data

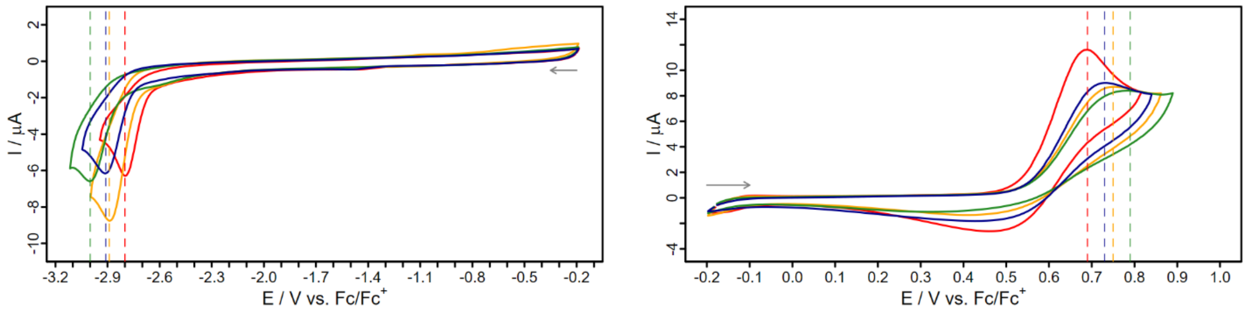

Figure S47. Cyclic voltammograms depicting the reduction (left) and oxidation (right) events of the complexes **C2** (red), **C3** (orange), **C4** (green) and **C5** (blue) in tetrahydrofuran with 0.1 M $[Bu_4N][PF_6]$ as the supporting electrolyte.

6 Density Functional Theory (DFT) Calculations

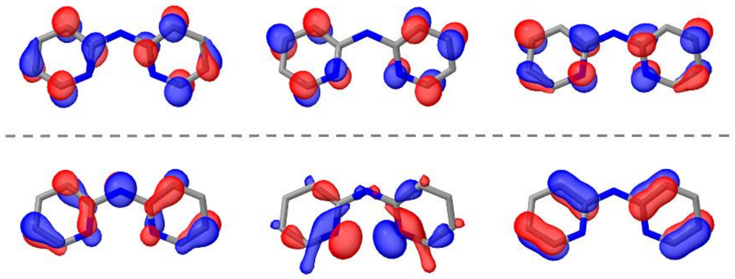

Figure S48. Representation of the LUMO+2, LUMO+1, LUMO, HOMO, HOMO-1 and HOMO-2 (from top left to bottom right) of **L2**. Volumes are depicted with an isosurface value of 0.05 (blue = negative, red = positive).

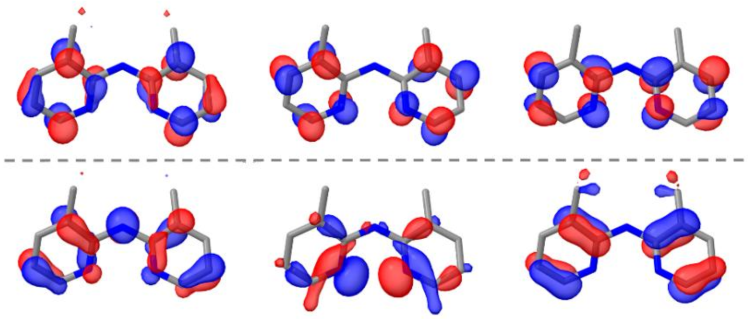

Figure S49. Representation of the LUMO+2, LUMO+1, LUMO, HOMO, HOMO-1 and HOMO-2 (from top left to bottom right) of **L3**. Volumes are depicted with an isosurface value of 0.05 (blue = negative, red = positive).

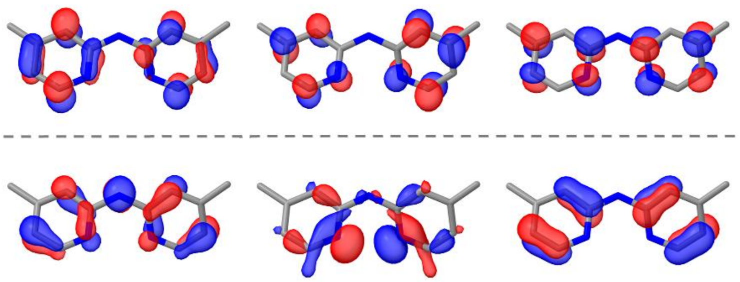

Figure S50. Representation of the LUMO+2, LUMO+1, LUMO, HOMO, HOMO-1 and HOMO-2 (from top left to bottom right) of **L4**. Volumes are depicted with an isosurface value of 0.05 (blue = negative, red = positive).

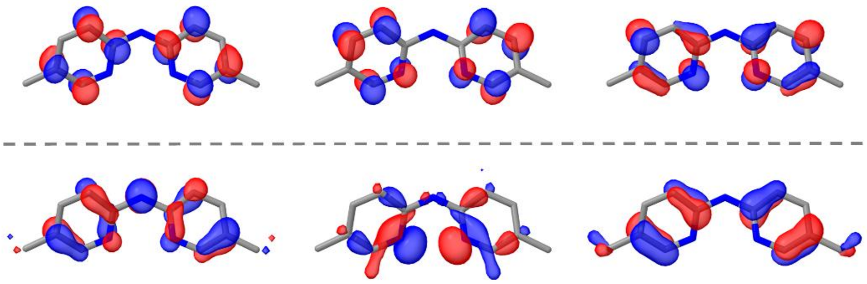

Figure S51. Representation of the LUMO+2, LUMO+1, LUMO, HOMO, HOMO-1 and HOMO-2 (from top left to bottom right) of **L5**. Volumes are depicted with an isosurface value of 0.05 (blue = negative, red = positive).

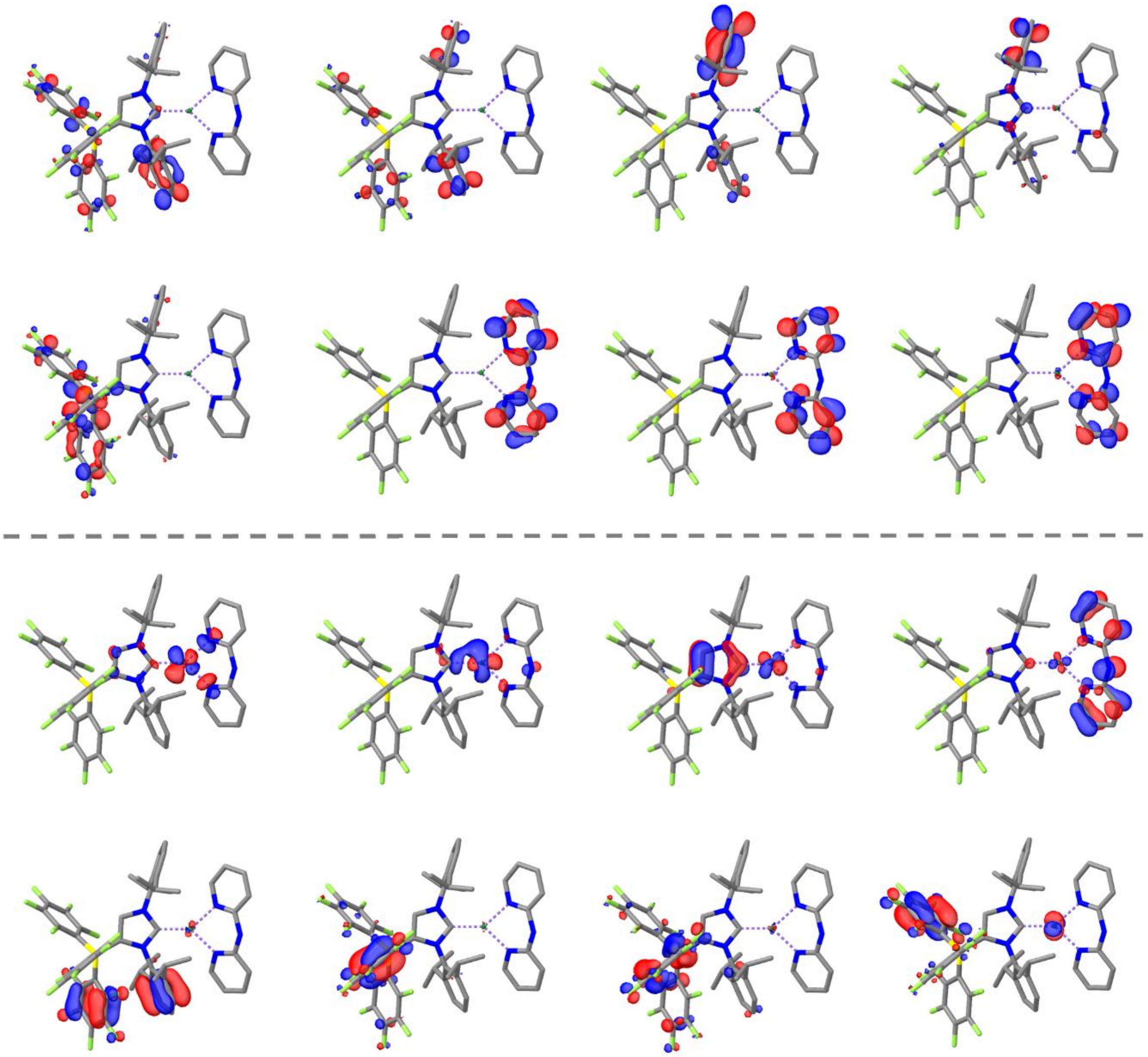

Figure S52. Representation of the LUMO+7, LUMO+6, LUMO+5, LUMO+4, LUMO+3, LUMO+2, LUMO+1, LUMO, HOMO, HOMO-1, HOMO-2, HOMO-3, HOMO-4, HOMO-5, HOMO-6 and HOMO-7 (from top left to bottom right) of **C2**. Volumes are depicted with an isosurface value of 0.05 (blue = negative, red = positive).

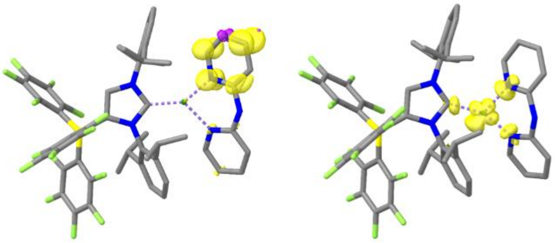

Figure S53. Representation of the spin density of the reduced (left) and oxidized (right) species of **C2** (isosurface value = 0.005).

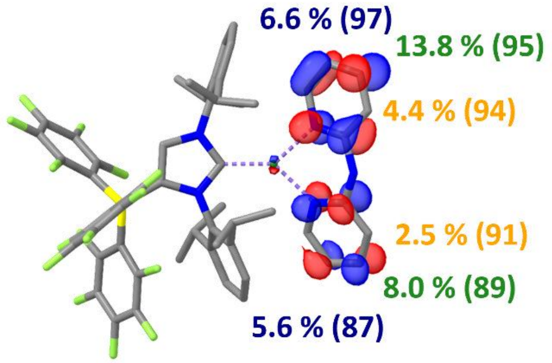

Figure S54. Representation of the LUMO of **C2** with the respective contributions of the atomic orbitals of the relevant carbon atoms obtained from Hirshfeld analysis.

Table S8. Results of the orbital composition analysis for the LUMO of **C2**.

Atom	Contribution / %	Atom	Contribution / %	Atom	Contribution / %
1 (Cu)	1.790	42 (C)	0.003	83 (H)	0.006
2 (F)	0.003	43 (H)	0.002	84 (H)	0.069
3 (F)	0.001	44 (H)	0.000	85 (C)	2.818
4 (F)	0.000	45 (H)	0.002	86 (H)	0.261
5 (F)	0.000	46 (C)	0.012	87 (C)	5.568
6 (F)	0.000	47 (H)	0.004	88 (H)	0.713
7 (F)	0.001	48 (C)	0.003	89 (C)	8.003
8 (F)	0.000	49 (H)	0.001	90 (H)	1.135
9 (F)	0.000	50 (H)	0.001	91 (C)	2.527
10 (F)	0.000	51 (H)	0.001	92 (C)	7.531
11 (F)	0.000	52 (C)	0.058	93 (C)	9.545
12 (F)	0.000	53 (H)	0.045	94 (C)	4.437
13 (F)	0.000	54 (H)	0.008	95 (C)	13.816
14 (F)	0.000	55 (H)	0.098	96 (H)	1.980
15 (F)	0.000	56 (C)	0.107	97 (C)	6.645
16 (F)	0.002	57 (C)	0.254	98 (H)	0.813
17 (N)	0.059	58 (C)	0.099	99 (C)	5.631
18 (N)	0.053	59 (H)	0.018	100 (H)	0.586
19 (N)	8.065	60 (C)	0.173	101 (H)	0.190
20 (N)	2.424	61 (H)	0.022	102 (C)	0.001
21 (H)	0.300	62 (C)	0.214	103 (C)	0.000
22 (N)	12.022	63 (H)	0.051	104 (C)	0.000
23 (C)	0.122	64 (C)	0.172	105 (H)	0.372
24 (C)	0.037	65 (C)	0.032	106 (C)	0.001
25 (C)	0.022	66 (H)	0.004	107 (C)	0.001
26 (H)	0.003	67 (C)	0.029	108 (B)	0.007
27 (C)	0.121	68 (H)	0.017	109 (C)	0.002
28 (C)	0.035	69 (H)	0.004	110 (C)	0.003
29 (C)	0.111	70 (H)	0.020	111 (C)	0.003
30 (H)	0.011	71 (C)	0.015	112 (C)	0.002
31 (C)	0.121	72 (H)	0.004	113 (C)	0.001
32 (H)	0.029	73 (H)	0.003	114 (C)	0.002
33 (C)	0.071	74 (H)	0.001	115 (C)	0.003
34 (H)	0.012	75 (C)	0.032	116 (C)	0.001
35 (C)	0.046	76 (H)	0.008	117 (C)	0.000
36 (C)	0.005	77 (C)	0.009	118 (C)	0.000
37 (H)	0.001	78 (H)	0.002	119 (C)	0.000
38 (C)	0.021	79 (H)	0.002	120 (C)	0.001
39 (H)	0.021	80 (H)	0.002	121 (C)	0.001
40 (H)	0.004	81 (C)	0.086		
41 (H)	0.029	82 (H)	0.162	Sum	100.004898

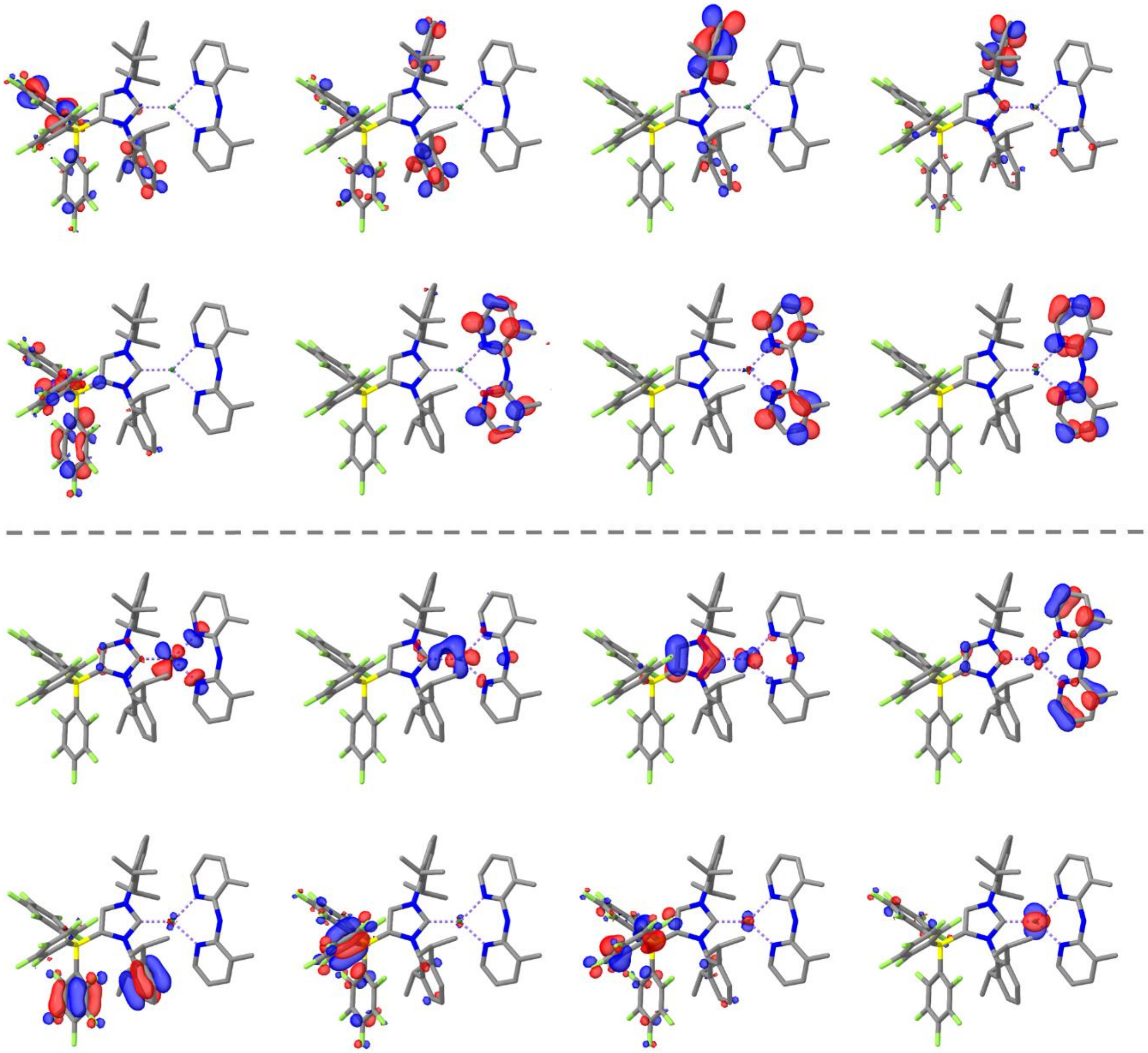

Figure S55. Representation of the LUMO+7, LUMO+6, LUMO+5, LUMO+4, LUMO+3, LUMO+2, LUMO+1, LUMO, HOMO, HOMO-1, HOMO-2, HOMO-3, HOMO-4, HOMO-5, HOMO-6 and HOMO-7 (from top left to bottom right) of **C3**. Volumes are depicted with an isosurface value of 0.05 (blue = negative, red = positive).

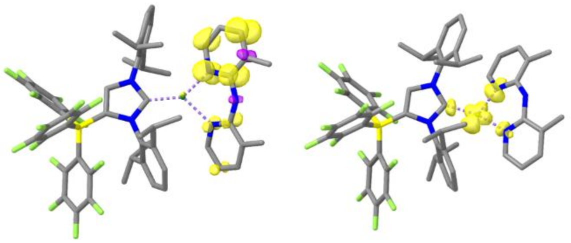

Figure S56. Representation of the spin density of the reduced (left) and oxidized (right) species of **C3** (isosurface value = 0.005).

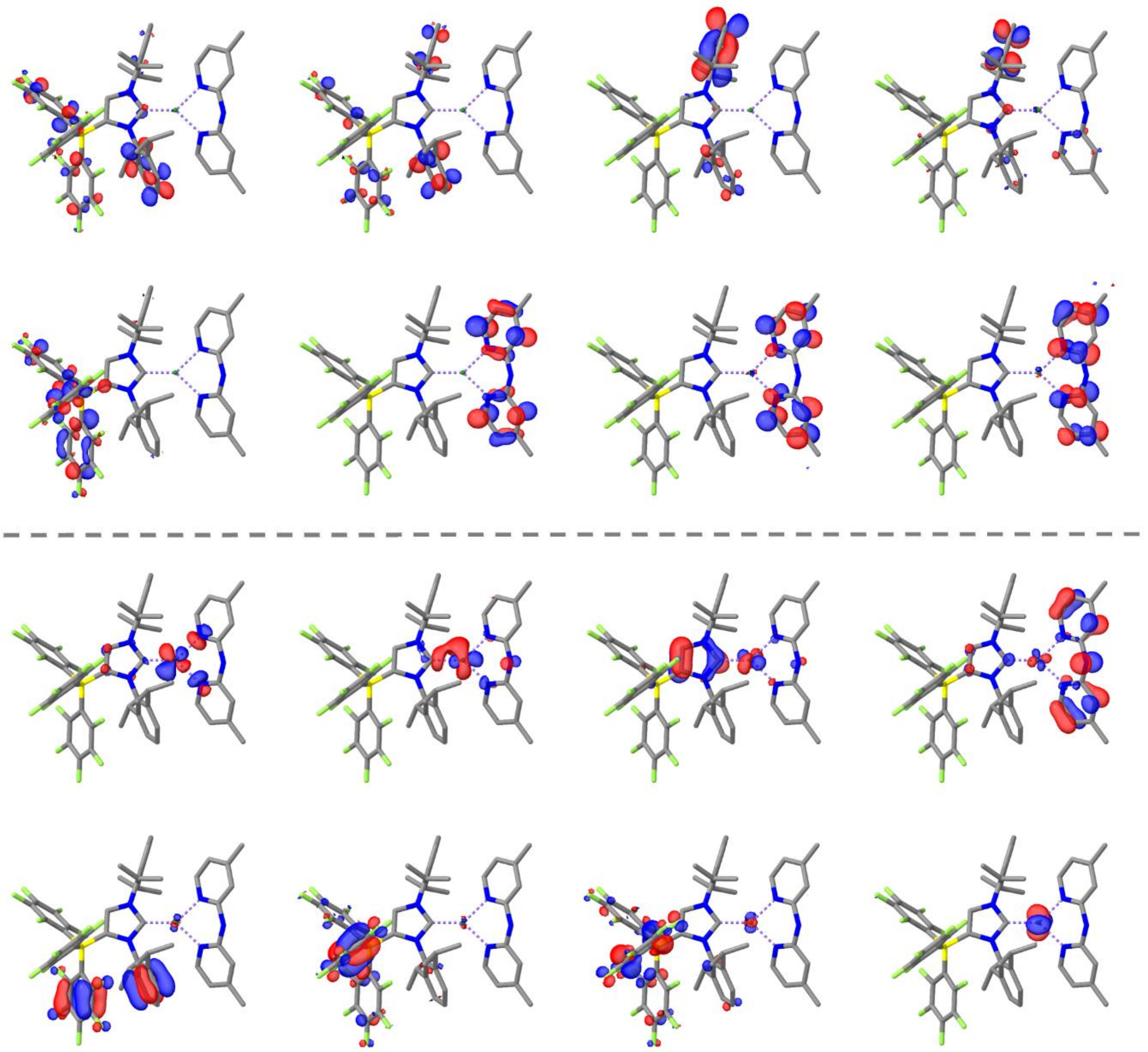

Figure S57. Representation of the LUMO+7, LUMO+6, LUMO+5, LUMO+4, LUMO+3, LUMO+2, LUMO+1, LUMO, HOMO, HOMO-1, HOMO-2, HOMO-3, HOMO-4, HOMO-5, HOMO-6 and HOMO-7 (from top left to bottom right) of **C4**. Volumes are depicted with an isosurface value of 0.05 (blue = negative, red = positive).

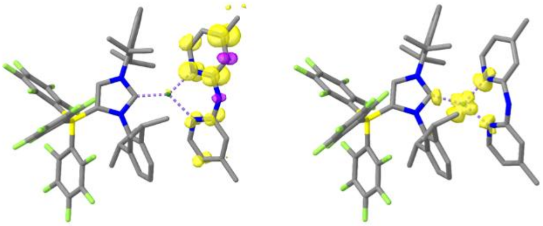

Figure S58. Representation of the spin density of the reduced (left) and oxidized (right) species of **C4** (isosurface value = 0.005).

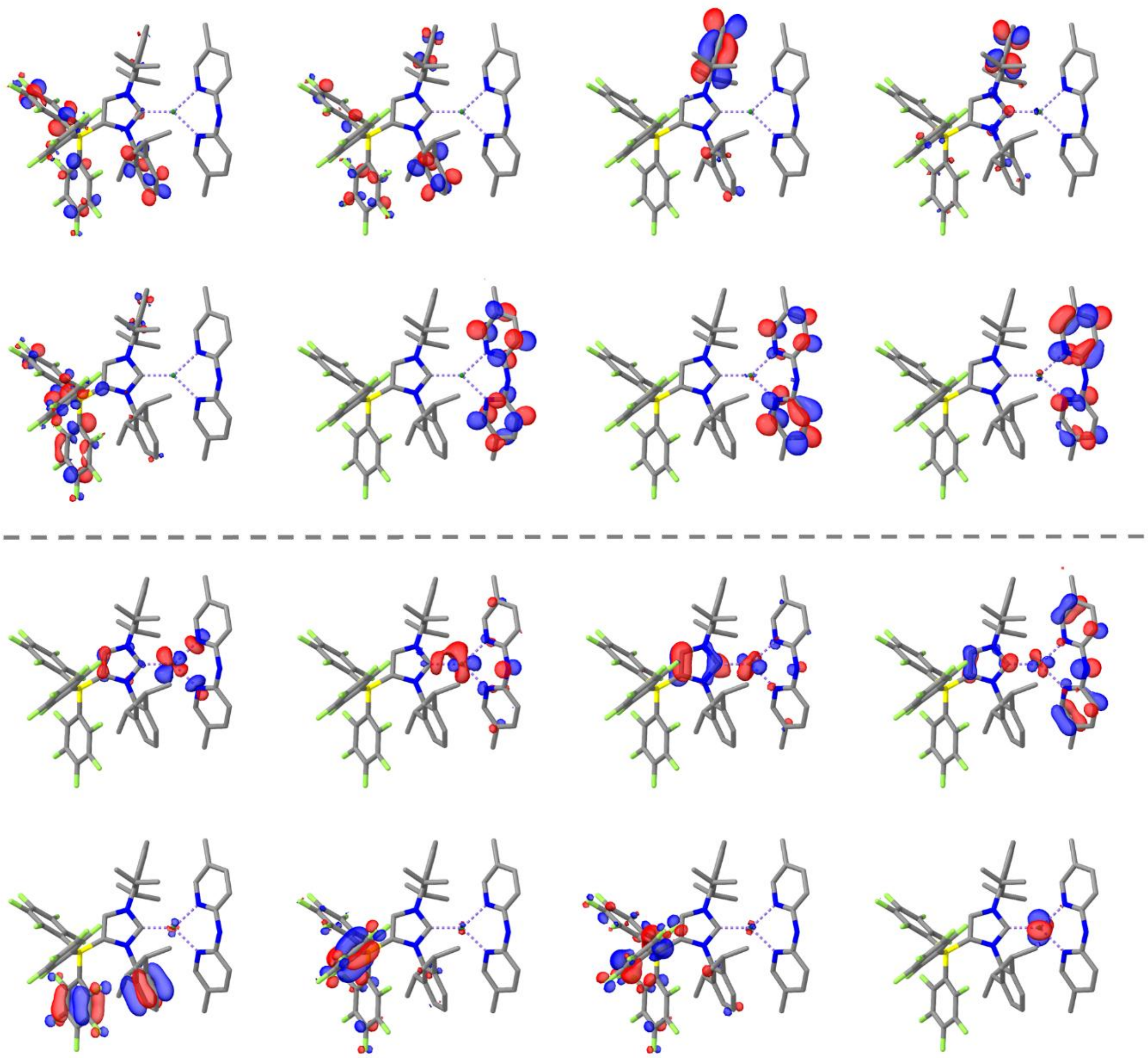

Figure S59. Representation of the LUMO+7, LUMO+6, LUMO+5, LUMO+4, LUMO+3, LUMO+2, LUMO+1, LUMO, HOMO, HOMO-1, HOMO-2, HOMO-3, HOMO-4, HOMO-5, HOMO-6 and HOMO-7 (from top left to bottom right) of **C5**. Volumes are depicted with an isosurface value of 0.05 (blue = negative, red = positive).

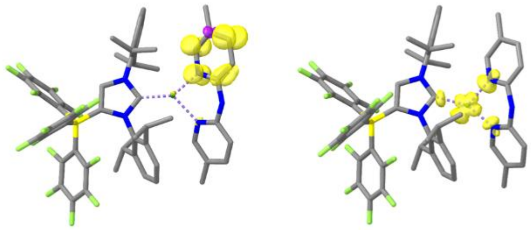

Figure S60. Representation of the spin density of the reduced (left) and oxidized (right) species of **C5** (isosurface value = 0.005).

7 Time-Dependent Density Functional Theory (TDDFT)

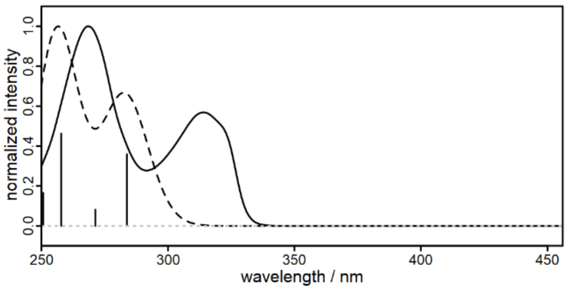

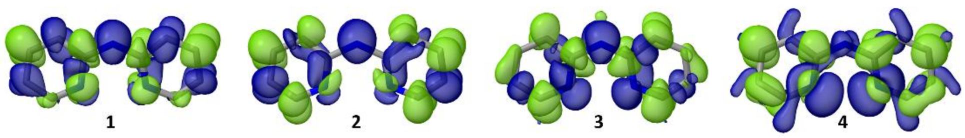

Figure S61. Experimental (solid line, top) and calculated UV/vis absorption spectrum (dashed, top) of **L2** in tetrahydrofuran. The electron difference densities of the corresponding states (blue = -0.001, green = +0.001) are depicted below. Calculated spectrum was acquired by broadening the distinct transition energies with a gaussian line shape (fwhm: 2500 cm^{-1}). Only excitations with $f_{osc} > 0.01$ are shown.

Table S9. Excitation energies, oscillator strengths (f_{osc}) and corresponding transitions of **L2**. Only excitations with $f_{osc} > 0.01$ and corresponding orbital contributions with an OC ≥ 0.1 are given.

State	Exc. Energy		f_{osc}	Orbital Contribution			Transition
#	cm^{-1}	nm		OC	occ. orb.	virt. orb.	
1	35235.1	283.8	0.359	0.922	HOMO	LUMO	π* ← π
2	36859.9	271.3	0.081	0.930	HOMO	LUMO+1	π* ← π
3	38792.8	257.8	0.463	0.856	HOMO	LUMO+2	π* ← π
4	39886.7	250.7	0.165	0.841	HOMO-1	LUMO	π* ← n

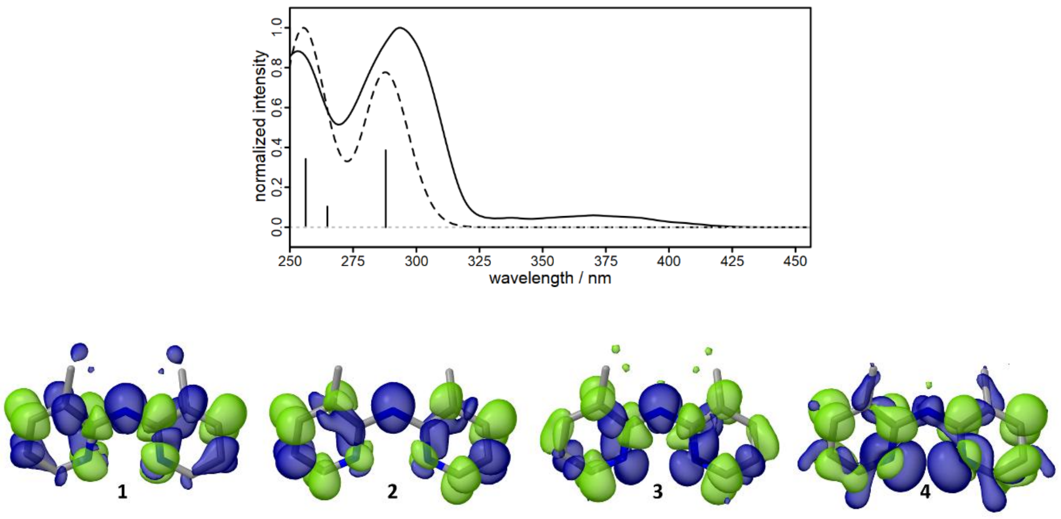

Figure S62. Experimental (solid line, top) and calculated UV/vis absorption spectrum (dashed, top) of **L3** in tetrahydrofuran. The electron difference densities of the corresponding states (blue = -0.001, green = +0.001) are depicted below. Calculated spectrum was acquired by broadening the distinct transition energies with a gaussian line shape (fwhm: 2500 cm^{-1}). Only excitations with $f_{osc} > 0.01$ are shown.

Table S10. Excitation energies, oscillator strengths (f_{osc}) and corresponding transitions of **L3**. Only excitations with $f_{osc} > 0.01$ and corresponding orbital contributions with an OC ≥ 0.1 are given.

**State**	**Exc. Energy**		**f_{osc}**	**Orbital Contribution**			**Transition**
#	cm^{-1}	nm		OC	occ. orb.	virt. orb.	
1	34735.8	287.9	0.386	0.936	HOMO	LUMO	π* ← π
2	37771.3	264.8	0.104	0.913	HOMO	LUMO+1	π* ← π
3	39017.4	256.3	0.343	0.842	HOMO	LUMO+2	π* ← π
4	40098.2	249.4	0.170	0.824	HOMO-1	LUMO	π* ← n

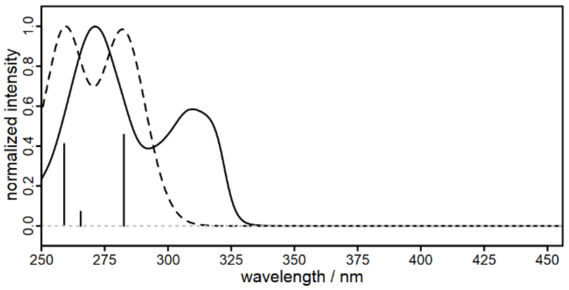

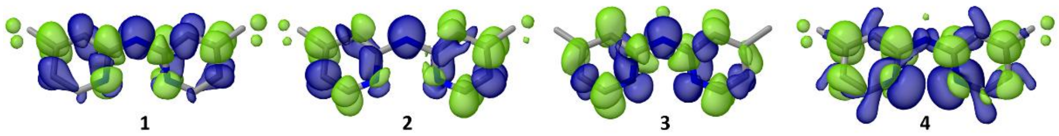

Figure S63. Experimental (solid line, top) and calculated UV/vis absorption spectrum (dashed, top) of **L4** in tetrahydrofuran. The electron difference densities of the corresponding states (blue = -0.001, green = +0.001) are depicted below. Calculated spectrum was acquired by broadening the distinct transition energies with a gaussian line shape (fwhm: 2500 cm^{-1}). Only excitations with $f_{osc} > 0.01$ are shown.

Table S11. Excitation energies, oscillator strengths (f_{osc}) and corresponding transitions of **L4**. Only excitations with $f_{osc} > 0.01$ and corresponding orbital contributions with an OC ≥ 0.1 are given.

**State**	**Exc. Energy**		**f_{osc}**	**Orbital Contribution**			**Transition**
#	cm^{-1}	nm		OC	occ. orb.	virt. orb.	
1	35390.3	282.6	0.457	0.914	HOMO	LUMO	π* ← π
2	37664.2	265.5	0.071	0.916	HOMO	LUMO+1	π* ← π
3	38610.8	259.0	0.410	0.869	HOMO	LUMO+2	π* ← π
4	40850.1	244.8	0.123	0.891	HOMO-1	LUMO	π* ← n

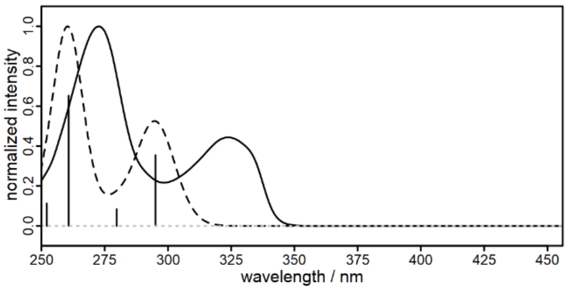

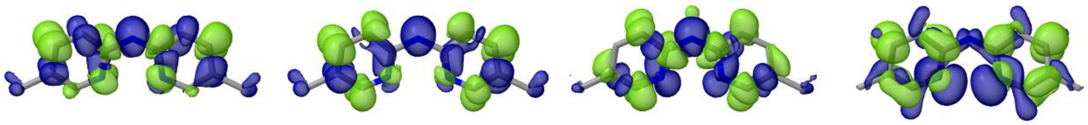

Figure S64. Experimental (solid line, top) and calculated UV/vis absorption spectrum (dashed, top) of **L5** in tetrahydrofuran. The electron difference densities of the corresponding states (blue = -0.001, green = +0.001) are depicted below. Calculated spectrum was acquired by broadening the distinct transition energies with a gaussian line shape (fwhm: 2500 cm^{-1}). Only excitations with $f_{osc} > 0.01$ are shown.

Table S12. Excitation energies, oscillator strengths (f_{osc}) and corresponding transitions of **L5**. Only excitations with $f_{osc} > 0.01$ and corresponding orbital contributions with an OC ≥ 0.1 are given.

**State**	**Exc. Energy**		**f_{osc}**	**Orbital Contribution**			**Transition**
#	cm^{-1}	nm		OC	occ. orb.	virt. orb.	
1	33883.4	295.1	0.354	0.945	HOMO	LUMO	π* ← π
2	35739.4	279.8	0.084	0.941	HOMO	LUMO+1	π* ← π
3	38341.6	260.8	0.653	0.920	HOMO	LUMO+2	π* ← π
4	39660.0	252.1	0.112	0.872	HOMO-1	LUMO	π* ← n

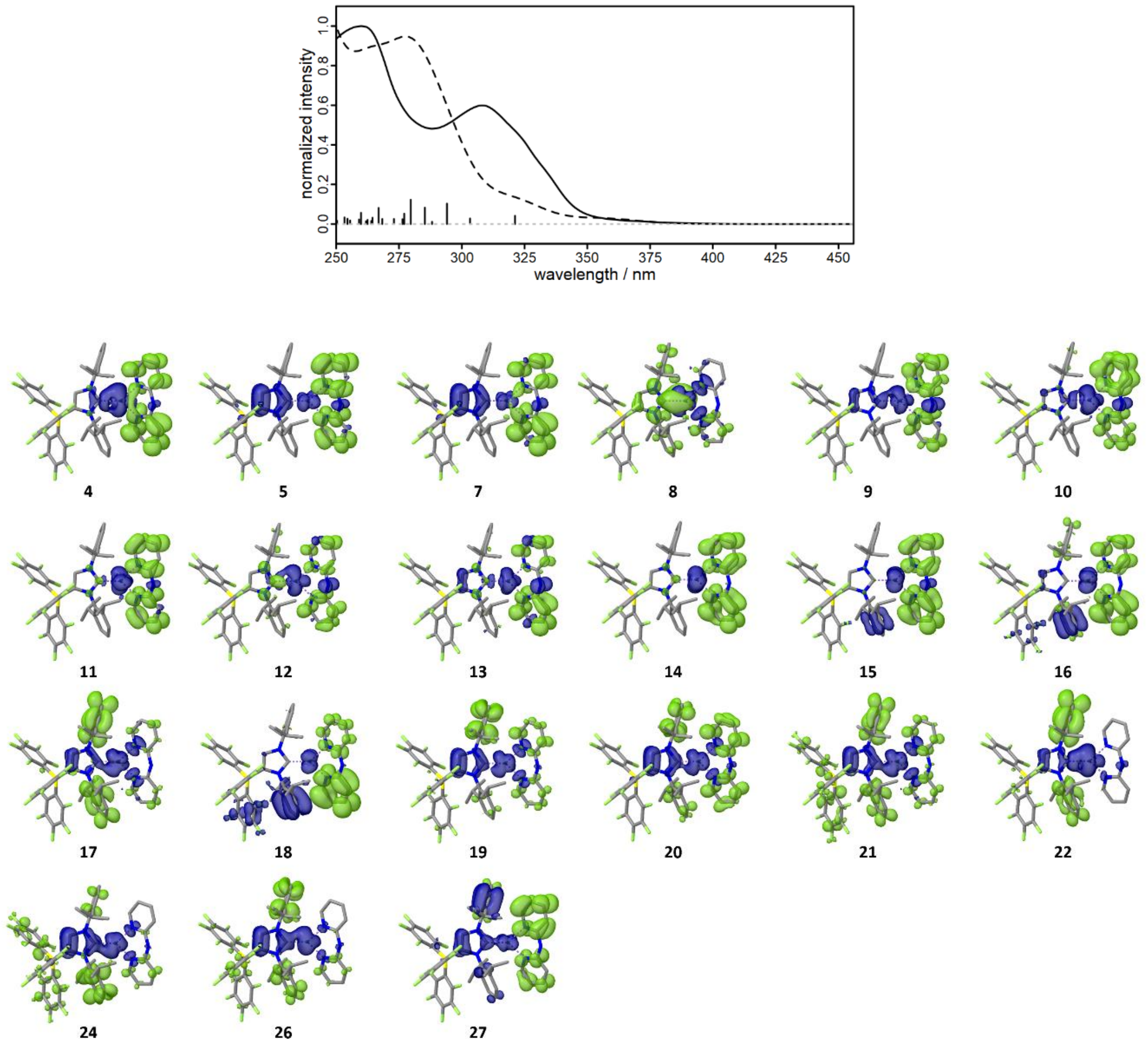

Figure S65. Experimental (solid line, top) and calculated UV/vis absorption spectrum (dashed, top) of **C2** in tetrahydrofuran. The electron difference densities of the corresponding states (blue = -0.001, green = +0.001) are depicted below. Calculated spectrum was acquired by broadening the distinct transition energies with a gaussian line shape (fwhm: 2500 cm^{-1}). Only excitations with $f_{osc} > 0.01$ are shown.

Table S13. Excitation energies, oscillator strengths (f_{osc}) and corresponding transitions of **C2**. Only excitations with $f_{osc} > 0.01$ and corresponding orbital contributions with an OC ≥ 0.1 are given.

**State**	**Exc. Energy**		**f_{osc}**	**Orbital Contribution**			**Transition**
#	cm^{-1}	nm		OC	occ. orb.	virt. orb.	
4	31137.3	321.2	0.041	0.936	HOMO-1	LUMO+1	$\pi^*_{L2} \leftarrow d_{Cu}$
5	32971.4	303.3	0.028	0.860	HOMO-2	LUMO	$\pi^*_{L2} \leftarrow d_{Cu}, \pi_{NHC}$
7	34002.4	294.1	0.103	0.855	HOMO-2	LUMO+1	$\pi^*_{L2} \leftarrow d_{Cu}, \pi_{NHC}$
8	34701.3	288.2	0.111	0.516	HOMO	LUMO+4	$\pi^*_{NHC} \leftarrow d_{Cu}, N_{L2}$
9	35046.5	285.3	0.083	0.494	HOMO-3	LUMO	$\pi^*_{L2} \leftarrow d_{Cu}$
				0.410	HOMO-1	LUMO+2	
10	35747.0	279.7	0.123	0.145	HOMO-8	LUMO	$\pi^*_{L2} \leftarrow d_{Cu}$
				0.253	HOMO-3	LUMO	
				0.397	HOMO-1	LUMO+2	
11	36094.0	277.1	0.052	0.459	HOMO-8	LUMO	$\pi^*_{L2} \leftarrow d_{Cu}$
				0.189	HOMO-7	LUMO	
12	36184.7	276.4	0.024	0.236	HOMO-3	LUMO+1	$\pi^*_{L2}, \pi^*_{NHC} \leftarrow d_{Cu}, n_{NH}$
				0.281	HOMO-1	LUMO+4	
13	36635.0	273.0	0.026	0.507	HOMO-3	LUMO+1	$\pi^*_{L2} \leftarrow d_{Cu}, n_{NH}$
				0.135	HOMO-1	LUMO+4	
14	37256.1	268.4	0.026	0.129	HOMO-10	LUMO	$\pi^*_{L2} \leftarrow d_{Cu}$
				0.408	HOMO-8	LUMO+1	
				0.161	HOMO-7	LUMO+1	
15	37469.9	266.9	0.081	0.255	HOMO-10	LUMO	$\pi^*_{L2} \leftarrow d_{Cu}, \pi_{Dipp}$
				0.126	HOMO-9	LUMO	
				0.112	HOMO-4	LUMO	
				0.122	HOMO-3	LUMO+1	
16	37803.8	264.5	0.032	0.258	HOMO-10	LUMO+1	$\pi^*_{L2} \leftarrow d_{Cu}, \pi_{Dipp}$
				0.121	HOMO-9	LUMO+1	
				0.139	HOMO-4	LUMO+1	
17	37879.6	264.0	0.015	0.167	HOMO	LUMO+3	$\pi^*_{Dipp} \leftarrow d_{Cu}, \pi_{NHC}, N_{L2}$
				0.555	HOMO	LUMO+5	
18	38096.7	262.5	0.020	0.108	HOMO-10	LUMO+1	$\pi^*_{L2} \leftarrow d_{Cu}, \pi_{Dipp}$
				0.113	HOMO-9	LUMO+1	
				0.303	HOMO-4	LUMO	
19	38181.5	261.9	0.013	0.193	HOMO-2	LUMO+2	$\pi^*_{L2}, \pi^*_{Dipp} \leftarrow d_{Cu}, \pi_{NHC}, N_{L2}$
				0.159	HOMO	LUMO+3	
				0.153	HOMO	LUMO+5	
				0.165	HOMO	LUMO+6	
20	38483.2	259.9	0.056	0.435	HOMO-2	LUMO+2	$\pi^*_{L2}, \pi^*_{Dipp} \leftarrow d_{Cu}, \pi_{NHC}, N_{L2}$
				0.249	HOMO	LUMO+6	
21	38597.5	259.1	0.022	0.118	HOMO-2	LUMO+2	$\pi^*_{Dipp} \leftarrow d_{Cu}, \pi_{NHC}, N_{L2}$
				0.251	HOMO	LUMO+3	

				0.123	HOMO	LUMO+5	
				0.159	HOMO	LUMO+7	
				0.106	HOMO	LUMO+8	
22	39119.6	255.6	0.181	0.654	HOMO-1	LUMO+5	$\pi^*_{Dipp} \leftarrow d_{Cu}, \pi_{NHC}$
24	39293.8	254.5	0.027	0.246	HOMO-2	LUMO+3	$\pi^*_{Dipp} \leftarrow d_{Cu}, \pi_{NHC}, N_{L2}$
				0.214	HOMO	LUMO+6	
26	39485.0	253.3	0.033	0.300	HOMO-2	LUMO+4	$\pi^*_{Dipp} \leftarrow d_{Cu}, \pi_{NHC}, N_{L2}$
				0.175	HOMO-1	LUMO+5	
27	39928.4	250.4	0.016	0.178	HOMO-11	LUMO	$\pi^*_{L2} \leftarrow d_{Cu}, \pi_{NHC}, \pi_{Dipp}$
				0.166	HOMO-10	LUMO	
				0.156	HOMO-9	LUMO	

N_{L2} assigns the N-donor orbitals of the coordinating N-atoms. n_{NH} assigns the lone pair of the amine function.

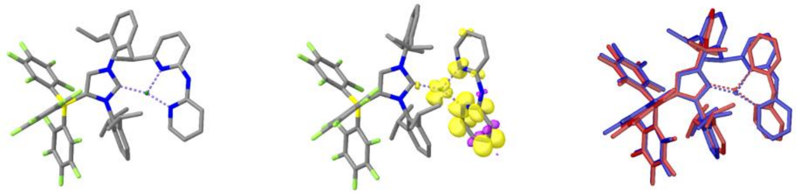

Figure S66. Optimized geometries of the S_1 state (left) and T_1 state (center) and overlay of the geometries of both excited states (S_1 = blue, T_1 = red, right) of **C2**. The T_1 state (center) is depicted with the corresponding spin density (isosurface value = 0.005).

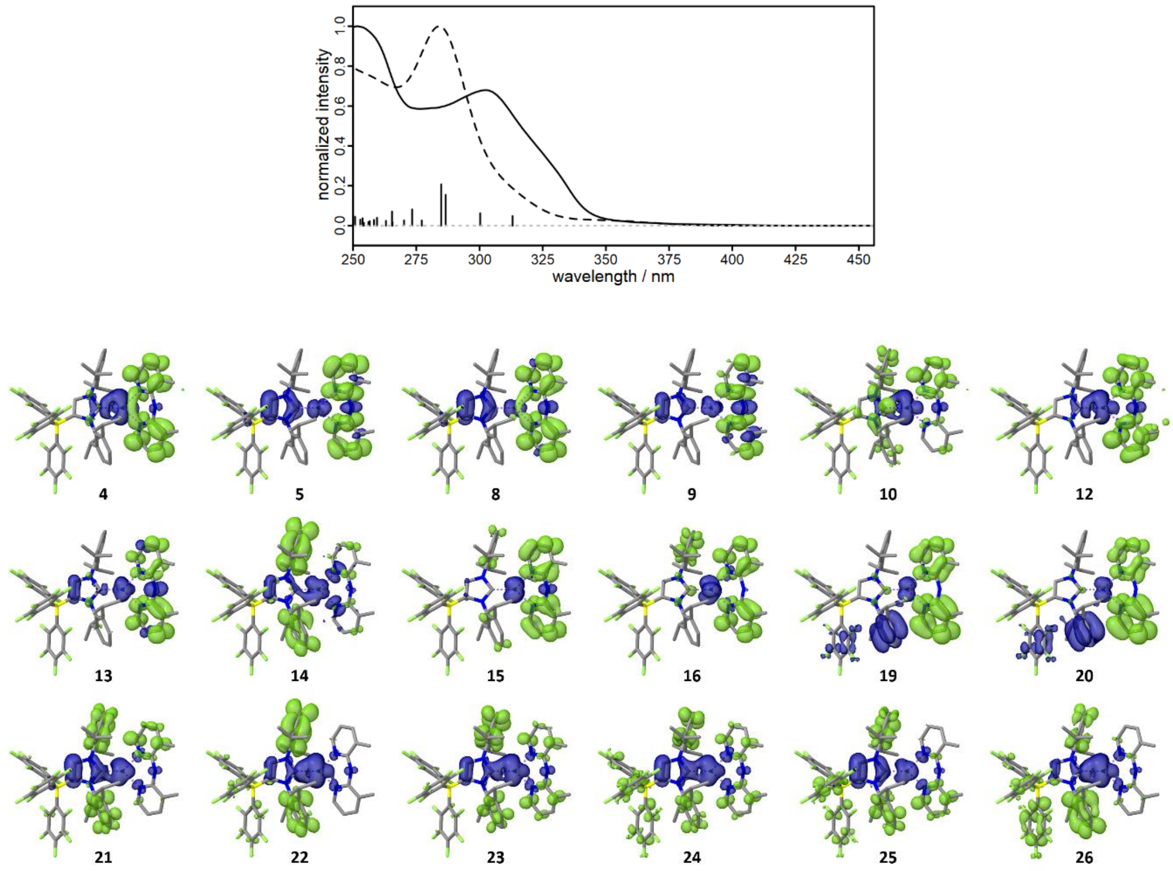

Figure S67. Experimental (solid line, top) and calculated UV/vis absorption spectrum (dashed, top) of **C3** in tetrahydrofuran. The electron difference densities of the corresponding states (blue = -0.001, green = +0.001) are depicted below. Calculated spectrum was acquired by broadening the distinct transition energies with a gaussian line shape (fwhm: 2500 cm^{-1}). Only excitations with $f_{osc} > 0.01$ are shown.

Table S14. Excitation energies, oscillator strengths (f_{osc}) and corresponding transitions of **C3**. Only excitations with $f_{osc} > 0.01$ and corresponding orbital contributions with an OC ≥ 0.1 are given.

State	Exc. Energy		f_{osc}	Orbital Contribution			Transition
#	cm^{-1}	nm		OC	occ. orb.	virt. orb.	
4	31943.3	313.1	0.048	0.908	HOMO-1	LUMO+1	$\pi^*_{L3} \leftarrow d_{Cu}$
5	33295.7	300.3	0.062	0.860	HOMO-2	LUMO	$\pi^*_{L3} \leftarrow d_{Cu}, \pi_{NHC}$
8	34887.0	286.6	0.155	0.815	HOMO-2	LUMO+1	$\pi^*_{L3} \leftarrow d_{Cu}, \pi_{NHC}$
9	35100.1	284.9	0.207	0.731	HOMO-3	LUMO	$\pi^*_{L3} \leftarrow d_{Cu}, \pi_{NHC}$
				0.104	HOMO-1	LUMO+2	
10	36074.6	277.2	0.026	0.220	HOMO-1	LUMO+2	$\pi^*_{NHC}, \pi^*_{L3} \leftarrow d_{Cu}$
				0.322	HOMO-1	LUMO+4	
12	36574.9	273.4	0.081	0.579	HOMO-1	LUMO+2	$\pi^*_{L3} \leftarrow d_{Cu}$
13	37013.0	270.2	0.026	0.582	HOMO-3	LUMO+1	$\pi^*_{L3} \leftarrow d_{Cu}, \pi_{NHC}, n_{NH}$
14	37648.8	265.6	0.018	0.104	HOMO	LUMO+3	$\pi^*_{Dipp} \leftarrow d_{Cu}, \pi_{NHC}, N_{L3}$
				0.604	HOMO	LUMO+5	
15	37680.1	265.4	0.070	0.151	HOMO-10	LUMO	$\pi^*_{L3} \leftarrow d_{Cu}, n_{NH}$
				0.301	HOMO-9	LUMO	
				0.130	HOMO-3	LUMO+1	
16	38023.4	263.0	0.024	0.481	HOMO-7	LUMO+1	$\pi^*_{L3}, \pi^*_{Dipp} \leftarrow d_{Cu}$
19	38539.8	259.5	0.039	0.141	HOMO-10	LUMO+1	$\pi^*_{L3} \leftarrow d_{Cu}, \pi_{Dipp}$
				0.210	HOMO-9	LUMO+1	
				0.264	HOMO-4	LUMO	
20	38717.5	258.3	0.029	0.269	HOMO-9	LUMO+1	$\pi^*_{L3} \leftarrow d_{Cu}, \pi_{Dipp}$
				0.379	HOMO-4	LUMO	
21	38967.4	256.6	0.022	0.317	HOMO-2	LUMO+2	$\pi^*_{Dipp} \leftarrow d_{Cu}, \pi_{NHC}$
				0.173	HOMO-1	LUMO+5	
22	39042.6	256.1	0.018	0.531	HOMO-1	LUMO+5	$\pi^*_{Dipp} \leftarrow d_{Cu}, \pi_{NHC}$
23	39318.4	254.3	0.014	0.237	HOMO-2	LUMO+2	$\pi^*_{Dipp}, \pi^*_{L3} \leftarrow d_{Cu}, \pi_{NHC}$
				0.239	HOMO-1	LUMO+6	
24	39398.1	253.8	0.037	0.137	HOMO-2	LUMO+2	$\pi^*_{Dipp}, \pi^*_{L3} \leftarrow d_{Cu}, \pi_{NHC}$
				0.117	HOMO-2	LUMO+3	
				0.193	HOMO-1	LUMO+6	
				0.132	HOMO	LUMO+8	
25	39545.4	252.9	0.029	0.332	HOMO-2	LUMO+4	$\pi^*_{Dipp}, \pi^*_{WCA} \leftarrow d_{Cu}, \pi_{NHC}$
				0.117	HOMO	LUMO+4	
26	39870.0	250.8	0.045	0.267	HOMO-1	LUMO+3	$\pi^*_{Dipp}, \pi^*_{WCA} \leftarrow d_{Cu}, \pi_{NHC}$
				0.209	HOMO-1	LUMO+8	

N_{L3} assigns the N-donor orbitals of the coordinating N-atoms. n_{NH} assigns the lone pair of the amine function.

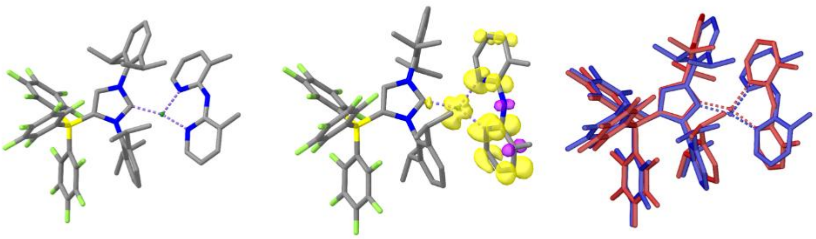

Figure S68. Optimized geometries of the S_1 state (left) and T_1 state (center) and overlay of the geometries of both excited states (S_1 = blue, T_1 = red, right) of **C3**. The T_1 state (center) is depicted with the corresponding spin density (isosurface value = 0.005).

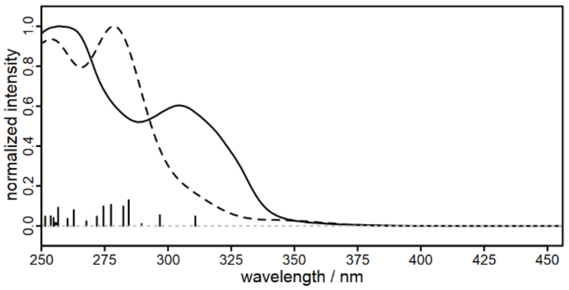

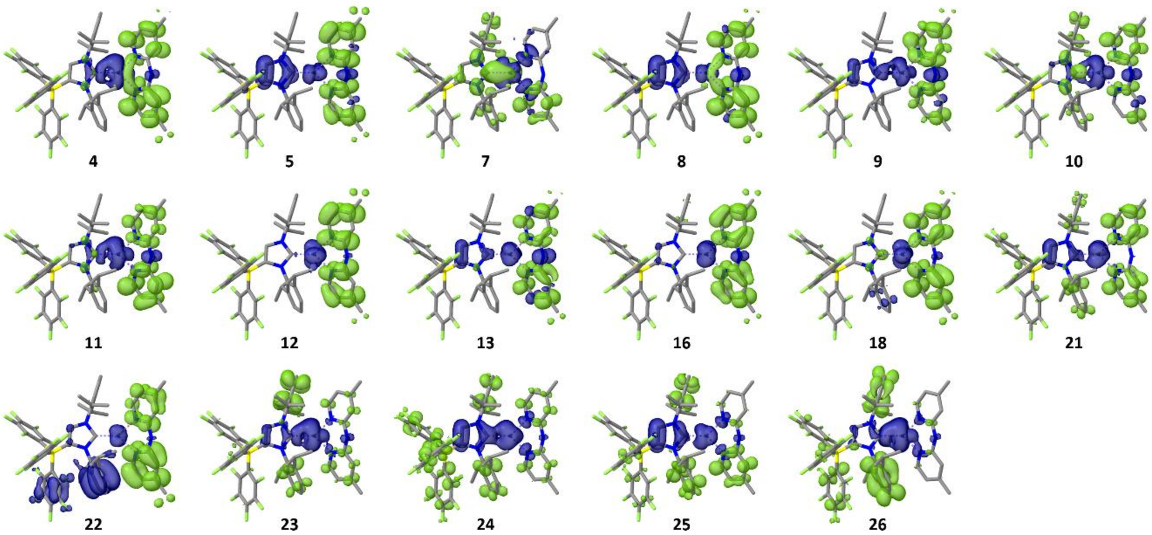

4 5 7 8 9 10

11 12 13 16 18 21

22 23 24 25 26

Figure S69. Experimental (solid line, top) and calculated UV/vis absorption spectrum (dashed, top) of **C4** in tetrahydrofuran. The electron difference densities of the corresponding states (blue = -0.001, green = +0.001) are depicted below. Calculated spectrum was acquired by broadening the distinct transition energies with a gaussian line shape (fwhm: 2500 cm^{-1}). Only excitations with $f_{osc} > 0.01$ are shown.

Table S15. Excitation energies, oscillator strengths (f_{osc}) and corresponding transitions of **C4**. Only excitations with $f_{osc} > 0.01$ and corresponding orbital contributions with an OC ≥ 0.1 are given.

State	Exc. Energy		f_{osc}	Orbital Contribution			Transition
#	cm^{-1}	nm		OC	occ. orb.	virt. orb.	
4	32176.5	310.8	0.047	0.914	HOMO-1	LUMO+1	$\pi^*_{L4} \leftarrow d_{Cu}$
5	33689.0	296.8	0.054	0.821	HOMO-2	LUMO	$\pi^*_{L4} \leftarrow d_{Cu}, \pi_{NHC}$
7	34533.3	289.6	0.102	0.513	HOMO	LUMO+4	$\pi^*_{NHC} \leftarrow d_{Cu}, N_{L4}$
8	35149.6	284.5	0.129	0.835	HOMO-2	LUMO+1	$\pi^*_{L4} \leftarrow d_{Cu}, \pi_{NHC}, n_{NH}$
9	35412.2	282.4	0.098	0.475	HOMO-3	LUMO	$\pi^*_{L4} \leftarrow d_{Cu}, \pi_{NHC}, n_{NH}$
				0.390	HOMO-1	LUMO+2	
10	36038.6	277.5	0.106	0.195	HOMO-3	LUMO	$\pi^*_{NHC}, \pi^*_{L4} \leftarrow d_{Cu}$
				0.138	HOMO-1	LUMO+2	
				0.296	HOMO-1	LUMO+4	
11	36430.3	274.5	0.098	0.135	HOMO-3	LUMO	$\pi^*_{L4} \leftarrow d_{Cu}, n_{NH}$
				0.361	HOMO-1	LUMO+2	
				0.197	HOMO-1	LUMO+4	
12	36774.3	271.9	0.046	0.762	HOMO-7	LUMO	$\pi^*_{L4} \leftarrow d_{Cu}$
13	37336.2	267.8	0.023	0.172	HOMO-9	LUMO	$\pi^*_{L4} \leftarrow d_{Cu}, \pi_{NHC}, n_{NH}$
				0.603	HOMO-3	LUMO+1	
16	38052.0	262.8	0.079	0.438	HOMO-9	LUMO	$\pi^*_{L4} \leftarrow d_{Cu}$
				0.135	HOMO-7	LUMO+1	
				0.162	HOMO-3	LUMO+1	
18	38412.0	260.3	0.035	0.617	HOMO-7	LUMO+1	$\pi^*_{L4} \leftarrow d_{Cu}$
21	38970.1	256.6	0.091	0.306	HOMO-9	LUMO+1	$\pi^*_{L4} \leftarrow d_{Cu}, \pi_{NHC}$
				0.103	HOMO-2	LUMO+2	
22	39069.9	256.0	0.014	0.490	HOMO-4	LUMO	$\pi^*_{L4} \leftarrow d_{Cu}, \pi_{Dipp}, \pi_{WCA}$
23	39160.3	255.4	0.015	0.109	HOMO-1	LUMO+3	$\pi^*_{Dipp} \leftarrow d_{Cu}, \pi_{NHC}$
				0.268	HOMO-1	LUMO+6	
24	39242.6	254.8	0.041	0.129	HOMO-2	LUMO+3	$\pi^*_{Dipp}, \pi^*_{WCA} \leftarrow d_{Cu}, \pi_{NHC}, N_{L4}$
				0.116	HOMO-1	LUMO+6	
				0.113	HOMO	LUMO+3	
25	39421.5	253.7	0.049	0.323	HOMO-2	LUMO+4	$\pi^*_{Dipp}, \pi^*_{L4} \leftarrow d_{Cu}, \pi_{NHC}, N_{L4}$
				0.113	HOMO	LUMO+4	
26	39763.9	251.5	0.046	0.240	HOMO-1	LUMO+3	$\pi^*_{Dipp}, \pi^*_{WCA} \leftarrow d_{Cu}$
				0.193	HOMO-1	LUMO+7	
				0.165	HOMO-1	LUMO+8	

N_{L4} assigns the N-donor orbitals of the coordinating N-atoms. n_{NH} assigns the lone pair of the amine function.

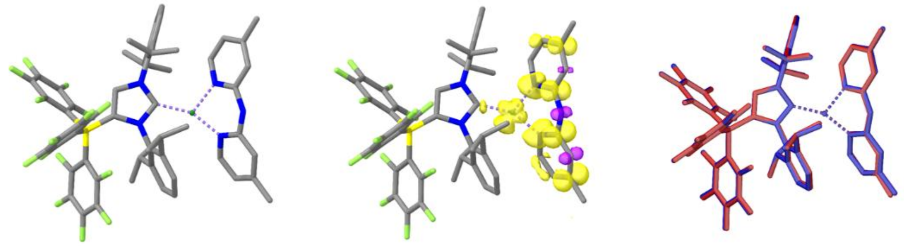

Figure S70. Optimized geometries of the S_1 state (left) and T_1 state (center) and overlay of the geometries of both excited states (S_1 = blue, T_1 = red, right) of **C4**. The T_1 state (center) is depicted with the corresponding spin density (isosurface value = 0.005).

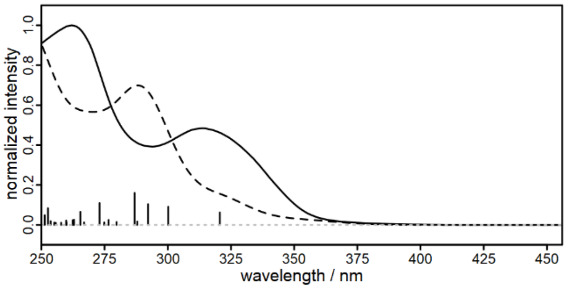

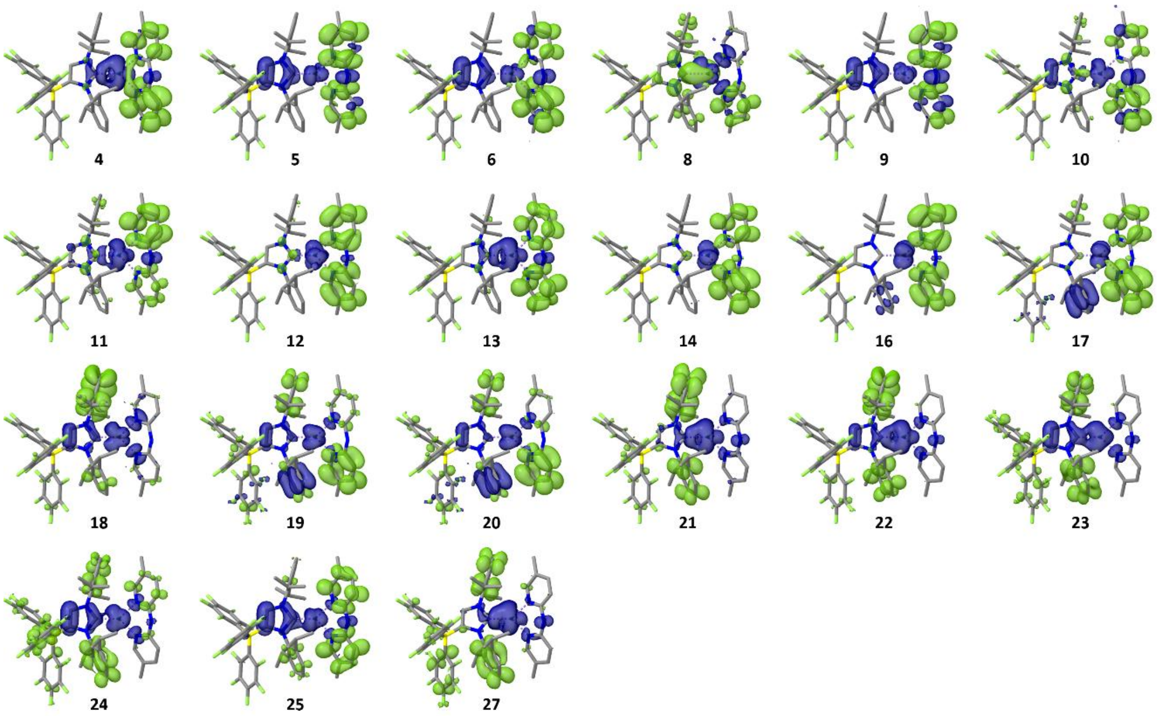

Figure S71. Experimental (solid line, top) and calculated UV/vis absorption spectrum (dashed, top) of **C5** in tetrahydrofuran. The electron difference densities of the corresponding states (blue = -0.001, green = +0.001) are depicted below. Calculated spectrum was acquired by broadening the distinct transition energies with a gaussian line shape (fwhm: 2500 cm^{-1}). Only excitations with $f_{osc} > 0.01$ are shown.

Table S16. Excitation energies, oscillator strengths (f_{osc}) and corresponding transitions of **C5**. Only excitations with $f_{osc} > 0.01$ and corresponding orbital contributions with an OC ≥ 0.1 are given.

State	Exc. Energy		f_{osc}	Orbital Contribution			Transition
#	cm^{-1}	nm		OC	occ. orb.	virt. orb.	
4	31195.8	320.6	0.062	0.897	HOMO-1	LUMO+1	$\pi^*_{L5} \leftarrow d_{Cu}$
5	33316.6	300.2	0.091	0.846	HOMO-2	LUMO	$\pi^*_{L5} \leftarrow d_{Cu}, \pi_{NHC}, n_{NH}$
6	34221.2	292.2	0.103	0.819	HOMO-2	LUMO+1	$\pi^*_{L5} \leftarrow d_{Cu}, \pi_{NHC}, n_{NH}$
8	34732.7	287.9	0.017	0.412	HOMO	LUMO+4	$\pi^*_{NHC}, d_{Cu} \leftarrow d_{Cu}, N_{L5}$
9	34858.7	286.9	0.160	0.799	HOMO-3	LUMO	$\pi^*_{L5} \leftarrow d_{Cu}, \pi_{NHC}, n_{NH}$
10	35741.4	279.8	0.014	0.549	HOMO-3	LUMO+1	$\pi^*_{L5} \leftarrow d_{Cu}, \pi_{NHC}, n_{NH}$
				0.155	HOMO-1	LUMO+4	
11	36153.9	276.6	0.025	0.184	HOMO-7	LUMO	$\pi^*_{L5} \leftarrow d_{Cu}, n_{NH}$
				0.199	HOMO-3	LUMO+1	
				0.286	HOMO-1	LUMO+2	
				0.113	HOMO-1	LUMO+4	
12	36380.8	274.9	0.012	0.610	HOMO-7	LUMO	$\pi^*_{L5} \leftarrow d_{Cu}$
13	36633.4	273.0	0.109	0.540	HOMO-1	LUMO+2	$\pi^*_{L5} \leftarrow d_{Cu}, n_{NH}$
14	37464.3	266.9	0.012	0.757	HOMO-7	LUMO+1	$\pi^*_{L5} \leftarrow d_{Cu}$
16	37677.4	265.4	0.066	0.102	HOMO-11	LUMO	$\pi^*_{L5} \leftarrow d_{Cu}$
				0.452	HOMO-9	LUMO	
17	38039.5	262.9	0.026	0.332	HOMO-9	LUMO+1	$\pi^*_{L5} \leftarrow d_{Cu}, \pi_{Dipp}$
				0.153	HOMO-4	LUMO+1	
18	38105.6	262.4	0.023	0.150	HOMO	LUMO+3	$\pi^*_{Dipp} \leftarrow d_{Cu}, \pi_{NHC}, N_{L5}$
				0.320	HOMO	LUMO+5	
				0.249	HOMO	LUMO+6	
19	38468.0	260.0	0.012	0.162	HOMO-4	LUMO	$\pi^*_{Dipp}, \pi^*_{L5} \leftarrow d_{Cu}, \pi_{Dipp}$
				0.116	HOMO	LUMO+4	
20	38493.4	259.8	0.021	0.153	HOMO-4	LUMO	$\pi^*_{Dipp}, \pi^*_{L5} \leftarrow d_{Cu}, \pi_{Dipp}$
				0.104	HOMO	LUMO+4	
21	38791.9	257.8	0.011	0.233	HOMO-1	LUMO+3	$\pi^*_{Dipp} \leftarrow d_{Cu}$
				0.461	HOMO-1	LUMO+5	
22	39109.9	255.7	0.010	0.195	HOMO-1	LUMO+5	$\pi^*_{Dipp} \leftarrow d_{Cu}, N_{L5}, n_{NH}, \pi_{NHC}$
				0.221	HOMO-1	LUMO+6	
				0.110	HOMO	LUMO+6	
23	39207.2	255.1	0.012	0.102	HOMO-1	LUMO+6	$\pi^*_{Dipp} \leftarrow d_{Cu}, N_{L5}, n_{NH}, \pi_{NHC}$
				0.126	HOMO	LUMO+6	
				0.156	HOMO	LUMO+8	
24	39421.9	253.7	0.019	0.180	HOMO-2	LUMO+4	$\pi^*_{Dipp} \leftarrow d_{Cu}, N_{L4}, \pi_{NHC}$
				0.118	HOMO	LUMO+8	
25	39594.3	252.6	0.084	0.607	HOMO-2	LUMO+2	$\pi^*_{L5} \leftarrow d_{Cu}, \pi_{NHC}$
27	39800.9	251.3	0.049	0.183	HOMO-1	LUMO+3	$\pi^*_{Dipp} \leftarrow d_{Cu}, n_{NH}$
				0.139	HOMO-1	LUMO+4	

0.139	HOMO-1	LUMO+7
0.197	HOMO-1	LUMO+8

N_{L5} assigns the N-donor orbitals of the coordinating N-atoms. n_{NH} assigns the lone pair of the amine function.

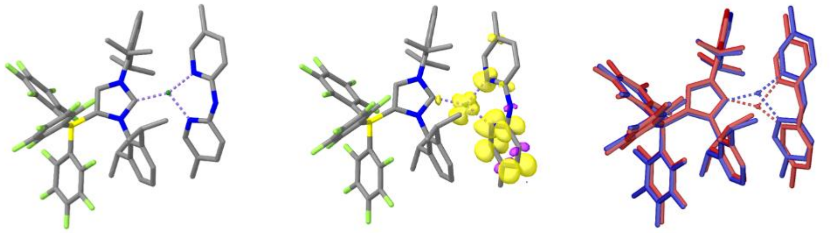

Figure S72. Optimized geometries of the S_1 state (left) and T_1 state (center) and overlay of the geometries of both excited states (S_1 = blue, T_1 = red, right) of **C5**. The T_1 state (center) is depicted with the corresponding spin density (isosurface value = 0.005).

Table S17. Relevant angles (in °) around the Cu(I) center of the optimized relaxed geometries of S_0, S_1 and T_1 states of the complexes **C2**-**C5**.

	**Angle**	**S_0**	**S_1**	**T_1**
**C2**	C1-Cu1-N3	136.47	160.76	155.55
	C1-Cu1-N5	134.63	107.70	112.75
	N3-Cu1-N5	87.87	91.01	89.91
**C3**	C1-Cu1-N3	137.07	159.70	155.01
	C1-Cu1-N5	134.52	107.77	112.23
	N3-Cu1-N5	87.63	91.64	90.31
**C4**	C1-Cu1-N3	136.61	154.65	154.68
	C1-Cu1-N5	134.61	112.37	111.97
	N3-Cu1-N5	87.79	91.45	90.84
**C5**	C1-Cu1-N3	135.69	114.81	154.69
	C1-Cu1-N5	135.34	153.78	113.12
	N3-Cu1-N5	87.76	90.73	89.98

8 UV/vis Absorption

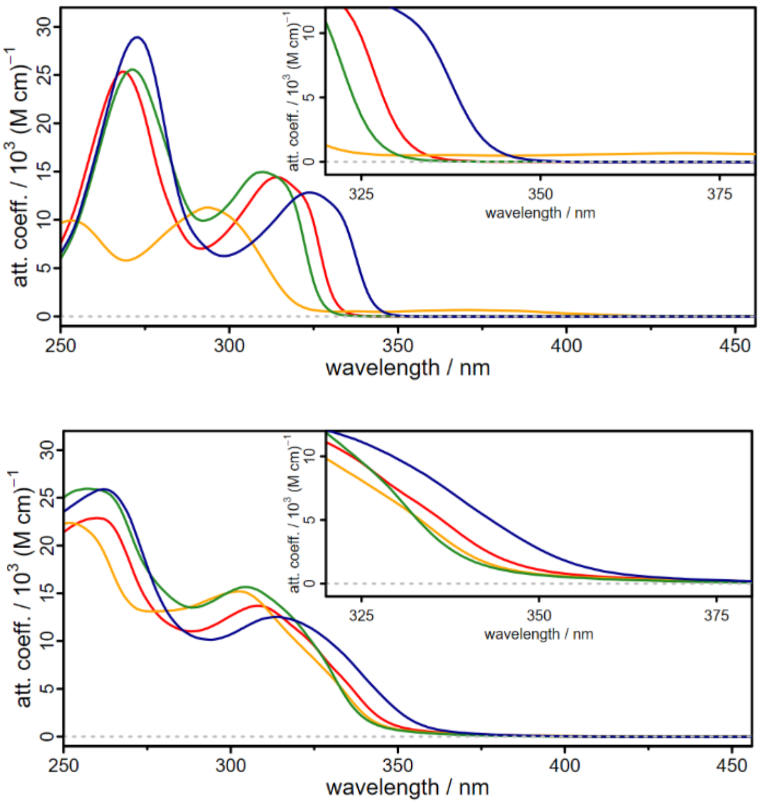

Figure S73. Experimental UV/vis absorption spectra of the ligands top; **L2** (red), **L3** (orange), **L4** (green) and **L5** (blue) and of the complexes bottom; **C2** (red), **C3** (orange), **C4** (green) and **C5** (blue). The inset shows a zoom of the respective spectra in the range from 320 to 380 nm.

9 Time-resolved Emission

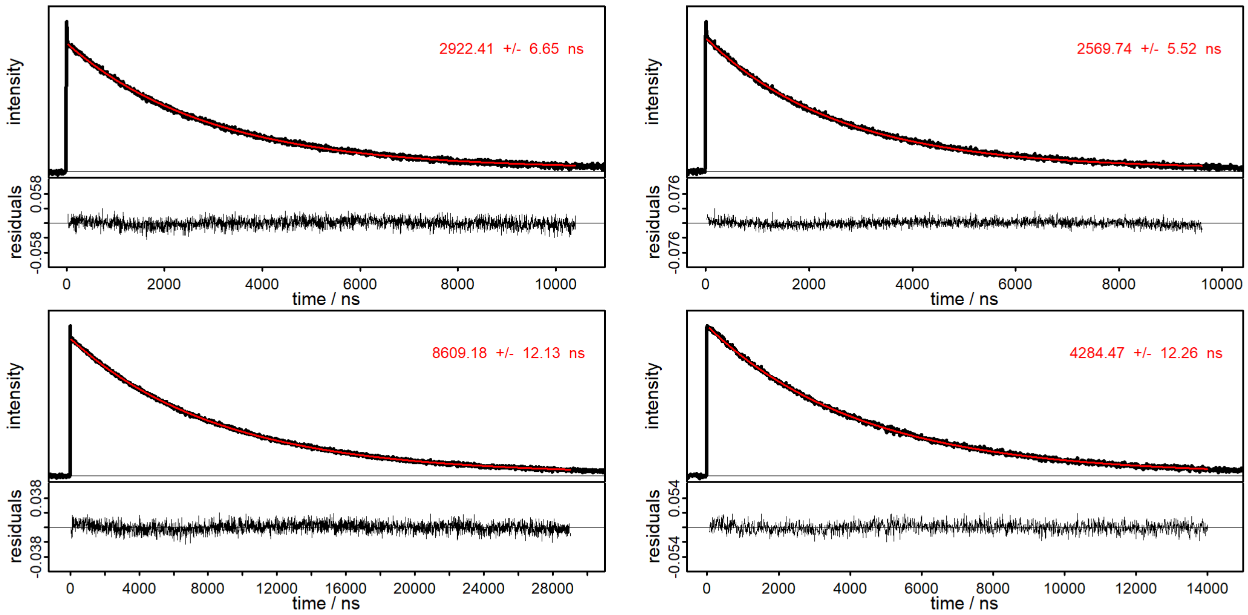

Figure S74. Emission decay curves obtained from time-resolved emission measurements of **C2** (top left), **C3** (top right), **C4** (bottom left) and **C5** (bottom right) in tetrahydrofuran (inert). All lifetimes were estimated using a mono-exponential fit function (shown in red). Residuals of the fit are depicted below.

10 Temperature Dependent Spectroscopy

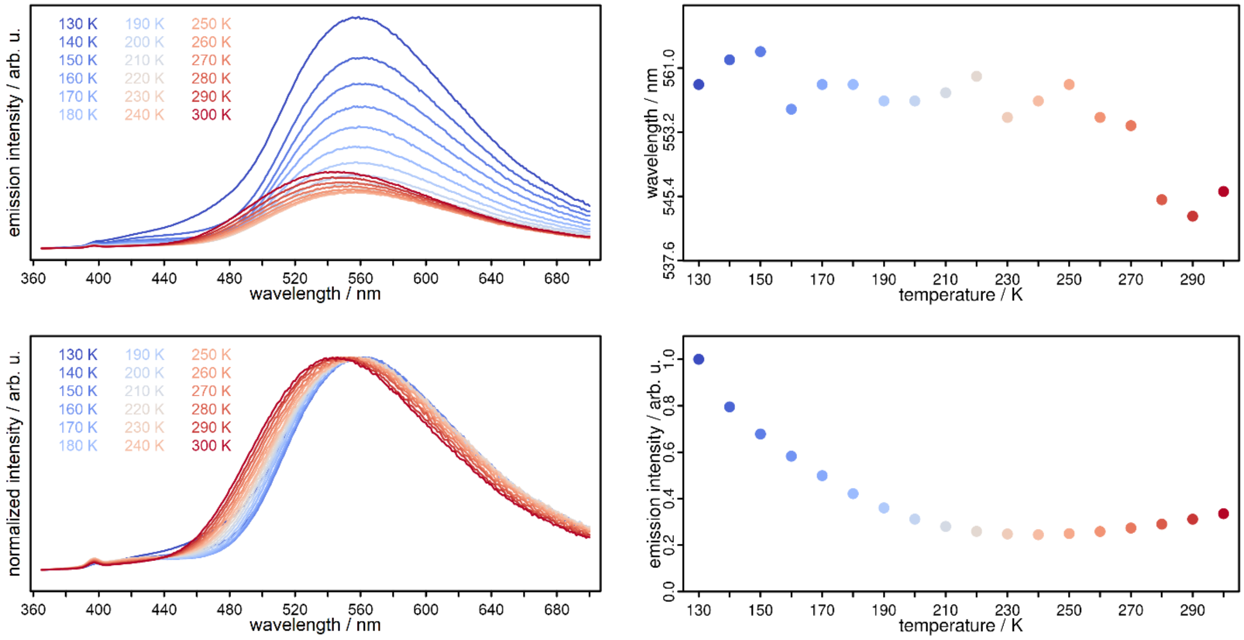

Figure S75. Temperature dependent emission spectra (top left), normalized temperature dependent emission spectra (bottom left), wavelength of emission maxima (top right) and emission intensity at different temperatures (bottom right) of **C2** in dry and inert 2-methyltetrahydrofuran.

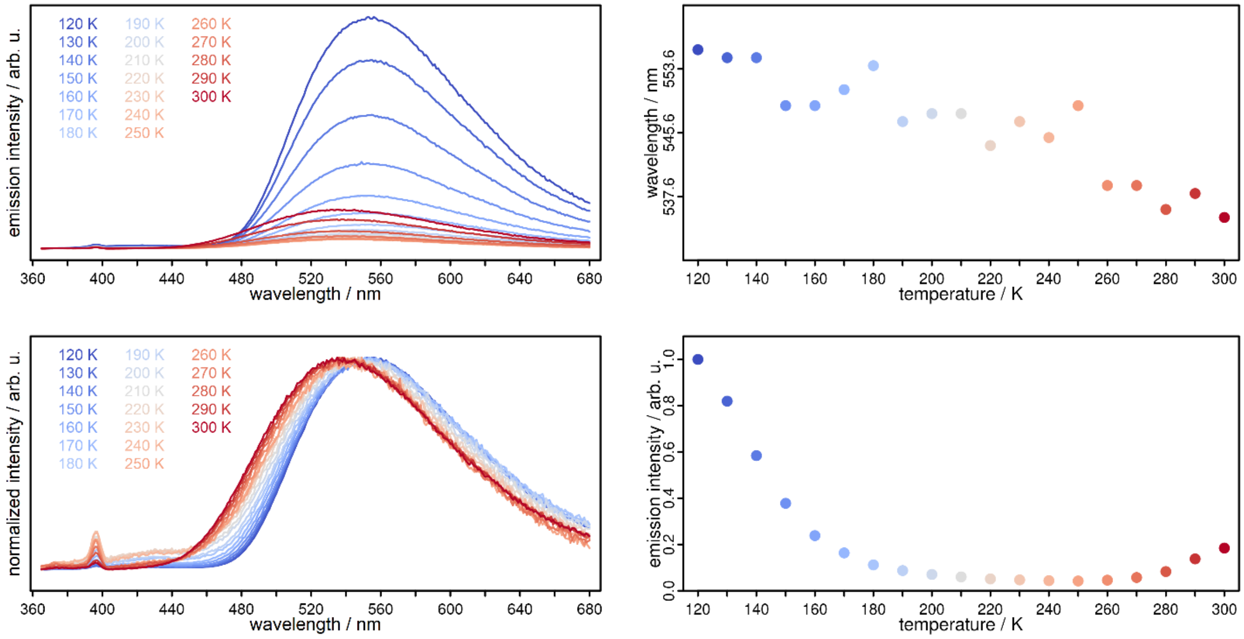

Figure S76. Temperature dependent emission spectra (top left), normalized temperature dependent emission spectra (bottom left), wavelength of emission maxima (top right) and emission intensity at different temperatures (bottom right) of **C3** in dry and inert 2-methyltetrahydrofuran.

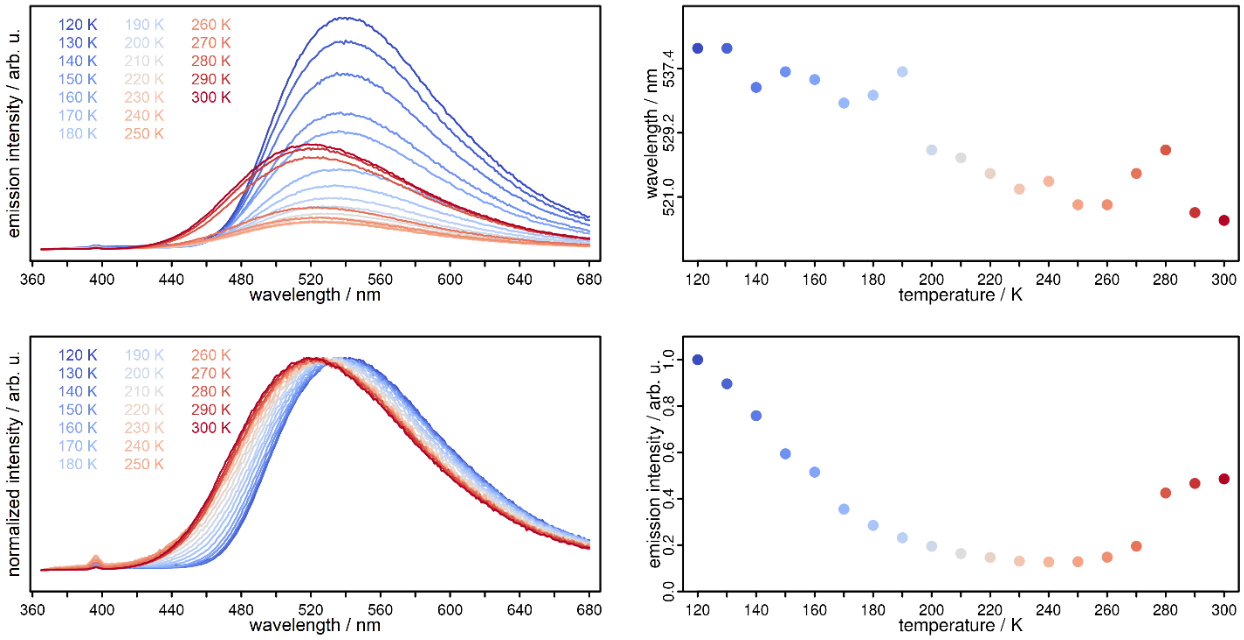

Figure S77. Temperature dependent emission spectra (top left), normalized temperature dependent emission spectra (bottom left), wavelength of emission maxima (top right) and emission intensity at different temperatures (bottom right) of **C4** in dry and inert 2-methyltetrahydrofuran.

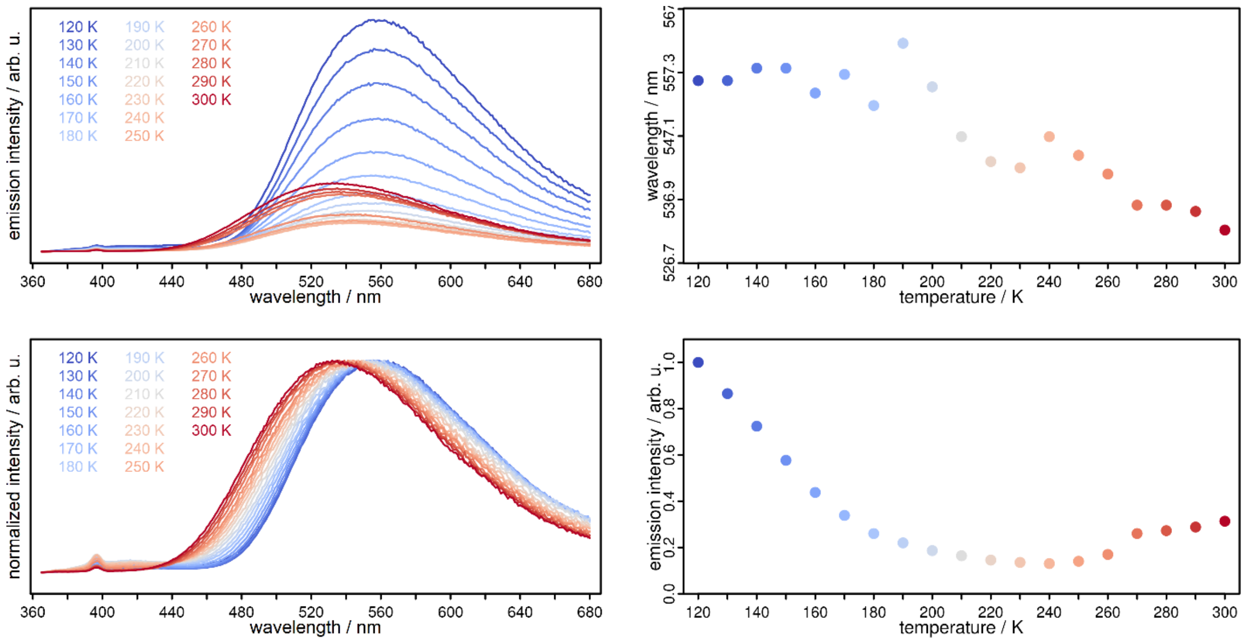

Figure S78. Temperature dependent emission spectra (top left), normalized temperature dependent emission spectra (bottom left), wavelength of emission maxima (top right) and emission intensity at different temperatures (bottom right) of **C5** in dry and inert 2-methyltetrahydrofuran.

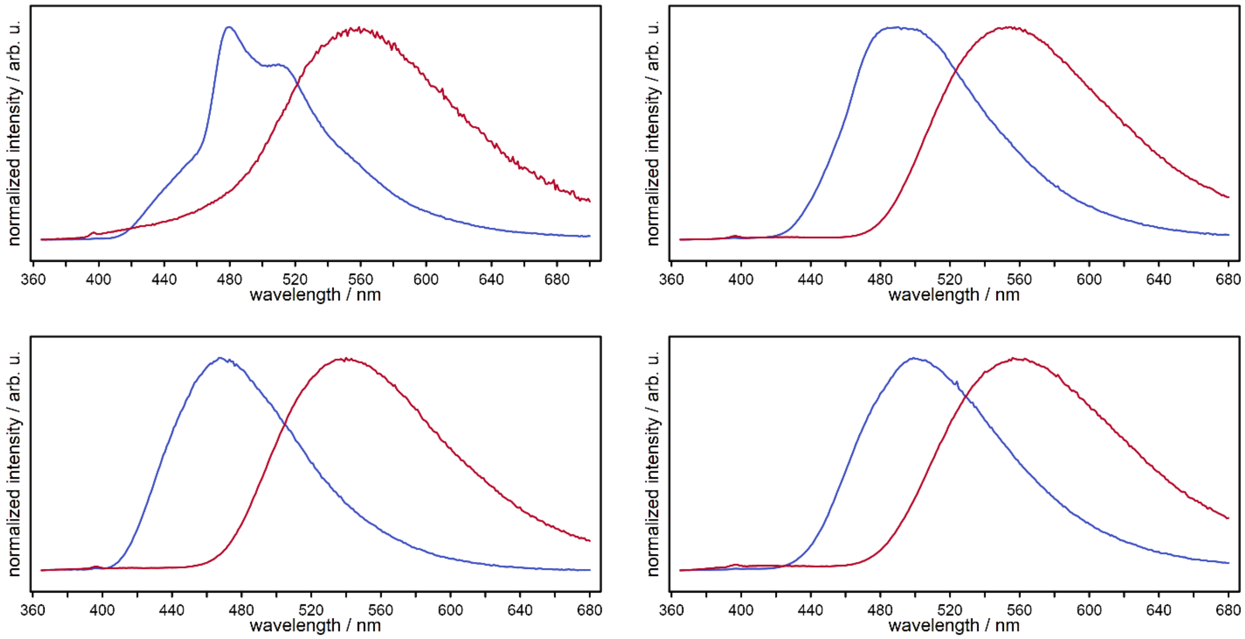

Figure S79. Normalized emission spectra at 80 K (blue) and 130 K (red) of **C2** (top left), **C3** (top right), **C4** (bottom left) and **C5** (bottom right) in dry and inert 2-methyltetrahydrofuran.

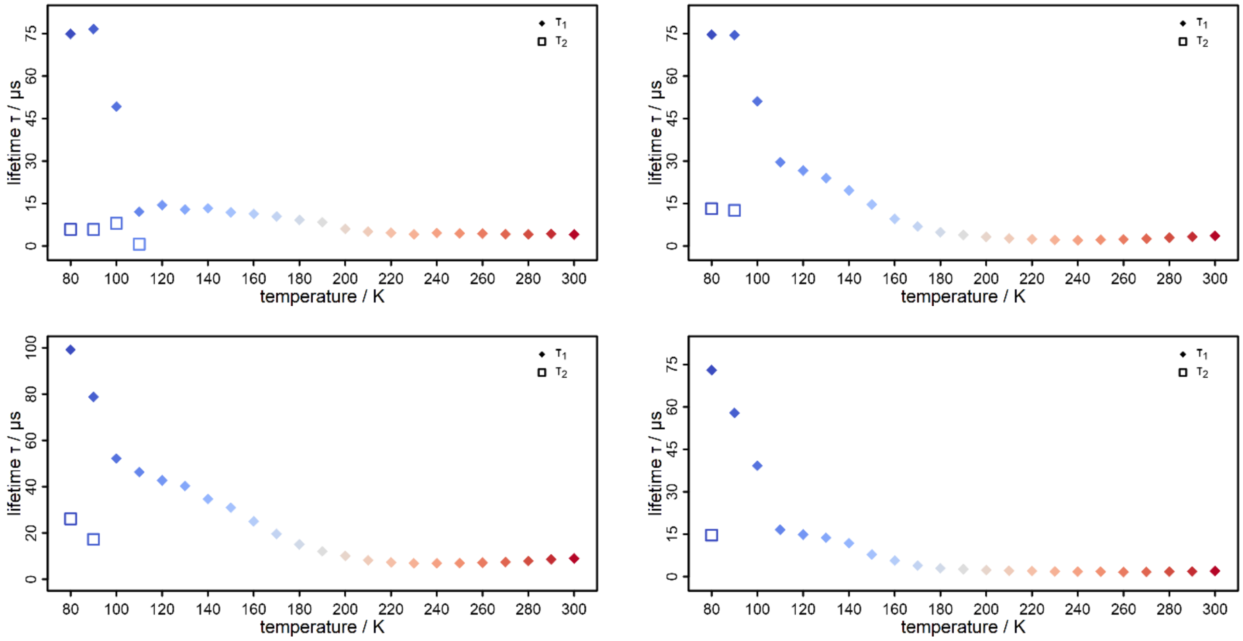

Figure S80. Excited-state lifetimes of **C2** (top left), **C3** (top right), **C4** (bottom left) and **C5** (bottom right) between 80 K (blue) and 300 K (red) in dry and inert 2-methyltetrahydrofuran.

To calculate the energy separations between the S_1 and T_1 states $\Delta\boldsymbol{E}_{\boldsymbol{S_1-T_1}}$, the observed, temperature dependent excited-state lifetimes $\boldsymbol{\tau(T)}$ between 300 K and 120 K were fitted to eq. S5 wherein $\boldsymbol{\tau(T_1)}$ denotes the triplet and $\boldsymbol{\tau(S_1)}$ the singlet decay time.[34–37]

$$\tau(\mathrm{T}) = \frac{3 + \exp\left[-\frac{\Delta E_{S_1-T_1}}{k_B T}\right]}{\frac{3}{\tau(T_1)} + \frac{1}{\tau(S_1)}\exp\left[-\frac{\Delta E_{S-T}}{k_B T}\right]} \qquad \text{(eq. S5)}$$

Table S18. Results of the fit of eq. S5 to the observed, temperature dependent excited-state lifetimes $\tau(T)$ between 300 K and 120 K shown in Figure S81.

Complex	$\Delta E_{S_1-T_1}$ / eV	$\tau(S_1)$ / ns	$\tau(T_1)$ / µs
**C2**	0.06 ± 0.01	110.8 ± 45.9	16.3 ± 1.5
**C3**	0.09 ± 0.01	10.0 ± 7.0	36.1 ± 5.0
**C4**	0.06 ± 0.01	129.8 ± 59.8	59.3 ± 9.2
**C5**	0.07 ± 0.01	15.7 ± 9.7	23.3 ± 4.9

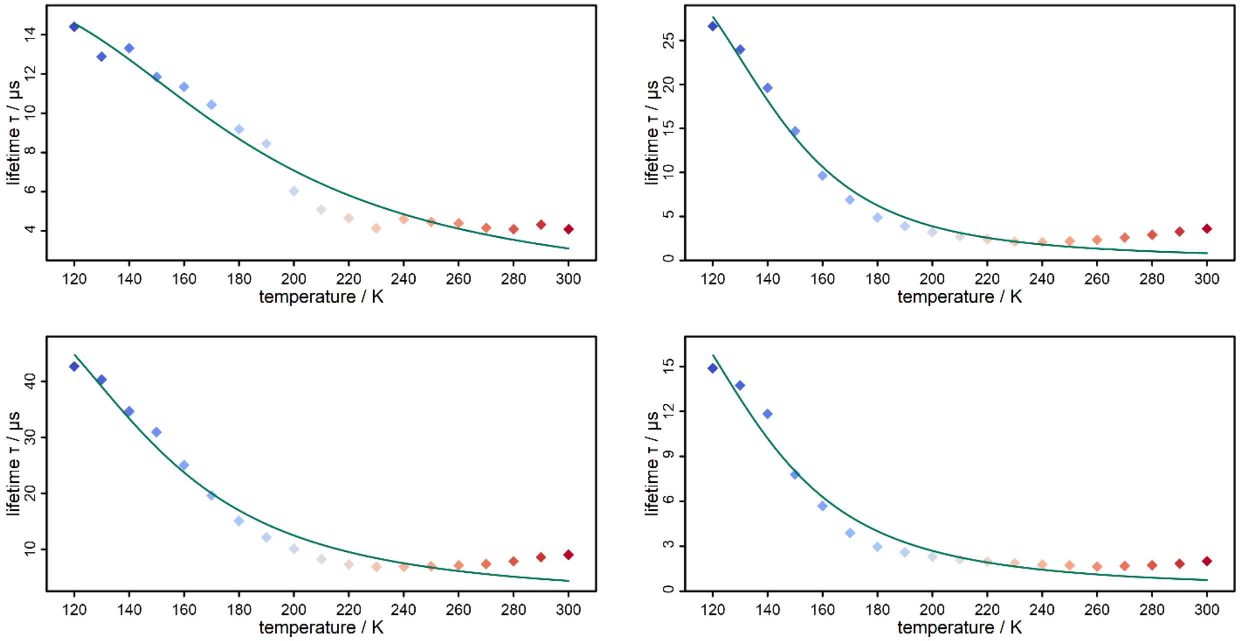

Figure S81. Excited-state lifetimes of **C2** (top left), **C3** (top right), **C4** (bottom left) and **C5** (bottom right) between 120 K (blue) and 300 K (red) in dry and inert 2-methyltetrahydrofuran. A Fit to eq. S5 is shown in dark green.

11 Determination of E^{00}

The zero-zero excitation energies (E^{00}) were estimated by a tangential fit of the steady-state emission at room temperature.

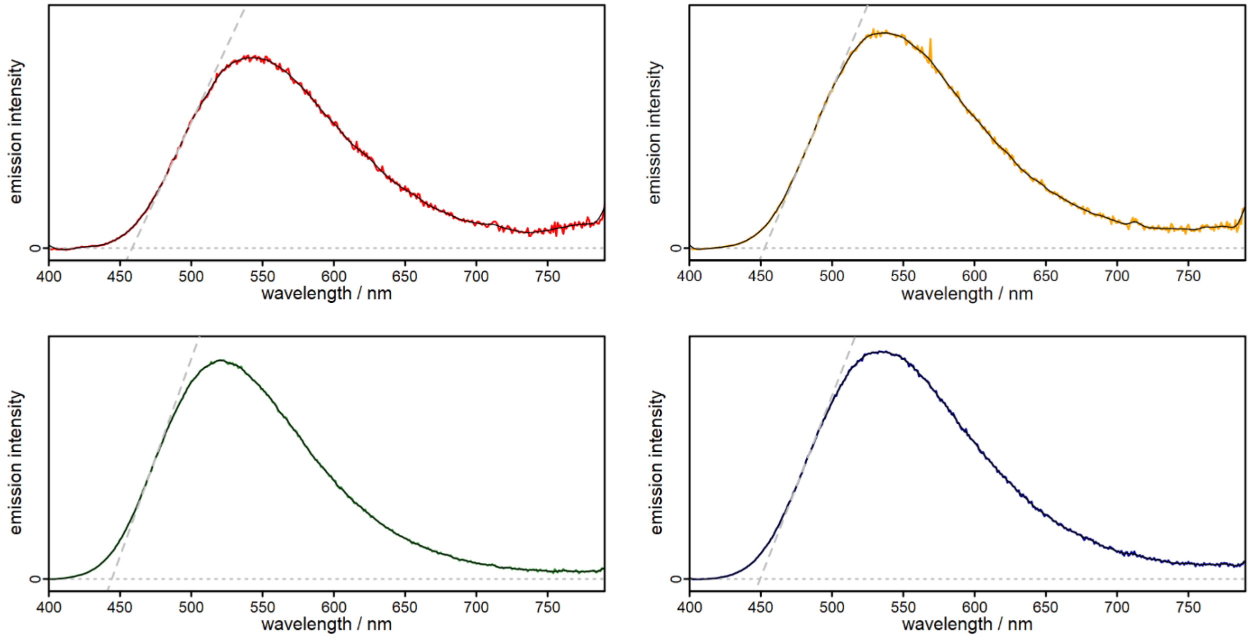

Figure S82. Determination of the zero-zero transition energy E^{00} by construction of a tangent on the emission onset for **C2** (top left, tangent at 490 nm, x-intercept = 458 nm), **C3** (top right, tangent at 485 nm, x-intercept = 453 nm), **C4** (bottom left, tangent at 472 nm, x-intercept = 444 nm) and **C5** (bottom right, tangent at 481 nm, x-intercept = 450 nm). The values for E^{00} were determined as the intercept with the x-axis.

Since the observed emission is proposed to originate from the singlet state *via* TADF at room temperature, the determined energies were attributed to the energies stored in the first excited singlet states (eq. S6).

$$E^{00} = E_{S_1} \qquad \text{(eq. S6)}$$

By subtracting the experimentally determined energy gaps ($\Delta E_{S_1\text{-}T_1}$, Table S18) from the former values, the triplet energies (E_{T_1}) were further estimated (eq. S7).

$$E_{T_1} = E_{S_1} - \Delta E_{S_1\text{-}T_1} \qquad \text{(eq. S7)}$$

12 Photostability

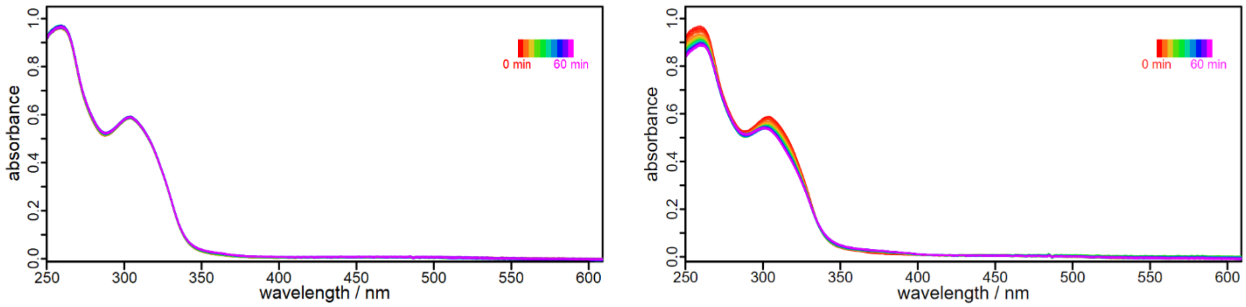

Figure S83. UV/vis absorption spectra of **C4** in tetrahydrofuran (inert) over a time course of 1 h without irradiation (left) and while being irradiated with a Xe-arc lamp (P = 121 W) equipped with a 280 nm cut-off filter (right).

Photostability measurements were carried out for the complex **C4**. While no detectable changes were observed when the sample was kept in dark, only minor spectral changes occurred upon irradiation. Although this may indicate a limited extent of photodegradation, these changes are minimal and the compound can be considered photostable under the applied conditions. A similar behavior is expected for the related complexes **C2**, **C3** and **C5**.

13 Stern-Volmer Quenching Experiments

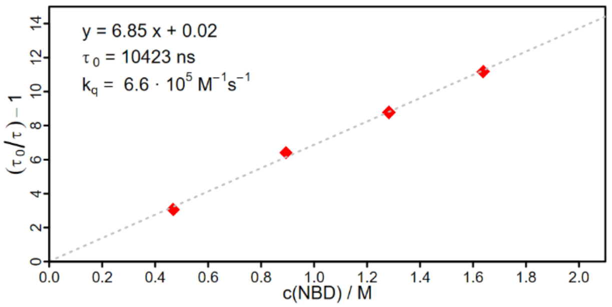

Figure S84. Stern-Volmer analysis of **C4** with increasing concentration of NBD in inert THF.

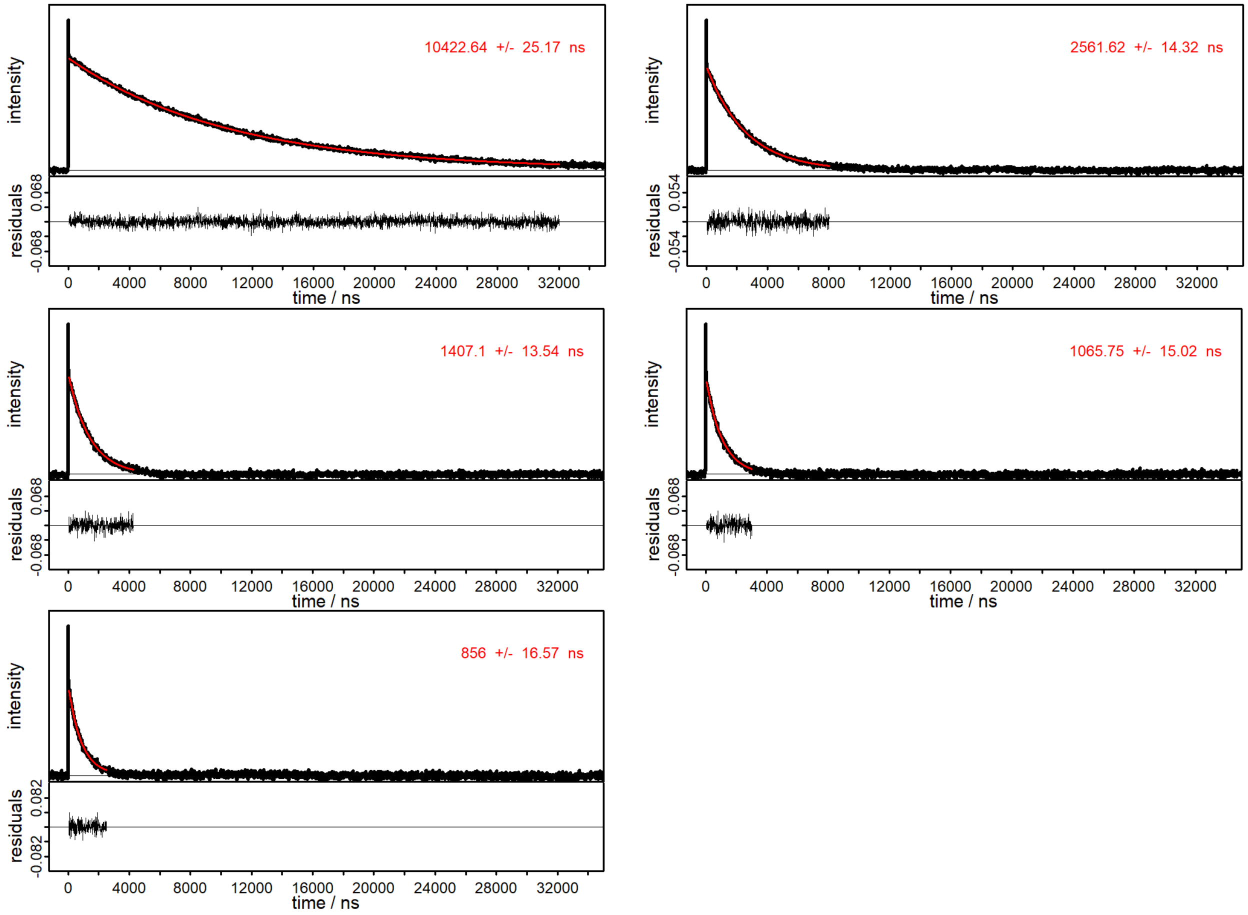

Figure S85. Emission decay curves of **C4** in inert THF in the absence of NBD and with increasing concentration of NBD (top right to bottom left).

THF was dried over Na/K, distilled under Ar atmosphere, stored over molecular sieves and filtrated over a Whatman™ glass microfiber filter before use. This solvent preparation different from the standard procedure described above is most likely the reason for the slightly longer emission lifetime observed.

14 Photocatalysis

General procedure.
Stock solution A (300 µL; contains **C2**-**5**, 0.54 µmol, 1 mol% in THF-d_8) and stock solution B (350 µL; contains NBD, 54 µmol, 1 eq.; $PhSiMe_3$, 27 µmol, 0.5 eq. in THF-d_8) were combined. The solution was irradiated (Figure S86) and NMR spectroscopic measurements were performed each 30 min or 60 min as detailed below.

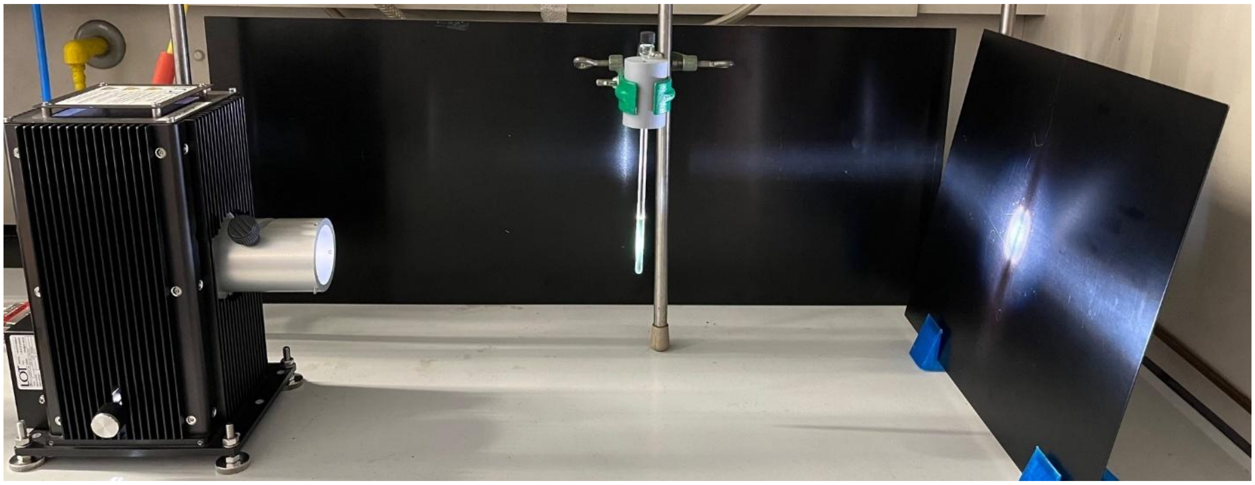

Figure S86. Irradiation setup showing the xenon arc lamp (left), the sample in an NMR tube fixated by a 3D-printed holder (middle) and the beam stop (right).

Spectroscopic investigations.
All spectra were automatically baseline and phase corrected. In the ^{1}H NMR spectrum recorded before irradiation the multiplet at 1.91-1.52 ppm of NBD was integrated to a value of 2. The area for the signal at 0.24 ppm corresponding to the internal standard $PhSiMe_3$ was determined. In the following monitoring of the same solution was this value set as constant and the changes of the signals corresponding to NBD and QC determined. The yield and conversion were determined by using the values obtained by integration *i* for the signals assigned by index *n* (eq. S8 and S9) as detailed in Figure S87. For NBD $i_n(0)$ is the value for of the integrals at 0 min and for QC the theoretical values of 2, 4, 2. The values of $i_n(t)$ are detailed in Figure S89 to S100. The obtained yield and conversion are summarized in Table S19.

$$yield = \frac{1}{3}\left(\frac{i_a(t) \times 100}{2} + \frac{i_b(t) \times 100}{4} + \frac{i_c(t) \times 100}{2}\right) \quad \text{(eq. S8)}$$

$$conversion = 100 - \left(\frac{1}{3} \times \sum_{n=1}^{3} \frac{i_n(t) \times 100}{i_n(0)}\right) \quad \text{(eq. S9)}$$

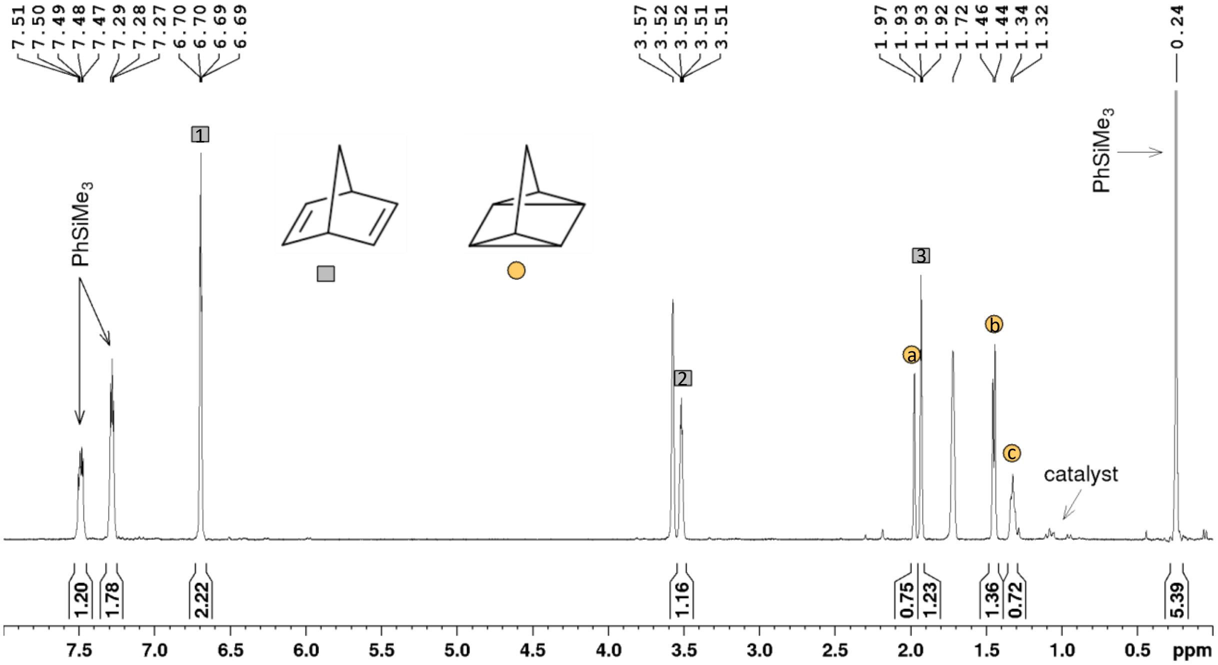

Figure S87. Detailed assignment of the signals in the ^{1}H NMR spectrum in THF-d_8 for the different molecules present in the catalysis after irradiating the solution of NBD with catalyst **C4** for 30 min with $PhSiMe_3$ as an internal standard.

Table S19. Determined yield and conversion for the catalytic transformation of NBD to QC calculated with eq. S8 and S9 using the values determined by integration as detailed in Figure S89 to S100.

	**0 min** yield (conversion)	**30 min** yield (conversion)	**60 min** yield (conversion)	**90 min** yield (conversion)	**120 min** yield (conversion)	**60 min dark**[b] yield (conversion)
/	0 % (0 %)	n. d.	7 % (7 %)	n. d.	12 % (11 %)	n. d.
**C4**[a]	0 % (0 %)	n. d.	0 % (0 %)	n. d.	0 % (0 %)	n. d.
**C2**	0 % (0 %)	n. d.	38 % (38 %)	n. d.	n. d.	n. d.
**C3**	0 % (0 %)	n. d.	26 % (26 %)	n. d.	n. d.	n. d.
**C4**	0 % (0 %)	35 % (37 %)	60 % (63 %)	76 % (79 %)	87 % (90 %)	86 % (90 %)
**C5**	0 % (0 %)	n. d.	65 % (66 %)	n. d.	n. d.	n. d.

n. d. = not determined. [a] Without irradiation; sample was kept in the dark. [b] Determined after 120 min of irradiation.

The signals for QC at 1.25-1.36 ppm and 1.95-2.00 ppm have a minimal overlap with neighboring signals resulting, if taken into account, for t = 0 min in a yield of 1 % (Figure S88). The stock solution was diluted to 1 % and the intensity of the signals determined to be more than six times higher than the baseline noise and well above the determination limit. At t = 0 min no peaks corresponding to QC can be found. Signals a and c (Figure S87) are thus omitted for the determination of the yield at t = 0 min. If any conversion took place, all signals of QC were considered for the determination of the yield.

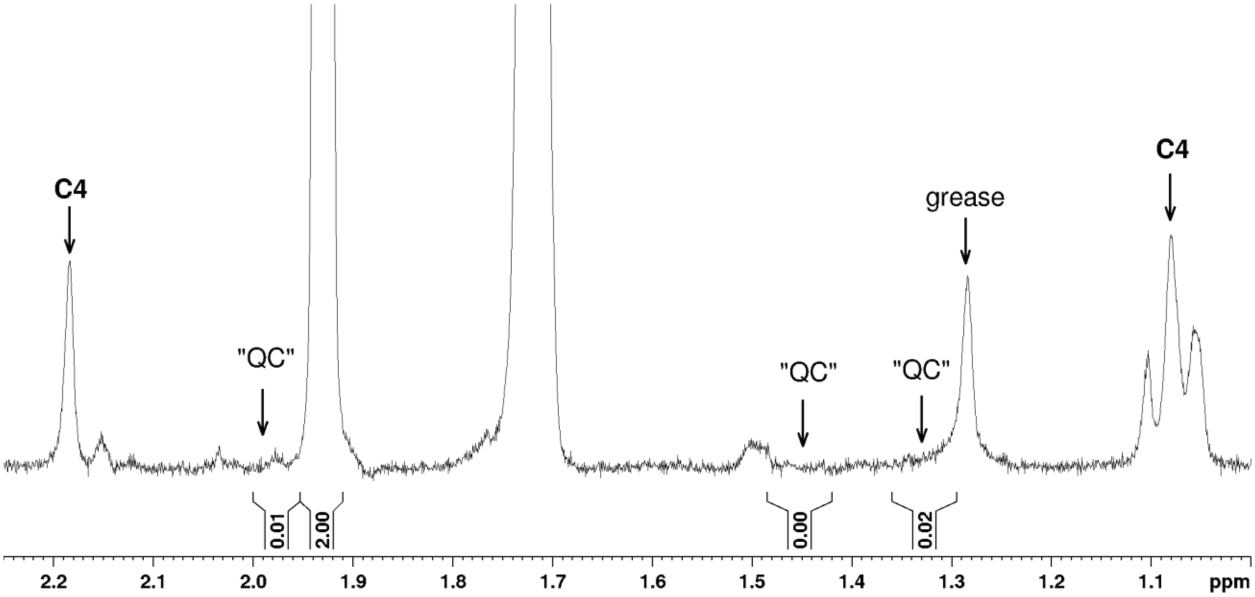

Figure S88. Zoom in on the ^{1}H NMR spectrum in THF-d_8 of a solution before irradiation containing **C4** showing the overlap of the areas integrated for QC with neighboring signals.

Recording one ^{1}H NMR spectrum with the chosen parameters took in total approximately 30 min. To exclude a conversion of QC to NBD the sample was monitored after the photocatalysis. If kept 1 h in the dark and even upon heating to 55 °C no changes in the spectrum were observed, proving the suitability of the chosen monitoring technique (Figure S93 and S94).

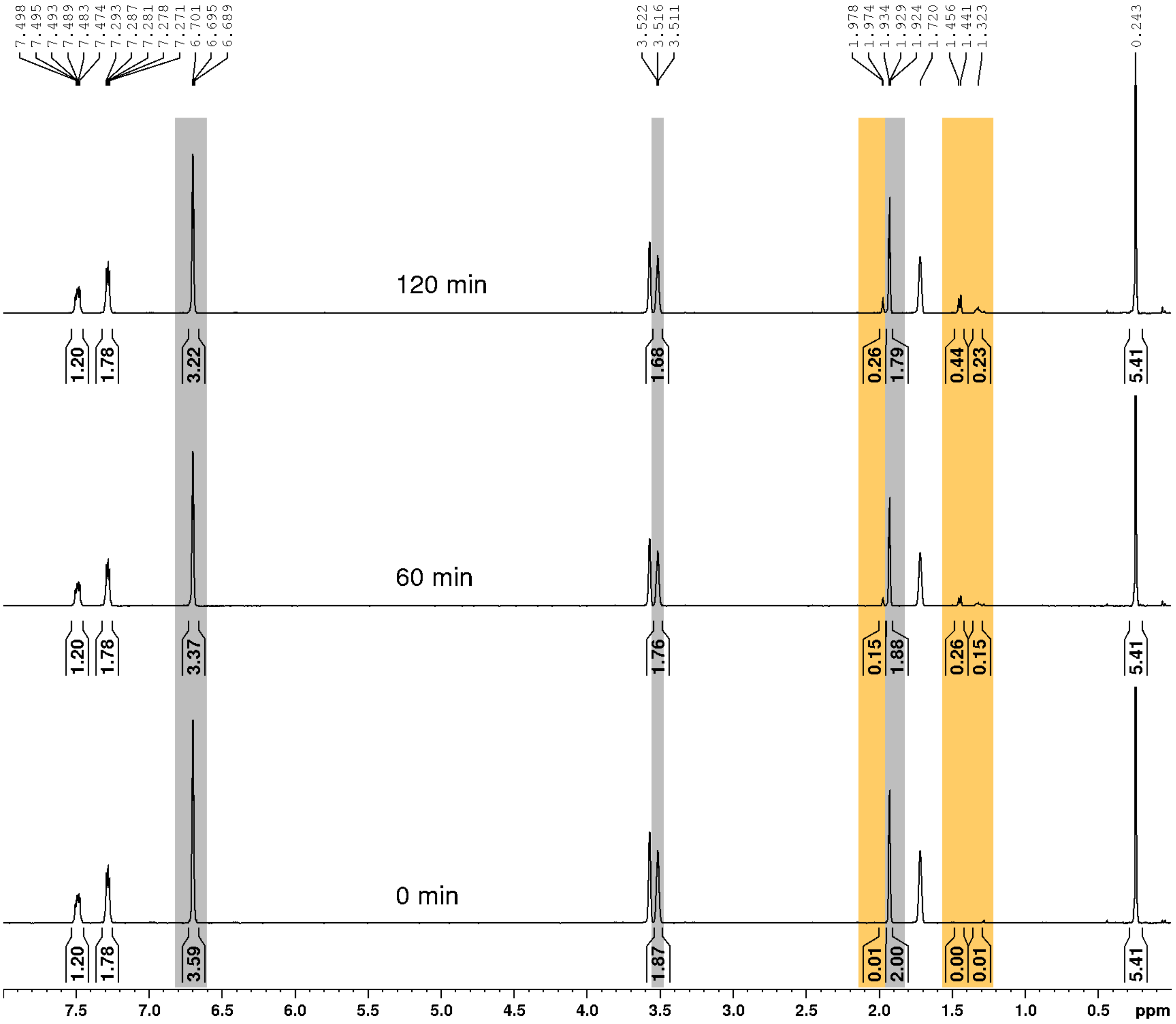

Figure S89. ^{1}H NMR spectroscopic monitoring for the catalytic conversion of NBD to QC without catalyst in THF-d_8.

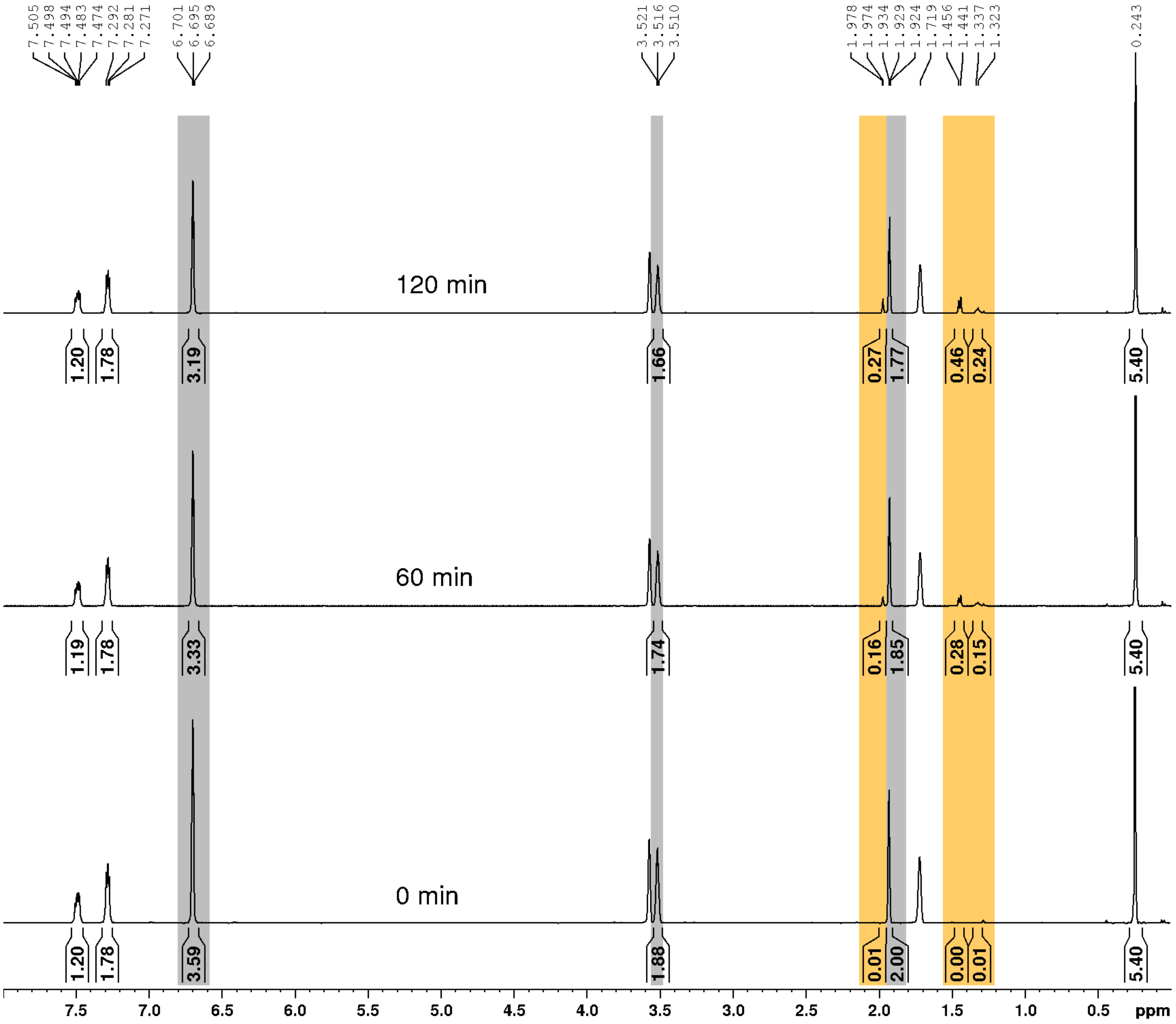

Figure S90. ^{1}H NMR spectroscopic monitoring for the catalytic conversion of NBD to QC without catalyst in THF-d_8 (duplicate).

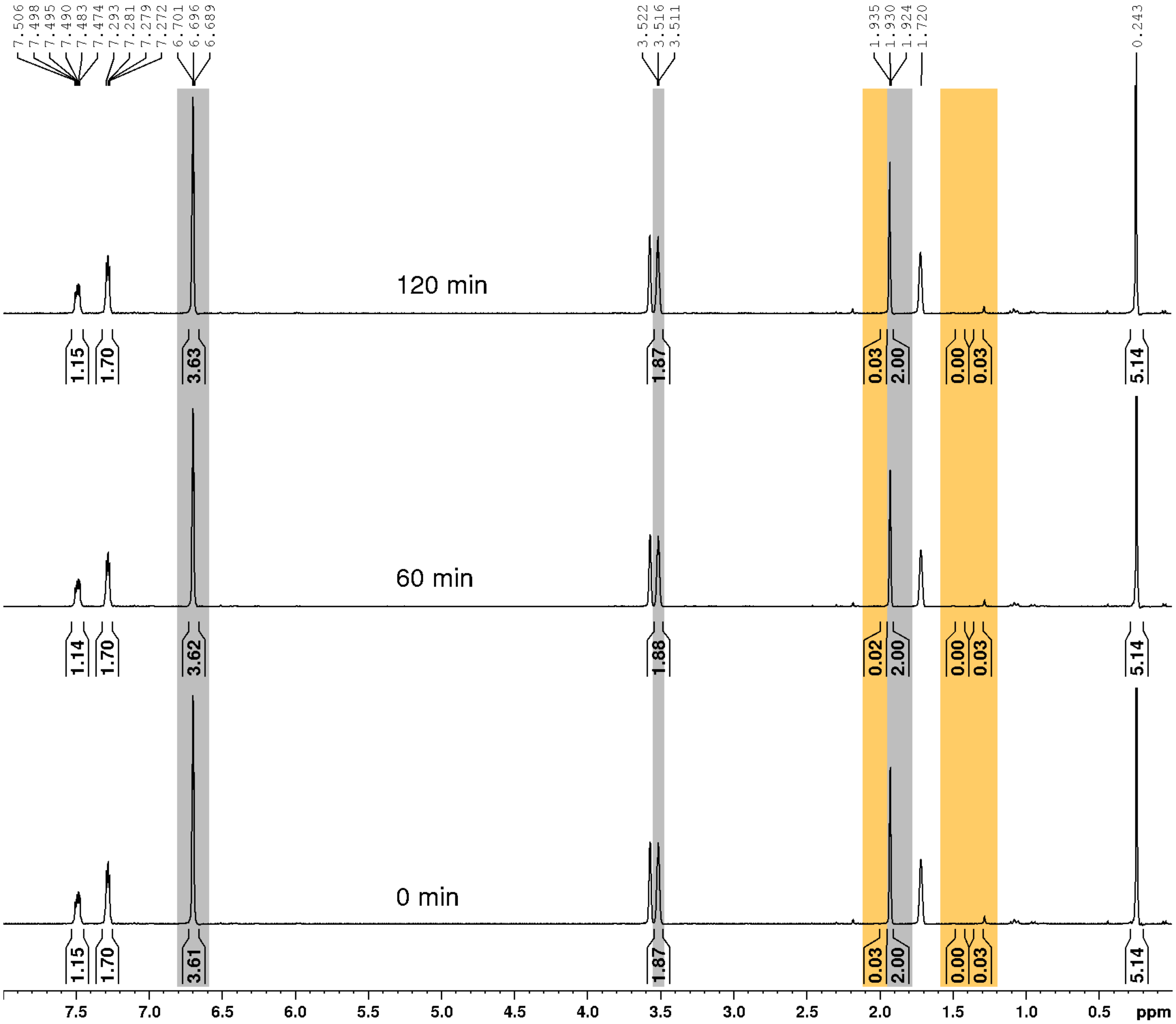

Figure S91. ^{1}H NMR spectroscopic monitoring for the catalytic conversion of NBD to QC using $B(C_6F_5)_3$-IDipp-Cu-4,4'Me_2Hdpa (**C4**) as a catalyst in THF-d_8 without irradiation.

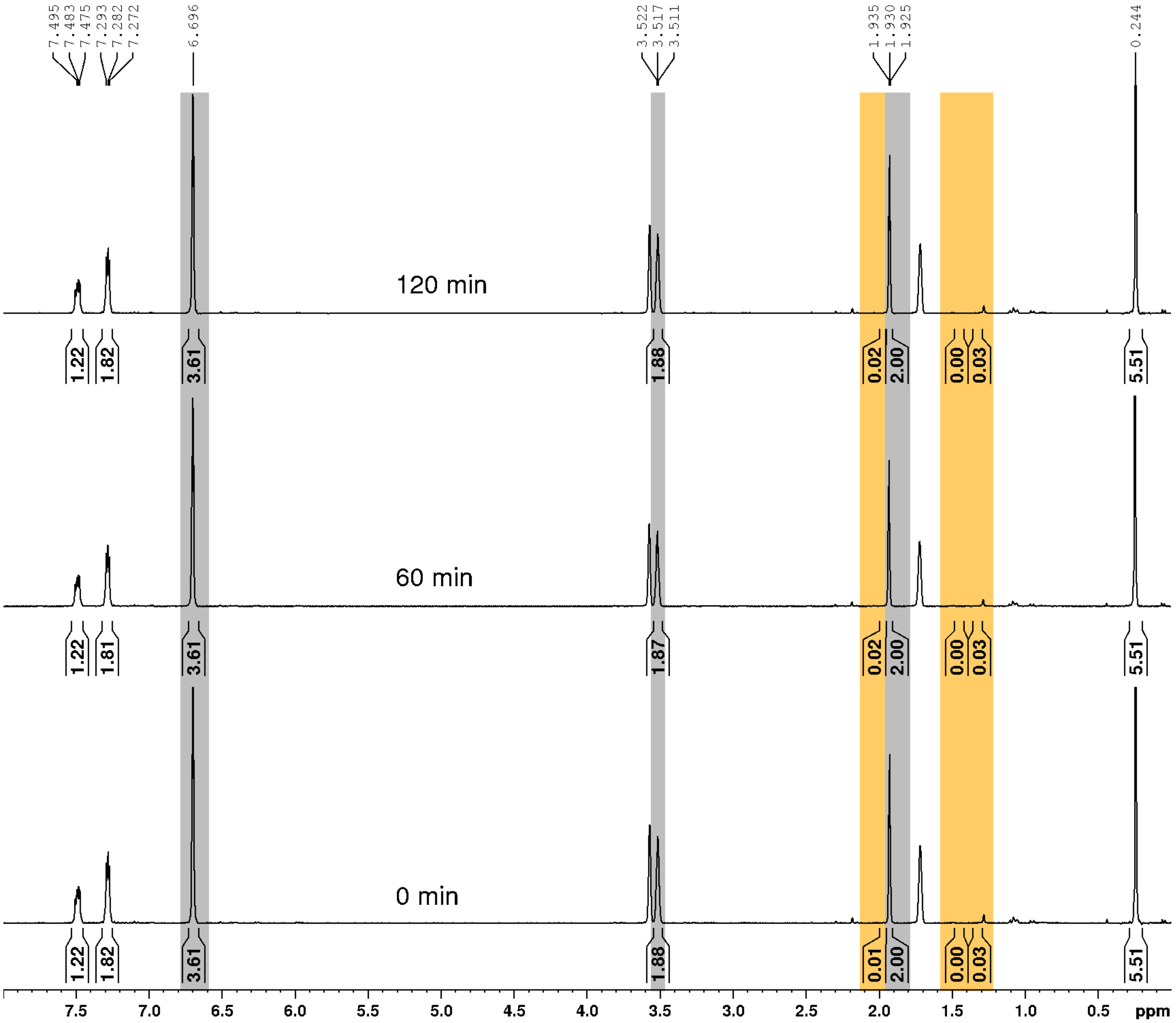

Figure S92. ^{1}H NMR spectroscopic monitoring for the catalytic conversion of NBD to QC using $B(C_6F_5)_3$-IDipp-Cu-4,4'Me_2Hdpa (**C4**) as a catalyst in THF-d_8 without irradiation (duplicate).

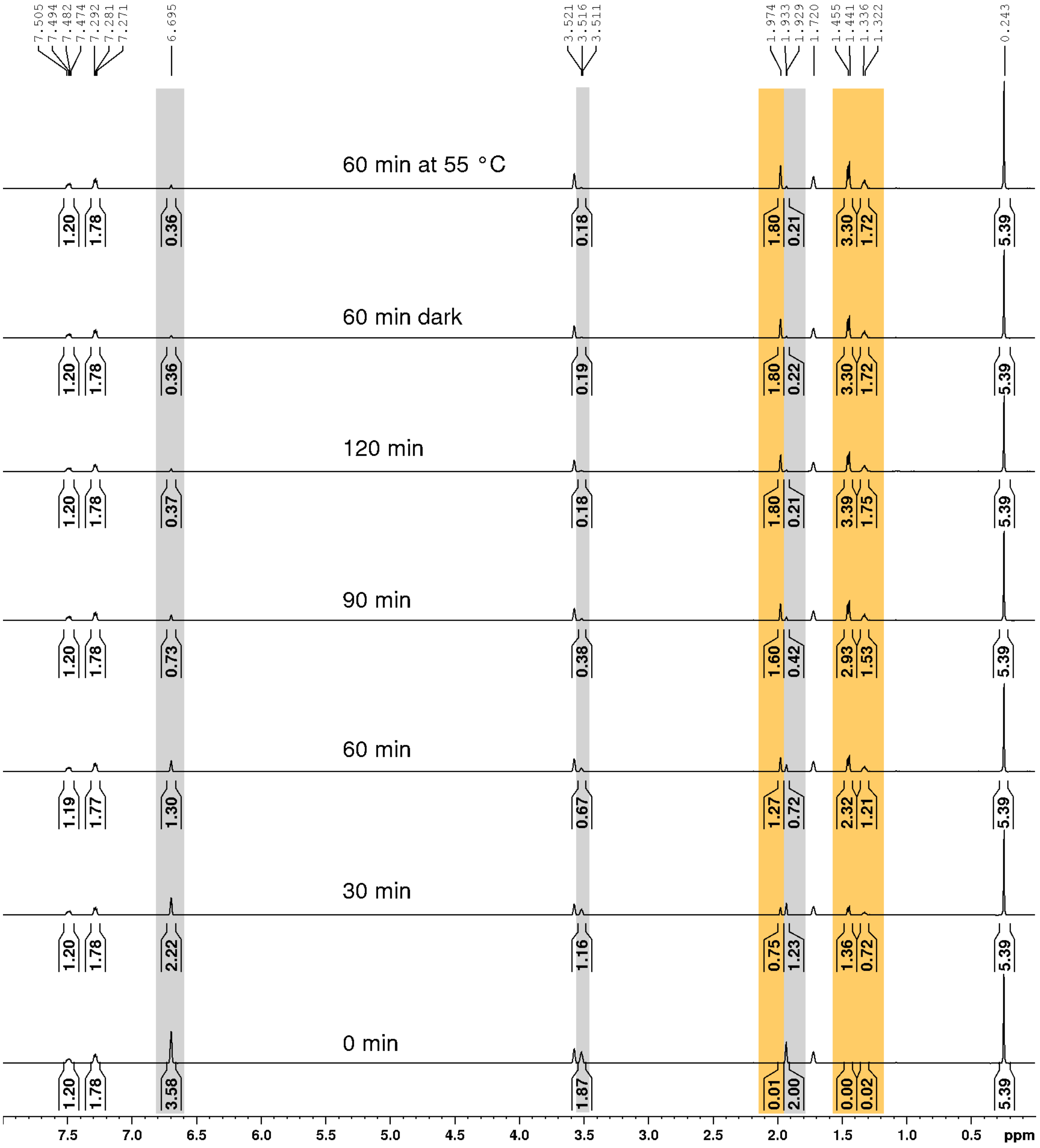

Figure S93. ^{1}H NMR spectroscopic monitoring for the catalytic conversion of NBD (grey) to QC (orange) using $B(C_6F_5)_3$-IDipp-Cu-4,4'Me_2Hdpa (**C4**) as a catalyst in THF-d_8.

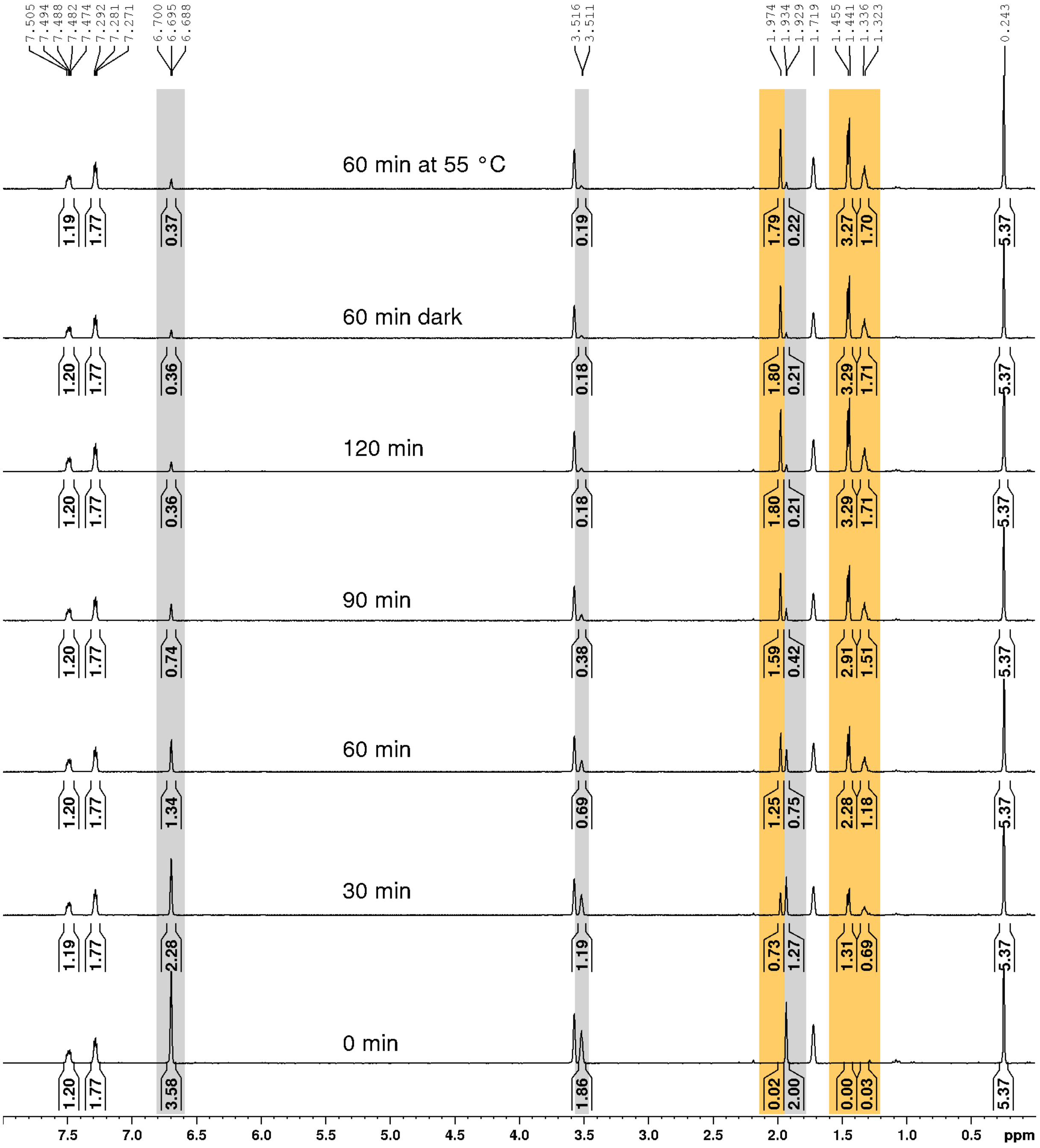

Figure S94. ^{1}H NMR spectroscopic monitoring for the catalytic conversion of NBD (grey) to QC (orange) using $B(C_6F_5)_3$-IDipp-Cu-4,4'Me_2Hdpa (**C4**) as a catalyst in THF-d_8 (duplicate).

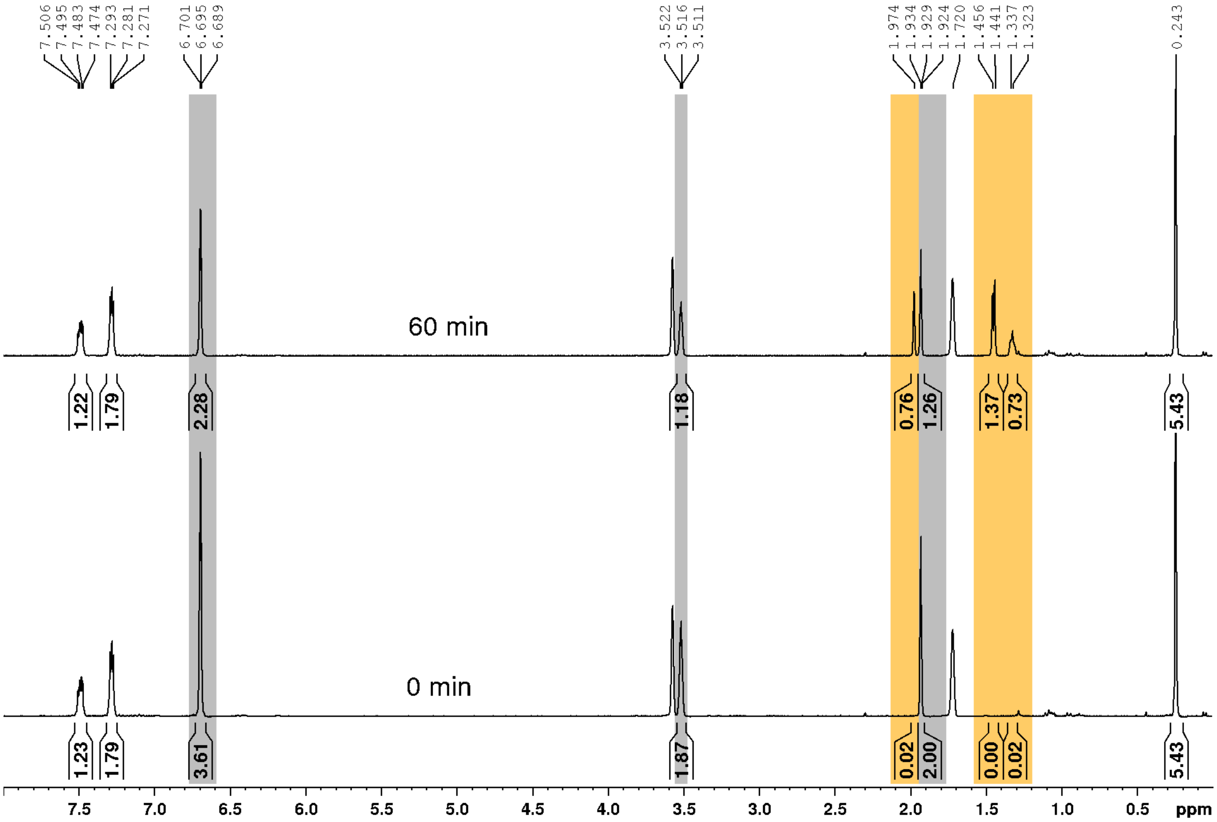

Figure S95. ^{1}H NMR spectroscopic monitoring for the catalytic conversion of NBD to QC using $B(C_6F_5)_3$-IDipp-Cu-Hdpa (**C2**) as a catalyst in THF-d_8.

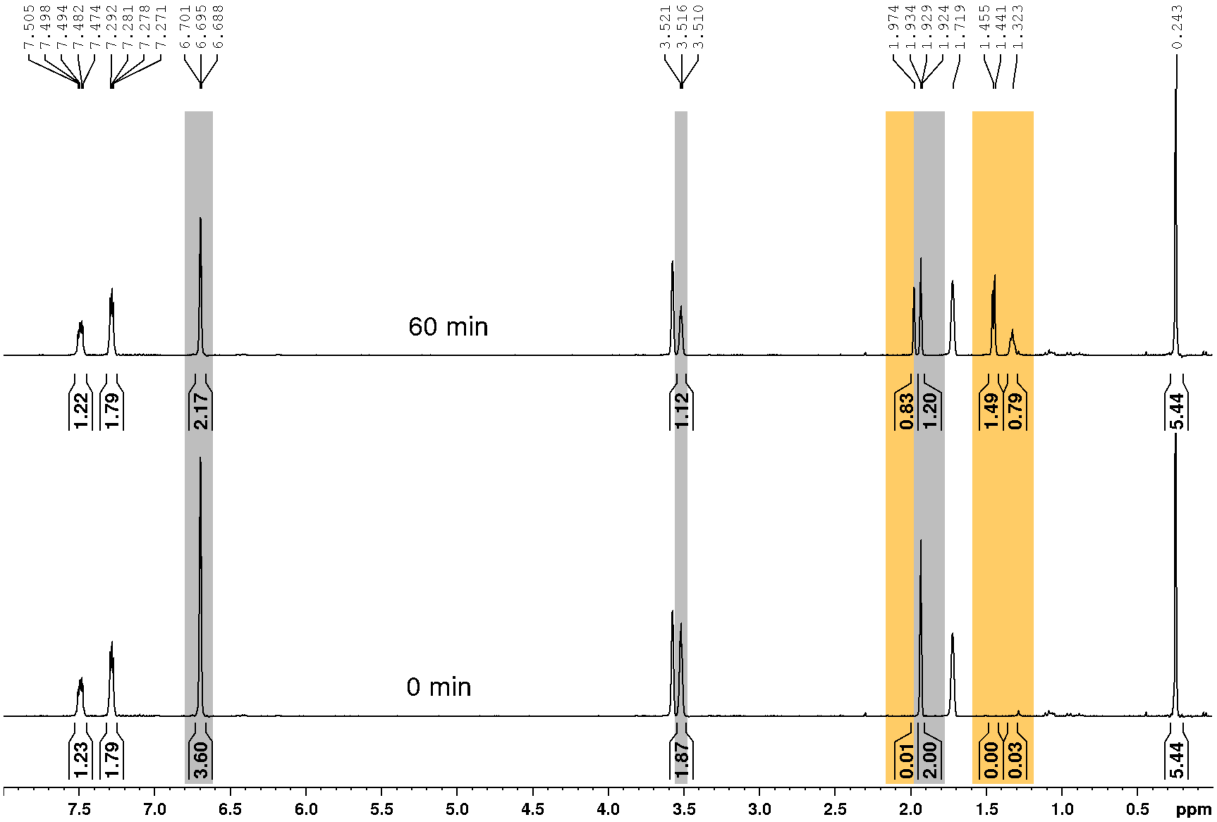

Figure S96. ^{1}H NMR spectroscopic monitoring for the catalytic conversion of NBD to QC using $B(C_6F_5)_3$-IDipp-Cu-Hdpa (**C2**) as a catalyst in THF-d_8 (duplicate).

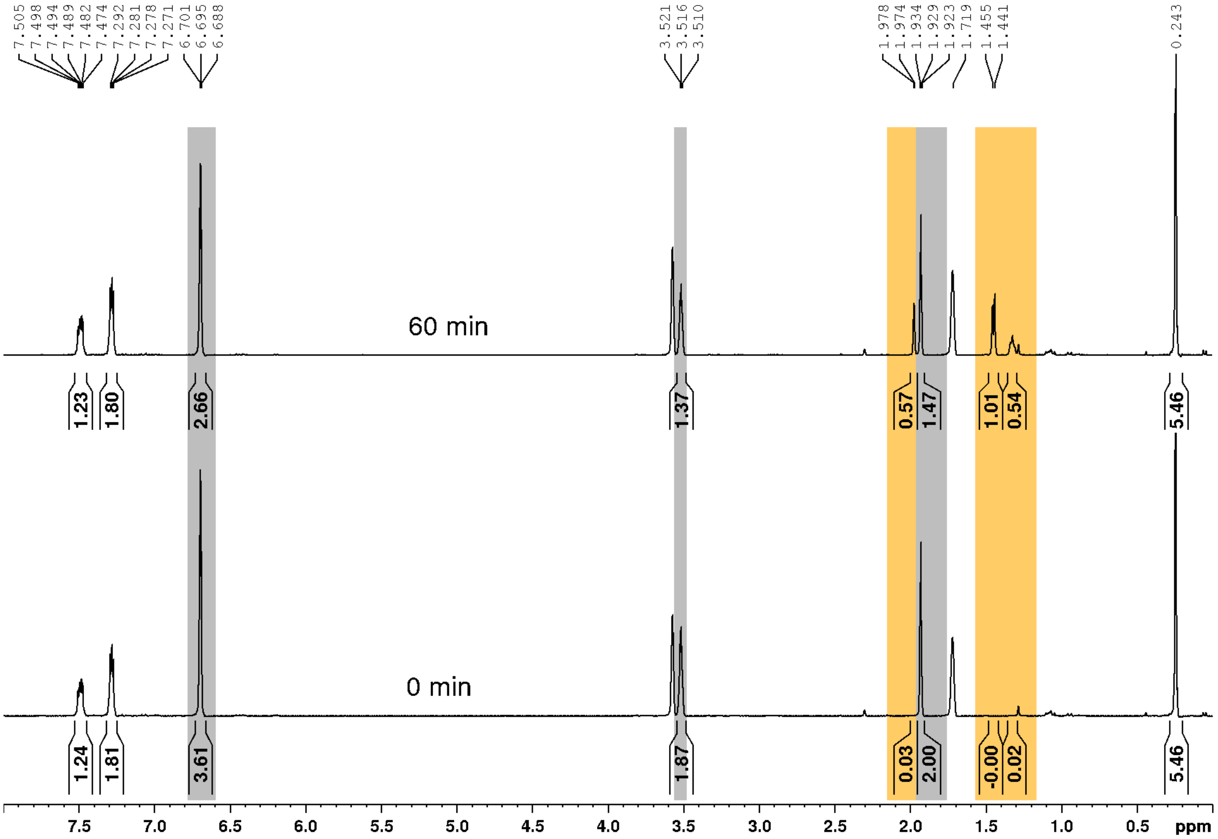

Figure S97. ^{1}H NMR spectroscopic monitoring for the catalytic conversion of NBD to QC using $B(C_6F_5)_3$-IDipp-Cu-3,3'Me_2Hdpa (**C3**) as a catalyst in THF-d_8.

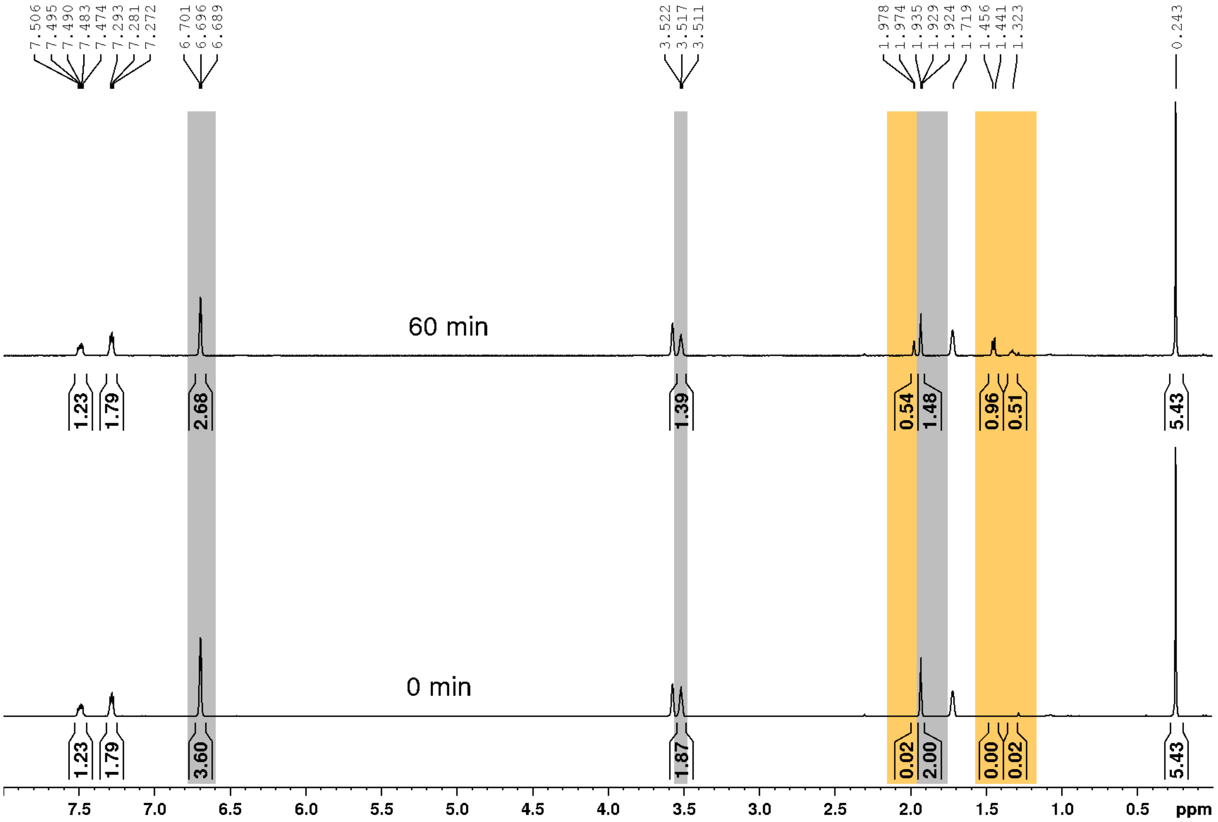

Figure S98. ^{1}H NMR spectroscopic monitoring for the catalytic conversion of NBD to QC using $B(C_6F_5)_3$-IDipp-Cu-3,3'Me_2Hdpa (**C3**) as a catalyst in THF-d_8 (duplicate).

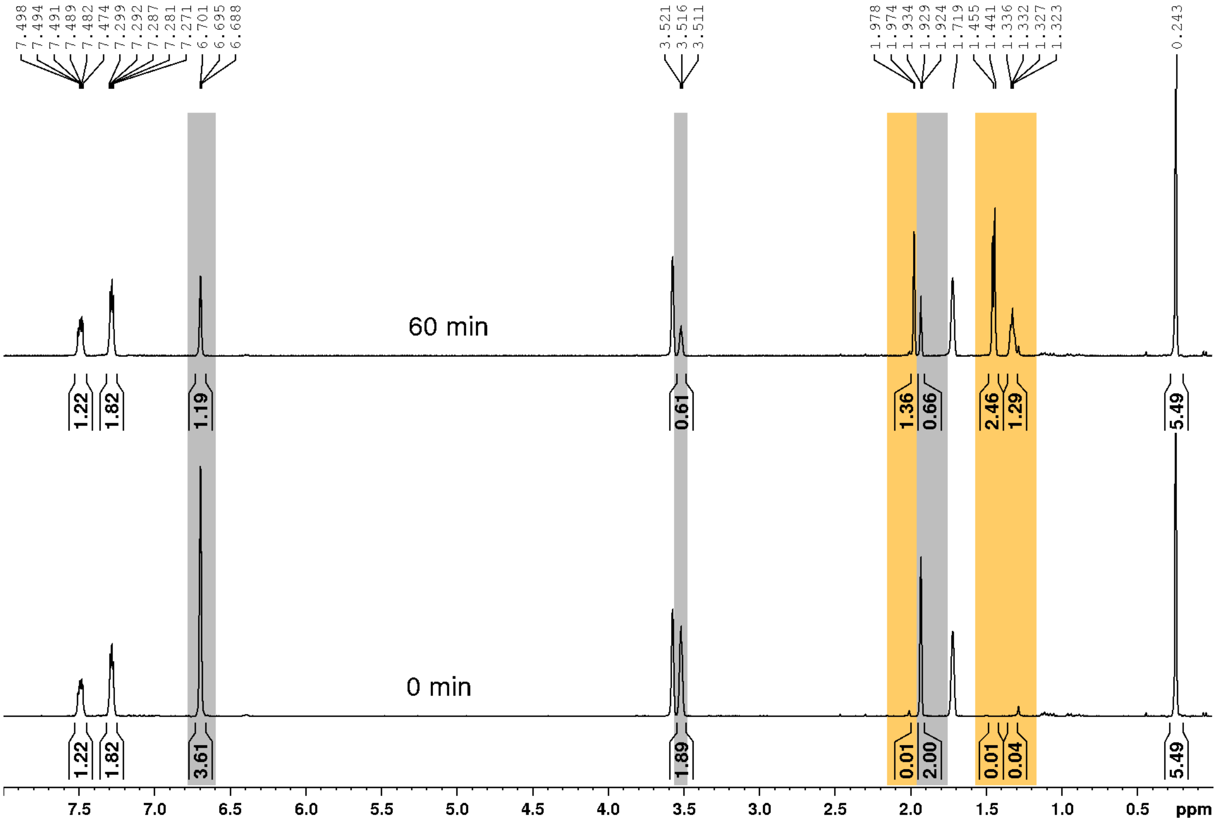

Figure S99. ^{1}H NMR spectroscopic monitoring for the catalytic conversion of NBD to QC using $B(C_6F_5)_3$-IDipp-Cu-5,5'Me_2Hdpa (**C5**) as a catalyst in THF-d_8.

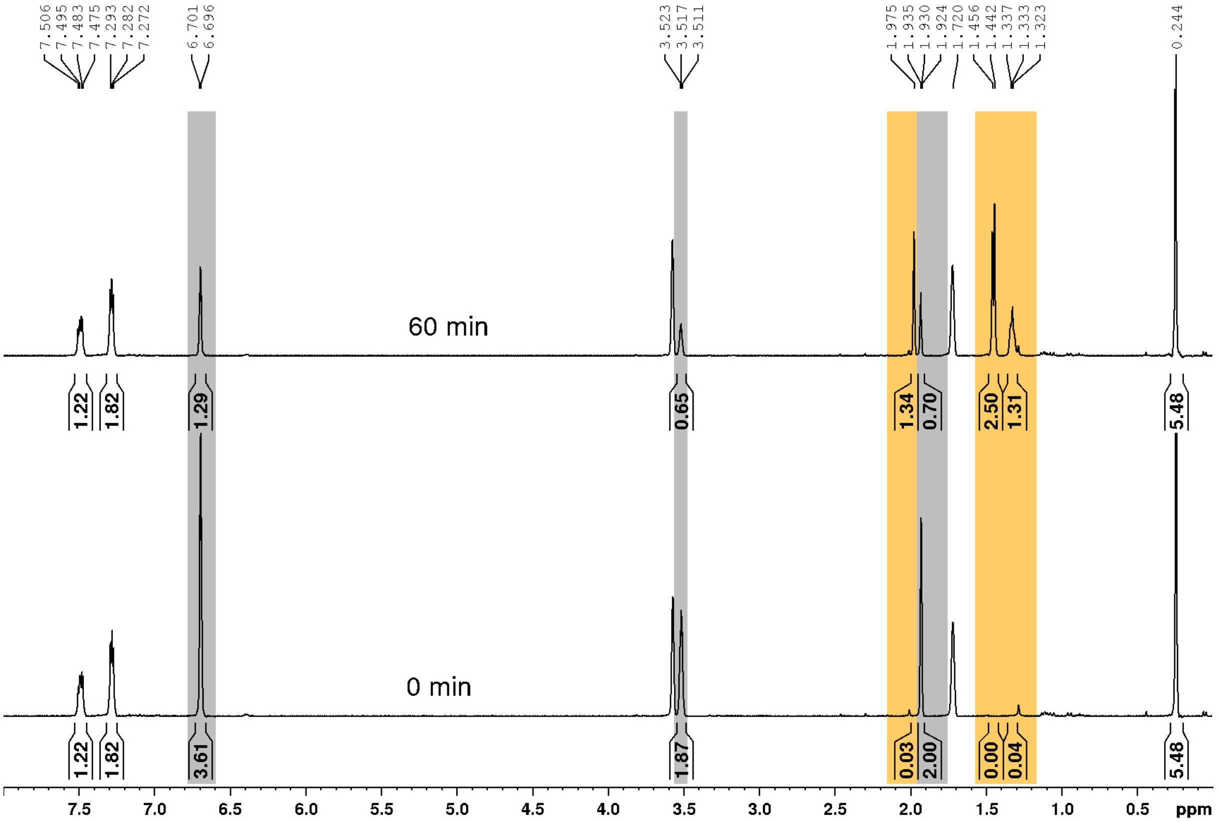

Figure S100. ^{1}H NMR spectroscopic monitoring for the catalytic conversion of NBD to QC using $B(C_6F_5)_3$-IDipp-Cu-5,5'Me_2Hdpa (**C5**) as a catalyst in THF-d_8 (duplicate).

To determine how complexes **C2**-**C5** perform in comparison to established Ir-based catalysts, experiments using Ir(dFppy)$_3$ (tris[3,5-difluoro-2-(2-pyridinyl)phenyl]-iridium) were performed. Stock solutions containing Ir(dFppy)$_3$ (c = 1.81 mmol/L) and NBD (c = 154 mmol/L) were combined and if necessary diluted with THF-d_8 to reach a total volume of 0.65 mL to obtain final NBD concentrations of 83 mmol/L, 42 mmol/L or 10 mmol/L and 1 mol% of Ir(dFppy)$_3$. PhSiMe$_3$ (2 mg, 14 µmol, 0.25 eq.) was added as an internal standard. The solution was irradiated and NMR spectroscopic measurements were performed after 5 min, 30 min or 60 min as detailed below. Due to the relatively low selectivity of the sensitizer under standard conditions, and the absence of observable side products in the ^{1}H NMR spectra, control experiments at lower concentration were carried out with single determination. The overall selectivity was found to increase when catalytic experiments were performed under diluted conditions, which may be indicative of polymerization side reactions at higher concentrations.

Table S20. Determined yield and conversion for the catalytic transformation of NBD to QC calculated with eq. S8 and S9 using the values determined by integration as detailed in Figure S101 to S105.

NBD conc. in mmol/L	**0 min** yield (conversion)	**5 min** yield (conversion)	**30 min** yield (conversion)	**60 min** yield (conversion)
83	0 % (0 %)	27 % (44 %)	74 % (96 %)	76 % (99 %)
83[a]	0 % (0 %)	n. d.	0 % (0 %)	n. d.
42	0 % (0 %)	n. d.	91 % (94 %)	n. d.
10	0 % (0 %)	n. d.	80 % (81 %)	n. d.

n. d. = not determined. [a] Without irradiation; sample was kept in the dark.

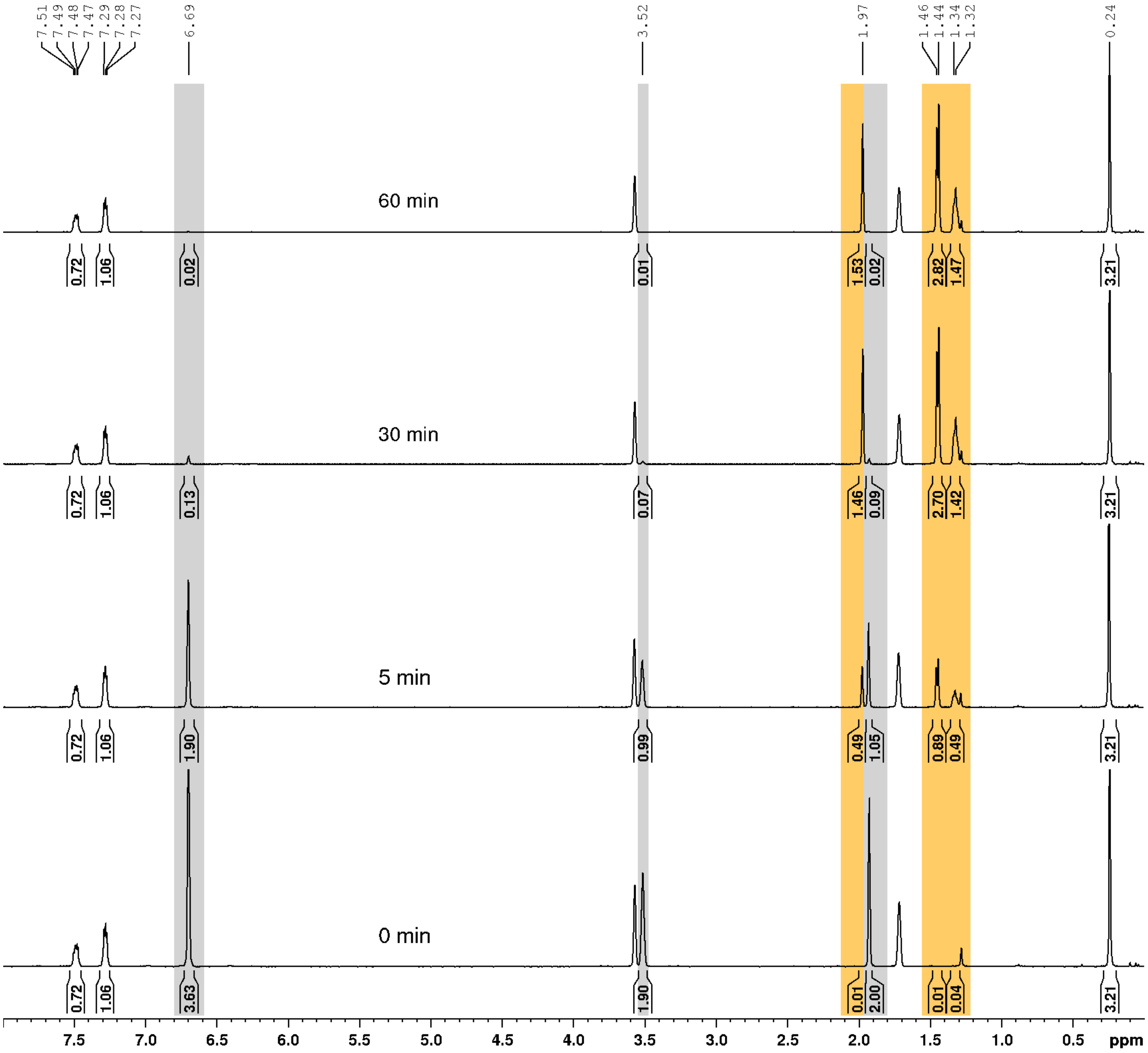

Figure S101. ^{1}H NMR spectroscopic monitoring for the catalytic conversion of NBD (c = 83 mmol/L) to QC using Ir(dFppy)$_3$ (1 mol%) as a catalyst in THF-d_8.

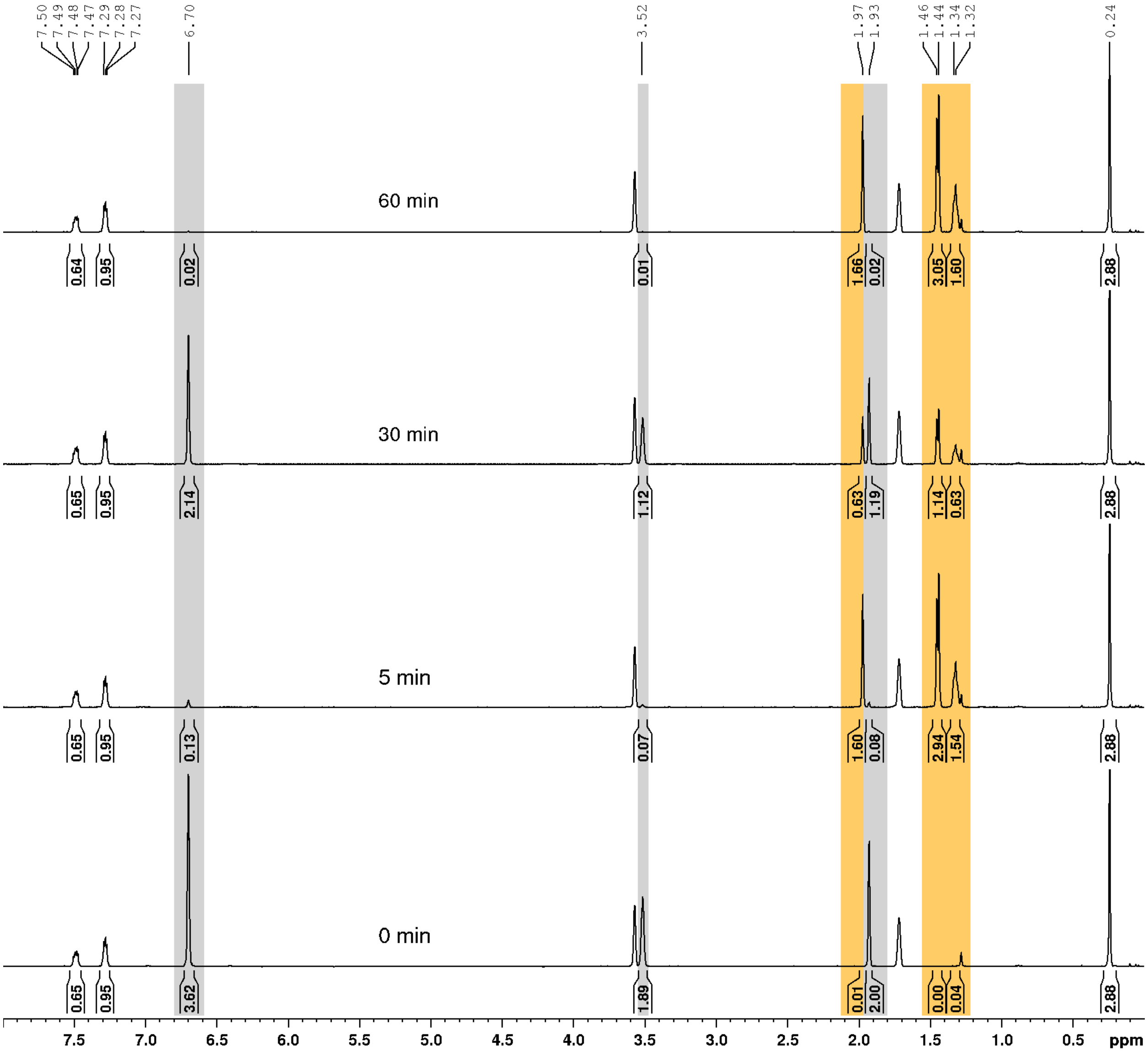

Figure S102. ^{1}H NMR spectroscopic monitoring for the catalytic conversion of NBD (c = 83 mmol/L) to QC using Ir(dFppy)$_3$ (1 mol%) as a catalyst in THF-d_8 (duplicate).

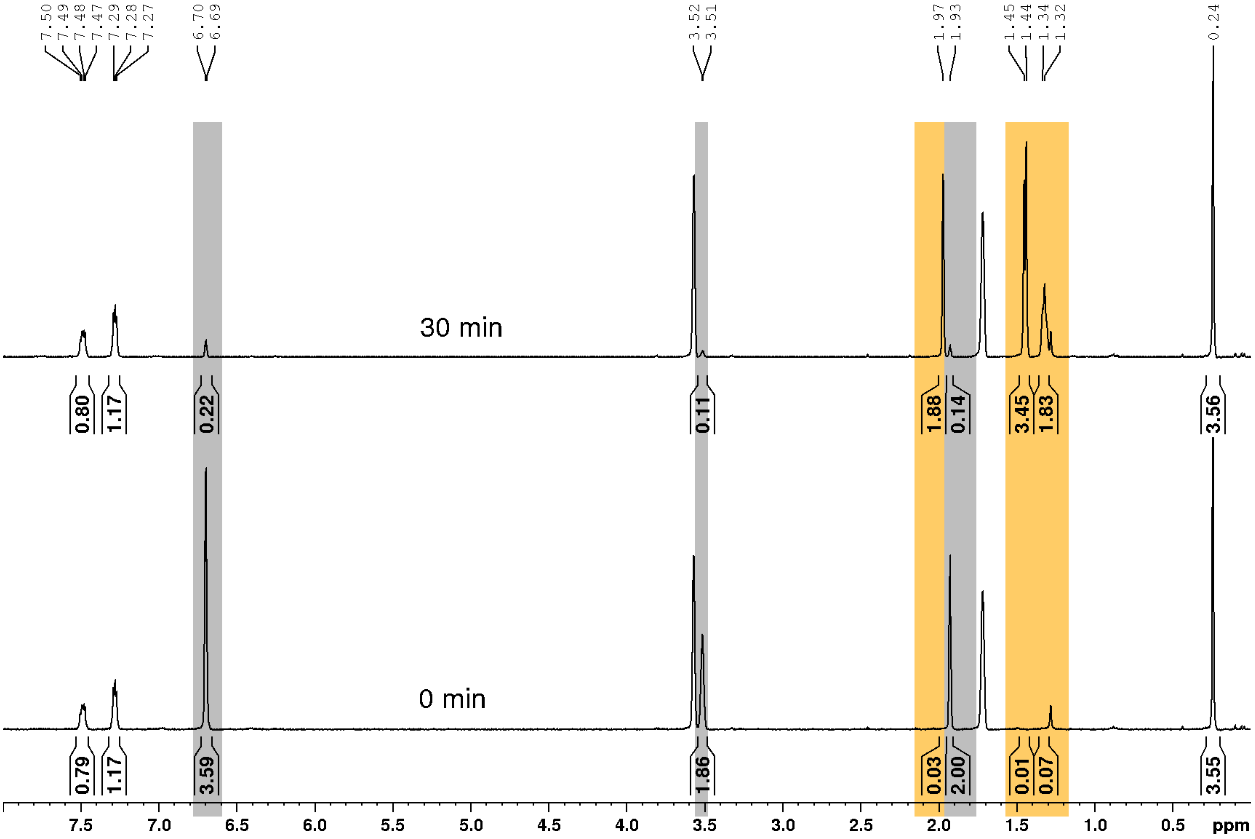

Figure S103. ^{1}H NMR spectroscopic monitoring for the catalytic conversion of NBD (c = 42 mmol/L) to QC using Ir(dFppy)$_3$ (1 mol%) as a catalyst in THF-d_8.

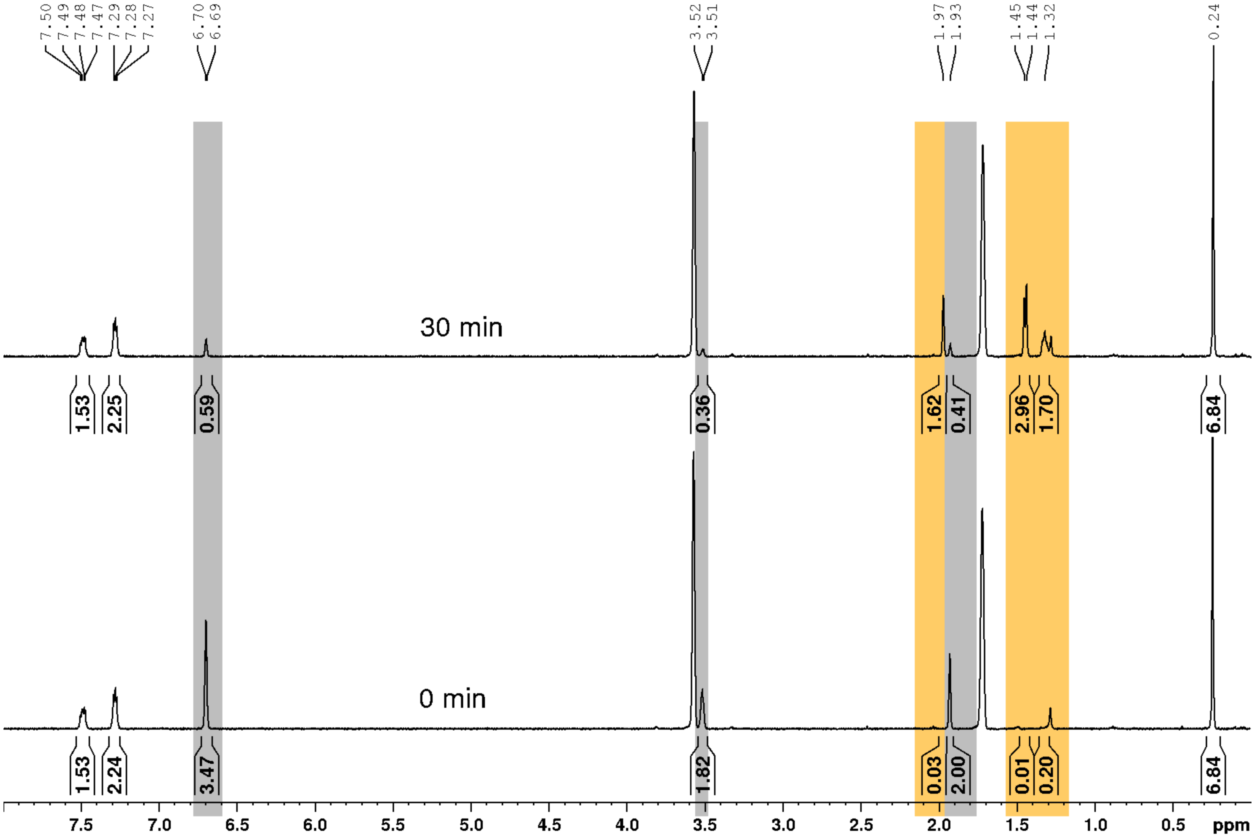

Figure S104. ^{1}H NMR spectroscopic monitoring for the catalytic conversion of NBD (c = 10 mmol/L) to QC using Ir(dFppy)$_3$ (1 mol%) as a catalyst in THF-d_8.

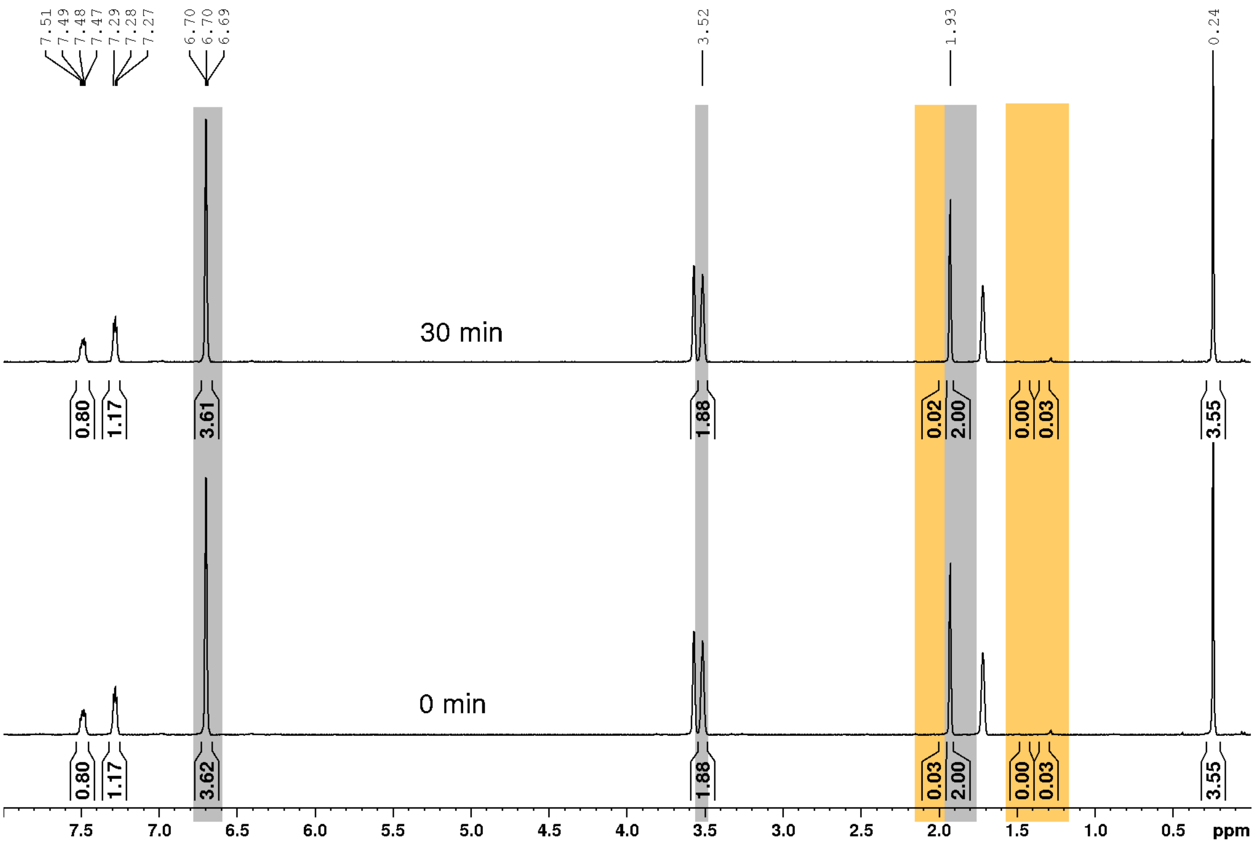

Figure S105. ^{1}H NMR spectroscopic monitoring for the catalytic conversion of NBD (c = 83 mmol/L) to QC using Ir(dFppy)$_3$ (1 mol%) as a catalyst in THF-d_8 without irradiation.

15 References

(1) Rigaku Oxford Diffraction, CrysAlisPRO Software System, version 1.171.42.64a and 1.171.42.101a 2023, Rigaku Corporation, Oxford, UK.

(2) Sheldrick, G. M. SHELXT - Integrated space-group and crystal-structure determination. *Acta Cryst.* **2015**, *A71*, 3–8.

(3) Sheldrick, G. M. Crystal structure refinement with SHELXL. *Acta Cryst.* **2015**, *C71*, 3–8.

(4) Dolomanov, O. V.; Bourhis, L. J.; Gildea, R. J.; Howard, J. A. K.; Puschmann, H. OLEX2: a complete structure solution, refinement and analysis program. *J. Appl. Crystallogr.* **2009**, *42*, 339–341.

(5) Kronig, S.; Theuergarten, E.; Daniliuc, C. G.; Jones, P. G.; Tamm, M. Anionic N-Heterocyclic Carbenes That Contain a Weakly Coordinating Borate Moiety. *Angew. Chem. Int. Ed.* **2012**, *51*, 3240–3244.

(6) Marion, R.; Sguerra, F.; Di Meo, F.; Sauvageot, E.; Lohier, J.-F.; Daniellou, R.; Renaud, J.-L.; Linares, M.; Hamel, M.; Gaillard, S. NHC Copper(I) Complexes Bearing Dipyridylamine Ligands: Synthesis, Structural, and Photoluminescent studies. *Inorg. Chem.* **2014**, *53*, 9181–9191.

(7) Planer, S.; Frosch, J.; Koneczny, M.; Trzybiński, D.; Woźniak, K.; Grela, K.; Tamm, M. Heterobimetallic Coinage Metal-Ruthenium Complexes Supported by Anionic N-Heterocyclic Carbenes. *Chem. Eur. J.* **2021**, *27*, 15218–15226.

(8) Neese, F. The ORCA program system. *WIREs Comput. Mol. Sci.* **2012**, *2*, 73–78.

(9) Neese, F. Software update: The ORCA program system—Version 5.0. *WIREs Comput. Mol. Sci.* **2022**, *12*, e1606.

(10) Adamo, C.; Barone, V. Toward reliable density functional methods without adjustable parameters: The PBE0 model. *J. Chem. Phys.* **1999**, *110*, 6158–6170.

(11) Weigend, F.; Ahlrichs, R. Balanced basis sets of split valence, triple zeta valence and quadruple zeta valence quality for H to Rn: Design and assessment of accuracy. *Phys. Chem. Chem. Phys.* **2005**, *7*, 3297–3305.

(12) Vahtras, O.; Almlöf, J.; Feyereisen, M. W. Integral approximations for LCAO-SCF calculations. *Chem. Phys. Lett.* **1993**, *213*, 514–518.

(13) Neese, F. An Improvement of the Resolution of the Identity Approximation for the Formation of the Coulomb Matrix. *J. Comput. Chem.* **2003**, *24*, 1740–1747.

(14) Neese, F.; Wennmohs, F.; Hansen, A.; Becker, U. Efficient, approximate and parallel Hartree–Fock and hybrid DFT calculations. A ‘chain-of-spheres’ algorithm for the Hartree–Fock exchange. *Chem. Phys.* **2009**, *356*, 98–109.

(15) Weigend, F. Accurate Coulomb-fitting basis sets for H to Rn. *Phys. Chem. Chem. Phys.* **2006**, *8*, 1057–1065.

(16) Bursch, M.; Mewes, J.-M.; Hansen, A.; Grimme, S. Best-Practice DFT Protocols for Basic Molecular Computational Chemistry. *Angew. Chem. Int. Ed.* **2022**, *61*, e202205735.

(17) Grimme, S.; Antony, J.; Ehrlich, S.; Krieg, H. A consistent and accurate *ab initio* parametrization of density functional dispersion correction (DFT-D) for the 94 elements H-Pu. *J. Chem. Phys.* **2010**, *132*, 154104.

(18) Grimme, S.; Ehrlich, S.; Goerigk, L. Effect of the Damping Function in Dispersion Corrected Density Functional Theory. *J. Comput. Chem.* **2011**, *32*, 1456–1465.

(19) Barone, V.; Cossi, M. Quantum Calculation of Molecular Energies and Energy Gradients in Solution by a Conductor Solvent Model. *J. Phys. Chem. A* **1998**, *102*, 1995–2001.

(20) Lu, T.; Chen, F. Multiwfn: A Multifunctional Wavefunction Analyzer. *J. Comput. Chem.* **2012**, *33*, 580–592.

(21) Lu, T. A comprehensive electron wavefunction analysis toolbox for chemists, Multiwfn. *J. Chem. Phys.* **2024**, *161*, 082503.

(22) Lu, T.; Chen, F. Calculation of Molecular Orbital Composition. *Acta Chim. Sin.* **2011**, *69*, 2393.

(23) Hirata, S.; Head-Gordon, M. Time-dependent density functional theory within the Tamm–Dancoff approximation. *Chem. Phys. Lett.* **1999**, *314*, 291–299.

(24) Goddard, T. D.; Huang, C. C.; Meng, E. C.; Pettersen, E. F.; Couch, G. S.; Morris, J. H.; Ferrin, T. E. UCSF ChimeraX: Meeting modern challenges in visualization and analysis. *Protein Sci.* **2018**, *27*, 14–25.

(25) Pettersen, E. F.; Goddard, T. D.; Huang, C. C.; Meng, E. C.; Couch, G. S.; Croll, T. I.; Morris, J. H.; Ferrin, T. E. UCSF ChimeraX: Structure visualization for researchers, educators, and developers. *Protein Sci.* **2021**, *30*, 70–82.

(26) Meng, E. C.; Goddard, T. D.; Pettersen, E. F.; Couch, G. S.; Pearson, Z. J.; Morris, J. H.; Ferrin, T. E. UCSF ChimeraX: Tools for structure building and analysis. *Protein Sci.* **2023**, *32*, e4792.

(27) Mejía, E.; Luo, S.-P.; Karnahl, M.; Friedrich, A.; Tschierlei, S.; Surkus, A.-E.; Junge, H.; Gladiali, S.; Lochbrunner, S.; Beller, M. A Noble-Metal-Free System for Photocatalytic Hydrogen Production from Water. *Chem. Eur. J.* **2013**, *19*, 15972–15978.

(28) Demas, J. N.; Crosby, G. A. Measurement of Photoluminescence Quantum Yields. A Review. *J. Phys. Chem.* **1971**, *75*, 991–1024.

(29) Doettinger, F.; Kleeberg, C.; Queffélec, C.; Tschierlei, S.; Pellegrin, Y.; Karnahl, M. Rich or poor: the impact of electron donation and withdrawal on the photophysical and photocatalytic properties of copper(I) complexes. *Catal. Sci. Technol.* **2023**, *13*, 4092–4106.

(30) Kober, E. M.; Caspar, J. V.; Lumpkin, R. S.; Meyer, T. J. Application of the Energy Gap Law to Excited-State Decay of Osmium(II)-Polypyridine Complexes: Calculation of Relative Nonradiative Decay Rates from Emission Spectral Profiles. *J. Phys. Chem.* **1986**, *90*, 3722–3734.

(31) Lakowicz, J. R. *Principles of fluorescence spectroscopy*, 3. ed.; Springer: New York, NY, 2006.

(32) Spek, A. L. PLATON SQUEEZE: a tool for the calculation of the disordered solvent contribution to the calculated structure factors. *Acta Cryst.* **2015**, *C71*, 9–18.
(33) Kratzert, D.; Krossing, I. Recent improvements in DSR. *J. Appl. Crystallogr.* **2018**, *51*, 928–934.
(34) Czerwieniec, R.; Leitl, M. J.; Homeier, H. H.; Yersin, H. Cu(I) complexes – Thermally activated delayed fluorescence. Photophysical approach and material design. *Coord. Chem. Rev.* **2016**, *325*, 2–28.
(35) Deaton, J. C.; Switalski, S. C.; Kondakov, D. Y.; Young, R. H.; Pawlik, T. D.; Giesen, D. J.; Harkins, S. B.; Miller, A. J. M.; Mickenberg, S. F.; Peters, J. C. E-Type Delayed Fluorescence of a Phosphine-Supported $Cu_2(\mu\text{-}NAr_2)_2$ Diamond Core: Harvesting Singlet and Triplet Excitons in OLEDs. *J. Am. Chem. Soc.* **2010**, *132*, 9499–9508.
(36) Hofbeck, T.; Monkowius, U.; Yersin, H. Highly Efficient Luminescence of Cu(I) Compounds: Thermally Activated Delayed Fluorescence Combined with Short-Lived Phosphorescence. *J. Am. Chem. Soc.* **2015**, *137*, 399–404.
(37) Elie, M.; Weber, M. D.; Di Meo, F.; Sguerra, F.; Lohier, J.-F.; Pansu, R. B.; Renaud, J.-L.; Hamel, M.; Linares, M.; Costa, R. D.*; et al.* Role of the Bridging Group in Bis-Pyridyl Ligands: Enhancing Both the Photo- and Electroluminescent Features of Cationic (IPr)Cu^I Complexes. *Chem. Eur. J.* **2017**, *23*, 16328–16337.
